# Systems Network Integration of Transcriptomic, Proteomic, and Bioinformatic Analyses Reveals the Mechanism of XuanYunNing Tablets in Meniere’s Disease via JAK-STAT Pathway Modulation

**DOI:** 10.3390/ph18091266

**Published:** 2025-08-25

**Authors:** Zhengsen Jin, Chunguo Wang, Yifei Gao, Xiaoyu Tao, Chao Wu, Siyu Guo, Jiaqi Huang, Jiying Zhou, Chuanqi Qiao, Keyan Chai, Hua Chang, Chun Li, Xun Zou, Jiarui Wu

**Affiliations:** 1 Department of Clinical Pharmacology of Traditional Chinese Medicine, School of Chinese Materia Medica, Beijing University of Chinese Medicine, Beijing 100029, China; 2 Beijing Institute of Traditional Chinese Medicine, Beijing University of Chinese Medicine, Beijing 100029, China; 3 Guilin Sanjin Pharmaceutical Co., Ltd., Guilin 541004, China

**Keywords:** Meniere’s disease, endolymphatic hydrops, XuanYunNing tablets, transcriptomics, proteomics

## Abstract

**Background:** Meniere’s disease (MD) is a rare inner ear disorder characterized by endolymphatic hydrops and symptoms such as vertigo and hearing loss, with no curative treatment currently available. XuanYunNing tablets (XYN) have been clinically used to treat MD, but their molecular mechanisms remain unclear. **Objective:** This study aimed to systematically evaluate the pharmacological effects of XYN in a guinea pig model of MD and to elucidate the underlying molecular mechanisms of both MD pathogenesis and XYN intervention through integrated multi-omics analyses, including transcriptomics, proteomics, and bioinformatics. **Methods:** A guinea pig model of endolymphatic hydrops was induced by intraperitoneal injection of desmopressin acetate (dDAVP). Pharmacodynamic efficacy was evaluated via behavioral scoring and histopathological analysis. The differentially expressed genes (DEGs) and differentially expressed proteins (DEPs) modulated by XYN treatment were identified using high-throughput transcriptomic and proteomic sequencing. These data were integrated through multi-omics bioinformatic analysis. Key molecular targets and signaling pathways were further validated using RT-qPCR and Western blotting. **Results:** Pharmacological evaluations showed that guinea pigs in the model group exhibited a 26% increase in endolymphatic hydrops area, while high-dose XYN treatment reduced this area by 19% and significantly improved functional parameters, including overall physiological condition (e.g., weight and general appearance), auricular reflexes to low-, medium-, and high-frequency sound stimuli, nystagmus, and the righting reflex. High-throughput sequencing combined with integrative omics analysis identified 513 potential molecular targets of XYN. Subsequent network and module analyses pinpointed the JAK-STAT signaling pathway as the central axis. Mendelian randomization (MR) analysis further supported a causal relationship between MD and metabolic, immune, and inflammatory traits, reinforcing the central role of JAK-STAT signaling in both MD progression and XYN-mediated intervention. Mechanistic studies confirmed that XYN downregulated IFNG, IFNGR1, JAK1, p-STAT3/STAT3, and AOX at both mRNA and protein levels, thereby inhibiting aberrant JAK-STAT pathway activation in MD model animals. In addition, a total of 125 chemical constituents were identified in XYN by UHPLC-MS analysis. ZBTB20 and other molecules were identified as potential blood-based biomarkers for MD. **Conclusions:** This study reveals that XYN alleviates MD symptoms by disrupting a pathological cycle driven by JAK-STAT signaling, inflammation, and metabolic dysfunction. These findings support the clinical potential of XYN in the treatment of Meniere’s disease and may inform the development of novel therapeutic strategies.

## 1. Introduction

Ménière’s disease (MD) is an inner ear disorder pathologically characterized by endolymphatic hydrops. Clinically, MD exhibits considerable heterogeneity, with a classic triad comprising episodic vestibular dysfunction and progressive auditory impairment. Its hallmark symptoms include recurrent episodes of rotational vertigo lasting from 20 min to 12 h, fluctuating sensorineural hearing loss predominantly affecting low-to-mid frequencies, and cochlear symptoms such as tinnitus or aural fullness. The disease typically follows a chronic and progressive course [1,2].

Although MD was first systematically described by Prosper Ménière in 1861, its precise pathogenesis remains incompletely understood [3]. Current evidence implicates a combination of factors, including structural and functional disturbances of the inner ear, ionic imbalance, as well as inflammatory and immune-related mechanisms [4,5]. The pathological hallmark and diagnostic gold standard of MD is endolymphatic hydrops [6].

MD predominantly affects middle-aged and older adults and exerts a profound impact on their quality of life [7,8]. Available treatment modalities include pharmacological and surgical approaches. However, current interventions are largely symptomatic and do not offer a definitive cure. Thus, the development of effective therapeutic strategies remains a key focus of ongoing MD research [9,10,11].

In recent years, traditional Chinese medicine (TCM) has demonstrated unique advantages in the prevention and treatment of inner ear disorders such as vertigo, tinnitus, and hearing loss. By adhering to the principle of syndrome differentiation and treatment, TCM aligns closely with the modern medical paradigms of “precision medicine” and “personalized therapy.” It emphasizes a holistic approach, conceptually resonating with the principles of modern systems biology and aligning closely with the core tenets of network pharmacology, which integrates multi-target, multi-pathway, and multi-level analyses to elucidate the complex interactions between drugs and biological systems [12,13].

XYN is a proprietary Chinese medicine formulation composed of extracts from ten distinct medicinal herbs, selected according to traditional pharmacopeia principles. It is commonly prescribed in clinical settings to alleviate symptoms such as dizziness and tinnitus and has been applied in the treatment of MD [14,15]. However, existing clinical data are predominantly derived from studies with small sample sizes or combination therapies, and high-quality experimental evidence from basic research remains insufficient.

**Figure 1:**
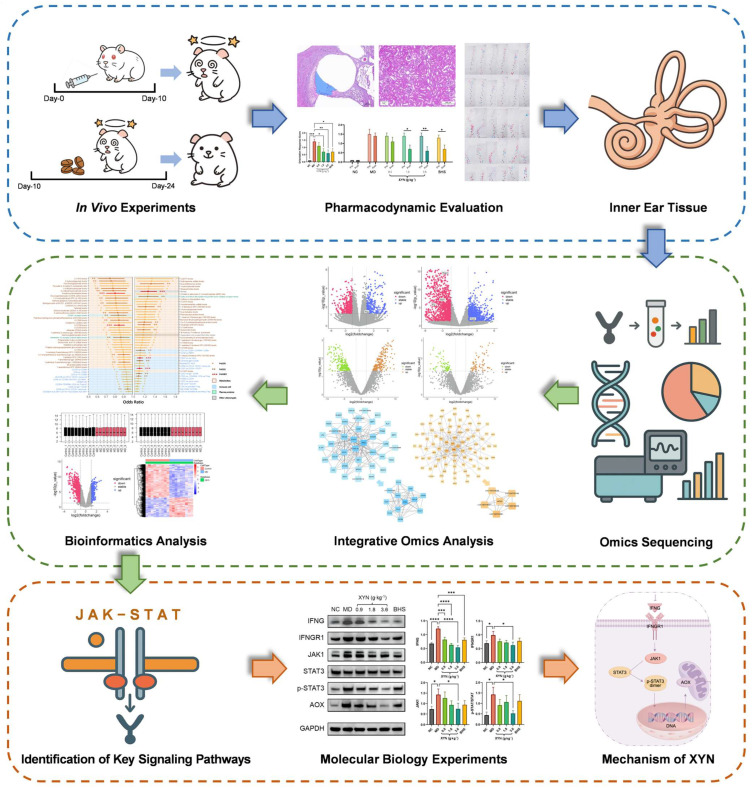
Overview of the proposed framework. Note: NC, normal control group; MD, model group; 0.9, low-dose XYN group; 1.8, medium-dose XYN group; 3.6, high-dose XYN group; BHS, positive control group treated with betahistine. * *p* < 0.05, ** *p* < 0.01, *** *p* < 0.001, **** *p* < 0.0001.

In light of the above, as shown in Figure 1, this study aims to undertake a comprehensive and systematic investigation of XYN and its potential therapeutic effects on MD. The therapeutic efficacy of XYN will first be evaluated using a guinea pig model of endolymphatic hydrops. Subsequently, transcriptomic and proteomic analyses will be employed to construct molecular regulatory networks and to identify key molecules and associated signaling pathways. Particular attention will be given to the JAK-STAT signaling pathway, which has been increasingly recognized for its pivotal role in mediating inflammation, immune responses, and cellular homeostasis—processes that are closely implicated in MD pathogenesis. This pathway also emerged as a high-confidence candidate in our integrative omics screening, supporting its selection as a focus for further mechanistic investigation. In addition, public databases and bioinformatics tools will be leveraged to preliminarily identify potential diagnostic biomarkers and risk factors for MD from an interaction network perspective.

By integrating multi-level and multi-dimensional network-based approaches, this study endeavors to provide robust experimental and theoretical evidence supporting the clinical application of XYN in MD treatment, thereby establishing a solid foundation for the development of novel, safe, and effective therapeutic strategies.

## 2. Results

### 2.1. UHPLC-MS-Based Chemical Profiling of XYN

**Figure 2:**
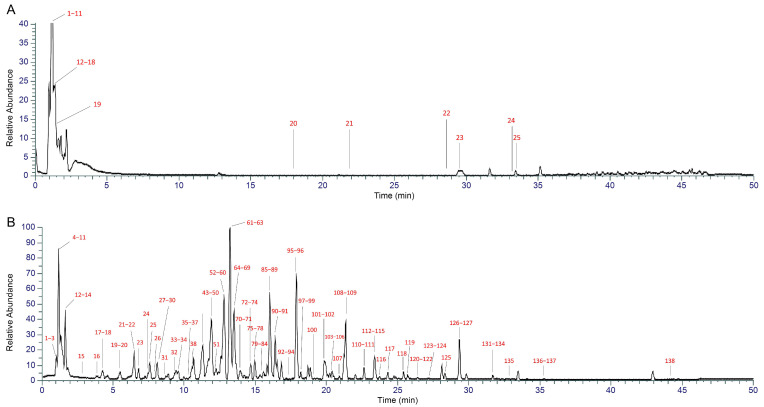
Total ion chromograms of XYN extract: (**A**) positive ion mode; (**B**) negative ion mode.

The extract of XYN was analyzed under positive ion mode. Major chromatographic peaks in the total ion chromatogram were systematically identified based on retention time, accurate mass measurements from high-resolution mass spectrometry, and MS/MS fragmentation patterns. The identification results are presented in Figure 2A. Similarly, the extract was analyzed under negative ion mode. The identification results are shown in Figure 2B.

In total, 125 compounds were identified, comprising 45 flavonoids, 29 terpenoids, 17 phenylpropanoids, 14 alkaloids, 13 organic acids, 3 coumarins, 1 lignan, 1 quinone, and 2 others. The above results were obtained through manual interpretation without the use of reference standards. Further confirmation requires the acquisition and comparison of authentic reference compounds. Detailed information is provided in the Table S1.

### 2.2. XYN Administration Alleviates Endolymphatic Hydrops and Improves Associated Symptoms

#### 2.2.1. Validation of the Endolymphatic Hydrops Model and Post-Modeling Re-Grouping

Baseline balance across groups following re-grouping is presented in the Figure S1. With the exception of the blank control group, guinea pigs in the remaining experimental groups exhibited relatively uniform distributions in terms of weight and behavioral indices, with no significant deviations observed. This indicates that re-grouping effectively eliminated individual and modeling-related differences and ensured consistent baseline conditions prior to drug administration.

In addition, guinea pigs in the model group demonstrated reduced weight and impaired behavioral performance, confirming the successful establishment of the endolymphatic hydrops model.

#### 2.2.2. XYN Attenuates Endolymphatic Hydrops in the Cochlea

**Figure 3:**
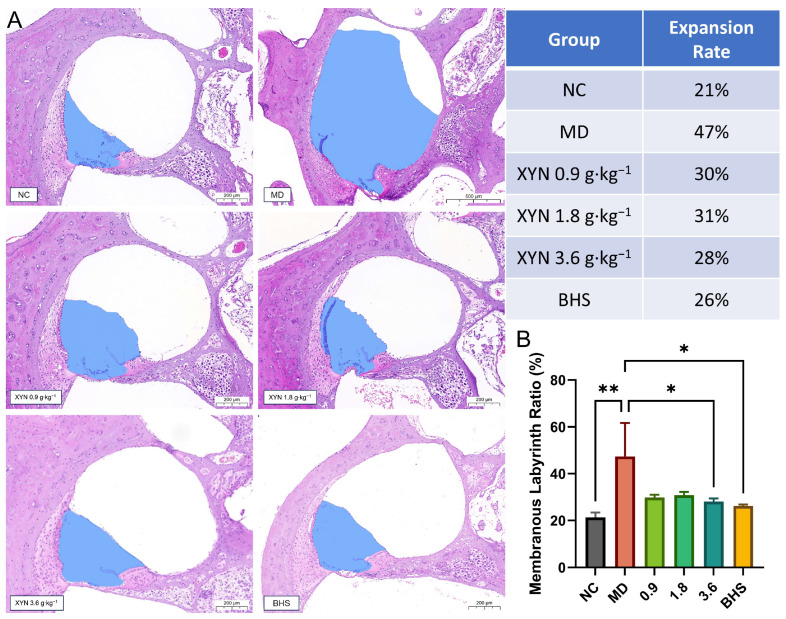
XYN alleviates endolymphatic hydrops in the first turn of the cochlear scala media. (**A**) Cross-sectional view of the endolymphatic labyrinth. The blue-shaded area indicates the endolymphatic labyrinth. (**B**) Quantitative analysis of cochlear cross-sections. The expansion rate refers to the ratio of the endolymphatic labyrinth area to the total cochlear area. Note: NC, normal control group; MD, model group; 0.9, low-dose XYN group; 1.8, medium-dose XYN group; 3.6, high-dose XYN group; BHS, positive control group treated with betahistine. * *p* < 0.05, ** *p* < 0.01; *n* = 3.

Figure 3 illustrates the degree of endolymphatic hydrops in the first turn of the cochlear scala media cross-sections across different groups. In the blank control group, the endolymphatic space accounted for approximately 21% of the cross-sectional area of the scala media. In contrast, this proportion increased to 47% in the model group. The proportions were 30%, 31%, and 28% in the low-, medium-, and high-dose XYN groups, respectively, and 26% in the betahistine (BHS)-treated group.

These findings indicate that, compared to the blank control group, guinea pigs in the model group exhibited clear enlargement of the endolymphatic space, confirming the presence of endolymphatic hydrops consistent with the pathological features of Meniere’s disease. Relative to the model group, animals in all treatment groups showed a reduction in hydrops severity, suggesting that both XYN and BHS effectively alleviated endolymphatic hydrops in guinea pigs.

#### 2.2.3. XYN Improves Gait Trajectories in Guinea Pigs

**Figure 4:**
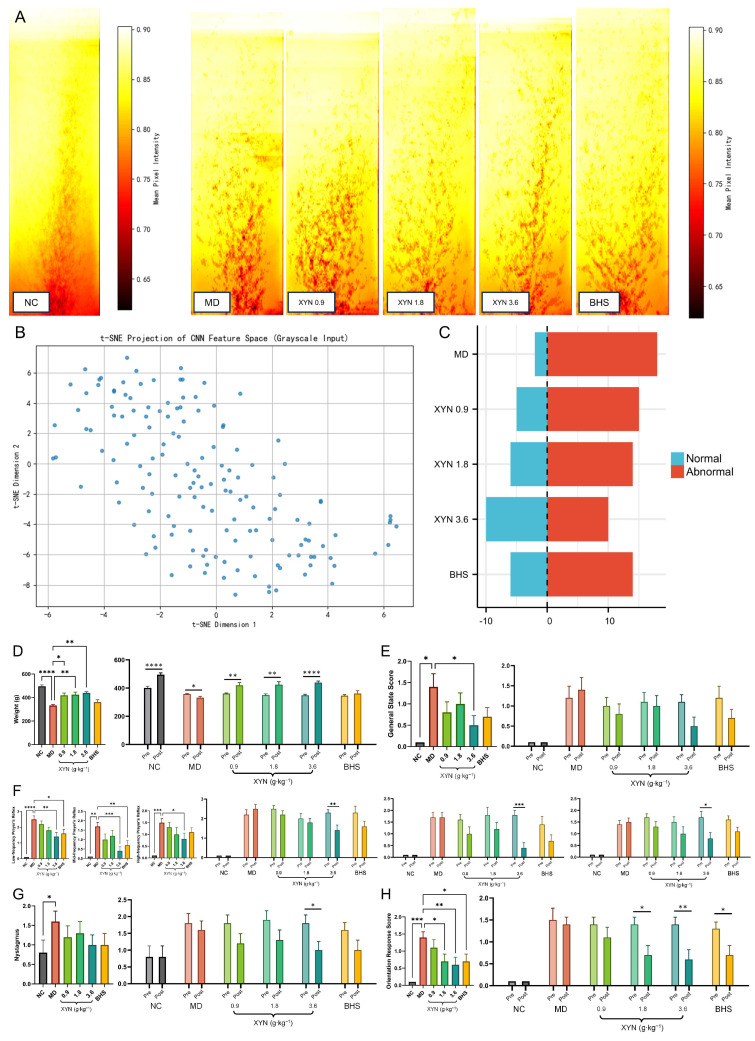
XYN improves behavioral performance in guinea pig. (**A**) Grayscale heatmaps of footprint distribution for the blank group (training set) and other groups. (**B**) t-SNE scatter plot showing dimensionality reduction and clustering. (**C**) Gait anomaly detection results based on the classification model. (**D**) Weight. (**E**) General condition. (**F**) Auricular reflex at low, medium, and high frequencies. (**G**) Nystagmus. (**H**) Righting reflex test. Note: NC, normal control group; MD, model group; 0.9, low-dose XYN group; 1.8, medium-dose XYN group; 3.6, high-dose XYN group; BHS, positive control group treated with betahistine. * *p* < 0.05, ** *p* < 0.01, *** *p* < 0.001, **** *p* < 0.0001; *n* = 10.

As shown in Figure 4A–C, gait anomaly detection was conducted using a convolutional neural network (CNN) for feature extraction combined with a one-class support vector machine (OCSVM) classifier. The results showed that in the model group, 18 out of 20 gait footprints were identified as abnormal. In contrast, the high-dose XYN group showed only 10 abnormal footprints, and all other treatment groups had fewer gait anomalies compared to the model group. These findings suggest that XYN can improve gait abnormalities to some extent in guinea pigs with endolymphatic hydrops.

#### 2.2.4. XYN Improves Behavioral Indices in Guinea Pigs Across Groups

Behavioral assessments in this study included general condition (weight and overall appearance), auricular reflexes (low-, medium-, and high-frequency stimulation), nystagmus, and righting reflex.

As shown on the left side of Figure 4D–F, the results showed that guinea pigs in the model group had significantly lower weight compared to the blank control group, while all other behavioral indices were significantly elevated, indicating more severe dysfunction. In contrast, guinea pigs in the high-dose XYN group exhibited significantly greater weight than those in the model group, along with marked improvements in all behavioral parameters.

#### 2.2.5. XYN Improves Behavioral Indicators Before and After Treatment Within the Same Guinea Pigs

Considering that individual differences among guinea pigs may influence the precision of behavioral assessments, we further analyzed changes in behavioral indices before and after treatment within the same animals.

As shown on the right side of Figure 4D–F, in the blank control group, no significant differences were observed in any behavioral indicators before and after treatment, nor were there changes in general condition scores across all groups. In the model group, no statistically significant differences were detected before and after treatment; however, there was a trend toward worsening behavioral performance. In the high-dose XYN group, post-treatment assessments showed improvement in all behavioral indicators except general condition. Other treatment groups also demonstrated varying degrees of behavioral improvement, although none matched the efficacy observed in the high-dose group.

### 2.3. The JAK-STAT Signaling Pathway as a Core Axis in the Pathogenesis of Endolymphatic Hydrops and the Therapeutic Action of XYN

#### 2.3.1. Differentially Expressed mRNAs and Proteins Associated with Endolymphatic Hydrops and XYN Intervention

**Figure 5:**
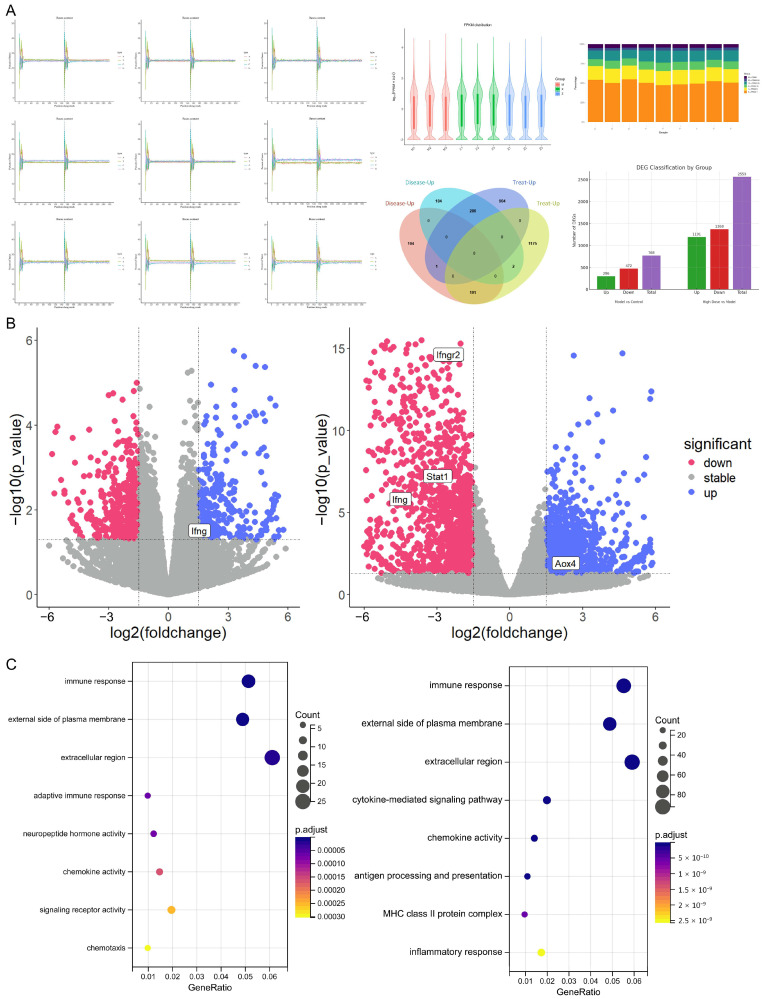
Transcriptomic analysis results. (**A**) Quality control of RNA sequencing data. (**B**) Volcano plot of DEGs. (**C**) Functional enrichment analysis of DEGs. Note: Inner ear tissues from guinea pigs were used for RNA sequencing (*n* = 3). No abnormalities were detected during quality control of the RNA sequencing data.

According to Figure 5, transcriptomic analysis revealed that, compared to the blank control group, the model group exhibited 768 DEGs, with 296 upregulated and 472 downregulated. When comparing the high-dose XYN group to the model group, 2559 DEGs were identified, including 1191 upregulated and 1368 downregulated genes.

**Figure 6:**
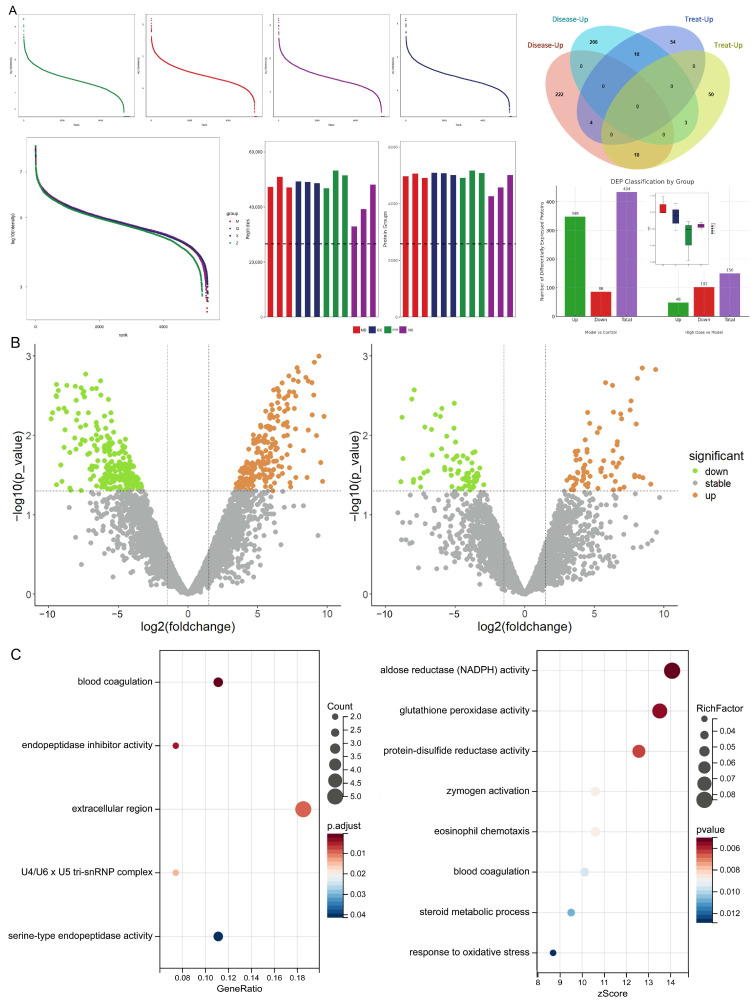
Proteomic analysis results. (**A**) Quality control of proteomic data. (**B**) Volcano plot of DEPs. (**C**) Functional enrichment analysis of DEPs. Note: Inner ear tissues from guinea pigs were used for proteomic analysis (*n* = 3). No abnormalities were detected during quality control of the proteomic data.

According to Figure 6, proteomic analysis showed 434 DEPs between the model and blank groups, with 348 upregulated and 86 downregulated. In comparison to the model group, the high-dose XYN group exhibited 150 DEPs, of which 48 were upregulated and 102 were downregulated.

GO and KEGG enrichment analyses were performed separately on DEGs and DEPs associated with disease onset and drug intervention, stratified by up- and downregulation. The enriched pathways were predominantly involved in immune response, inflammation, metabolism, and cytoskeletal regulation. Notably, both mRNA and protein datasets were significantly enriched in the cytokine–cytokine receptor interaction, JAK-STAT signaling pathway, and NF-κB signaling pathway, among others. Detailed enrichment analysis results are provided in the Figures S2–S5.

#### 2.3.2. Metabolism, Inflammation, and Immunity-Related Phenotypes Are Associated with Meniere’s Disease Onset

Given the lack of clearly defined research directions in MD, MR analysis was first performed to identify meaningful investigative targets, which subsequently guided the interpretation of high-throughput sequencing results in this study.

Based on previous research and the transcriptomic and proteomic findings outlined earlier, metabolic, immune, and inflammatory factors or pathways were prioritized for MR analysis. GWAS summary statistics for both exposures and outcomes were obtained from the Finnish biobank, with all relevant data accessed in September 2024. The Finnish data were derived from the most recent r-11 version of the database. Given the large size of the datasets, additional validation was conducted to ensure the robustness of the findings.

SNPs were selected based on a genome-wide significance threshold of *p* < 5 × 10^−6^ across all GWAS datasets, except for the immune cell phenotype dataset, where the threshold was relaxed to *p* < 1 × 10^−5^ to ensure adequate SNP inclusion for analysis. The dataset of potential related phenotypes of MD is provided in the Table S2.

A total of 2275 two-sample MR analyses were conducted, treating candidate phenotypes as exposure variables and MD as the outcome. All instrumental variables had F-statistics > 10, indicating sufficient strength. IVW analysis identified 112 exposure phenotypes with a causal relationship to MD, among which 57 phenotypes were positively associated (OR > 1, *p* < 0.05) and 55 were negatively associated (OR < 1, *p* < 0.05).

Specifically, out of 1400 metabolic traits, 75 were associated with MD. Among 731 immune cell phenotypes, 32 were associated. From 91 inflammatory circulating proteins, 3 were associated. Further analysis showed that 35 metabolic traits, 19 immune cell phenotypes, and 1 protein were positively associated with MD risk, while 40 metabolic traits, 13 immune phenotypes, and 2 proteins were negatively associated. In addition, both otitis media and anxiety disorders were found to have a significant positive correlation with MD risk.

**Figure 7:**
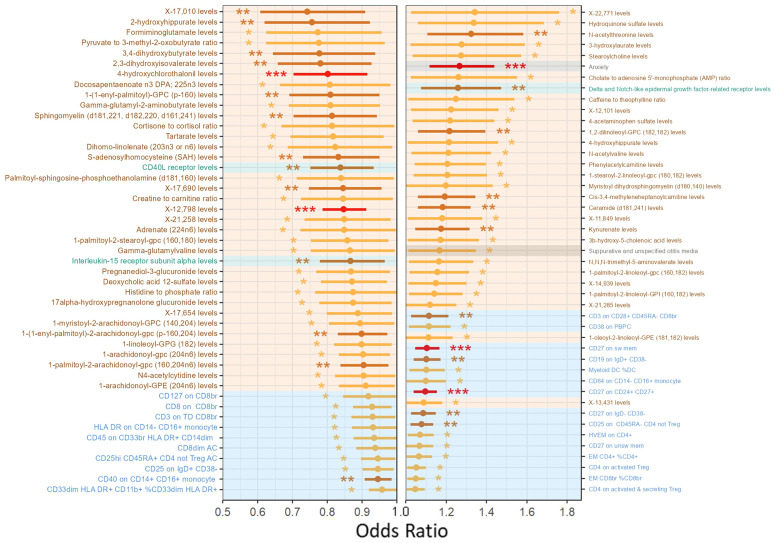
Risk factor landscape of MD. Note: 75 of 1400 metabolites, 32 of 731 immune cell phenotypes, and 3 of 91 inflammatory circulating proteins were linked to MD. Specifically, 35 metabolites, 19 immune cell phenotypes, and 1 protein showed a positive association, while 40 metabolites, 13 immune cell phenotypes, and 2 proteins were negatively associated with MD incidence. Additionally, otitis media and anxiety disorders both demonstrated a positive association with MD incidence. Sensitivity analyses revealed no heterogeneity (*p*-value of Q test > 0.05) or horizontal pleiotropy (*p*-value of MR-Egger intercept method > 0.05) among these exposure phenotypes. Bayesian-weighted MR was applied to 110 phenotypes from the top three largest datasets. This analysis identified 90 significant exposure phenotypes with consistent results across both primary MR and Bayesian-weighted MR analyses, all demonstrating the same effect direction. Including otitis media and anxiety disorders, this produced a total of 92 critical exposure phenotypes, forming the risk factor map for MD, * *p* < 0.05, ** *p* < 0.01, *** *p* < 0.001.

To enhance reliability and confirm consistency, Bayesian-weighted MR analysis was conducted on 110 phenotypes from the three largest datasets. This analysis confirmed 90 significant exposure phenotypes with consistent effect directions in both standard and Bayesian MR approaches. As shown in Figure 7, including otitis media and anxiety, a total of 92 key exposure phenotypes were identified, forming a comprehensive risk factor profile for MD. Among them, 45 phenotypes were positively associated with MD risk, and 47 were negatively associated.

Overall, metabolism-related phenotypes exerted the strongest influence on MD risk, followed by inflammatory protein and immune cell–related phenotypes.

#### 2.3.3. The JAK-STAT Signaling Pathway as a Core Axis in Endolymphatic Hydrops Pathogenesis and XYN Intervention

After stringent filtering, 191 genes were identified as pathologically upregulated in MD and downregulated following XYN treatment, whereas 286 genes exhibited the opposite trend—pathologically downregulated and pharmacologically upregulated. Similarly, 18 proteins were identified in each of the corresponding categories. Altogether, 209 molecules were classified as pathologically upregulated and pharmacologically downregulated, and 304 as pathologically downregulated and pharmacologically upregulated. These findings resulted in the identification of 513 potential molecular targets of XYN in the treatment of MD.

**Figure 8:**
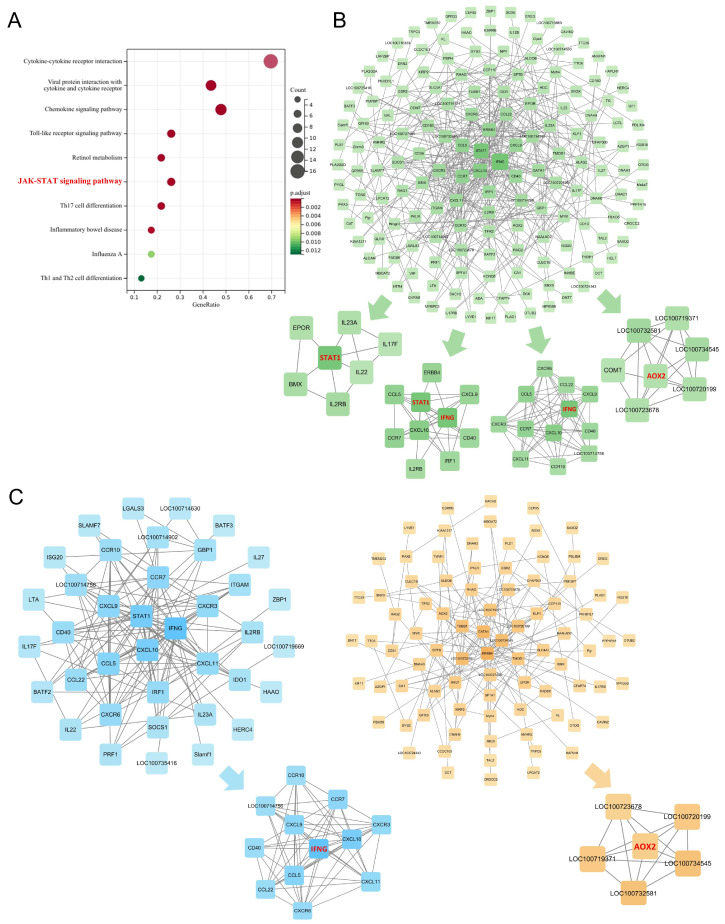
Integrated omics analysis. (**A**) KEGG enrichment analysis of potential molecular targets modulated by XYN Pian in MD. (**B**) PPI network and MCODE-derived subnetworks of potential targets. A total of 513 candidate targets were identified, including 209 molecules that were pathologically upregulated and pharmacologically downregulated, and 304 that were pathologically downregulated and pharmacologically upregulated. STAT1, IFNG, and AOX2 emerged as key nodes within the MCODE-defined subclusters. (**C**) Stratified PPI subnetworks based on differential regulation patterns. The left panel shows 209 molecules that were pathologically upregulated and pharmacologically downregulated, where MCODE analysis identified IFNG and STAT1 as core nodes. The right panel displays 304 molecules that were pathologically downregulated and pharmacologically upregulated, with AOX2 identified as a key node in the corresponding subnetwork.

As shown in Figure 8, PPI network analysis was conducted after removing unconnected nodes. The resulting PPI network for these 513 candidate molecules consisted of 161 nodes and 368 edges. Using Molecular Complex Detection (MCODE), a Cytoscape plugin that identifies highly interconnected regions based on network topology, four subnetworks were identified according to node degree and clustering scores. Key nodes included IFNG, AOX2, STAT1, and IL22. These candidate molecules were primarily enriched in the JAK-STAT signaling pathway, cytokine–cytokine receptor interaction, and NF-κB signaling pathway, highlighting their relevance to inflammatory and immune responses.

Further stratification of the PPI network into two subnetworks—based on pathological upregulation/pharmacological downregulation and vice versa—yielded the following: The 209 pathologically upregulated, pharmacologically downregulated molecules formed a subnetwork comprising 38 nodes and 136 edges. MCODE analysis identified IFNG and STAT1 as high-degree nodes, with IFNG emerging as a key molecule within the core subnetwork. The 304 pathologically downregulated, pharmacologically upregulated molecules formed a subnetwork of 86 nodes and 129 edges, where AOX2 was identified as a prominent node in the MCODE-defined core subnetwork.

Key nodes across these networks—including AOX2, IFNG, STAT1, and IL22—are well-recognized components of the JAK-STAT signaling pathway, and many (e.g., STAT and AOX family members) are closely associated with lipid metabolism. In conjunction with the MR analysis findings from Section 2.3.2, this strongly supports the JAK-STAT pathway as a central regulatory axis in both the pathogenesis of endolymphatic hydrops and the therapeutic mechanism of XYN.

#### 2.3.4. The hsa-miR-3148/ZBTB20 Regulatory Axis as a Potential Blood Biomarker for Meniere’s Disease

While findings from animal and basic research provide valuable mechanistic insights, a translational gap remains before clinical application can be achieved. Therefore, this section focuses on identifying potential blood biomarkers for MD using RNA sequencing data from clinical MD patients.

Two datasets—GSE202657 and GSE176560—were selected after initial screening for quality and relevance. In the GSE202657 dataset, 968 differentially expressed mRNAs were identified, comprising 355 upregulated and 613 downregulated transcripts. Functional enrichment analysis indicated that these dysregulated mRNAs were predominantly associated with immune system functions and fundamental cellular processes.

**Figure 9:**
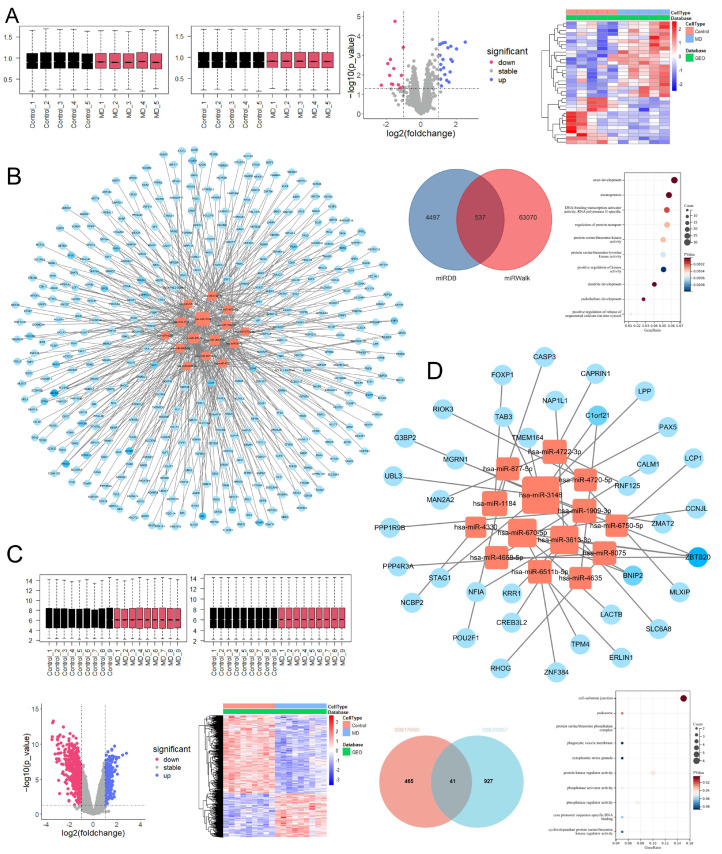
Analysis of blood biomarkers in MD patients. (**A**) Analysis and identification of differentially expressed molecules in the GSE176560 dataset; (**B**) miRNA–target interaction network based on the GSE176560 dataset; (**C**) analysis and identification of differentially expressed molecules in the GSE202657 dataset; (**D**) refined miRNA–mRNA regulatory network constructed from both datasets.

As shown in Figure 9, from the GSE176560 dataset, 34 differentially expressed miRNAs were identified, of which 21 were upregulated and 13 were downregulated. These miRNAs were analyzed using the miRDB and miRWALK databases, yielding 506 predicted target mRNAs. Functional enrichment analysis indicated that these targets were mainly associated with protein function, immune regulation, and developmental pathways.

The 34 differentially expressed miRNAs and their predicted target mRNAs were imported into Cytoscape to construct a miRNA–mRNA regulatory network, after removing isolated nodes and unconnected subnetworks. The resulting network consisted of 442 nodes and 457 edges, including 15 miRNAs and 427 mRNAs. Among these, hsa-miR-3148 had the highest node degree among miRNAs, and ZBTB20 had the highest node degree among mRNAs.

Intersecting the mRNA lists from both datasets led to the identification of 41 core mRNAs associated with MD pathogenesis. A refined miRNA–mRNA regulatory network was then constructed using these 41 mRNAs and the 34 differentially expressed miRNAs from GSE176560, excluding isolated nodes. The resulting network comprised 50 nodes and 41 edges, including 14 miRNAs and 36 mRNAs. Notably, hsa-miR-3148 and ZBTB20 remained the highest-degree nodes in the network, and a regulatory interaction between them was identified.

Importantly, ZBTB20 was also found to be significantly differentially expressed in both the transcriptomic and proteomic profiles of the guinea pig model in this study, further supporting its potential relevance as a clinical blood biomarker for MD.

### 2.4. Overactivation of the JAK-STAT Signaling Pathway in MD and Its Suppression by XYN Treatment

**Figure 10:**
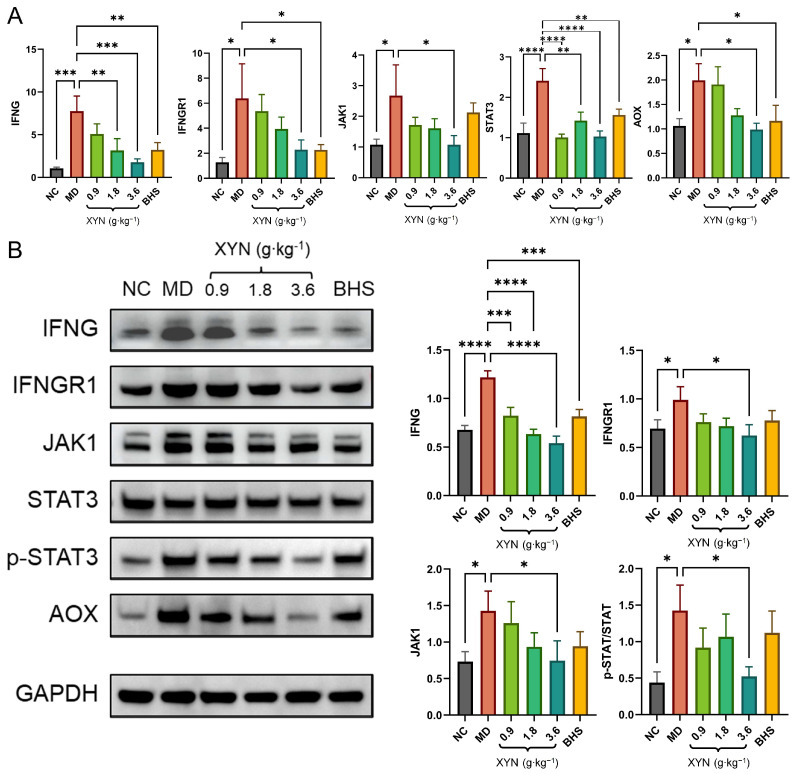
Molecular validation of the JAK-STAT signaling pathway. (**A**) Relative mRNA expression levels of key components in the JAK-STAT pathway; (**B**) Western blot bands and relative protein expression levels of corresponding molecules. Note: NC, normal control group; MD, model group; 0.9, low-dose XYN group; 1.8, medium-dose XYN group; 3.6, high-dose XYN group; BHS, positive control group treated with betahistine. * *p* < 0.05, ** *p* < 0.01, *** *p* < 0.001, **** *p* < 0.0001; *n* = 6.

As shown in Figure 10, RT-qPCR results revealed that components of the JAK-STAT signaling pathway were significantly overactivated at the transcriptional level in the model group, with statistically significant differences observed. Treatment with XYN or BHS led to varying degrees of suppression of this pathway. IFNG and STAT3 expression levels were significantly downregulated in the medium- and high-dose XYN groups, as well as the BHS-treated group; IFNGR1 and AOX were significantly downregulated in the high-dose XYN group and the BHS group; JAK1 expression was significantly reduced only in the high-dose XYN group.

Western blot results similarly demonstrated overactivation of JAK-STAT pathway proteins in the model group, with significant differences compared to controls. Treatment with XYN and BHS resulted in suppression of these protein expressions to varying extents. Expression levels of IFNG and AOX were significantly downregulated in all treatment groups; However, expression of IFNGR1, JAK1, and phosphorylated STAT/total STAT ratio (p-STAT/STAT) was significantly downregulated only in the high-dose XYN group. While other groups showed a downward trend, these changes were not statistically significant.

The sequences of primers and the details of antibodies used in the experiments are provided in Table 1 and Table 2. Statistical summaries of significant differences in gene and protein expression levels across experimental groups are presented in Table 3 and Table 4. For data that did not reach statistical significance, sensitivity analyses were not conducted.

## 3. Discussion

Traditional Chinese medicine (TCM) offers a distinctive perspective on the pathogenesis of vertigo. Classical TCM teachings suggest that “no vertigo occurs without phlegm” and “no vertigo occurs without deficiency,” highlighting that impaired blood circulation, meridian obstruction, and failure of blood to nourish the brain are key mechanisms underlying the condition [16]. In the context of modern TCM theory, MD is classified under the syndromic categories of “Xuan Yun” (vertigo) and “Er Xuan Yun” (aural vertigo) [16,17,18,19]. Its core pathogenesis reflects a “root deficiency with branch excess” pattern [20,21,22].

Notably, the pathological hallmark of MD—endolymphatic hydrops—aligns closely with the TCM concept of “phlegm–fluid retention (tan yin)”, which is believed to result from dysfunction in San Jiao (the Triple Energizer) and disrupted fluid metabolism [23].

XYN, composed of *Alisma plantago-aquatica* subsp. orientale Sam., *Atractylodes macrocephala* Koidz., *Wolfiporia cocos* (F.A. Wolf) Ryvarden & Gilb., *Pinellia ternate* (Thunb) Breit., *Ligustrum lucidum* Ait., *Eclipta prostrata* L., *Chrysanthemum morifolium* Ramat., *Achyranthes bidentata* Blume., *Citrus reticulata* Blanco., and *Glycyrrhiza uralensis* Fisch. (the plant name has been checked with http://www.worldfloraonline.org, accessed on 11 May 2025), was supplied by Guilin Sanjin Pharmaceutical Co., Ltd. (Guilin, China), batch number 230902. XYN exerts spleen-strengthening, dampness-draining, kidney-nourishing, and liver-pacifying effects. It is particularly effective for treating vertigo syndromes related to phlegm-dampness obstruction and deficiency of the liver and kidney [24,25], making it a promising candidate for the clinical management of MD. Clinical studies involving hundreds of patients have demonstrated that XYN provides a statistically significant higher total effective rate in treating MD compared to control treatments, suggesting its potential to effectively alleviate MD-related symptoms [14,15,25]. *Atractylodes macrocephala* has been reported to modulate the JAK-STAT signaling cascade as part of its synergistic mechanism in treating ulcerative colitis [26]. Network pharmacology studies have revealed that Citrus reticulata exerts therapeutic effects through targets significantly enriched in the JAK-STAT and related immune pathways [27]. Moreover, *Pinellia ternate* (Thunb) has been shown to activate the phosphorylation of JAK2, STAT1, STAT4, and STAT5 in a dose- and time-dependent manner, contributing to its immunomodulatory and antitumor activities [28]. These findings suggest that the JAK-STAT regulatory effects of XYN may be partly attributed to the concerted actions of its monomeric components.

Mechanistic studies on MD predominantly rely on the guinea pig model of endolymphatic hydrops, which can be reliably induced by arginine vasopressin (AVP) or its analog dDAVP [29]. Behavioral tests such as the rotating platform test and auricular reflex assessments are commonly used to evaluate vertigo and hearing impairment [9,10]. In this study, a guinea pig model of endolymphatic hydrops was successfully constructed and validated through cochlear pathology and multiple behavioral indicators. The therapeutic efficacy of XYN was comprehensively assessed in this model.

By integrating high-throughput sequencing with MR analysis, the JAK-STAT signaling pathway was identified as a central molecular axis involved both in the pathogenesis of endolymphatic hydrops and the therapeutic mechanism of XYN.

There is accumulating evidence supporting the immune-inflammatory mechanism underlying MD. In a subset of patients, anti-inner ear antibodies and circulating immune complexes have been detected in the serum [30]. Moreover, levels of inflammatory mediators such as interleukin-1β, IL-1 receptor antagonist, IL-6, heat shock protein 70, and tumor necrosis factor-α have been shown to correlate closely with hearing loss severity and disease progression [31,32]. In addition, total serum IgE is commonly elevated in MD patients and is positively associated with the grade of endolymphatic hydrops, the stage of hearing loss, and functional impairment scores [33,34,35].

At the molecular level, the JAK-STAT signaling pathway serves as a central node in cytokine-mediated inflammatory responses. It is composed of ligand–receptor pairs such as IFNG, members of the JAK family, and STATs [36,37]. Upon binding of pro-inflammatory cytokines like IFNG to their receptors, JAKs are activated and phosphorylate STATs, which in turn promote the polarization of M1-type macrophages and amplify the production of pro-inflammatory mediators [38,39]. Conversely, inhibition of this pathway has been shown to significantly reduce inflammation and tissue damage [40,41].

These findings suggest that humoral immune dysregulation and cytokine imbalance act synergistically, driving JAK-STAT-dependent inflammatory cascades, which represent a fundamental pathological basis for the progression and fluctuation of MD symptoms.

The JAK-STAT signaling pathway plays a pivotal role not only in immune and inflammatory regulation, but is also closely associated with a variety of metabolic disorders, including obesity, insulin resistance, non-alcoholic fatty liver disease, and atherosclerosis [42,43]. In the context of MD, metabolic disturbances may contribute to disease pathogenesis by disrupting the local inner ear environment. Specifically, lipid metabolism imbalance—such as cholesterol deposition or altered fatty acid composition—may impair the osmoregulatory function of the endolymphatic sac, potentially triggering or exacerbating endolymphatic hydrops [44,45].

The AOX family plays a key role in maintaining the homeostasis of omega-3 polyunsaturated fatty acids (e.g., DHA), thereby regulating membrane fluidity and signal transduction in inner ear cells, as well as alleviating local inflammation [46].

Notably, chronic activation of the JAK-STAT pathway in MD may exacerbate cochlear damage by amplifying oxidative stress. For example, STAT3 promotes the expression of NADPH oxidase complexes, leading to elevated reactive oxygen species levels. In parallel, peroxidation byproducts of lipid metabolism, such as 4-hydroxy-2-nonenal, require detoxification via the AOX system. However, this detoxification process itself generates additional oxidative species, such as superoxide and hydrogen peroxide, thereby further increasing oxidative burden [47,48]. Excessive ROS accumulation compromises the cochlea’s antioxidant defense mechanisms, resulting in mitochondrial DNA damage and structural impairment of stereocilia proteins in sensory hair cells—lesions that are strongly associated with the sensorineural hearing loss observed in MD [49]. Additionally, ROS can oxidatively modify JAK kinases and their negative regulatory factors, disrupting feedback inhibition, sustaining JAK-STAT activation, and forming a positive feedback loop between oxidative stress and inflammation.

Although this study focused on the JAK-STAT signaling pathway as a central regulatory axis, other highly enriched pathways identified in our analysis may also hold significant relevance. Previous research has suggested that genetic alterations in Toll-like receptor pathways and cytokine-related signaling—particularly involving interleukin-1β and tumor necrosis factor-α—are closely associated with the inflammatory processes observed in Meniere’s disease [50,51]. These findings highlight the potential importance of broader immune and inflammatory networks beyond JAK-STAT signaling. In addition, both the observed therapeutic effects in the guinea pig model and the proposed mechanisms of XYN action should be further corroborated using independent human datasets.

This study also explored potential biomarkers in the blood of MD patients. Notably, ZBTB20 deficiency has been shown to significantly reduce the endocochlear potential, disrupt the high-potassium/low-sodium environment of the endolymph, and induce hydrops by impairing fluid homeostasis [52,53]. ZBTB20 also suppresses the differentiation of root cells and the spiral prominence in the lateral wall, thereby reducing the expression of ion and water transport proteins such as Na^+^/K^+^-ATPase and AQP4, exacerbating fluid accumulation. Additionally, ZBTB20 is involved in the suprachiasmatic nucleus-driven circadian rhythm, and its deficiency has been linked to circadian disruption and hearing loss [54]. In immune cells, ZBTB20 also regulates the NF-κB pathway, and its deficiency can contribute to chronic low-grade inflammation [55]. These findings suggest that blood-based biomarkers such as ZBTB20 may hold promise for the clinical diagnosis or early screening of MD.

Despite the promising findings presented in this study, several limitations should be acknowledged. Currently, there is a significant lack of fundamental research on MD, and data concerning the effects of XYN on human tissues or patients remain extremely limited. As a result, no suitable external datasets are presently available for independent validation of our findings. Future studies will aim to investigate the mechanistic basis and therapeutic relevance of XYN using human-derived tissues and well-characterized clinical samples.

## 4. Materials and Methods

### 4.1. Component Identification Results of XYN

Ultra-high performance liquid chromatography coupled with high-resolution mass spectrometry was employed for the chemical analysis. Chromatographic separation was carried out on a Waters ACQUITY UPLC BEH C18 column (100 mm × 2.1 mm, 1.7 μm; Dublin, Ireland) using a Vanquish UHPLC system (Thermo Scientific, Waltham, MA, USA) equipped with a binary gradient pump, autosampler, column oven, and diode array detector. The mobile phase consisted of acetonitrile (A) and 0.1% formic acid in water (B), with the following gradient program: 0–2 min, 95% B; 2–42 min, 95% to 10% B; 42–44 min, 10% B; 44–45 min, 10% to 95% B; 45–50 min, 95% B. The flow rate was set at 0.3 mL/min, the injection volume was 3 μL, and the column temperature was maintained at 35 °C.

Mass spectrometric detection was performed on a Q-Exactive Orbitrap quadrupole-Orbitrap mass spectrometer (Thermo Scientific, USA) equipped with a heated electrospray ionization source and controlled by Xcalibur 4.1 software. In positive ion mode, the spray voltage was 3.5 kV, with the ion source temperature set at 350 °C, capillary temperature at 300 °C, and the S-Lens RF level at 60 V. Sheath and auxiliary gas (nitrogen, >99.99%) were supplied at 40 and 20 arbitrary units, respectively. In negative ion mode, the spray voltage was 3.2 kV, with sheath and auxiliary gas flow rates adjusted to 35 and 10 units, respectively. Data were acquired in both full-scan (*m*/*z* 200–2000) and data-dependent MS/MS modes, with resolving powers of 70,000 for full scan and 17,500 for MS/MS. Fragmentation was achieved using higher-energy collisional dissociation with a normalized collision energy of 30 and stepped NCE of 50%.

For sample preparation, XYN were finely pulverized, and 1.0 g of powder was accurately weighed into a stoppered conical flask. Methanol (50 mL) was added, and the mixture was subjected to ultrasonic extraction for 40 min. After cooling, the extract was weighed again and adjusted with methanol to compensate for solvent loss. The solution was thoroughly mixed, filtered, and passed through a 0.22 μm microporous membrane prior to UHPLC-HRMS analysis.

### 4.2. Experimental Animals

Male albino Hartley guinea pigs, weighing 250 ± 10 g, were obtained from Beijing Fangyuan Yuan Breeding Facility (License No. SCXK [Beijing] 2020-0001; Animal Quality Certificate No. 110334241100018953). All experiments were conducted at the Laboratory Animal Center of Beijing University of Chinese Medicine (Facility License No. SCXK [Beijing] 2023-0011), with ethical approval granted by the Institutional Animal Care and Use Committee of the university (Approval No. BUCM-2024022707-1091).

Animals were housed under controlled environmental conditions: temperature 18–22 °C, relative humidity 40–60%, and a 12 h light/dark cycle. All procedures involving animal care and use were conducted in strict accordance with the Regulations for the Administration of Laboratory Animals (China), as well as the guidelines and requirements of the Animal Ethics Committee of Beijing University of Chinese Medicine.

### 4.3. Pharmacodynamic Evaluation Based on Endolymphatic Hydrops Model

#### 4.3.1. Establishment of the Guinea Pig Endolymphatic Hydrops Model

Intraperitoneal injection of dDAVP induces endolymphatic hydrops, leading to clinical manifestations such as vertigo and hearing loss, with minimal invasiveness and high safety [56,57]. Systemic administration of dDAVP has been successfully employed to model endolymphatic hydrops in experimental animals including rats, mice, and guinea pigs [58].

The modeling agent dDAVP was prepared at doses of 4 μg/kg and 8 μg/kg to generate low- and high-concentration solutions at 0.04 μg/μL and 0.08 μg/μL, respectively. Solutions were stored at −20 °C until use.

After a 7-day acclimation period, guinea pigs were intraperitoneally injected with the low-concentration dDAVP solution at a dose of 1 mL/kg daily for 7 consecutive days, followed by the high-concentration dDAVP solution at the same dose for 3 consecutive days. This protocol was used to induce endolymphatic hydrops in the guinea pigs.

#### 4.3.2. Behavioral Assessments

Behavioral performance of the guinea pigs was evaluated using several criteria, including general condition, auricular reflex, presence of nystagmus, and righting reflex. Scoring was performed according to the standards outlined in Table 5. Detailed scoring criteria are provided in the Table S3.

#### 4.3.3. Re-Grouping and Drug Administration

To account for individual variability among guinea pigs and subtle differences introduced during the modeling process, animals with successfully induced endolymphatic hydrops were re-grouped to balance baseline characteristics and ensure the scientific rigor and reliability of experimental outcomes. Each group (excluding the blank control group) was balanced in terms of weight, auricular reflex, nystagmus, and righting response to minimize bias caused by group-level deviations.

Animals were randomly assigned into six groups (*n* = 10 per group): model group, low-dose XYN group (0.9 g/kg), medium-dose XYN group (1.8 g/kg), high-dose XYN group (3.6 g/kg), positive control group treated with BHS (22.5 mg/kg), and blank control group. These dosing levels were preliminarily assessed in our pilot study, which indicated that they were well tolerated in guinea pigs without signs of distress or adverse effects. Drug or placebo treatments were administered once daily by oral gavage for 14 consecutive days. The blank and model groups received an equivalent volume of normal saline.

Following the treatment period, behavioral assessments were conducted as described in Section 4.3.2. Afterward, guinea pigs were anesthetized, and cochlear and renal tissues were harvested for subsequent analyses.

### 4.4. High-Throughput Sequencing and Bioinformatics Analysis

#### 4.4.1. Transcriptomic and Proteomic Analysis

Total RNA was extracted from guinea pig cochlear tissue and used for transcriptome library preparation. The libraries were sequenced on the Illumina NovaSeq 6000 platform, generating 150 bp paired-end reads. Data obtained from the Illumina (or BGI) platform were subsequently used for bioinformatics analysis.

For proteomic analysis, total protein was extracted from cochlear tissues. Peptides from each sample were analyzed using an Orbitrap™ Astral™ mass spectrometer (Thermo Scientific) coupled with a Vanquish Neo liquid chromatography system (Thermo Scientific) in data-independent acquisition (DIA) mode. DIA data were processed using DIA-NN version 1.8.1.

Both transcriptomic and proteomic data were aligned to the reference genome Cavia porcellus (NCBI Genome Assembly mCavPor4.1, https://www.ncbi.nlm.nih.gov/datasets/genome/-GCF_034190915.1/, accessed on 5 November 2023).

For downstream proteomic analysis, raw expression values were log2-transformed with a + 1 offset and linearly scaled to the range of 1–100. Differential expression analysis for both mRNA and proteins was performed using the limma package in R. Differentially expressed genes and proteins were defined based on a threshold of |log_2_ fold change (log_2_FC)| > 1.5 and *p* < 0.05, with multiple testing correction performed using the Benjamini–Hochberg method.

Gene Ontology (http://geneontology.org/, accessed on 5 March 2024) and Kyoto Encyclopedia of Genes and Genomes (https://www.kegg.jp/, accessed on 5 March 2024) enrichment analyses were carried out using the clusterProfiler package in R. Enrichment was performed against the background gene set from NCBI (GCF_034190915.1-RS_2024_02, https://www.ncbi.nlm.nih.gov/datasets/gene/GCF_034190915.1/, accessed on 5 November 2023). Biological categories with *p* < 0.05 were considered significantly enriched.

#### 4.4.2. Integrated Omics Analysis

To comprehensively identify key molecular targets, DEGs and DEPs were merged and categorized into two molecular subsets: (1) molecules upregulated under pathological conditions and downregulated after pharmacological treatment, (2) molecules downregulated under pathological conditions and upregulated following treatment. These subsets were considered potential molecular targets of XYN in the intervention of MD.

Each of the two molecular subsets, as well as their combined set, was imported into the STRING database (https://cn.string-db.org/, accessed on 5 March 2024) for protein–protein interaction (PPI) analysis. A high-confidence interaction score threshold (≥0.7) was applied, and isolated nodes were hidden to enhance interpretability. The resulting PPI networks were visualized using Cytoscape (version 3.9.1). To identify densely connected regions potentially representing functional protein complexes or signaling modules, the Molecular Complex Detection (MCODE) plugin was employed. MCODE clustering was performed across the entire network using the following parameters: Degree Cutoff = 2, Node Score Cutoff = 0.2, K-Core = 2, and Max Depth = 100. The Haircut option was enabled to eliminate singly connected peripheral nodes from clusters, while the Fluff option was disabled to maintain strict cluster boundaries. Loops were included in the analysis. Core candidate molecules were subsequently screened based on node degree and their presence within MCODE-defined subnetworks.

#### 4.4.3. Analysis of Risk Factors for Meniere’s Disease

This study conducted an analysis based on publicly available genome-wide association study (GWAS) datasets, strictly adhering to the STROBE-MR (Strengthening the Reporting of Observational Studies in Epidemiology Using MR) guidelines to ensure methodological rigor and reliability of results [54]. All data used were ethically approved and obtained with informed consent; no personal or identifiable information was involved.

A comprehensive search was conducted in the PubMed database for factors associated with the onset of MD, using keywords such as “Meniere’s disease etiology” and “risk factors for Meniere’s disease.”

Inclusion criteria required articles to directly address the etiology, pathophysiology, or risk factors of MD. Eligible literature types included scientific papers that contribute to understanding MD’s etiology, specifically original research articles, review articles, and pertinent case reports.

Exclusion criteria eliminated articles unrelated to MD etiology, including those focused exclusively on treatment, prognosis, or other otologic disorders, as well as case reports lacking substantial etiological insights. Additionally, low-quality studies—such as non-peer-reviewed articles, conference abstracts, studies with insufficient methodological rigor, and duplicate publications—were excluded.

To retrieve relevant GWAS data, we searched databases such as the Finnish Database, GWAS Catalog, and PubMed for studies on associated risk factors and collected data on potential exposure phenotypes and outcomes related to MD.

To comprehensively examine the relationship between exposure phenotypes and MD across all GWAS datasets, we applied a genome-wide significance threshold of 5 × 10^−6^. The F-statistic for each SNP was calculated to assess instrument strength, excluding any SNPs with an F-statistic below 10 to mitigate weak instrument bias.

To address linkage disequilibrium (LD) among SNPs and enhance the robustness of the analysis, we utilized reference data from the 1000 Genomes Project (https://github.com/mrcieu/gwasvcf/, accessed on 10 August 2024) as the reference panel for PLINK, the selected tool for LD calculation. Parameters were set to a 10,000 kb distance between SNPs, an r threshold of 0.001, and p1 and p2 thresholds set to 1. After merging exposure and outcome data, palindromic SNPs were excluded, leaving the remaining SNPs as the final IVs.

All statistical analyses were conducted in R (version 4.4.1). The primary MR analysis method was inverse variance weighted (IVW), implemented using the TwoSampleMR package in R. IVW is a meta-analysis-based approach that combines the Wald ratios of individual SNPs by weighting them inversely to their variance, assuming all instruments are valid and there is no horizontal pleiotropy. Post-analysis, heterogeneity was assessed via Cochran’s Q test, where a *p*-value above 0.05 suggests no heterogeneity among the IVs. Horizontal pleiotropy was evaluated with the MR-Egger method, and a leave-one-out approach was employed to assess whether causal effects were impacted by any individual SNP.

Results are reported as odds ratios (ORs) with 95% confidence intervals (CIs). In this study, *p*-values were interpreted as indicators of evidence strength, with statistical significance set at *p* < 0.05. Visualizations were created using the R packages ggplot2 and ComplexUpset.

#### 4.4.4. Analysis of Blood Biomarkers in Meniere’s Disease Patients

The Gene Expression Omnibus (GEO) database (https://www.ncbi.nlm.nih.gov/geo/, accessed on 10 August 2024) was queried using the keyword “Ménière disease,” and studies involving human blood samples were selected and screened for relevance. Both miRNA and mRNA datasets underwent preprocessing to ensure data quality and suitability for analysis.

Initial preprocessing steps included the removal of entries with missing values or duplicate data points. Subsequently, the data were normalized to bring all variables within a comparable range, facilitating reliable downstream analyses. Genes with missing expression values or duplicates were removed from the expression matrix, and those with expression levels below 5 were filtered out.

Differential expression analysis of miRNAs and mRNAs was performed using the limma package in R. Significant differentially expressed molecules were identified based on the criteria |log_2_ fold change (log_2_FC)| > 1.0 and adjusted *p* < 0.05, with *p*-values corrected using the Benjamini–Hochberg method.

Target mRNAs regulated by differentially expressed miRNAs were predicted using the miRDB (https://mirdb.org/, accessed on 15 August 2024) and miRWALK (http://mirwalk.umm.uni-heidelberg.de/, accessed on 15 August 2024) databases. The intersection of predicted targets was defined as core mRNAs implicated in MD pathogenesis. These core mRNAs were then used to construct a miRNA–mRNA regulatory network associated with MD, visualized using Cytoscape.

### 4.5. Molecular Biology Experiments

To validate the expression of the key pathways and core molecules identified in the preceding analyses, RT-qPCR and Western blot assays were performed.

For RT-qPCR, total RNA was reverse transcribed using the ReverTra Ace qPCR RT Kit. The resulting cDNA was mixed with DEPC-treated water, target gene primers, and SYBR Green reagent to prepare a 10 μL reaction system. Amplification was carried out using a three-step PCR protocol, followed by analysis of melting and amplification curves. Relative expression levels of target genes were calculated using the 2^−ΔΔCt^ method.

For Western blotting, total protein concentrations in each group were quantified using the BCA assay and adjusted to uniform levels with PBS. Protein samples were separated on 10% SDS-PAGE gels, loaded by group, and transferred to NC membranes using a wet-transfer sandwich method. Membranes were blocked with BSA at room temperature for 1.5 h, then incubated with primary antibodies overnight at 4 °C. After washing with TBST five times, membranes were incubated with secondary antibodies at room temperature for 1.5 h, followed by another five washes with TBST. Protein bands were visualized using ECL detection reagents on a chemiluminescent imaging system.

Band intensities were quantified using ImageJ software 1.50i. The relative expression of target proteins was calculated as the ratio of the target protein band intensity to that of the internal reference protein.

### 4.6. Statistical Analysis

All statistical analyses were conducted using R software (version 4.4.1). For MR, the primary analytical method employed was IVW analysis, implemented via the TwoSampleMR package. To assess the reliability of the causal estimates, Cochran’s Q test was used to evaluate heterogeneity among IVs, with *p* > 0.05 indicating no significant heterogeneity.

MR-Egger regression was applied to detect horizontal pleiotropy, and leave-one-out sensitivity analysis was used to determine whether the causal effect estimates were unduly influenced by any single SNP. Results were presented as odds ratios with 95% confidence intervals, and statistical significance was set at *p* < 0.05.

Data visualization was performed using the ggplot2 package in R to intuitively display the analytical outcomes. Quantitative results were expressed as the mean ± standard error of the mean. One-way ANOVA followed by Tukey’s multiple comparison test was used to analyze differences among multiple groups.

## 5. Conclusions

**Figure 11:**
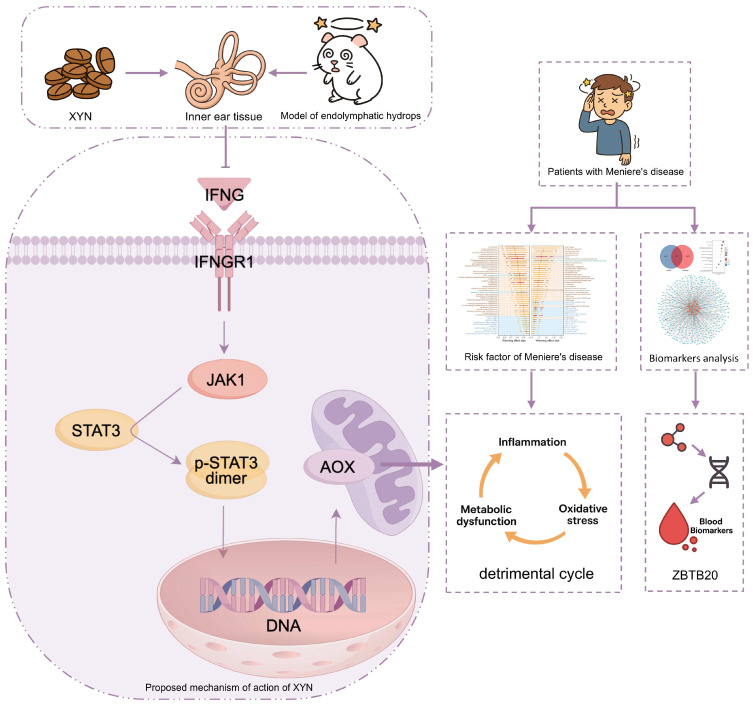
Schematic overview of the therapeutic mechanism of XYN and omics-based insights in MD. * *p* < 0.05, ** *p* < 0.01, *** *p* < 0.001.

This study, for the first time, systematically integrates transcriptomics, proteomics, animal experiments, and bioinformatics approaches to investigate the pathogenesis of MD and elucidate the therapeutic mechanisms of XYN from the perspective of molecular interaction networks. As shown in Figure 11, XYN administration alleviated endolymphatic hydrops in guinea pig models, accompanied by improvements in behavioral abnormalities such as vertigo and hearing loss, as confirmed by functional assessments and cochlear histopathology. Multi-omics and mechanistic analyses revealed dysregulation of several signaling pathways in MD, among which aberrant activation of the JAK-STAT pathway plays a central role by promoting a vicious cycle of inflammation, metabolic dysfunction, and oxidative stress. XYN intervention was shown to disrupt this pathological loop, thereby mitigating MD symptoms. ZBTB20 and other identified molecules emerged as potential blood-based biomarkers for the diagnosis and monitoring of MD.

In summary, these findings provide both experimental and theoretical support for the clinical application of XYN in MD treatment and contribute to the development of novel therapeutic strategies. Moreover, this integrative research framework offers valuable insights for exploring the role of traditional Chinese medicine in managing refractory and multifactorial diseases.

## Figures and Tables

**Table 1:** Primer information.

Gene	Primer Direction	Sequence (5′-3′)	Length
GAPDH	Forward	ATCAAGTGGGGTGATGCTGG	20
	Reverse	AAGATGCCTTTGAGGGAGCC	20
IFNG	Forward	AAGACAACAGCAGCAACAAGG	21
	Reverse	ACATGCTCGTCATTGACCGAAA	20
IFNGR1	Forward	GTGAACAGAAGTGAGACCCGA	21
	Reverse	AGAATACAGCAAGGGCACCG	20
JAK1	Forward	CCATGATGCGGAACATGCAG	20
	Reverse	GTTAATGCTGACCAGGGCCT	20
STAT3	Forward	CACCACAAAAGTCAGGCTGC	20
	Reverse	ACAGCCTGAGTAGTTCACGC	20
AOX	Forward	TCCCAGCTGGTGATCGAGTA	20
	Reverse	ACGGTGCTGGTTTCAGAGAG	20

**Table 2:** Antibody information.

Antibody	Catalog No.	Manufacturer	Dilution Ratio
HRP-conjugated Goat Anti-Rabbit IgG(H + L)	SA00001-2	Proteintech (Rosemont, IL, USA)	1:50,000
HRP-conjugated Goat Anti-Mouse IgG(H + L)	SA00001-1	Proteintech	1:10,000
STAT3 Rabbit Monoclonal antibody	A19566	ABclonal Technology (Wuhan, China)	1:10,000
Phospho-STAT3-Y705 Rabbit Monoclonal antibody	AP1468	ABclonal Technology	1:5000
IFN-gamma Rabbit Monoclonal antibody	A25684	ABclonal Technology	1:1000
JAK1 Monoclonal antibody	66466-1-Ig	Proteintech	1:3000
Aldehyde oxidase Polyclonal antibody	19495-1-AP	Proteintech	1:300
GAPDH Polyclonal antibody	10494-1-AP	Proteintech	1:20,000
IFN-gamma R1 Polyclonal antibody	10808-1-AP	Proteintech	1:600

**Table 3:** Significant mRNA-level differences.

Gene	Comparison	*p*-Value	Significance	Confidence Interval
IFNG	NC vs. MD	0.0002	***	−9.91 to −3.50
	MD vs. XYN-1.8	0.0064	**	1.39 to 7.81
	MD vs. XYN-3.6	0.0006	***	2.78 to 9.19
	MD vs. BHS	0.0072	**	1.32 to 7.73
IFNGR1	NC vs. MD	0.013	*	−9.08 to −1.16
	MD vs. XYN-3.6	0.0429	*	0.14 to 8.06
	MD vs. BHS	0.0421	*	0.16 to 8.08
JAK1	NC vs. MD	0.0252	*	−2.99 to −0.21
	MD vs. XYN-3.6	0.0254	*	0.21 to 2.99
STAT3	NC vs. MD	<0.0001	****	−1.88 to −0.71
	MD vs. XYN-0.9	<0.0001	****	0.82 to 1.99
	MD vs. XYN-1.8	0.0017	**	0.40 to 1.57
	MD vs. XYN-3.6	<0.0001	****	0.79 to 1.96
	MD vs. BHS	0.0061	**	0.26 to 1.43
AOX	NC vs. MD	0.0168	*	−1.69 to −0.18
	MD vs. XYN-3.6	0.0104	*	0.26 to 1.76
	MD vs. BHS	0.0324	*	0.07 to 1.58

* *p* < 0.05, ** *p* < 0.01, *** *p* < 0.001, **** *p* < 0.0001.

**Table 4:** Significant protein-level differences.

Protein	Comparison	*p*-Value	Significance	Confidence Interval
IFNG	NC vs. MD	<0.0001	****	−0.7333 to −0.3485
	MD vs. 0.9	0.0002	***	0.2048 to 0.5896
	MD vs. 1.8	<0.0001	****	0.3922 to 0.7771
	MD vs. 3.6	<0.0001	****	0.4851 to 0.8699
	MD vs. BHS	0.0002	***	0.2115 to 0.5963
IFNGR1	NC vs. MD	0.0482	*	−0.5889 to −0.002520
	MD vs. 3.6	0.0161	*	0.07293 to 0.6593
JAK1	NC vs. MD	0.0431	*	−1.372 to −0.02326
	MD vs. 3.6	0.0468	*	0.01032 to 1.359
p-STAT3/STAT3	NC vs. MD	0.013	*	−1.751 to −0.2238
	MD vs. 3.6	0.0222	*	0.1385 to 1.665
AOX	NC vs. MD	<0.0001	****	−1.857 to −0.9269
	NC vs. 0.9	0.0009	***	−1.302 to −0.3711
	MD vs. 0.9	0.0208	*	0.09051 to 1.021
	MD vs. 1.8	<0.0001	****	0.6883 to 1.619
	MD vs. 3.6	<0.0001	****	0.8443 to 1.775
	MD vs. BHS	0.0002	***	0.5183 to 1.449

* *p* < 0.05, *** *p* < 0.001, **** *p* < 0.0001.

**Table 5:** Behavioral assessment criteria.

Score	0	1	2	3
General condition	Normal	Mild gait instability	Gait instability with weight loss	Severe gait instability, inability to move normally, weight loss
Auricular reflex	Strong	Marked	Mild	Absent
Nystagmus	Absent	Mild	Moderate-frequency, moderate-amplitude	High-frequency, large-amplitude
Righting reflex	Rapid	Impaired righting response	Difficulty righting	No righting response

## Data Availability

The data in this study were obtained from open public databases, and all relevant data are freely accessible on the respective websites.

## Supplementary Materials

The following supporting information can be downloaded at https://www.mdpi.com/article/10.3390/ph18091266/s1, Table S1: Component Identification Results of XYN in Positive Ion Mode; Table S2: Potential Related Phenotypes of MD; Table S3: Scoring Criteria of Behavioral Assessments; Figure S1: Body weight and behavioral scores of guinea pigs in each group after regrouping; Figure S2: GO Enrichment Analysis of Differentially Expressed Genes; Figure S3: KEGG Enrichment Analysis of Differentially Expressed Genes; Figure S4: GO Enrichment Analysis of Differentially Expressed Proteins; Figure S5: KEGG Enrichment Analysis of Differentially Expressed Proteins.

## Abbreviations

AQP	Aquaporin
AVP	Arginine vasopressin
BHS	Betahistine
CNN	Convolutional neural network
dDAVP	Diamino-Cys1, D-Arg8-vasopressin
DEGs	Differentially expressed genes
DEPs	Differentially expressed proteins
GWAS	Genome-wide association studies
IV	Instrumental variable
IVW	Inverse-variance weighted
LD	Linkage disequilibrium
MD	Ménière disease
MCODE	Molecular complex detection
MR	Mendelian randomization
OCSVM	One-class support vector machine
PBS	Phosphate-buffered saline
RT-qPCR	Real-time quantitative PCR
SNP	Single nucleotide polymorphism
TCM	Traditional Chinese medicine
WB	Western blot
