# Supplementary material for: Systems Network Integration of Transcriptomic, Proteomic, and Bioinformatic Analyses Reveals the Mechanism of XuanYunNing Tablets in Meniere’s Disease via JAK-STAT Pathway Modulation
Source: Pharmaceuticals (Basel). 2025 Aug 25;18(9):1266. doi: 10.3390/ph18091266 (PMC12472466; doi:10.3390/ph18091266)
Supplement: Supplementary file 1 [file pharmaceuticals-18-01266-s001.zip › Table S3.pdf]

**1. General Condition Assessment**

Under conditions free from external disturbances, the guinea pig's spontaneous locomotor activity was observed both within the cage and on a flat surface. Gait abnormalities indicative of vertigo—such as unsteady movement or circling behavior—were noted. The animals were then scored according to the following criteria:

- **Score 0 (Normal):**
  The guinea pig moves freely in the cage or on a flat surface with stable, alternating gait patterns. Locomotion is smooth and unobstructed, with no signs of abnormal behavior.
- **Score 1 (Mild Gait Instability):**
  Occasional, mild gait instability is observed during ambulation. The animal may display slightly uncoordinated steps and has difficulty maintaining a straight path, but remains active with near-normal locomotor activity.
- **Score 2 (Marked Gait Instability and Impaired Ambulation):**
  The animal exhibits obvious unsteadiness or difficulty walking, with reduced movement speed and occasional instability or mild backward stepping. It cannot maintain a straight trajectory, and overall activity is reduced. Locomotion appears clearly uncoordinated and is often accompanied by mild weight loss.
- **Score 3 (Severe Gait Disturbance and Immobility):**
  The guinea pig displays severe gait abnormalities, cannot maintain posture, and may exhibit falling, head tilting, or inability to move freely. Locomotor activity is markedly diminished or absent. These signs are typically accompanied by significant weight loss.

**2. Auricular Reflex Assessment**

Sound stimuli were generated using an online tone generator (https://www.onlinemictest.com/zh/tone-generator/) with a duration of 0.5 seconds and sine waveform. Three frequency ranges were used:

- **Low frequency**: 900–1300 Hz
- **Mid frequency**: ~2750 Hz
- **High frequency**: 12,000–13,000 Hz

While the guinea pig was at rest and in a quiet environment, sound was delivered at a fixed volume from a speaker positioned approximately 15 cm directly above the head. Each frequency stimulus was applied three times. The presence or absence of auricular reflexes—such as ear twitching, pinna movement, or orienting toward the sound—was observed and recorded. Scoring criteria were as follows:

- **Score 0 (Strong Auricular Reflex):**
  The guinea pig shows an immediate and pronounced ear movement toward the sound source, or a rapid pinning of the ears against the head. The reaction is brisk and robust, sometimes accompanied by whole-body responses such as jumping or avoidance. Reflexes are clear and highly visible.
- **Score 1 (Moderate Auricular Reflex):**
  The ear visibly turns toward the sound source or shows mild pinning behavior, which is easily observable but less intense. The movement amplitude is smaller, and the response may be slightly delayed.
- **Score 2 (Mild Auricular Reflex):**
  Only slight ear movement is observed in response to sound, and the reflex may be

incomplete or minimally noticeable.

- **Score 3 (Absent Auricular Reflex):**
  No ear movement or orienting behavior is detected in response to any frequency stimulus. The ears remain stationary with no apparent reaction.

**3. Rotating Platform Test (Nystagmus Assessment)**

Under quiet conditions, the guinea pig was gently placed on a rotating platform with the body and head lightly restrained to keep the head relatively fixed. **Spontaneous nystagmus** was first observed and recorded, including its **frequency and amplitude**.

Subsequently, the restrained animal was rotated **counterclockwise at a constant speed of 1.5 seconds per revolution for a total of 6 revolutions**. Immediately after cessation of rotation, **post-rotational (mechanically induced) nystagmus** was observed, and its frequency and amplitude were recorded. Scoring was based on the following criteria:

- **Score 0 (No Nystagmus):**
  No nystagmus is observed under any condition. Eye movement is either absent or extremely minimal and transient.
- **Score 1 (Mild Nystagmus):**
  Visible nystagmus is present, but with low frequency and small amplitude. Characterized by mild ocular oscillations that disappear within 2 seconds.
- **Score 2 (Moderate Nystagmus):**
  Nystagmus is more pronounced, with moderate frequency and amplitude. Eye movements are clearly visible and rhythmic, lasting approximately 3–5 seconds.
- **Score 3 (Severe Nystagmus):**
  Very intense and rapid nystagmus, with high frequency and large amplitude. Often accompanied by head tremors or oscillations, lasting longer than 5 seconds.

**4. Righting Reflex Test**

After calming the guinea pig, it was gently placed in a **supine position** (abdomen up, back down), with its head and back stabilized. The animal was then placed on a platform and released. The **latency to righting**, the **sensitivity of the response**, and **coordination of movement** during the righting process were observed and scored according to the following criteria:

- **Score 0 (Rapid and Coordinated Righting):**
  The guinea pig responds immediately upon release, flipping over quickly and smoothly to regain an upright posture. The movement is well-coordinated and completed within 1 second.
- **Score 1 (Mildly Impaired Righting):**
  Righting is slightly delayed. The animal may need to adjust its posture before completing the maneuver, but regains a standing position within 2 seconds.
- **Score 2 (Difficult Righting):**
  The guinea pig shows clear difficulty in righting itself, requiring multiple attempts. Movements are poorly coordinated and the process is noticeably hindered.
- **Score 3 (Absent Righting Reflex):**
  The animal is unable to right itself from the supine position and fails to resume a normal posture. No effective righting response is observed, and the animal may remain immobile or adopt an abnormal posture.
