# Supplementary material for: Systems Network Integration of Transcriptomic, Proteomic, and Bioinformatic Analyses Reveals the Mechanism of XuanYunNing Tablets in Meniere’s Disease via JAK-STAT Pathway Modulation
Source: Pharmaceuticals (Basel). 2025 Aug 25;18(9):1266. doi: 10.3390/ph18091266 (PMC12472466; doi:10.3390/ph18091266)
Supplement: Supplementary file 1 [file pharmaceuticals-18-01266-s001.zip › Renal H&E Staining/results.pdf]

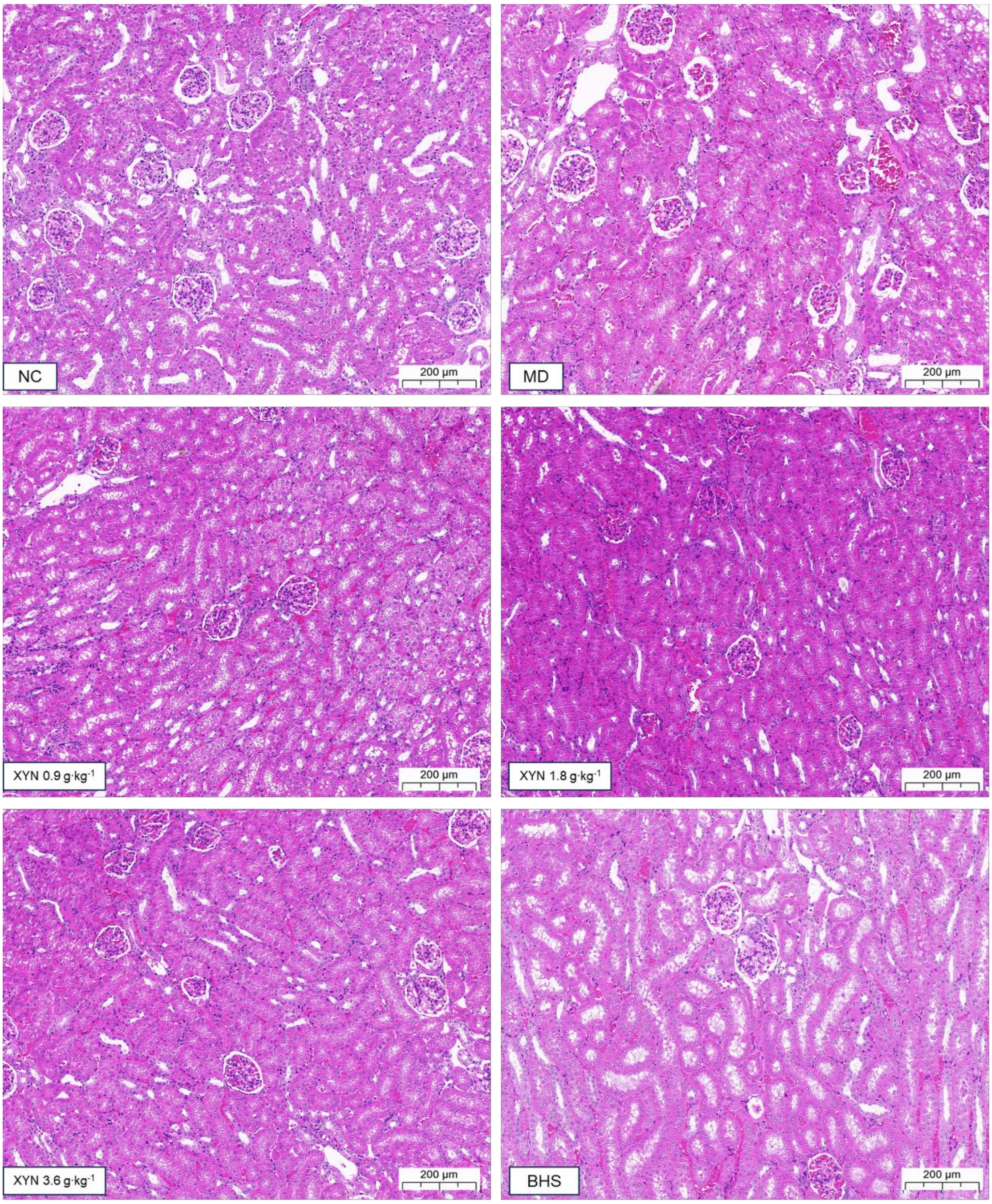

The HE staining results of the kidneys from guinea pigs in each group showed no significant abnormalities. This indicates that the modeling and drug administration procedures did not induce any pathological changes in the kidneys.

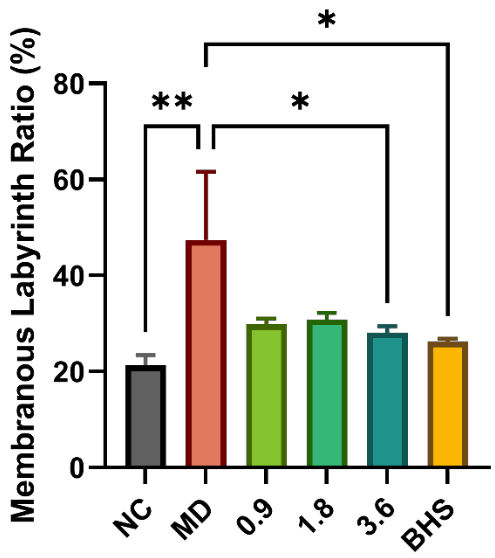

Bar chart of statistical results

	NC	MD	XYN-0.9	XYN-1.8	XYN-3.6	BHS
n1	23.80481	75.78752	32.17558	33.24507	27.54933	27.42466
n2	22.98514	35.05601	28.44473	30.96504	30.63554	25.86262
n3	17.07129	31.5277	28.82947	28.25265	26.21186	25.3558

statistical results
