# Supplementary material for: Systems Network Integration of Transcriptomic, Proteomic, and Bioinformatic Analyses Reveals the Mechanism of XuanYunNing Tablets in Meniere’s Disease via JAK-STAT Pathway Modulation
Source: Pharmaceuticals (Basel). 2025 Aug 25;18(9):1266. doi: 10.3390/ph18091266 (PMC12472466; doi:10.3390/ph18091266)
Supplement: Supplementary file 1 [file pharmaceuticals-18-01266-s001.zip › Basic Information of Xuan Yun Ning Pian and the Manufacturing Company.pdf]

[Drug Name]

Generic Name: XuanYunNing Tablets

Chinese Pinyin: Xuanyunning Pian

[Ingredients]

Active ingredients: *Alisma plantago-aquatica subsp. orientale* Sam., *Atractylodes macrocephala* Koidz., *Wolfiporia cocos (F.A. Wolf)* Ryvarden & Gilb., *Pinellia ternate (Thunb)* Breit., *Ligustrum lucidum* Ait., *Eclipta prostrata* L., *Chrysanthemum morifolium* Ramat., *Achyranthes bidentata* Blume., *Citrus reticulata* Blanco., and *Glycyrrhiza uralensis* Fisch.

Excipients: corn starch, silicon dioxide, microcrystalline cellulose, magnesium stearate, talcum powder, and film-coating premix.

[Description]

This product is a film-coated tablet. After removing the coating, the tablet appears brownish-yellow to brown. It has a slight odor and a mild taste.

[Functions and Indications]

Strengthens the spleen and eliminates dampness; nourishes the kidney and pacifies the liver.

Indicated for dizziness and vertigo caused by obstruction of phlegm-dampness and deficiency of liver and kidney.

[Specification]

Each tablet weighs 0.38 g, equivalent to 6 g of raw herbal materials.

[Dosage and Administration]

Oral administration. Take 2 to 3 tablets at a time, 3 to 4 times daily.

[Adverse Reactions]

Not yet clearly established.

[Contraindications]

Contraindicated in pregnant women and in individuals with exogenous febrile diseases.

[Precautions]

1. Avoid cold, greasy, and hard-to-digest foods.
2. Maintain a calm and optimistic mood during medication; avoid anger or emotional agitation.
3. Take the medicine after meals.
4. Patients with severe chronic diseases such as hypertension, heart disease, diabetes, liver or

kidney disorders should take this medicine under medical supervision.

5. If symptoms do not improve after 7 days of treatment, consult a physician.
6. Children and elderly or physically weak individuals should take this medicine under medical supervision.
7. Contraindicated in individuals allergic to this product. Use with caution in those with known allergic tendencies.
8. Do not use if the physical appearance of the product changes.
9. Keep out of reach of children.
10. Children should only use this product under adult supervision.
11. If you are currently taking other medications, consult a physician or pharmacist before using this product.
12. Once the composite foil pouch is opened, it is recommended to consume the tablets within one week.

[Drug Interactions]

Concurrent use with other drugs may cause interactions. Please consult a physician or pharmacist for details.

[Storage]

Keep sealed in a cool, dry place.

[Packaging]

Packed in polyvinyl chloride (PVC) hard film and pharmaceutical aluminum foil blister.

Each box contains 18, 24, or 36 tablets.

[Shelf Life]

36 months

[Standards of Execution]

National Drug Standard of the National Medical Products Administration: WS3-B-3482-98-1

[Approval Number]

National Medicine Standard Z45020605

[Date of Revision]

April 12, 2021

[Marketing Authorization Holder]

Guilin Sanjin Pharmaceutical Co., Ltd.

[Address of Marketing Authorization Holder]

No. 9 Renmin South Road, Lingui District, Guilin City, Guangxi, China

[Manufacturer]

Company Name: Guilin Sanjin Pharmaceutical Co., Ltd.

Manufacturing Address: No. 9 Renmin South Road, Lingui District, Guilin City, Guangxi, China

Postal Code: 541199

Telephone: 400-773-5858

Fax: +86-773-5812416

Website: www.sanjin.com.cn
