# Supplementary material for: Systems Network Integration of Transcriptomic, Proteomic, and Bioinformatic Analyses Reveals the Mechanism of XuanYunNing Tablets in Meniere’s Disease via JAK-STAT Pathway Modulation
Source: Pharmaceuticals (Basel). 2025 Aug 25;18(9):1266. doi: 10.3390/ph18091266 (PMC12472466; doi:10.3390/ph18091266)
Supplement: Supplementary file 1 [file pharmaceuticals-18-01266-s001.zip › Figure S1.pdf]

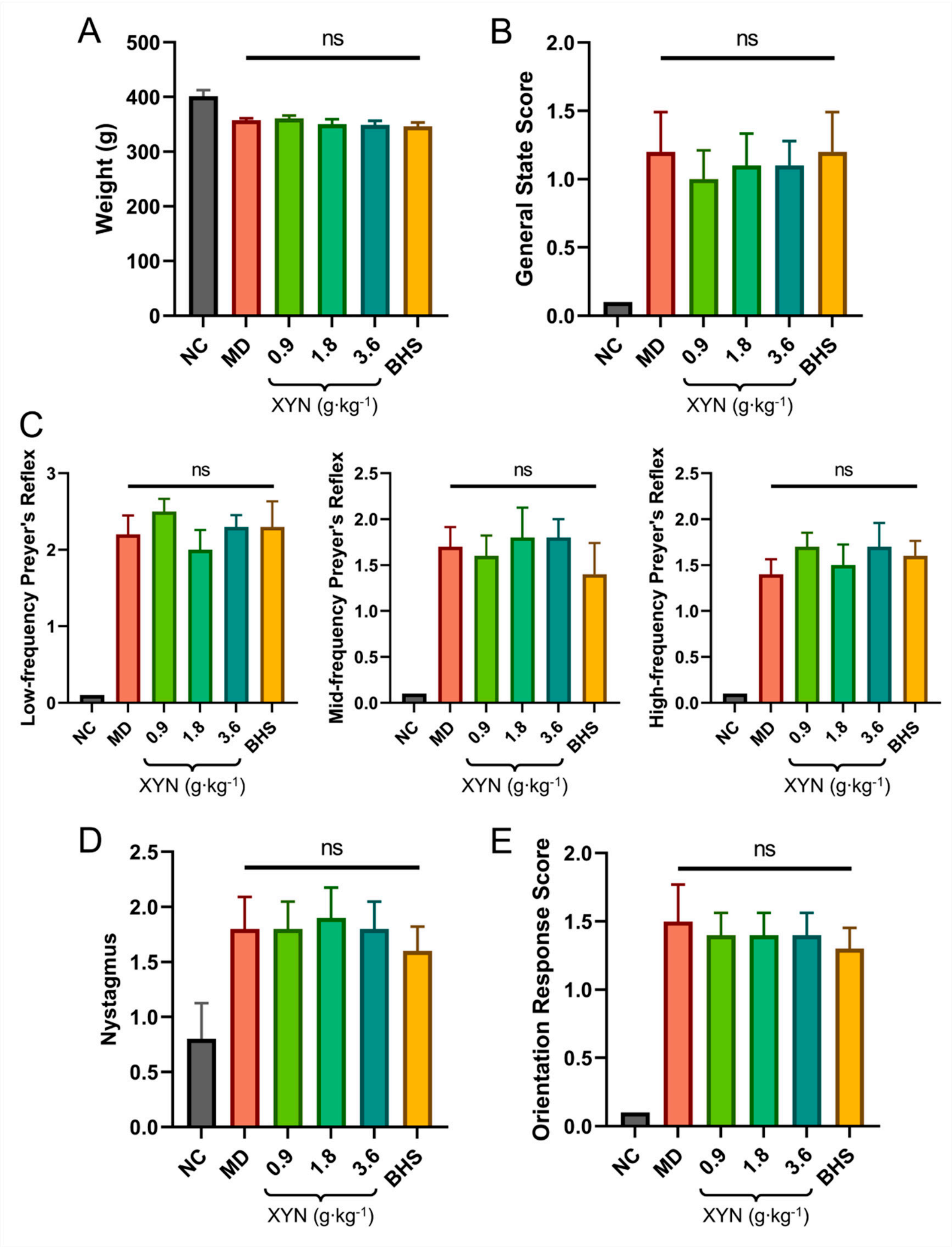

Figure S1. Body weight and behavioral scores of guinea pigs in each group after regrouping.

A: Weight; B: General condition observation;

C: Auricular Reflex at low, medium, and high frequencies;

D: Nystagmus Assessment; E: Righting Reflex Test.

Note: There were no significant differences in body weight or behavioral scores among the treatment groups.
