# Supplementary material for: Systems Network Integration of Transcriptomic, Proteomic, and Bioinformatic Analyses Reveals the Mechanism of XuanYunNing Tablets in Meniere’s Disease via JAK-STAT Pathway Modulation
Source: Pharmaceuticals (Basel). 2025 Aug 25;18(9):1266. doi: 10.3390/ph18091266 (PMC12472466; doi:10.3390/ph18091266)
Supplement: Supplementary file 1 [file pharmaceuticals-18-01266-s001.zip › Figures S2-S5.pdf]

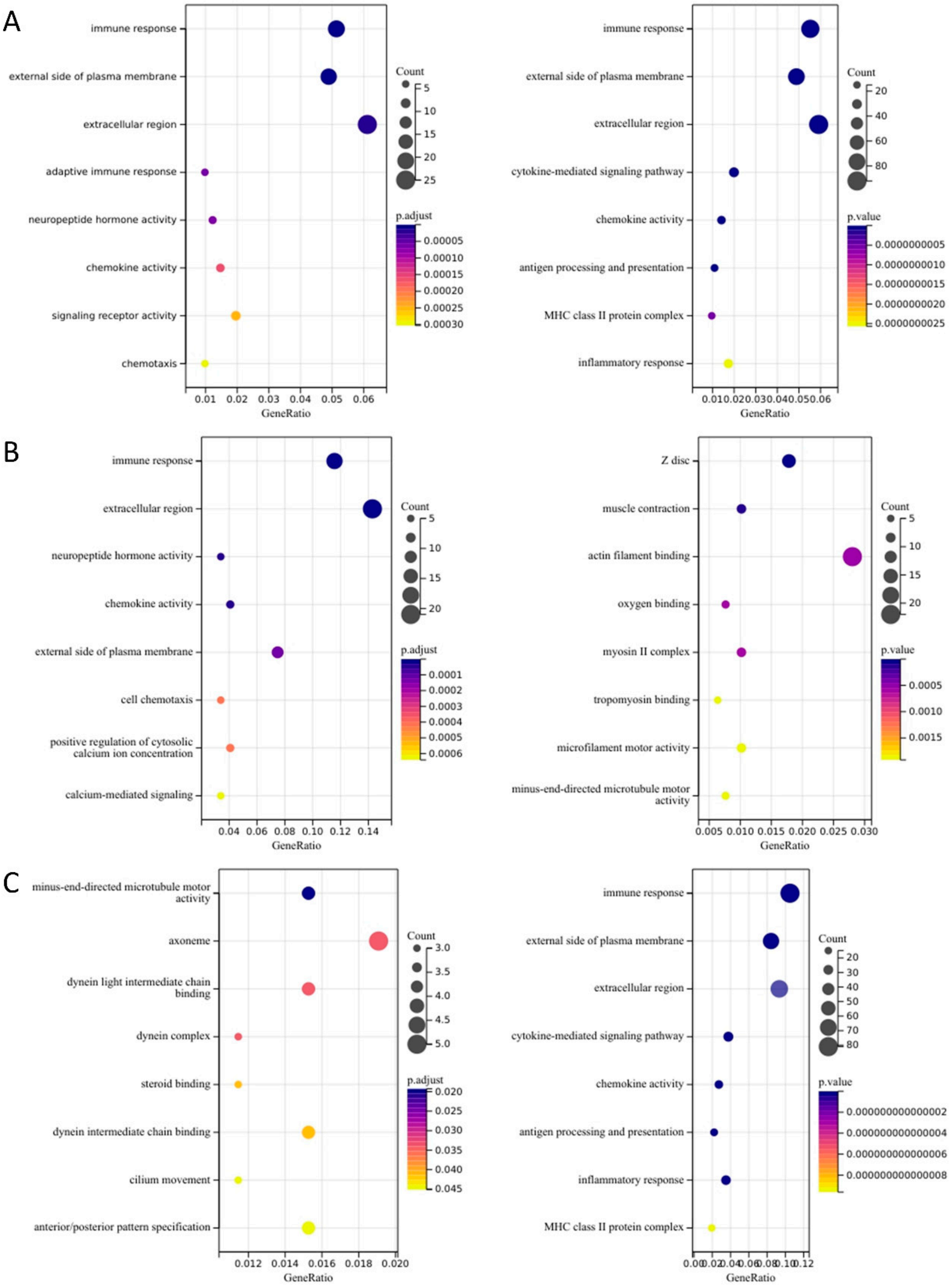

Figure S2. GO Enrichment Analysis of Differentially Expressed Genes

(A) GO enrichment results for DEGs in the modeling (left) and treatment (right) comparisons;

(B) GO enrichment of upregulated DEGs in both modeling and treatment comparisons;

(C) GO enrichment of downregulated DEGs in both modeling and treatment comparisons.

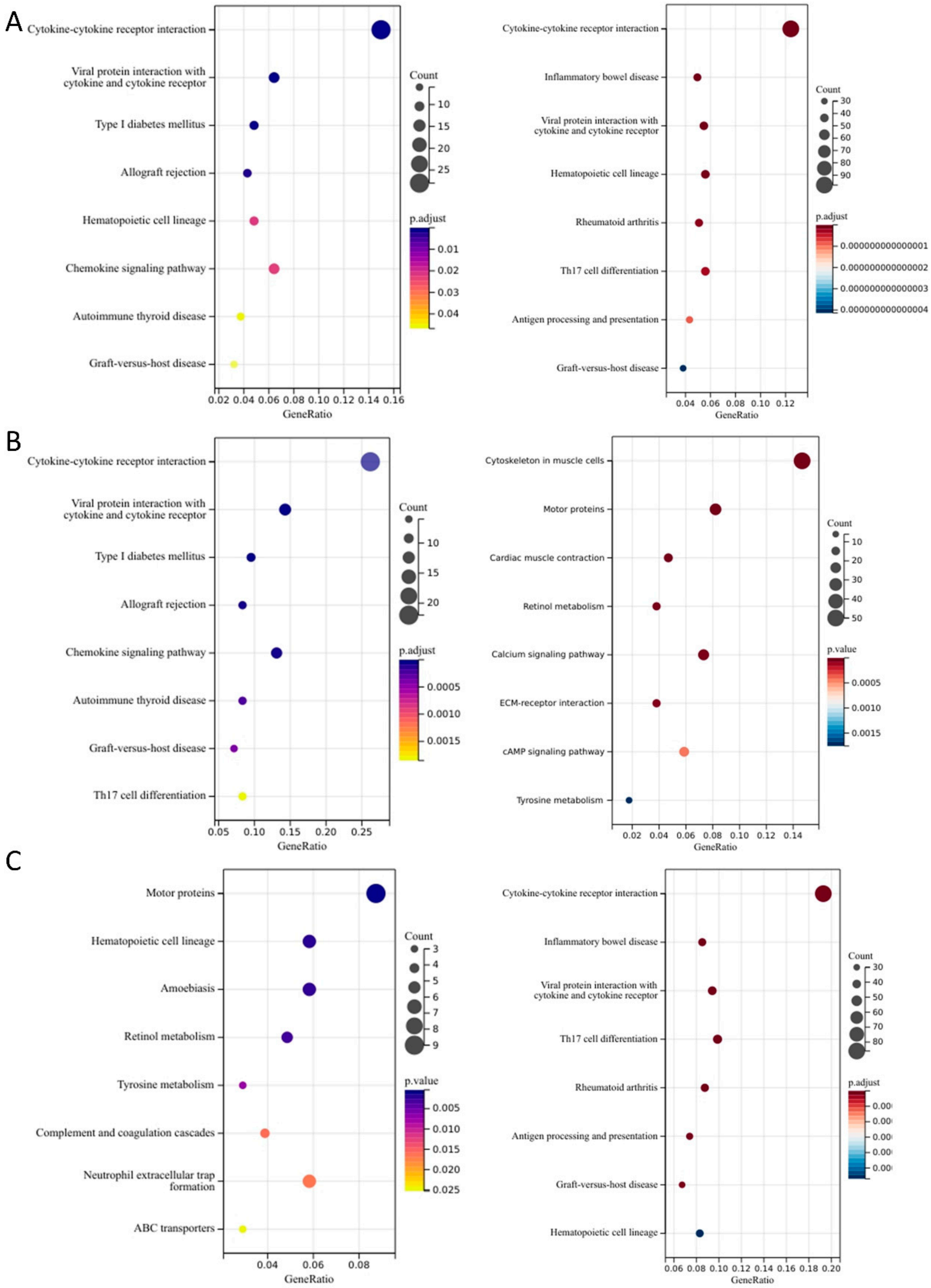

Figure S3. KEGG Enrichment Analysis of Differentially Expressed Genes

(A) KEGG enrichment results for DEGs in the modeling (left) and treatment (right) comparisons;

(B) KEGG enrichment of upregulated DEGs in both modeling and treatment comparisons;

(C) KEGG enrichment of downregulated DEGs in both modeling and treatment comparisons.

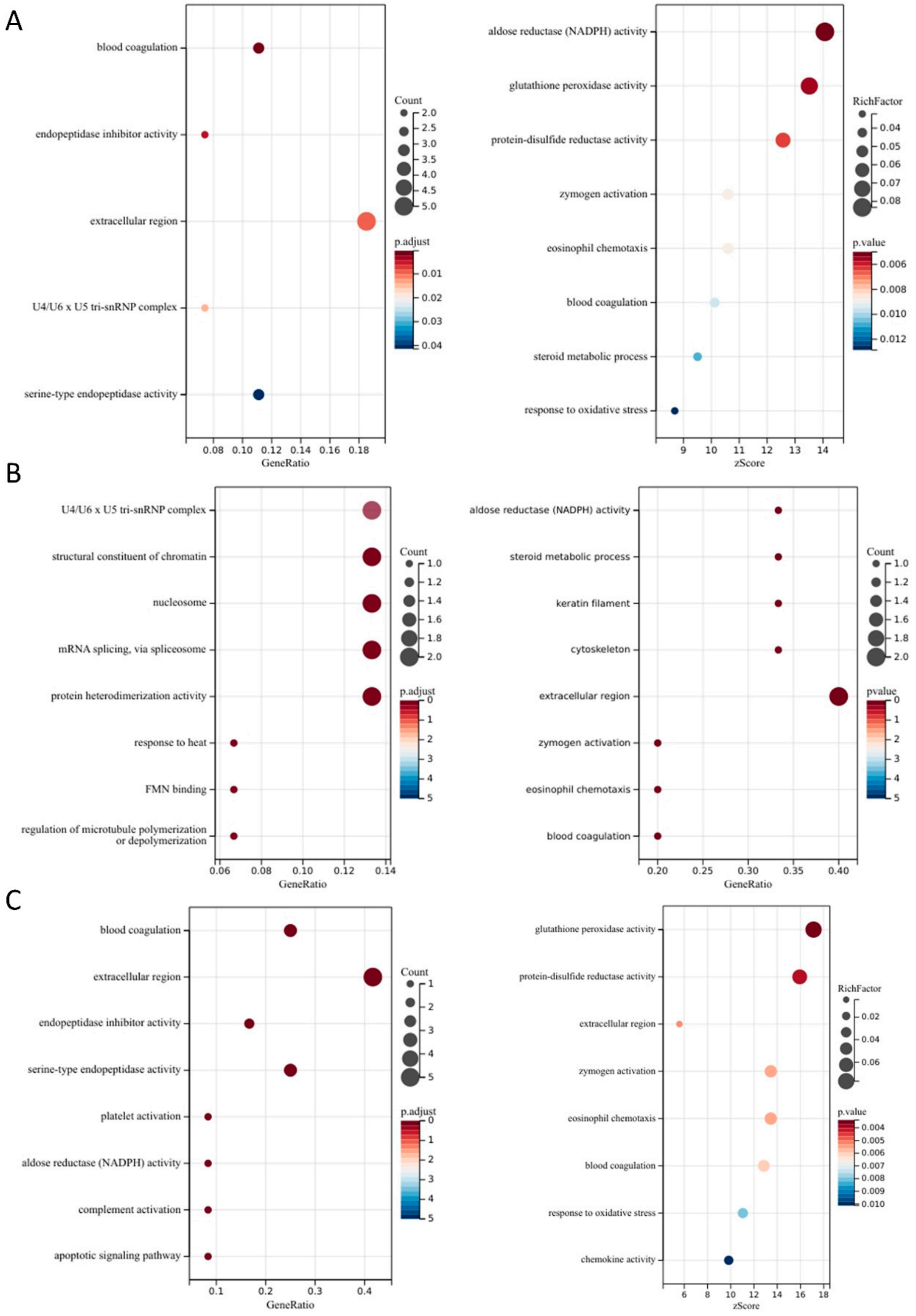

Figure S4. GO Enrichment Analysis of Differentially Expressed Proteins

(A) GO enrichment results for DEPs in the modeling (left) and treatment (right) comparisons;

(B) GO enrichment of upregulated DEPs in both modeling and treatment comparisons;

(C) GO enrichment of downregulated DEPs in both modeling and treatment comparisons.

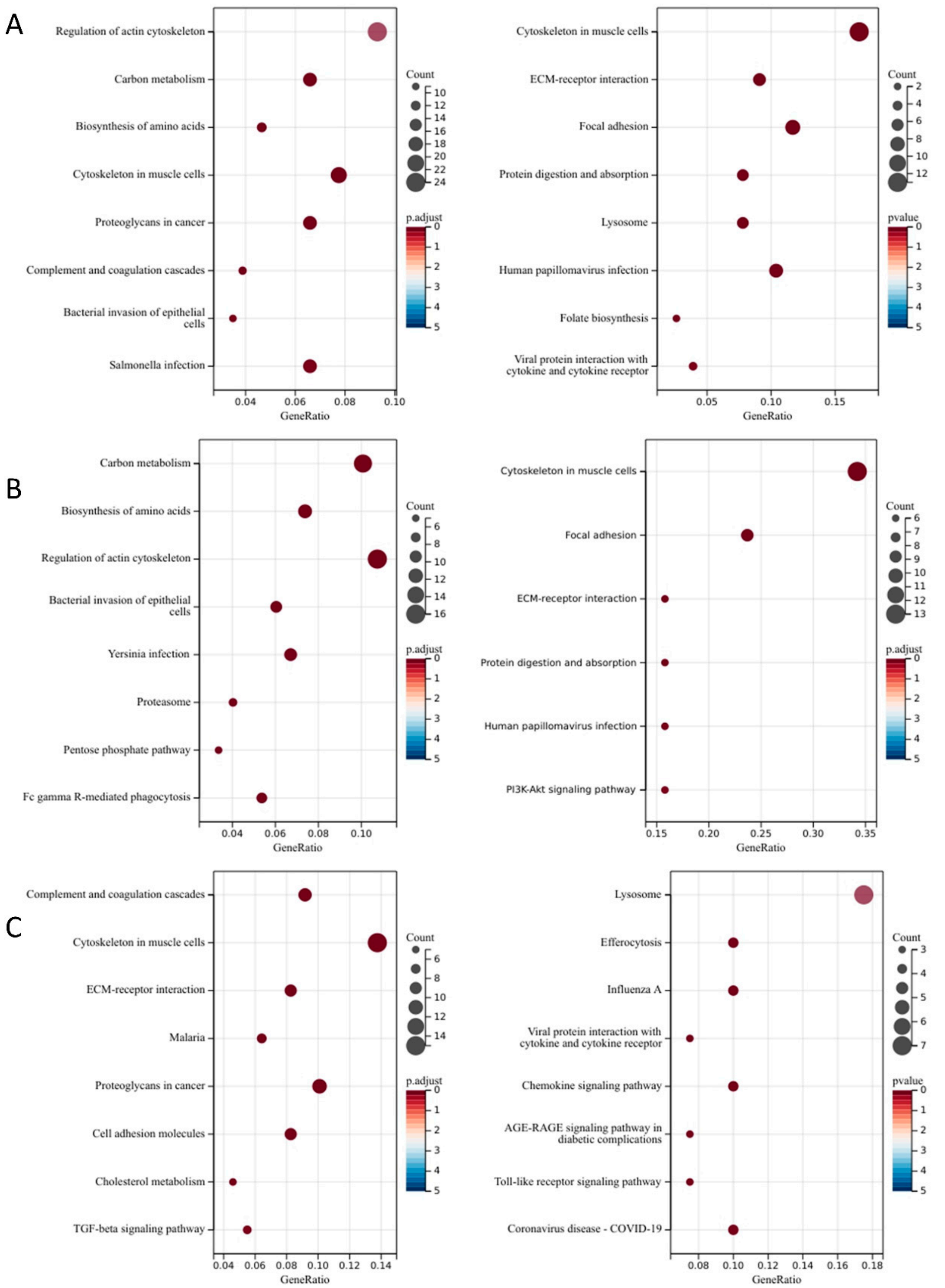

Figure S5. KEGG Enrichment Analysis of Differentially Expressed Proteins

(A) KEGG enrichment results for DEPs in the modeling (left) and treatment (right) comparisons;

(B) KEGG enrichment of upregulated DEPs in both modeling and treatment comparisons;

(C) KEGG enrichment of downregulated DEPs in both modeling and treatment comparisons.
