# Supplementary material for: Systems Network Integration of Transcriptomic, Proteomic, and Bioinformatic Analyses Reveals the Mechanism of XuanYunNing Tablets in Meniere’s Disease via JAK-STAT Pathway Modulation
Source: Pharmaceuticals (Basel). 2025 Aug 25;18(9):1266. doi: 10.3390/ph18091266 (PMC12472466; doi:10.3390/ph18091266)
Supplement: Supplementary file 1 [file pharmaceuticals-18-01266-s001.zip › Table S1.pdf]

Table S1. Component Identification Results of XYN in Positive Ion Mode

NO.	Molecular formula	t_R/min	*m/z*		ppm	MS/MS fragments	Structural type	Name	Peak area
			Theoretical value	Actual value					
1	C5H11N3O2	1.106	145.0851	145.0853	1.08	60.0566,86.0608,87.0449,104.0713,145.1058,146.0928,147.0971,205.5024	Alkaloids	4-[(Aminoiminomethyl)amino]butanoicacid	81378991
2	C21H16O6	1.112	364.0947	364.0988	11.22		Flavonoids	GancaoninF	4.32E+08
3	C5H13NO	1.157	103.0997	103.1003	5.74	118.0867,144.1021,156.0424,381.0799,543.1331,725.2064,1110.9283,1291.7289	Alkaloids	Choline	1.24E+10
4	C9H13NO2	1.169	167.0946	167.0948	1.24		Alkaloids	Synephrine	73059256
5	C5H11NO2	1.179	117.079	117.0794	3.67	113.0229,193.0343,351.0563,414.2754,595.7026,799.8326,837.3918	Alkaloids	Betaine	9.22E+09
6	C7H13NO2	1.18	143.0946	143.0949	1.66		Alkaloids	Stachydrine	3.35E+09
7	C10H13N5O4	1.183	267.0968	267.0972	1.73		Alkaloids	Adenosine	36379584
8	C6H11NO2	1.184	129.079	129.0793	2.27	70.0660,84.0816,85.0849,129.1109,130.0867,131.0901	Alkaloids	Achyranthine	3.92E+08
9	C7H7NO2	1.193	137.0477	137.0479	1.5		Alkaloids	Trigonelline	4.33E+08
10	C5H5N5	1.197	135.0545	135.0548	1.89	70.6717,94.0406,119.0358,135.0919,136.0622,137.1075,154.0507	Alkaloids	Adenine	36369961
11	C11H8N2	1.296	168.0688	168.0691	1.87		Alkaloids	Norharman	1.93E+08
12	C8H9NO2	1.364	151.0633	151.0637	2.1	60.0453,68.9831,81.0343,96.0450,106.0657,120.0449,134.0604,151.0965,152.0710,153.0750	Phenylpropanoids	Methylanthranilate	9.29E+08
13	C13H15NO3	1.39	233.1052	233.1055	1.26		Phenylpropanoids	4-(3-Methyl-2-butenoxy)isonitrosoaceto	1.55E+08

								phenone	
14	C8H11NO	1.391	137.0841	137.0843	1.55		Alkaloids	Tyramine	1.19E+08
15	C6H6N2O	1.393	122.048	122.0484	2.94	80.0503,96.0450,123.0558,124.0398,140.0344,170.6901,187.0831,256.6668	Alkaloids	Nicotinamide	1.27E+08
16	C9H11NO2	1.396	165.079	165.0793	2.14	57.0710,70.0660,81.0344,96.0451,106.0657,120.0812,134.0604,148.0760,166.0864,167.0907	Phenylpropanoids	Methyl2-(methylamino)benzoate	3.06E+08
17	C7H15NO4S	1.459	209.0722	209.0692	-14.46	81.0343,140.0345,178.0502,210.0766,211.0801,322.0334	Alkaloids	3-(N-Morpholino)propanesulfonicacid	64024731
18	C21H22O8	1.459	402.1315	402.1322	1.79		Flavonoids	Nobiletin	9.63E+08
19	C22H24O9	1.545	432.142	432.1429	2.01	69.7489,165.0552,271.0604,403.1031,433.1511	Flavonoids	Medicarpin-3-O-glucoside	7.44E+08
20	C12H18O3	18.055	210.1256	210.126	1.9	71.0501,95.0861,111.0447,123.0809,151.1123,153.0914,165.1276,178.0493,193.1221,210.0770,211.1333,212.1375	Terpenoids	(-)-Jasmonicacid	48643416
21	C13H20O3	21.921	224.1412	224.1416	1.6		Terpenoids	Methyljasmonate	8837637
22	C30H46O3	28.773	454.3447	454.3459	2.66	89.0606,121.1017,147.1171,219.1747,339.2683,383.2957,419.3325,455.3529,456.3565	Terpenoids	New poricoic acid	20817394
23	C30H44O4	29.57	468.324	468.3252	2.67	59.0501,107.0863,145.1016,175.1123,201.1279,215.1441,281.1910,295.2048,353.2484,381.2799,451.3218,469.3325	Terpenoids	Glycyrol	1.23E+09
24	C15H20O3	33.19	248.1412	248.1416	1.32	71.0865,107.9670,116.0535,133.0651,161.0601,179.0707,180.0737,207.1240,249.1878	Terpenoids	Atractylenolide III	15162406
25	C16H22O4	33.439	278.1518	278.1522	1.54	57.0708,97.6573,121.0286,149.0237,150.0271,167.0336,205.0866,279.0942	Phenylpropanoids	5,5'-Dibutoxy-2,2'-bifuran	2.6E+08

Table S1. Component Identification Results of XYN in Negative Ion Mode

NO.	Molecular formula	t_R/min	m/z		ppm	MS/MS fragments	Structural type	Name	Peak area
			Theoretical value	Actual value					
1	C12H8O4	1.142	216.0423	216.0398	-11.48	89.0230,179.0553,215.0331	Coumarins	5-Methoxypsoralen	6.48E+08
2	C6H12O7	1.153	196.0583	196.0577	-3.27	75.0072,195.0501,345.9791	Organic acids	Galactonic acid	9.63E+08
3	C5H10O6	1.154	166.0477	166.0468	-5.85		Organic acids	Ribonic acid	3.27E+08
4	C6H14O6	1.17	182.079	182.0783	-4.19		Others	D-Mannitol	9.66E+08
5	C7H12O6	1.182	192.0634	192.0627	-3.84	111.0073,191.0551,192.0585,397.2585	Organic acids	Quinic acid	9.76E+08
6	C4H6O5	1.218	134.0215	134.0204	-8.73	115.0023,133.0129,262.4294	Organic acids	Malic acid	1.38E+09
7	C6H8O6	1.224	176.0321	176.0313	-4.4	87.0073,175.0238,176.0262,261.2556,351.2978	Organic acids	(+)-Ascorbic acid	61730982
8	C5H8O4	1.246	132.0423	132.0414	-6.91		Organic acids	Glutaric acid	4E+08
9	C12H10O4	1.309	218.0579	218.0554	-11.56		Flavonoids	Kiyomal	2.82E+08
10	C18H16O8	1.318	360.0845	360.0828	-4.72		Flavonoids	Jaceidin	2.65E+08
11	C5H10O5	1.321	150.0528	150.0518	-7.11	59.0123,149.0443,150.0458,245.9536	Organic acids	beta-L-Arabinopyranose	2.47E+08
12	C9H12N2O6	1.65	244.0695	244.0703	3.03	99.9243,173.9614,243.9675	Alkaloids	Uridine	90017871
13	C5H8O5	1.796	148.0372	148.0361	-7.04		Organic acids	Citramalic acid	5.87E+08
14	C4H6O4	1.801	118.0266	118.0254	-10.4	116.9271,117.9272,237.8365	Organic acids	Succinic acid	6.31E+08
15	C6H6O3	2.813	126.0317	126.0305	-9.53		Organic acids	1,3,5-Trihydroxybenzene	1.61E+08
16	C7H6O4	3.88	154.0266	154.0256	-6.43		Organic acids	Gentisic acid	6.95E+08
17	C7H6O4	4.267	154.0266	154.0256	-6.43	109.0280,153.0181,154.0217	Organic acids	Gentisic acid	8.97E+08
18	C6H6O2	4.267	110.0368	110.0355	-11.65	109.028	Phenylpropanoids	Catechol	1.3E+08
19	C16H24O10	5.391	376.137	376.1374	1.3	59.0123,201.0161,331.1396,375.131	Terpenoids	7-Epiloganic acid	1.57E+08

						3,440.9364			
20	C16H18O9	5.492	354.0951	354.0955	1.28		Phenylpropanoids	Neochlorogenic acid	6.97E+08
21	C7H6O3	6.562	138.0317	138.0306	-8.09		Organic acids	Salicylic acid	2.41E+08
22	C6H6O2	6.598	110.0368	110.0355	-11.65		Phenylpropanoids	Catechol	71752285
23	C9H11NO2	6.795	165.079	165.0781	-5.46	106.0647,165.0546207.0385,306.1401	Phenylpropanoids	Methyl 2-(methylamino)benzoate	89663274
24	C8H9NO2	7.421	151.0633	151.0625	-5.74		Phenylpropanoids	Methyl anthranilate	46158925
25	C16H18O9	7.6	354.0951	354.0955	1.28	85.0279,179.0339,191.0551,192.0585,352.8752,353.0876,358.5428	Phenylpropanoids	Neochlorogenic acid	1.13E+09
26	C8H8O3	7.91	152.0473	152.0464	-6.41		Phenylpropanoids	Vanillin	1.45E+08
27	C16H18O9	8.097	354.0951	354.0955	1.28		Phenylpropanoids	Neochlorogenic acid	1.02E+09
28	C8H8O3	8.103	152.0473	152.0464	-6.41	109.0281,151.0389,152.0426	Phenylpropanoids	Vanillin	1.49E+08
29	C16H22O11	8.107	390.1162	390.1169	1.68	59.0124,121.0644,183.0651,345.1191,359.1089	Terpenoids	Oleoside	1.43E+08
30	C9H6O4	8.109	178.0266	178.0258	-4.54		Coumarins	Aesculetin	62526495
31	C7H6O2	8.734	122.0368	122.0356	-9.81	121.0281	Organic acids	Benzoic acid	1.82E+08
32	C27H30O15	9.306	594.1585	594.1596	1.83		Flavonoids	Luteolin-7-O-rutinoside	2.33E+08
33	C25H24O12	9.61	516.1268	516.1275	1.43		Phenylpropanoids	Isochlorogenic acid A	3.58E+08
34	C8H8O3	9.65	152.0473	152.0464	-6.41		Phenylpropanoids	Vanillin	74491673
35	C8H9NO2	10.342	151.0633	151.0624	-6.3	109.0154,150.0547	Phenylpropanoids	Methyl anthranilate	1.46E+08
36	C21H20O13	10.358	480.0904	480.0913	1.87	61.9857,165.9895,271.0240,316.0221,317.0301,318.0334,433.2435,479.0824	Flavonoids	Quercetagetin 7-glucoside	1.73E+08
37	C9H8O3	10.422	164.0473	164.0465	-5.47	119.0488,163.0388	Phenylpropanoids	p-Coumaric acid	65440184
38	C21H20O12	10.603	464.0955	464.0963	1.83		Flavonoids	Quercetin-3-O-	96831224

								glucoside	
39	C9H6O3	11.031	162.0317	162.0308	-5.76	73.0279,161.0232,162.0284,243.4122	Coumarins	Umbelliferone	91325156
40	C27H30O14	11.241	578.1636	578.1643	1.22	68.7114,137.0225,191.0347,241.7986,297.0763,383.0771,457.1140,576.1368,577.1559,578.1584	Flavonoids	Apigenin-7-O-rutinoside	1.47E+08
41	C10H10O4	11.315	194.0579	194.0573	-3.32	68.8475,149.0232,193.0497,194.0531	Phenylpropanoids	Ferulic acid	2.29E+09
42	C27H30O15	11.54	594.1585	594.1596	1.83		Flavonoids	Luteolin-7-O-rutinoside	96182922
43	C8H16O4	11.626	176.1049	176.104	-5.04	72.9916,75.0072,175.0965,176.0999,249.8980,303.6488	Others	1,4,7,10-Tetraoxacyclododecane	1.35E+08
44	C21H22O9	11.702	418.1264	418.1271	1.62	68.6830,91.0162,119.0488,135.0074,153.0181,194.9425,255.0660,256.0695,268.3706,416.1349,417.1193,418.1218a	Terpenoids	Glycyrrhizin	1.11E+09
45	C21H20O11	11.897	448.1006	448.1011	1.17		Flavonoids	Luteoloside	6.03E+09
46	C22H22O13	11.898	494.106	494.1066	1.22	69.1344,284.0315,285.0406,303.9464,447.0928,448.0938,493.2317	Flavonoids	Patuletin 7-glucoside	4.68E+08
47	C21H18O12	11.943	462.0798	462.0804	1.3	113.0211,175.0216,285.0403,286.0436,327.0509,461.0722,472.3338,740.1696	Flavonoids	Demethylwedelolactone 3-O-glucoside	5.07E+08
48	C21H20O12	11.999	464.0955	464.0963	1.83	85.0279,151.0024,175.0237,287.0559,288.0594,463.0876,464.0909	Flavonoids	Quercetin-3-O-glucoside	5.95E+08
49	C29H36O15	12.092	624.2054	624.2064	1.62		Phenylpropanoids	Acteoside	1.41E+08
50	C10H12O4	12.103	196.0736	196.073	-2.95		Phenylpropanoids	Xanthoxylin	1.27E+08
51	C9H6O4	12.239	178.0266	178.0258	-4.71		Coumarins	Aesculetin	53470632

52	C25H24O12	12.578	516.1268	516.1272	0.84		Phenylpropanoids	Isochlorogenic acid A	1.72E+09
53	C22H22O11	12.631	462.1162	462.1168	1.21		Flavonoids	Biochanin A-7-O-β-D-glucopyranoside	87221307
54	C27H32O14	12.694	580.1792	580.1803	1.9	151.0024,177.0181,271.0611,272.0646,295.0615,459.1151,579.1719,580.1751	Flavonoids	Naringin	3.29E+09
55	C27H30O14	12.695	578.1636	578.1643	1.22		Flavonoids	Apigenin-7-O-rutinoside	30240737
56	C29H36O15	12.696	624.2054	624.2064	1.62	161.0231,179.0338,461.1664,577.1570,623.1983,624.2015	Phenylpropanoids	Acteoside	6.28E+08
57	C31H42O17	12.803	686.2422	686.2436	1.97	101.0229,121.0280,223.0607,299.1135,453.1403,523.1812,524.1867,596.1743,685.2351	Phenylpropanoids	Oleonuezhenide	1.47E+10
58	C15H10O7	12.817	302.0427	302.0427	0.28		Flavonoids	Herbacetin	88949546
59	C15H8O7	12.91	300.027	300.0274	1.39	68.7315,161.0429,271.0246,299.0195	Flavonoids	Desmethylwedelolactone	1.34E+08
60	C8H8O3	12.926	152.0473	152.0464	-6.41		Phenylpropanoids	Vanillin	58866149
61	C21H20O10	13.212	432.1057	432.1059	0.54	65.0012,151.0024,240.0418,268.0375,270.0486,311.0560,431.0982	Flavonoids	Apigenin-7-O-glucoside	1.17E+10
62	C21H22O10	13.335	434.1213	434.1221	1.74	61.9868,151.0024,271.0611,432.1009,433.1124,602.9226,729.8777,781.3422	Flavonoids	Naringenin 7-O-beta-D-glucoside	4.41E+08
63	C21H18O11	13.345	446.0849	446.0855	1.37		Flavonoids	Apigenin-7-O-glucuronide	4.37E+08
64	C28H34O15	13.503	610.1898	610.191	2.01	69.0039,125.0205,164.0077,286.0481,301.0714,325.0710	Flavonoids	Hesperidin	6.49E+09
65	C25H24O12	13.509	516.1268	516.1272	0.84	93.0330,173.0443,255.0654,353.0874,447.0941,515.1190,516.1223,962.	Phenylpropanoids	Isochlorogenic acid A	2.03E+09

						2842			
66	C7H6O3	13.64	138.0317	138.0305	-8.43	93.0331,137.0231	Organic acids	Salicylic acid	3.55E+08
67	C29H32O17	13.657	652.164	652.1654	2.16	57.0331,165.9896,258.0168,287.0194,329.0301,345.0611,507.1142,589.1541,651.1567.652.1595	Flavonoids	3-Hydroxy-3-methyl-pentanedioic acid, 2'-ester with 3-(beta-D-glucopyranosyloxy)-5,7-dihydroxy-2-(4-hydroxy-3-methoxyphenyl)-8-methoxy-4H-1-benzopyran-4-one	3.65E+08
68	C8H14O	13.662	126.1045	126.1032	-9.72		Terpenoids	6-Methyl-5-hepten-2-one	14471358
69	C22H22O11	13.807	462.1162	462.117	1.77		Flavonoids	Biochanin A-7-O-β-D-glucopyranoside	51641189
70	C15H10O9S	13.947	366.0046	366.0049	0.94	70.5316,151.0022,199.0382,285.0403,364.9969	Flavonoids	Luteolin sulfate	9.78E+08
71	C23H23O12	14.083	491.119	491.1153	-7.54	151.0023,284.0323,285.0385,287.0559,443.1919,490,1069	Flavonoids	Cyanidin 3-(4"-acetylglucoside)	21031255
72	C25H32O13	14.565	540.1843	540.1852	1.67	95.0487,223.0606,275.0948,377.1240,539.1774,540.1810,662.4823	Phenylpropanoids	Oleuropein	2.12E+08
73	C15H10O4	14.576	254.0579	254.058	0.29	117.0331,135.0074,235.0591,235.0503	Quinones	Chrysophanol	67121307
74	C21H22O9	14.676	418.1264	418.1269	1.35		Terpenoids	Glycyrrhizin	9.87E+08
75	C21H22O9	15.048	418.1264	418.1269	1.34		Terpenoids	Glycyrrhizin	2.35E+08
76	C16H14O5	15.208	286.0841	286.0846	1.48	133.0280,199.0391,285.0403,286.0437	Flavonoids	Isosakuranetin	1.38E+08
77	C15H10O4	15.225	254.0579	254.0581	0.71	139.0751,151.0751,219.1384,263.13	Quinones	Chrysophanol	23095004

						06			
78	C25H32O13	15.25	540.1843	540.1855	2.24		Phenylpropanoids	Oleuropein	25185868
79	C32H42O13	15.344	634.2625	634.2639	2.19	71.0123,161.0446,269.0475,331.1915,427.2124,529.5552,587.1779,633.2564,634.2617	Terpenoids	Obacunone 17-glucoside	1.78E+08
80	C15H12O4	15.568	256.0736	256.0736	0.31		Flavonoids	Glycyrrhetinic acid	4.99E+08
81	C22H20O11	15.684	460.1006	460.1015	1.94	61.9857,151.0025,240.0423,268.0376,270.0489,311.0564,431.0976,459.0933,460.0966	Flavonoids	Wogonoside	2.95E+08
82	C15H20O4	15.734	264.1362	264.1365	1.4		Terpenoids	Tatridin A	1.55E+08
83	C48H64O27	15.824	1072.364	1072.366	2.43	101.0229,223.0605,353.0905,453.1399,523.1821,685.2349,771.2356,909.3038,1071.3566	Phenylpropanoids	Oleonuezhenide	1.66E+09
84	C15H12O6	15.845	288.0634	288.0639	1.65		Flavonoids	Eriodictyol	3.38E+08
85	C15H10O6	16.007	286.0477	286.048	0.87	83.0119,133.0279,199.0391,285.0403,286.0437	Flavonoids	Luteolin	5.72E+09
86	C25H32O12	16.061	524.1894	524.1901	1.29	101.0228,119.0336,299.1132,453.1399,523.1817,524.1850,631.0701,820.0648,966.6251	Phenylpropanoids	Ligstroside	78774189
87	C15H8O7	16.065	300.027	300.0272	0.67		Flavonoids	Desmethylwedelolactone	24922360
88	C15H10O7	16.075	302.0427	302.0429	0.79	69.3938,133.0279,255.0296,300.0251,301.0355,302.0387	Flavonoids	Herbacetin	1.31E+08
89	C16H12O5	16.187	284.0685	284.0687	0.95	57.0331,132.0200,268.0375,283.0610,284.0642	Flavonoids	Daphnetin	48853515
90	C28H34O14	16.4	594.1949	594.196	1.97	107.0104,164.0102,270.0531,285.0766,286.0800,309.0767,431.1123,592.1802,593.1876	Flavonoids	Didymin	7.59E+08

91	C16H10O7	16.525	314.0427	314.0431	1.54	69.4163,186.0296,269.0070,298.0117,313.0354	Flavonoids	Wedelolactone	1.23E+09
92	C10H12O4	17.483	196.0736	196.073	-2.79		Phenylpropanoids	Xanthoxylin	38021037
93	C22H22O11	17.601	462.1162	462.1174	2.47	89.0214,161.0427,283.0245,446.0851,461.1088,593.6417,730.5212,931.3649	Flavonoids	Biochanin A-7-O-β-D-glucopyranoside	1.14E+08
94	C15H10O5	17.871	270.0528	270.053	0.74	65.0015,151.0024,225.0551,269.0454	Flavonoids	Apigenin	6.13E+09
95	C15H12O5	17.919	272.0685	272.0686	0.53	119.0488,151.0025,177.0183,270.0488,271.0613	Flavonoids	Naringenin	3.54E+08
96	C33H40O18	18.199	724.2215	724.2233	2.59	57.0331,99.0436,125.0230,255.0679,329.0301,417.1191,418.1227,661.2166,722.2108,723.2144,724.2478	Flavonoids	Melitidin	5.01E+08
97	C16H12O6	18.331	300.0634	300.0638	1.36		Flavonoids	Eugenin	1.75E+08
98	C44H64O18	18.509	880.4093	880.4118	2.83		Terpenoids	Uralsaponin M	1.78E+08
99	C16H12O5	18.544	284.0685	284.0687	0.94		Flavonoids	Daphnetin	36445762
100	C42H68O17S	19.168	876.4177	876.4197	2.25	241.0016,449.1850,651.3561,713.3575,875.4108,876.4136	Terpenoids	Ecliptasaponin VI	98326343
101	C15H12O4	19.832	256.0736	256.0736	0.25	119.0489,135.0074,213.0550,255.0661,256.0694,340.2208,434.5719	Flavonoids	Glycyrrhetinic acid	9.96E+08
102	C48H76O19	20.164	956.4981	956.5006	2.6	321.0831,497.1142,795.4437,925.4799,955.4531,956.4654,985.4649	Terpenoids	Ginsenoside Ro	17248018
103	C42H62O17	20.227	838.3987	838.401	2.69	113.0229,193.0343,351.0563,414.2754,595.7026,799.8326,837.3918	Terpenoids	Licoricesaponin G2	4.65E+08
104	C32H42O13	20.331	634.2625	634.2642	2.57		Terpenoids	Obacunone 17-glucoside	8323160
105	C42H60O16	20.34	820.3881	820.3902	2.53	113.0230,193.0345,289.0596,351.0566,643.3513,694.4516,769.7073,819	Terpenoids	licoricesaponin E2	1.05E+08

						.3813			
106	C16H12O4	20.389	268.0736	268.0739	1.15	68.7340,223.0395,252.0424,267.0661,268.0693,300.1977	Flavonoids	7-Methoxy-4'-hydroxyflavone	4.91E+08
107	C47H74O18	20.543	926.4875	926.4897	2.36	71.0123,139.0021,157.0132,455.3542,551.3737,701.4261,763.4271,812.3760,925.4807	Terpenoids	Chikusetsusaponin IV	71308144
108	C47H74O18	21.043	926.4875	926.4897	2.36		Terpenoids	Chikusetsusaponin IV	32167633
109	C42H62O16	21.338	822.4038	822.4061	2.86	113.0231,193.0348,287.5161,351.0564,586.0700,694.3326,745.4648,821.3973	Terpenoids	Licorice secondary saponin D	4.45E+09
110	C42H62O16	22.639	822.4038	822.4061	2.86		Terpenoids	Licorice secondary saponin D	9.32E+08
111	C16H12O5	22.933	284.0685	284.0687	0.94		Flavonoids	Daphnetin	30907040
112	C16H14O5	23.156	286.0841	286.0845	1.37		Flavonoids	Isosakuranetin	99612317
113	C36H62O11S	23.368	702.4013	702.3969	-6.32	71.0123,161.0444,362.2239,587.3932,633.4006,701.3885,702.3922	Terpenoids	Ecliptasaponin IX	72436890
114	C36H58O12S	23.431	714.3649	714.3668	2.63	96.9585,166.9650,241.0018,300.6961,504.6021,713.3580,714.3611	Terpenoids	Ecliptasaponin V	5.4E+08
115	C36H58O9	23.512	634.4081	634.4094	2.04	101.0229,161.0447,206.6948,453.9695,523.8905,576.2598,633.4008,634.4048	Terpenoids	Ecliptasaponin A/D	64644637
116	C16H12O5	23.796	284.0685	284.0687	0.94		Flavonoids	Daphnetin	18997215
117	C21H20O6	24.306	368.126	368.1263	0.92	108.0440,238.0868,367.1205,456.1494	Flavonoids	Topazolin	4.48E+08
118	C20H18O6	25.694	354.1103	354.1109	1.54	125.0230,243.1023,284.0323,353.1030,440.8607	Lignans	(+)-Sesamin	3.42E+08
119	C36H54O10	25.878	646.3717	646.3731	2.21	75.0072,157.0131,227.6633,365.6607,469.3317,627.3540,645.3647,646.	Terpenoids	Glycyrrhetic acid 3-O-glucuronide	1.77E+08

						3681			
120	C22H22O6	26.184	382.1416	382.1422	1.46	109.0278,149.0593,201.0181,351.0872,381.1343,382.1376,391.1477	Flavonoids	Licoricone	1.02E+08
121	C21H18O6	26.402	366.1103	366.1108	1.24	69.2870,141.6559,295.0243,307.0244,365.1025	Flavonoids	Glycyrol	1.79E+08
122	C38H60O10	26.496	676.4187	676.4202	2.31	59.0124,101.0231,161.0443,615.4000,675.4115,676.4153	Terpenoids	3-O-(2-O-acetyl-β-D-glucopyranosyl) oleanolic acid	65172371
123	C20H16O5	27.2	336.0998	336.1002	1.37	129.9736,215.5954,305.0449,335.0925	Flavonoids	kanzonol W	1.03E+08
124	C20H20O4	27.624	324.1362	324.1365	1.03	135.0438,201.0912,213.0914,323.1290,324.1321	Flavonoids	Glabranin	98705030
125	C20H16O6	28.317	352.0947	352.0951	1.1	83.0123,199.0753,283.0973,351.0873,352.0906,453.9301	Flavonoids	Citrusinol	4.1E+08
126	C20H16O5	29.266	336.0998	336.1003	1.46		Flavonoids	kanzonol W	75150807
127	C25H28O4	29.464	392.1988	392.1995	1.83	132.0566,187.1118,221.0813,286.9193,391.1914,392.1948	Flavonoids	Hispaglabridin A	1.96E+08
128	C25H28O6	30.563	424.1886	424.1893	1.78		Flavonoids	Glyinflanin E	84604218
129	C15H22O2	30.642	234.162	234.1618	-0.69	70.5321,233.1542,234.1576	Terpenoids	[3S-(3alpha,4abeta,5beta,8aalpha)]-Octahydro-4a,5-dimethyl-3-(1-methylethenyl)-1,7-naphthalenedione	69412119
130	C30H44O5	30.658	484.3189	484.3196	1.46	70.4269,127.8903,181.6620,409.2760,483.3092,484.3123	Terpenoids	Poricoic acid B	1.13E+08
131	C25H28O6	31.675	424.1886	424.1893	1.57	57.0332,149.0960,193.0861,229.0865,230.0899,423.1810,424.1848	Flavonoids	Glyinflanin E	84718585

132	C25H26O6	31.963	422.1729	422.1737	1.71	70.0005,188.0106,239.0714,309.0404,365.1025,421.1656,422.1690	Flavonoids	Glyasperin A	1.27E+08
133	C31H46O5	32.064	498.3345	498.3353	1.62		Terpenoids	Poricoic acid A	73183384
134	C30H46O5	32.248	486.3345	486.3354	1.78		Terpenoids	Poricoic acid G	58365639
135	C25H48O11S	32.842	556.2917	556.2926	1.48	80.9636,164.9850,225.0071,299.0461,418.9182,469.3294,555.2840,556.2877	Phenylpropanoids	1-O-palmitoyl-3-O-(6'-thio-α-D-deoxypyranosyl) glycerol	23587052
136	C25H28O4	35.288	392.1988	392.1995	1.83		Flavonoids	Hispaglabridin A	80503311
137	C30H38O4	35.487	462.277	462.2753	-3.6		Terpenoids	Bislactone Atractylenolide	14346044
138	C36H58O8	44.181	618.4132	618.4145	2.16		Terpenoids	Chikusetsusaponin I	37609092
