# Supplementary figures and images for: Systems Network Integration of Transcriptomic, Proteomic, and Bioinformatic Analyses Reveals the Mechanism of XuanYunNing Tablets in Meniere’s Disease via JAK-STAT Pathway Modulation (part 2 of 2)
Source: Pharmaceuticals (Basel). 2025 Aug 25;18(9):1266. doi: 10.3390/ph18091266 (PMC12472466; doi:10.3390/ph18091266)

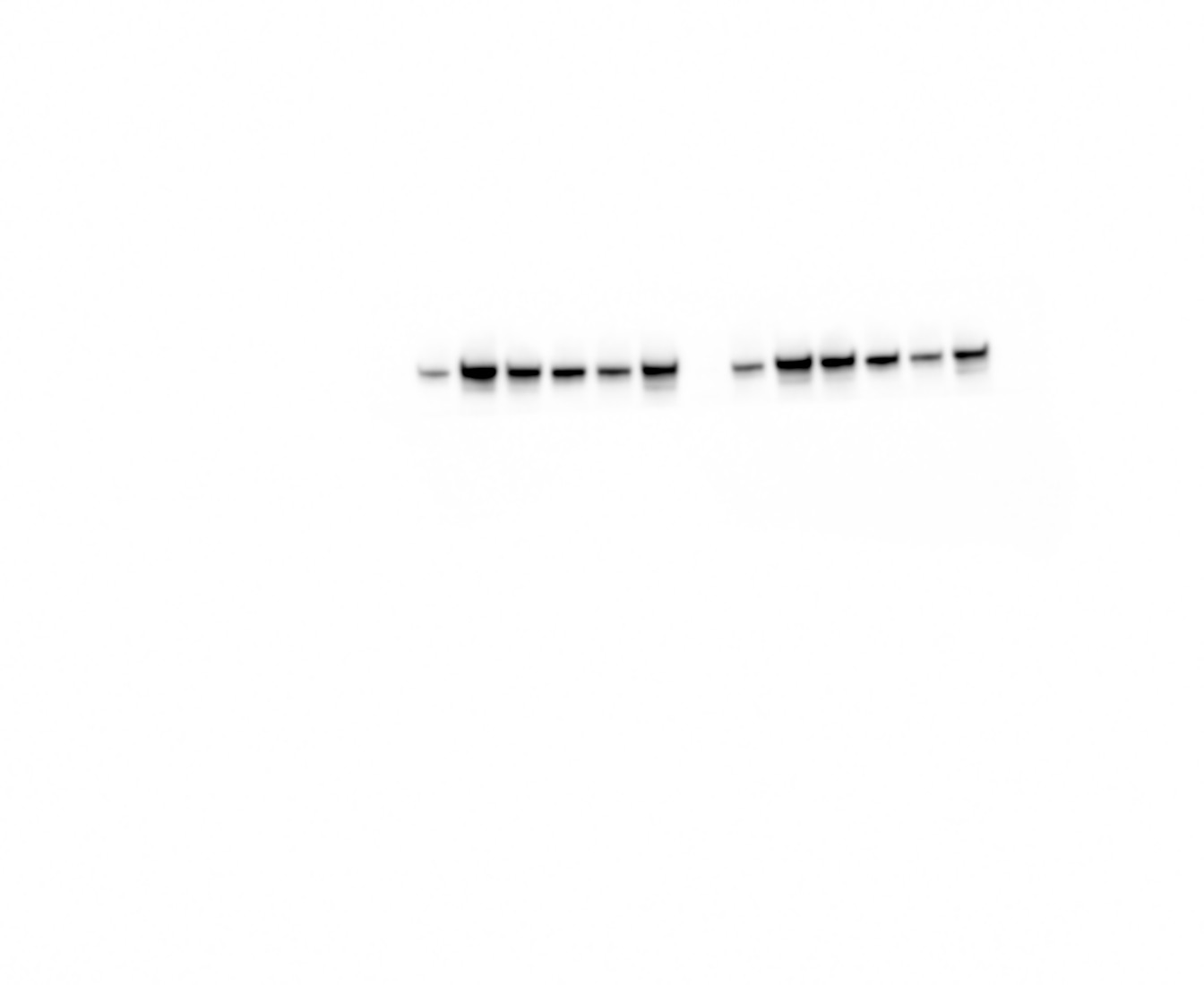

Supplement: Supplementary file 1 [file pharmaceuticals-18-01266-s001.zip › Western blot/p-STAT3/n1-n2 [Luminescence][p-STAT3].tif]

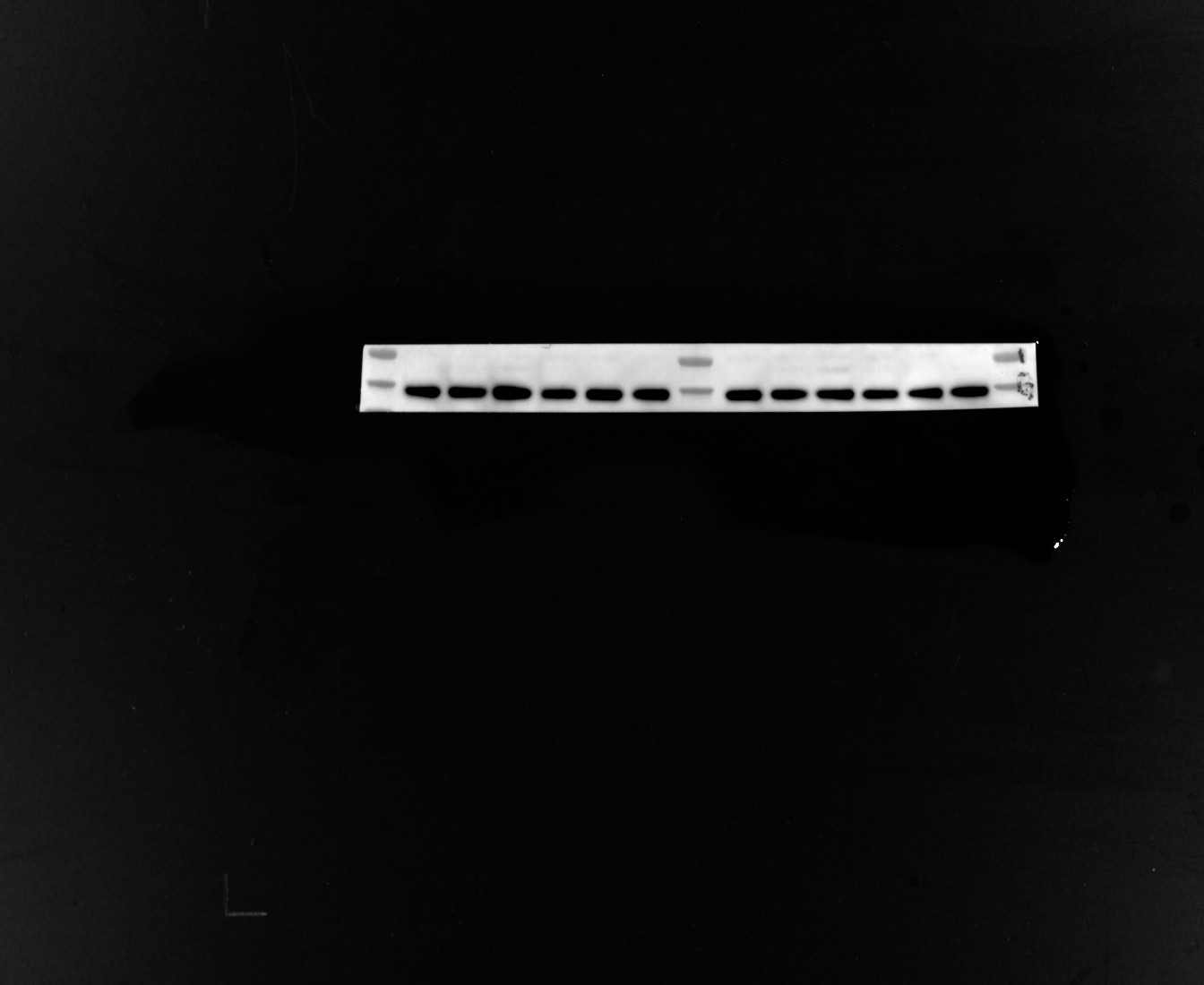

Supplement: Supplementary file 1 [file pharmaceuticals-18-01266-s001.zip › Western blot/p-STAT3/n1-n2 [Overlay][GAPDH].tif]

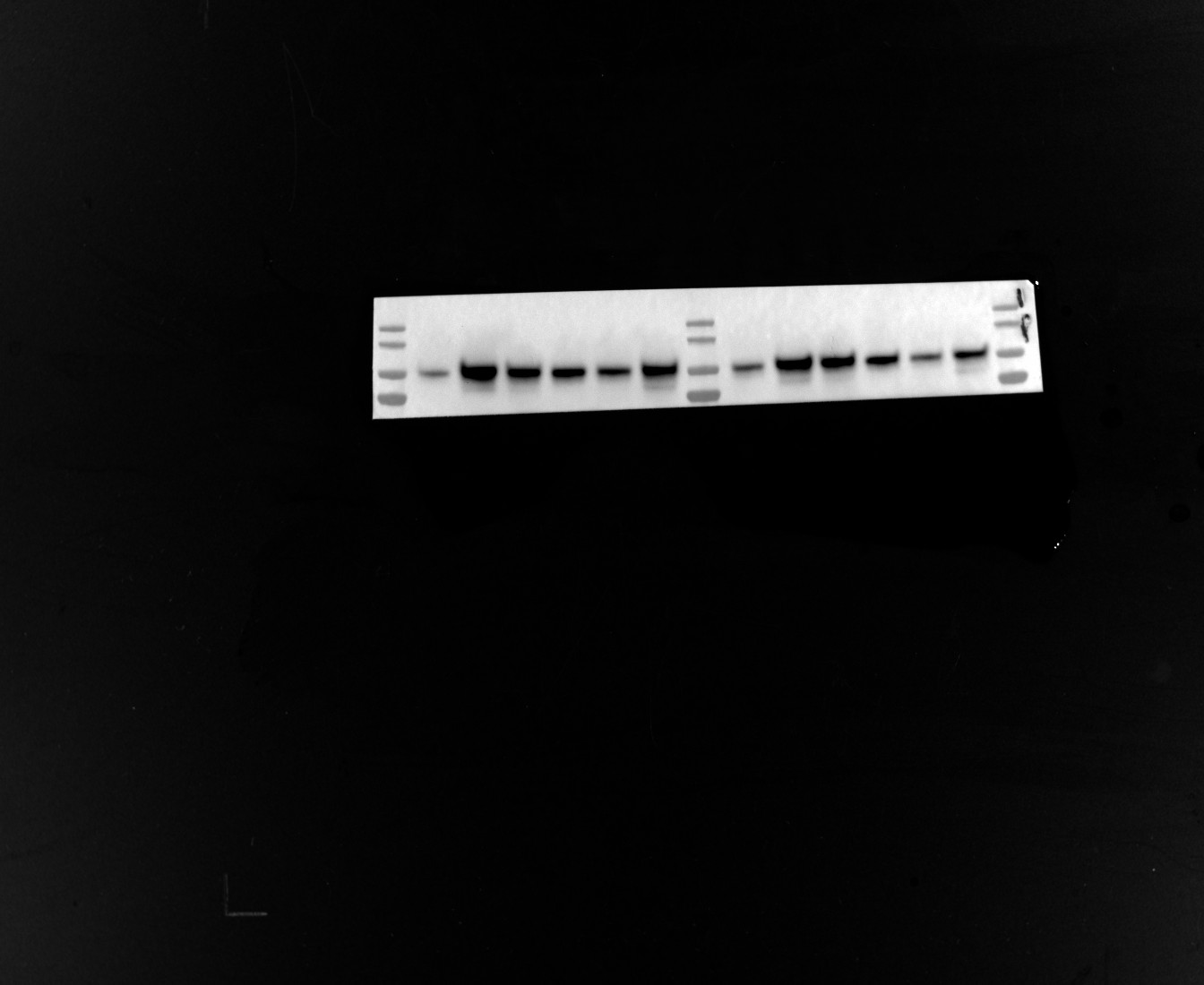

Supplement: Supplementary file 1 [file pharmaceuticals-18-01266-s001.zip › Western blot/p-STAT3/n1-n2 [Overlay][p-STAT3].tif]

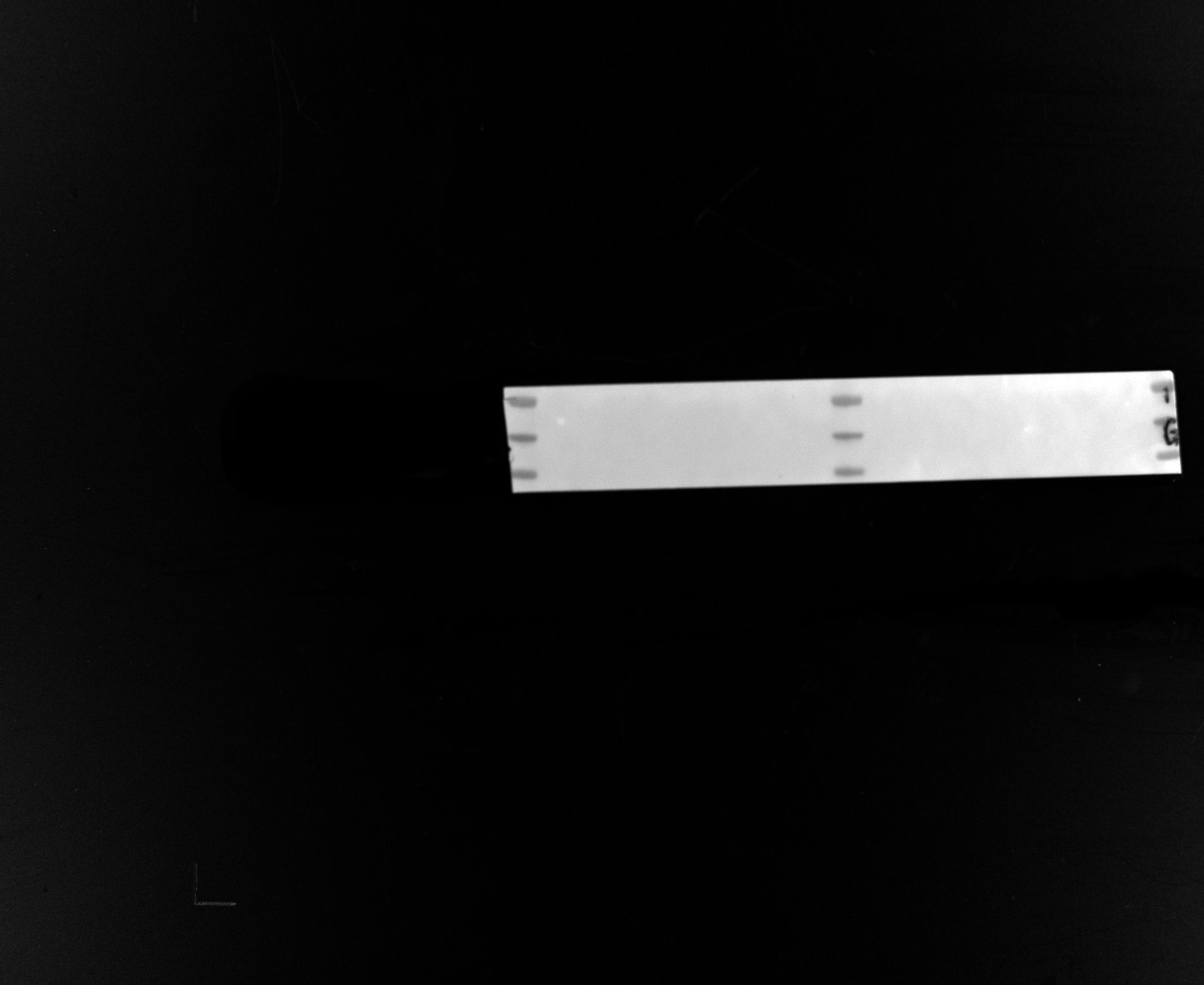

Supplement: Supplementary file 1 [file pharmaceuticals-18-01266-s001.zip › Western blot/p-STAT3/n3-n4 [Brightfield][GAPDH].tif.tif]

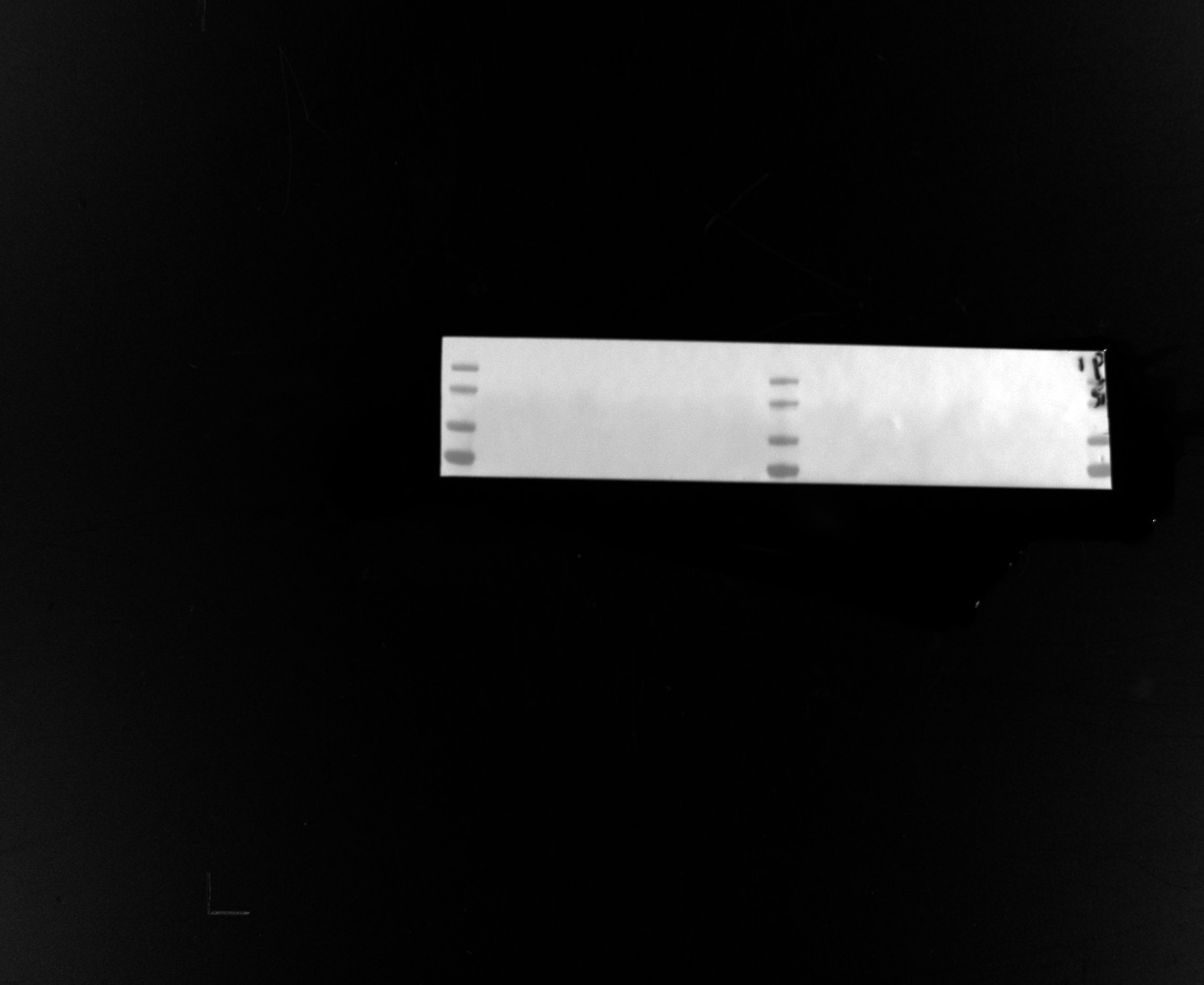

Supplement: Supplementary file 1 [file pharmaceuticals-18-01266-s001.zip › Western blot/p-STAT3/n3-n4 [Brightfield][p-STAT3].tif]

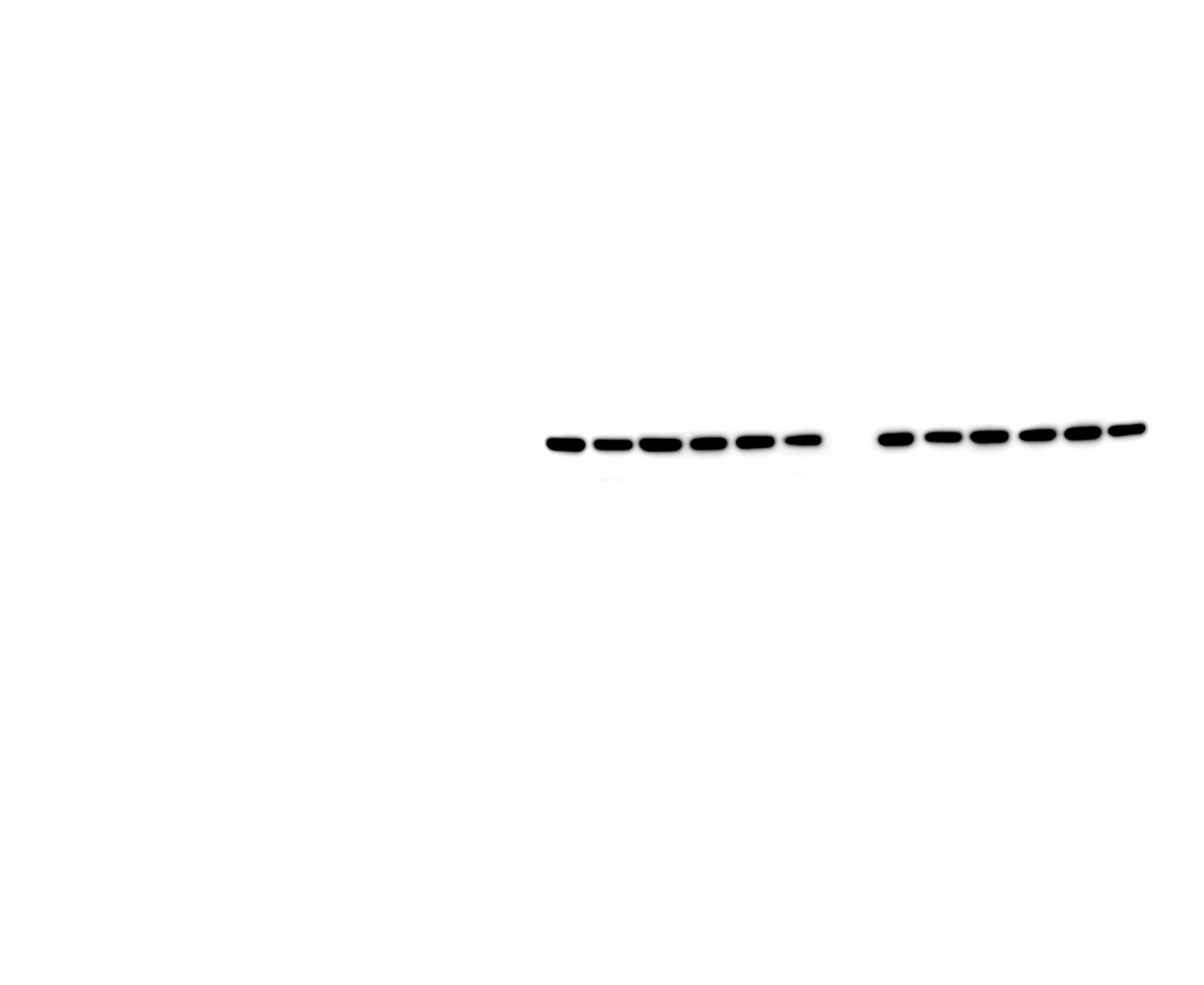

Supplement: Supplementary file 1 [file pharmaceuticals-18-01266-s001.zip › Western blot/p-STAT3/n3-n4 [Luminescence][GAPDH].tif]

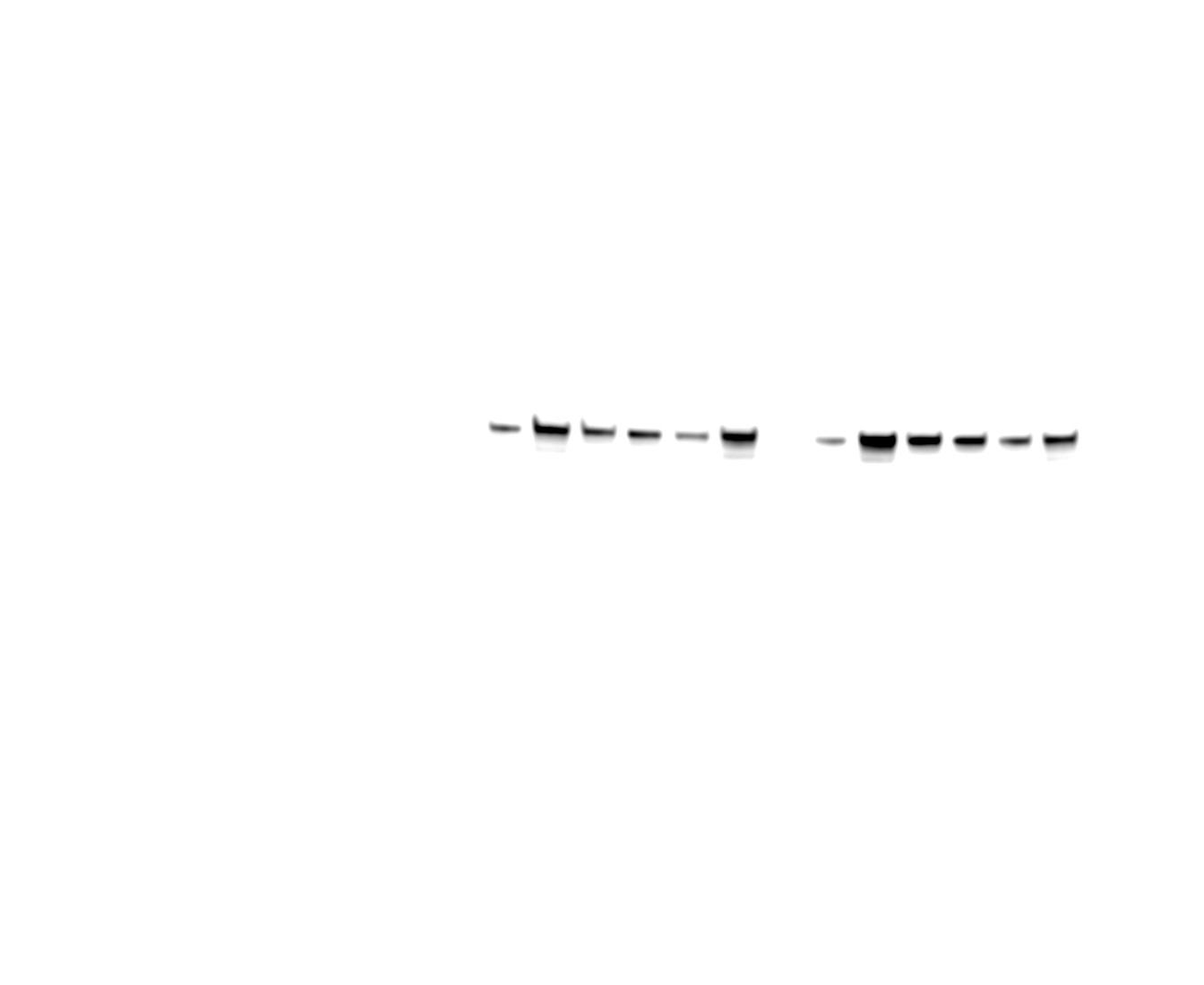

Supplement: Supplementary file 1 [file pharmaceuticals-18-01266-s001.zip › Western blot/p-STAT3/n3-n4 [Luminescence][p-STAT3].tif]

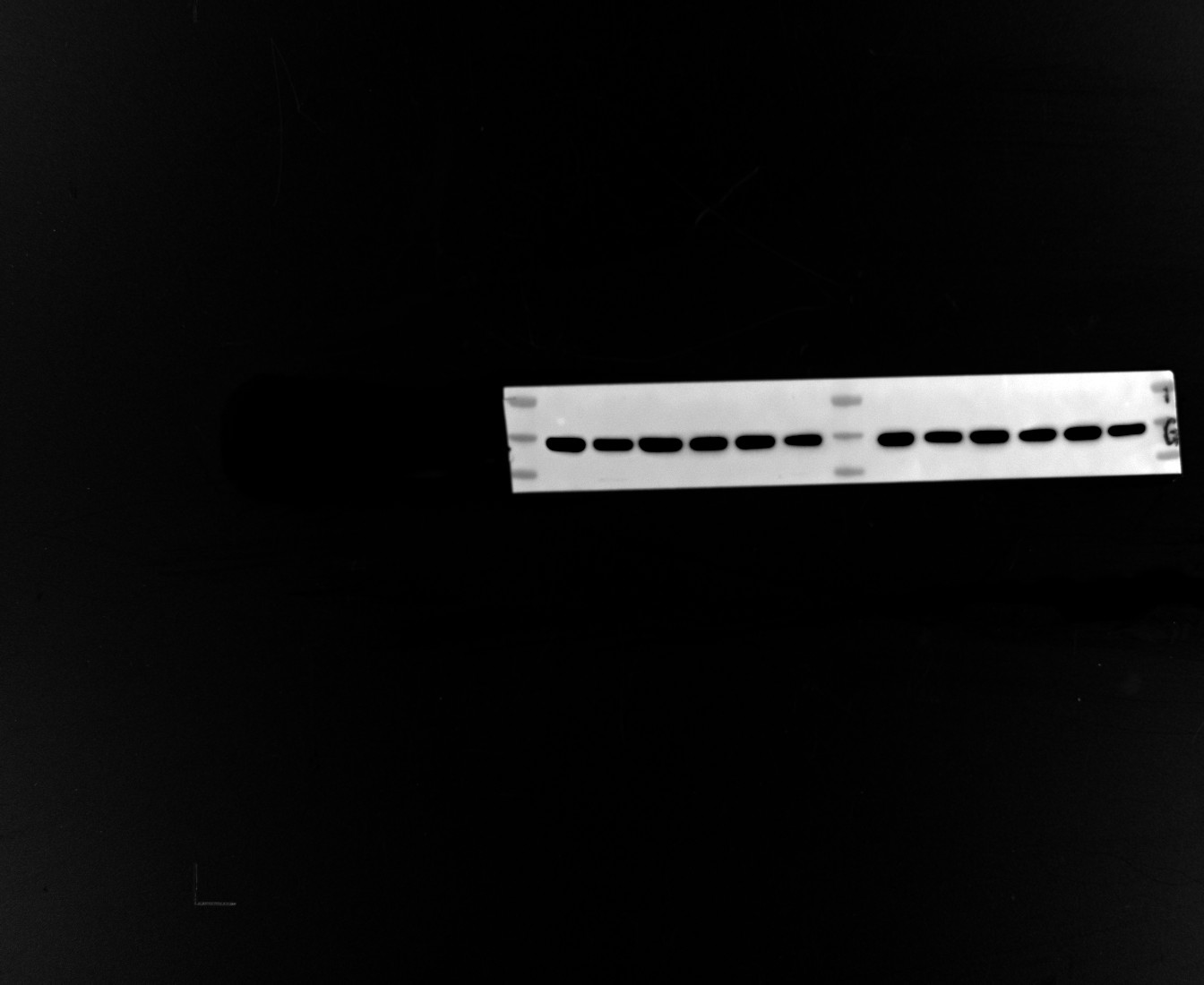

Supplement: Supplementary file 1 [file pharmaceuticals-18-01266-s001.zip › Western blot/p-STAT3/n3-n4 [Overlay][GAPDH].tif]

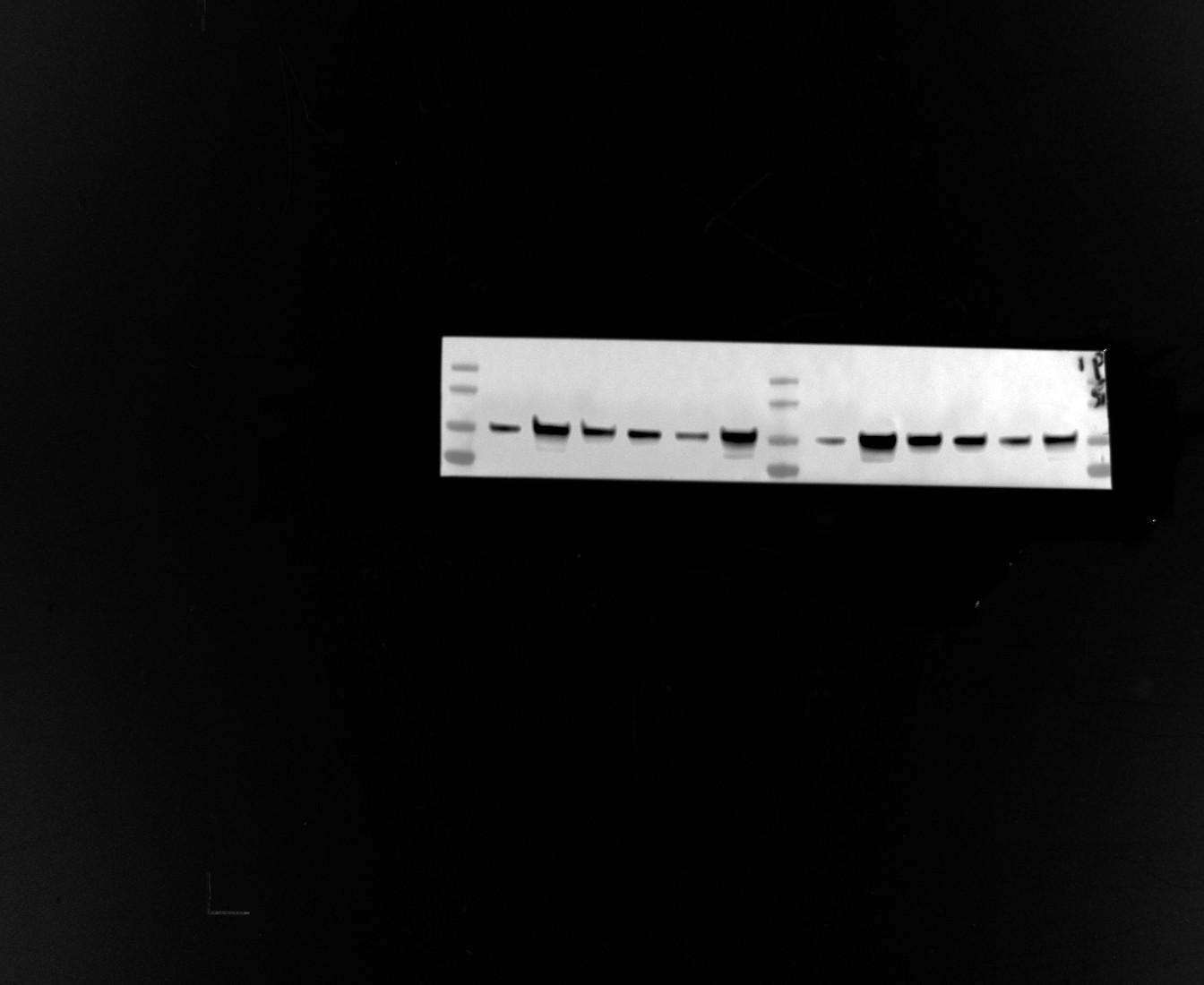

Supplement: Supplementary file 1 [file pharmaceuticals-18-01266-s001.zip › Western blot/p-STAT3/n3-n4 [Overlay][p-STAT3].tif]

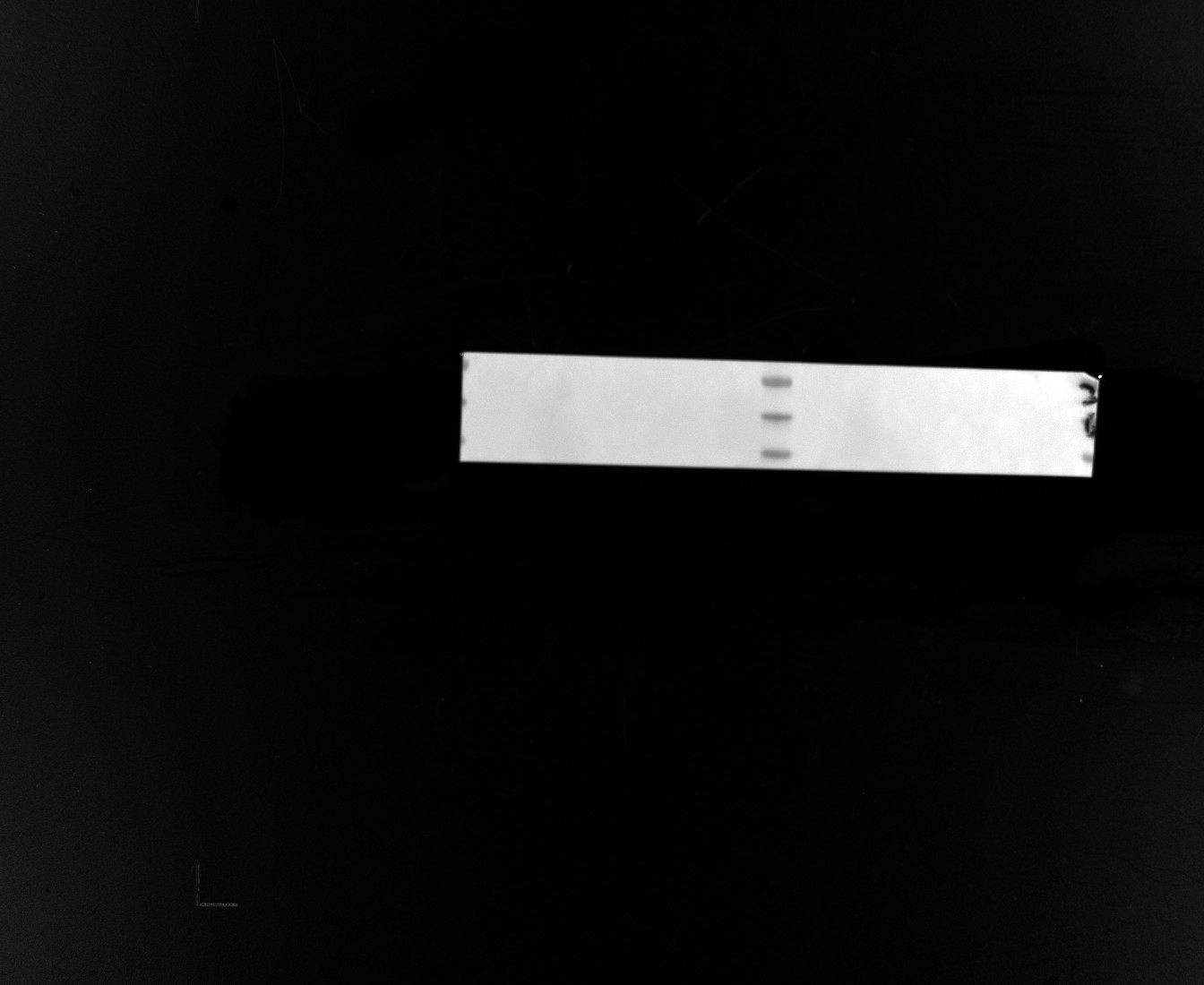

Supplement: Supplementary file 1 [file pharmaceuticals-18-01266-s001.zip › Western blot/p-STAT3/n5-n6 [Brightfield][GAPDH].tif]

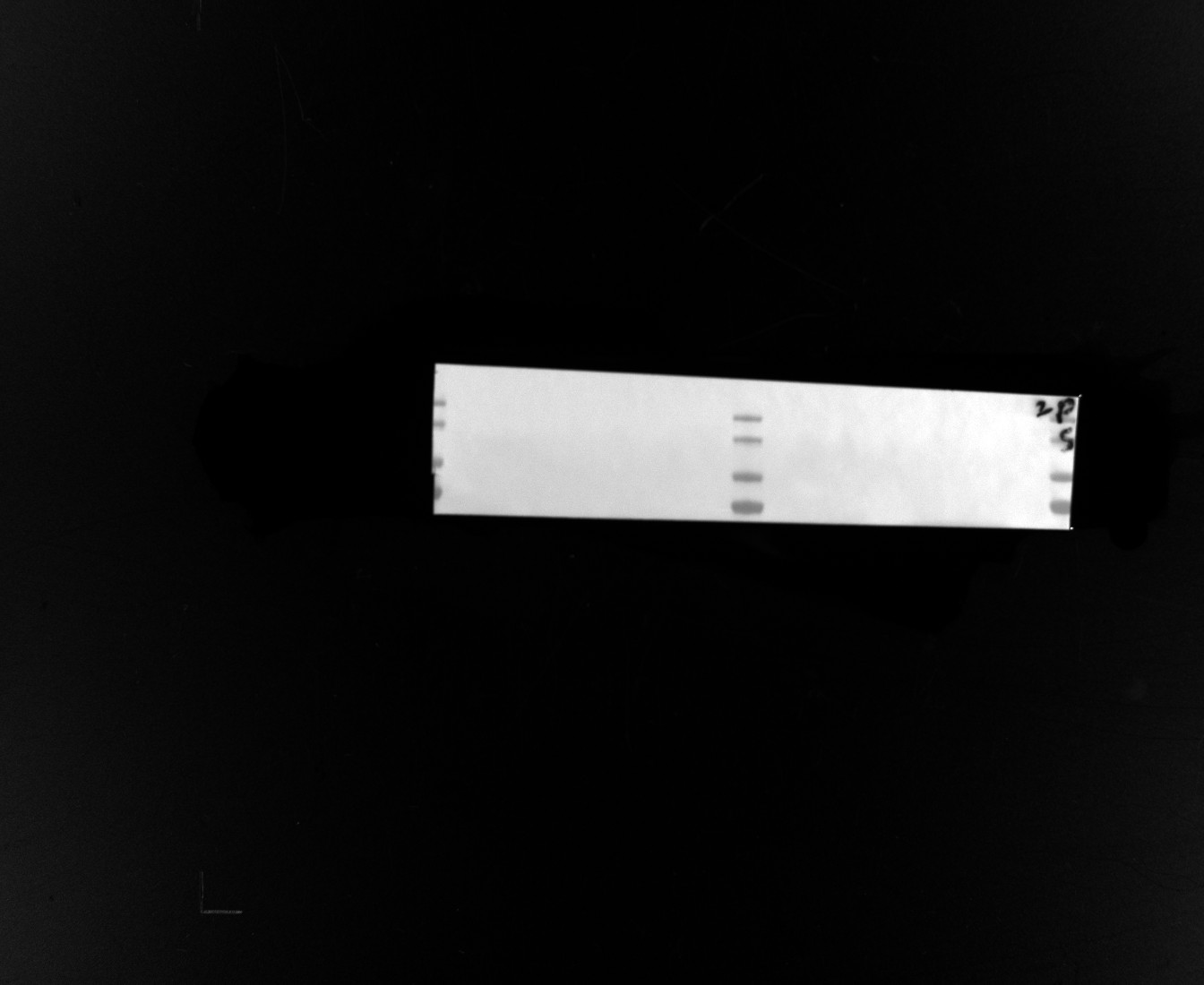

Supplement: Supplementary file 1 [file pharmaceuticals-18-01266-s001.zip › Western blot/p-STAT3/n5-n6 [Brightfield][p-STAT3].tif]

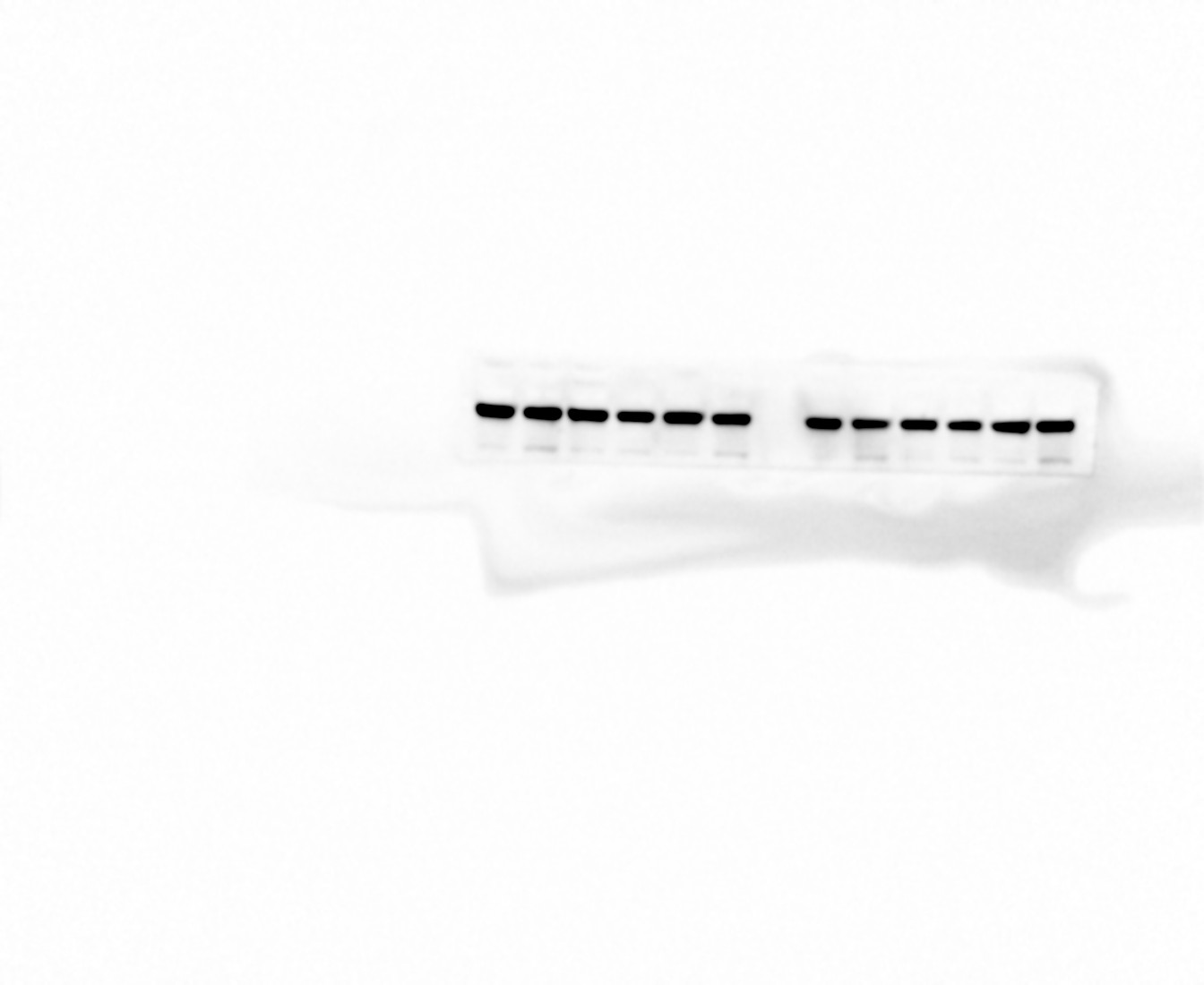

Supplement: Supplementary file 1 [file pharmaceuticals-18-01266-s001.zip › Western blot/p-STAT3/n5-n6 [Luminescence][GAPDH].tif]

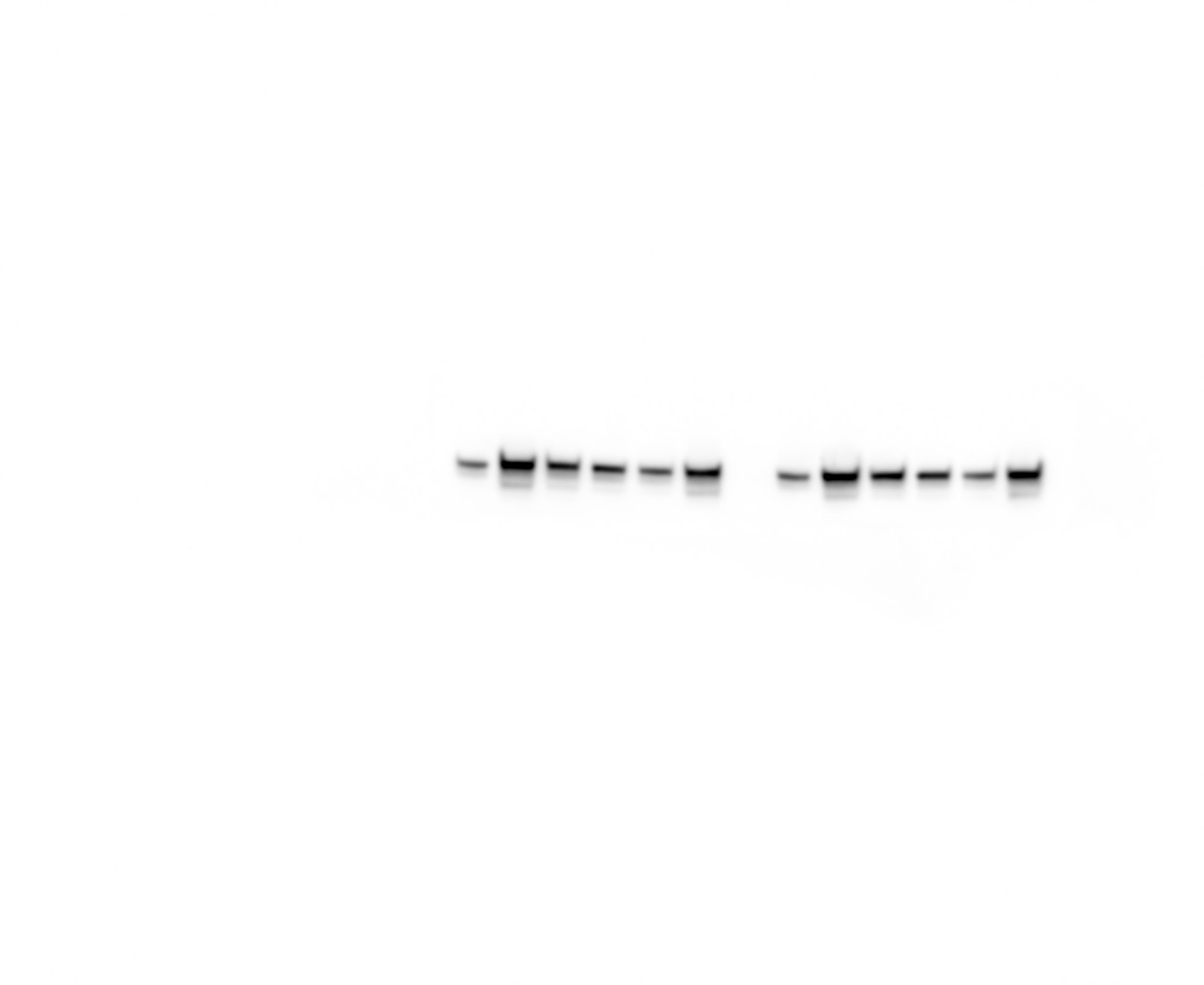

Supplement: Supplementary file 1 [file pharmaceuticals-18-01266-s001.zip › Western blot/p-STAT3/n5-n6 [Luminescence][p-STAT3].tif]

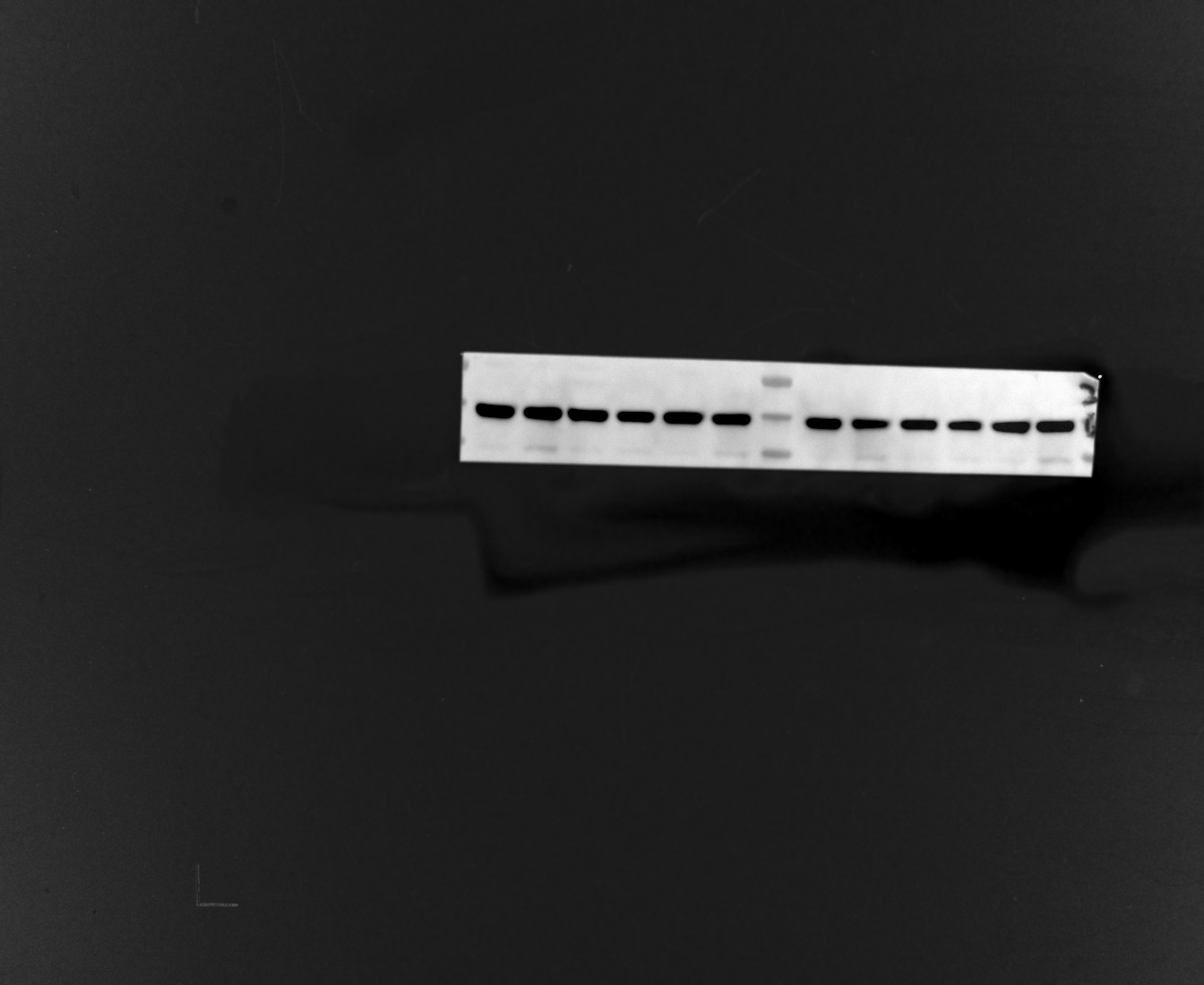

Supplement: Supplementary file 1 [file pharmaceuticals-18-01266-s001.zip › Western blot/p-STAT3/n5-n6 [Overlay][GAPDH].tif]

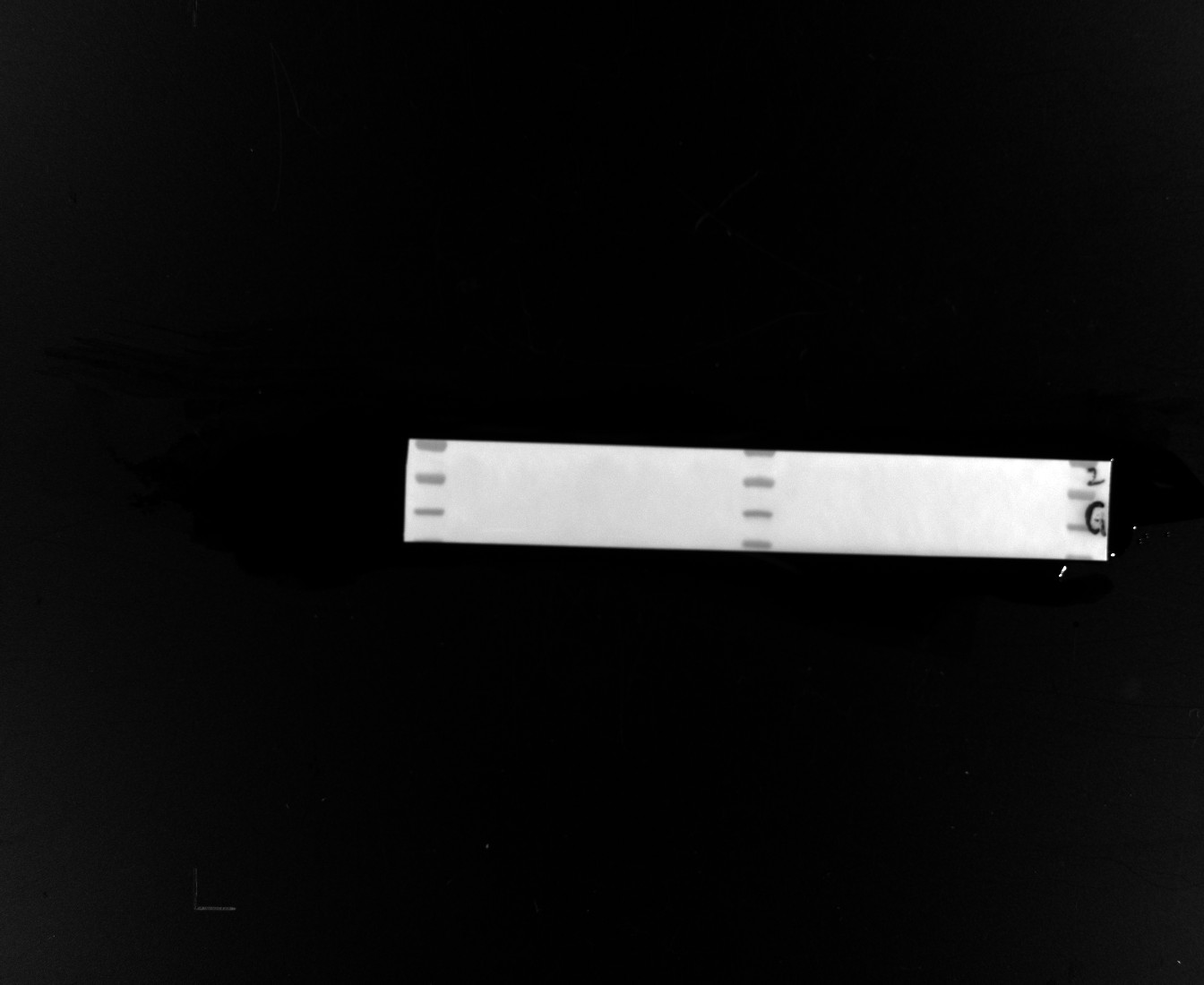

Supplement: Supplementary file 1 [file pharmaceuticals-18-01266-s001.zip › Western blot/STAT3/n1-n2 [Brightfield][GAPDH].tif]

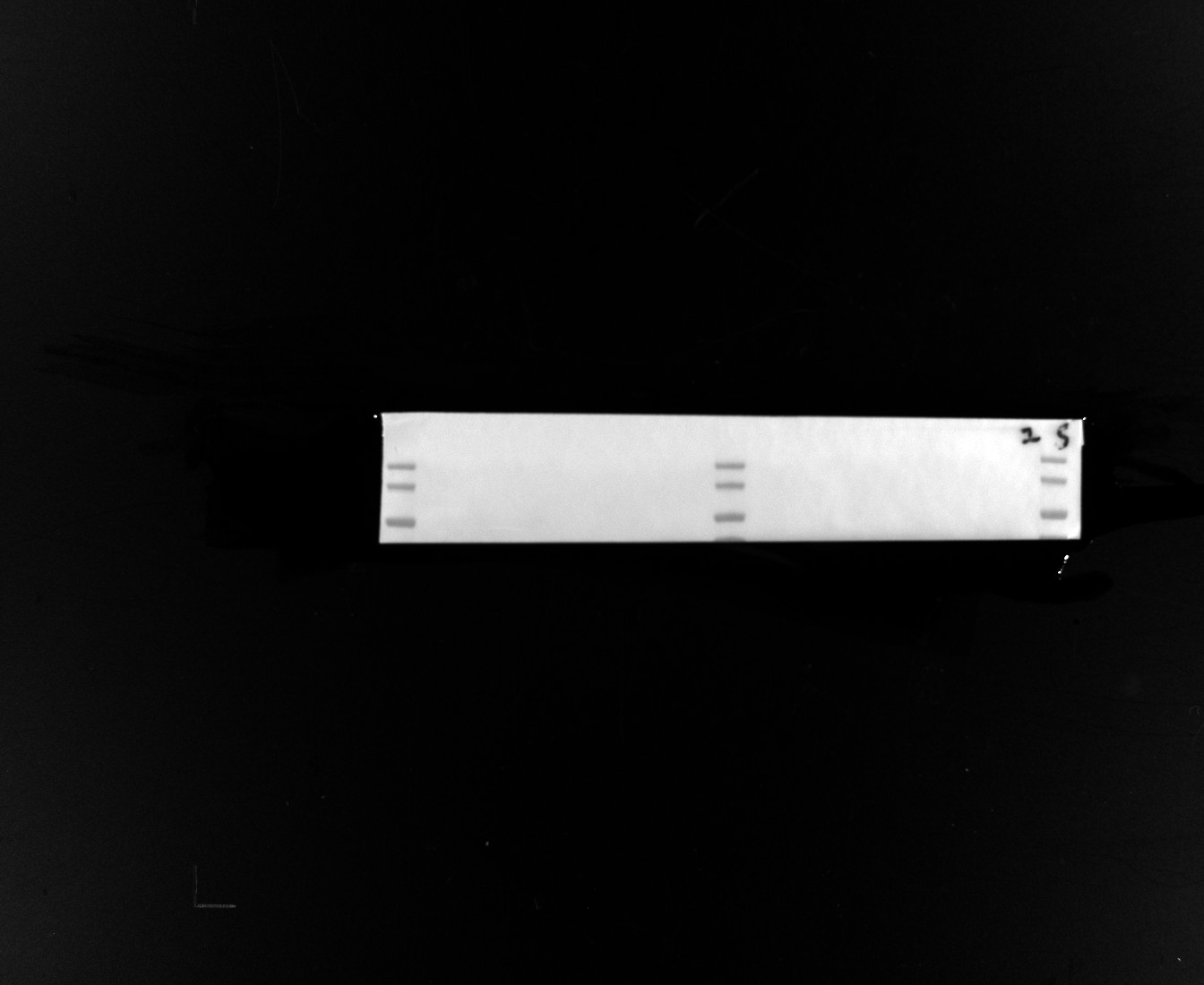

Supplement: Supplementary file 1 [file pharmaceuticals-18-01266-s001.zip › Western blot/STAT3/n1-n2 [Brightfield][STAT3].tif]

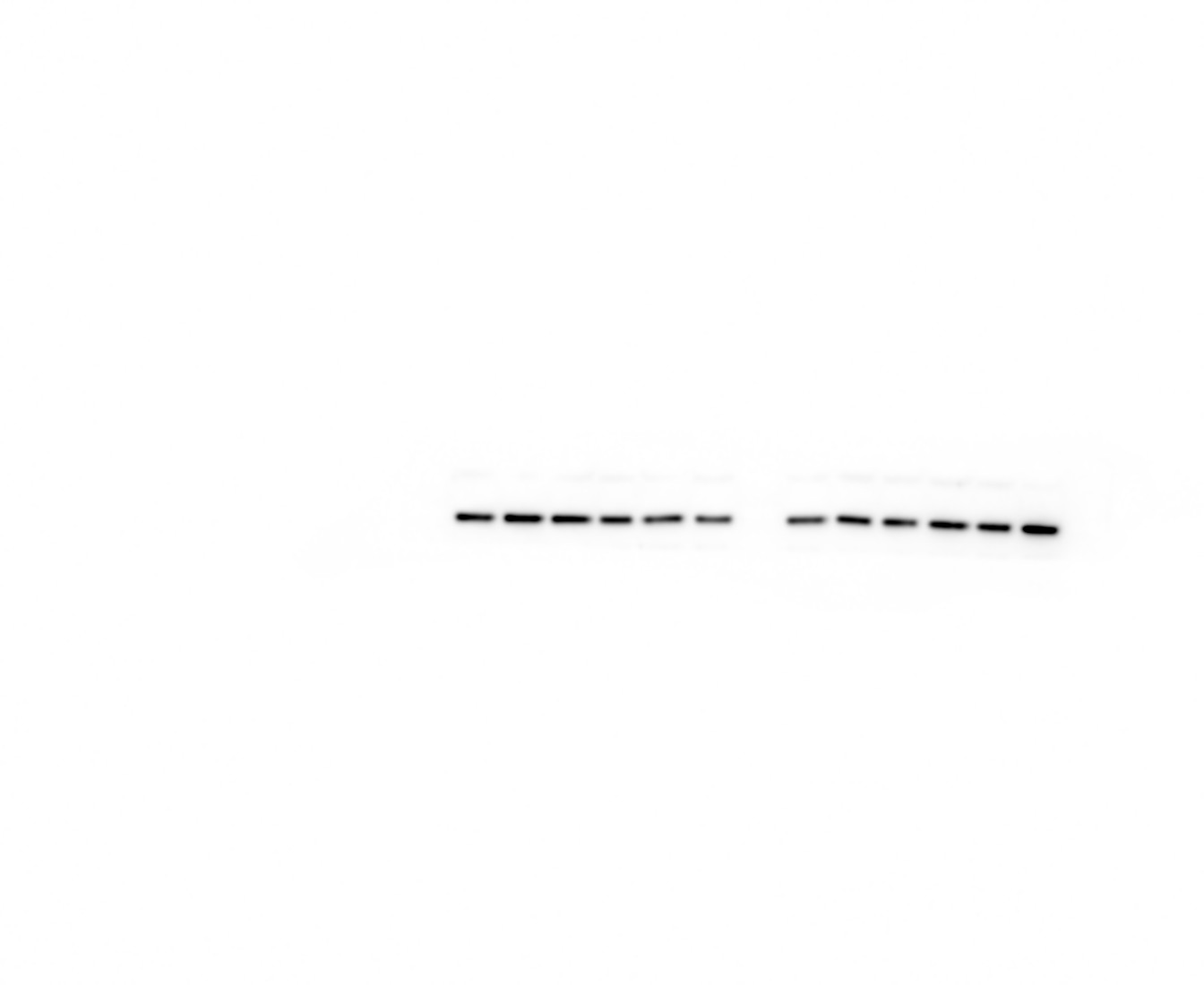

Supplement: Supplementary file 1 [file pharmaceuticals-18-01266-s001.zip › Western blot/STAT3/n1-n2 [Luminescence][GAPDH].tif]

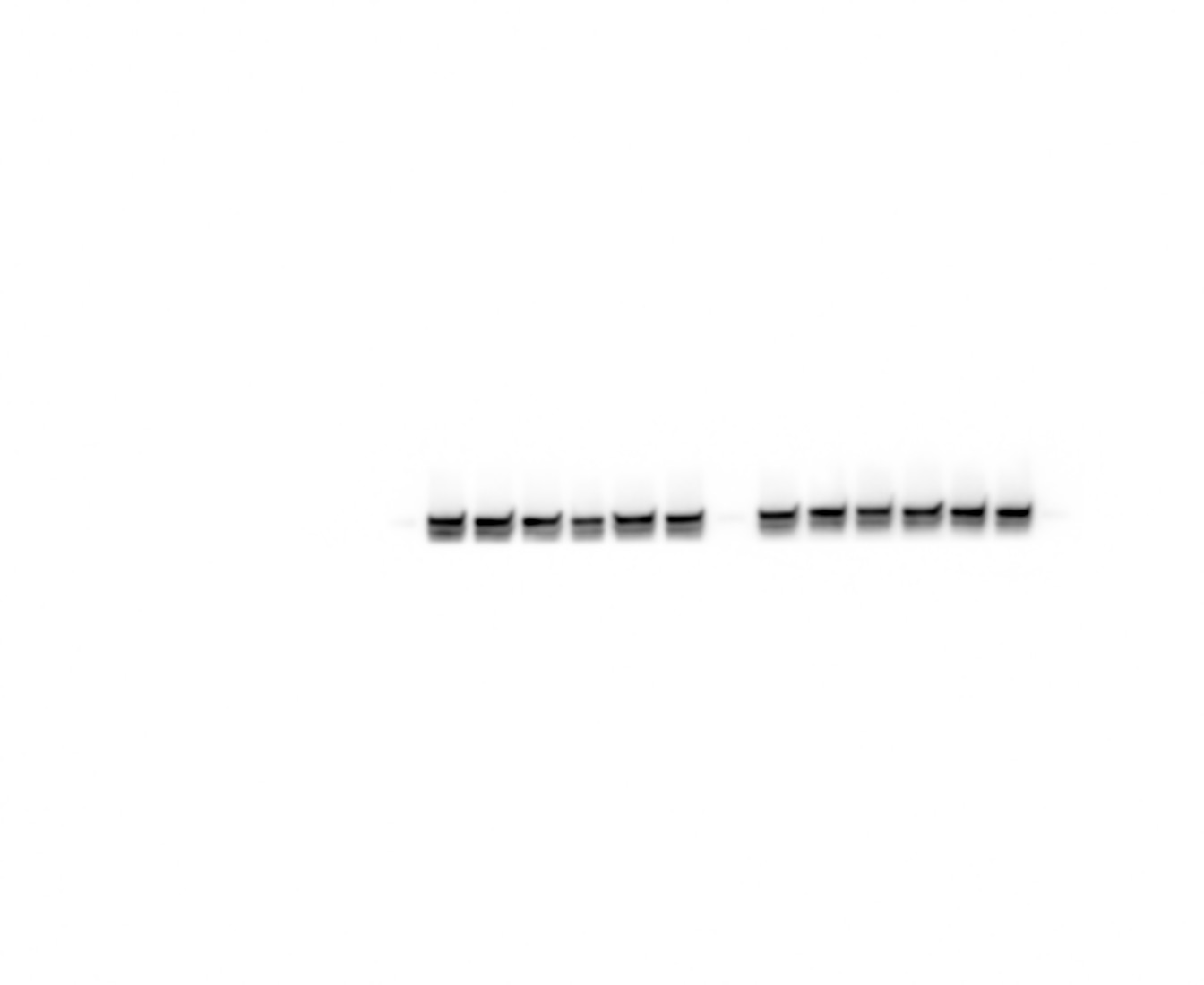

Supplement: Supplementary file 1 [file pharmaceuticals-18-01266-s001.zip › Western blot/STAT3/n1-n2 [Luminescence][STAT3].tif]

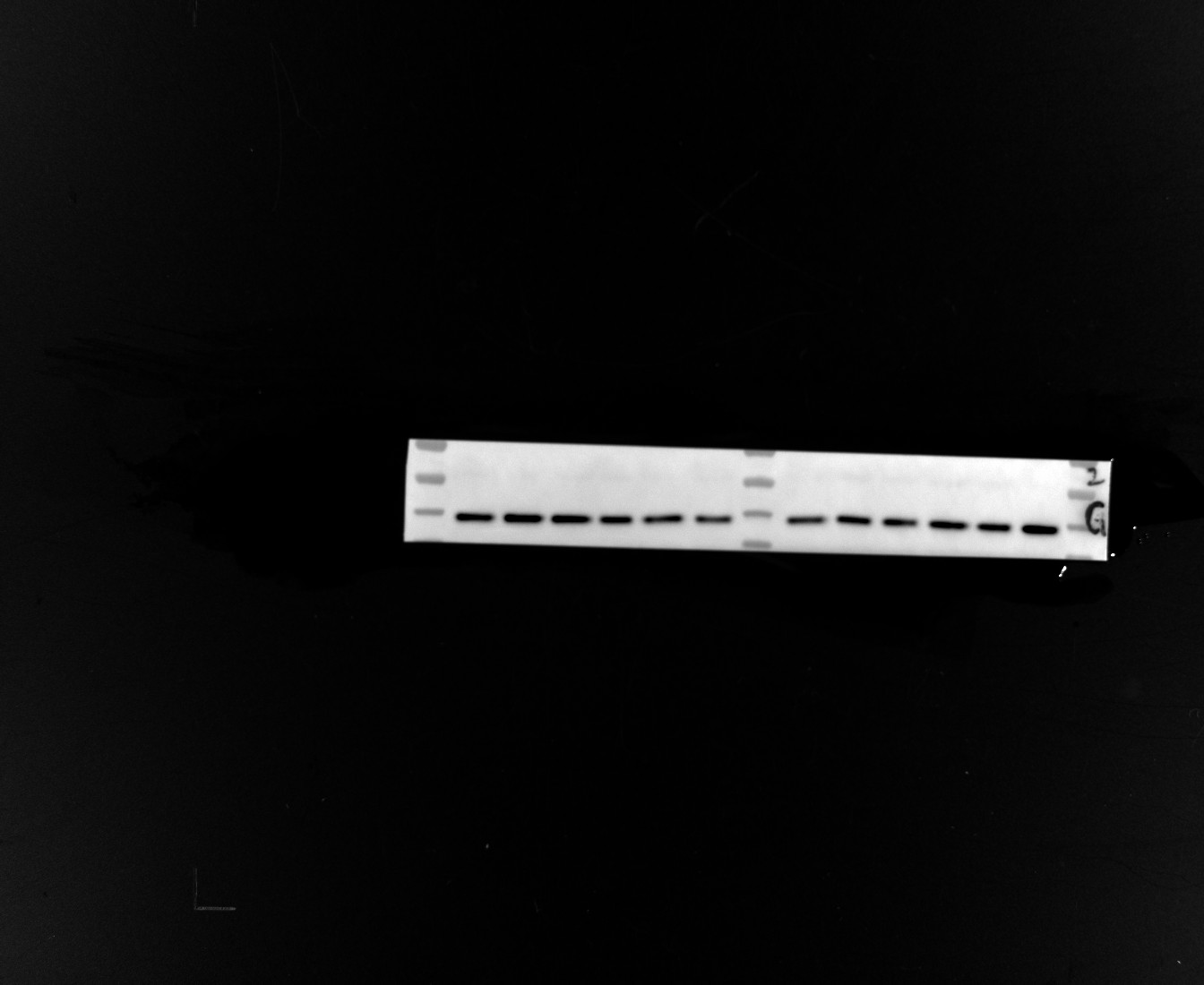

Supplement: Supplementary file 1 [file pharmaceuticals-18-01266-s001.zip › Western blot/STAT3/n1-n2 [Overlay][GAPDH].tif]

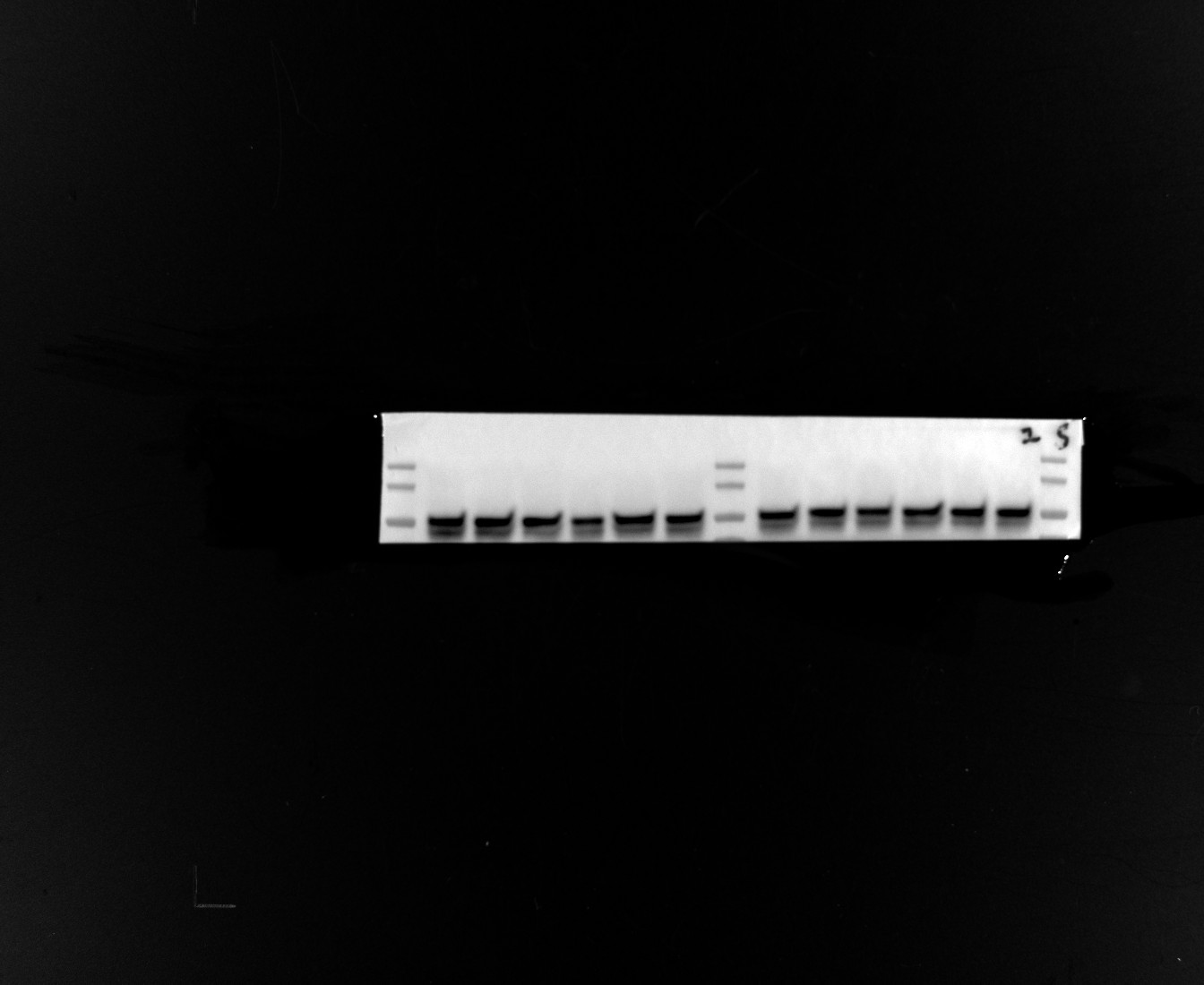

Supplement: Supplementary file 1 [file pharmaceuticals-18-01266-s001.zip › Western blot/STAT3/n1-n2 [Overlay][STAT3].tif]

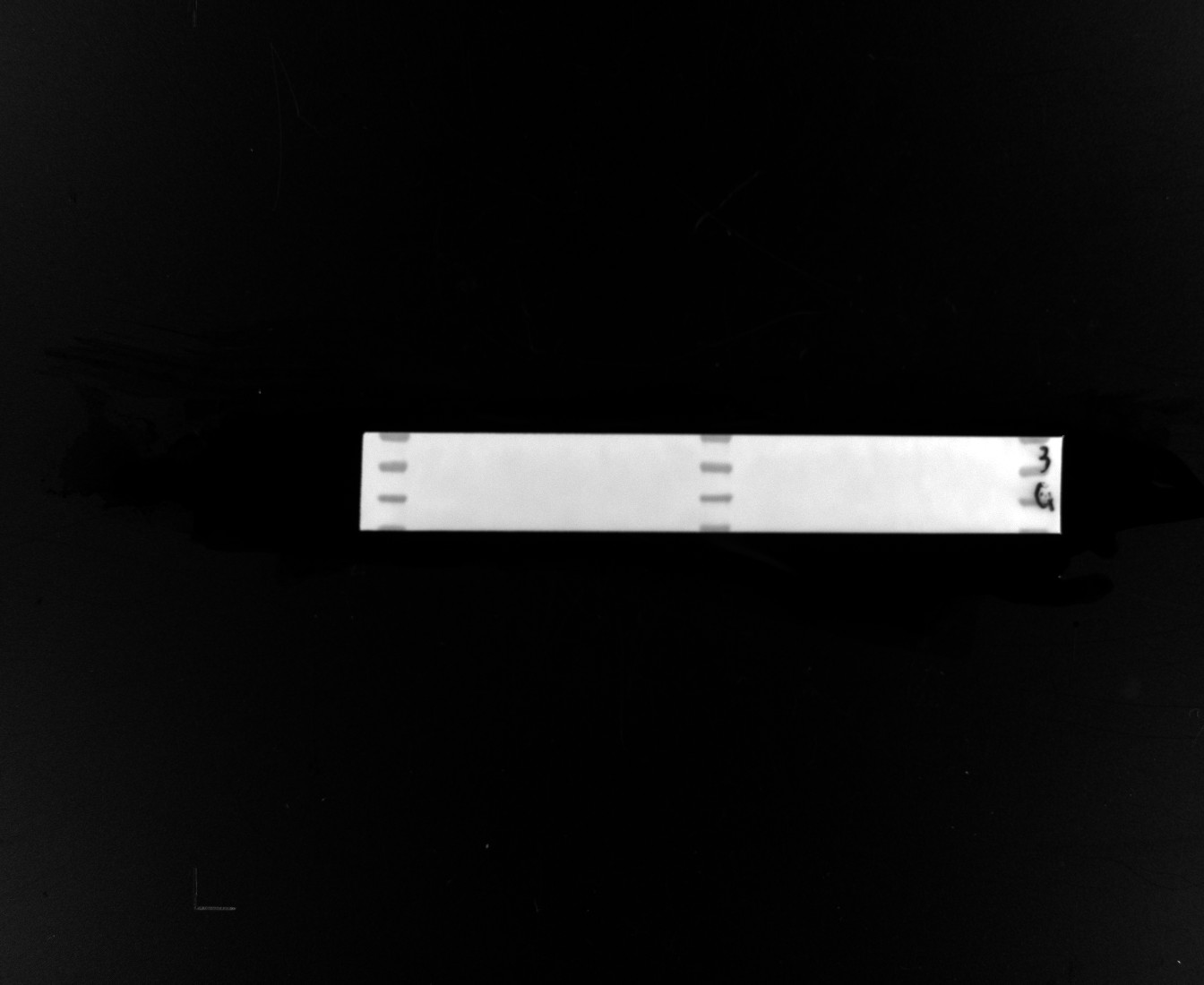

Supplement: Supplementary file 1 [file pharmaceuticals-18-01266-s001.zip › Western blot/STAT3/n3-n4 [Brightfield][GAPDH].tif]

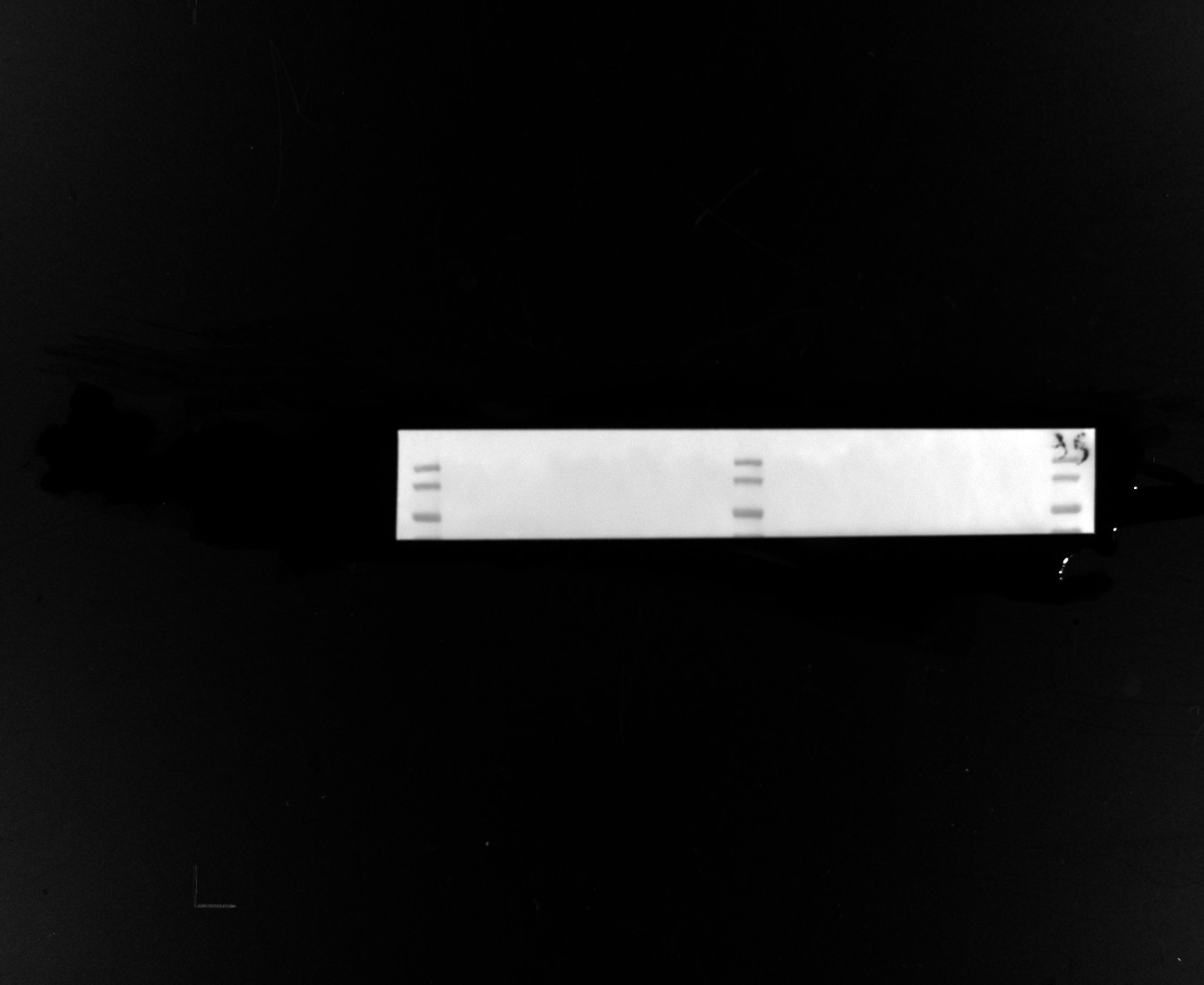

Supplement: Supplementary file 1 [file pharmaceuticals-18-01266-s001.zip › Western blot/STAT3/n3-n4 [Brightfield][STAT3].tif]

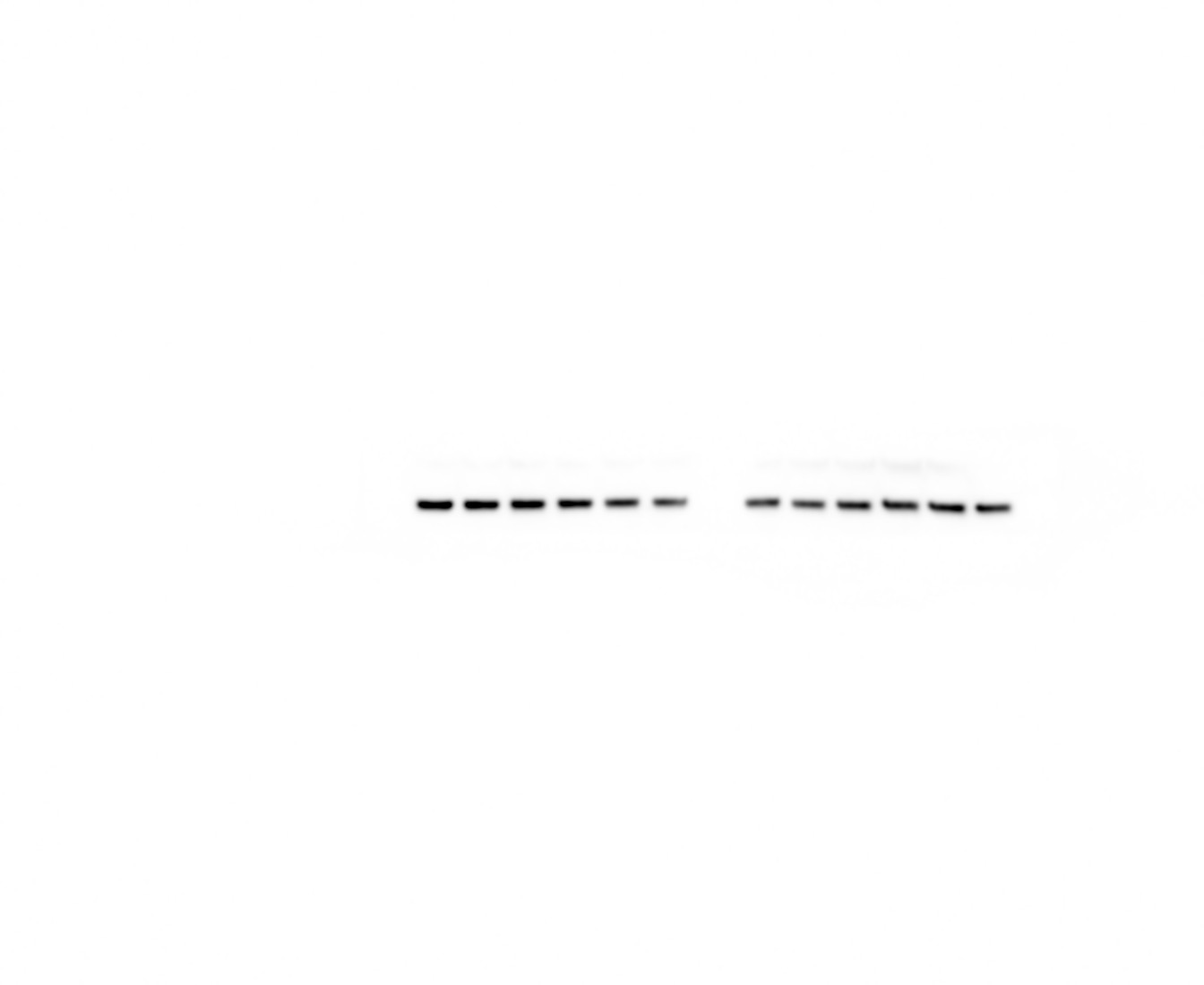

Supplement: Supplementary file 1 [file pharmaceuticals-18-01266-s001.zip › Western blot/STAT3/n3-n4 [Luminescence][GAPDH].tif]

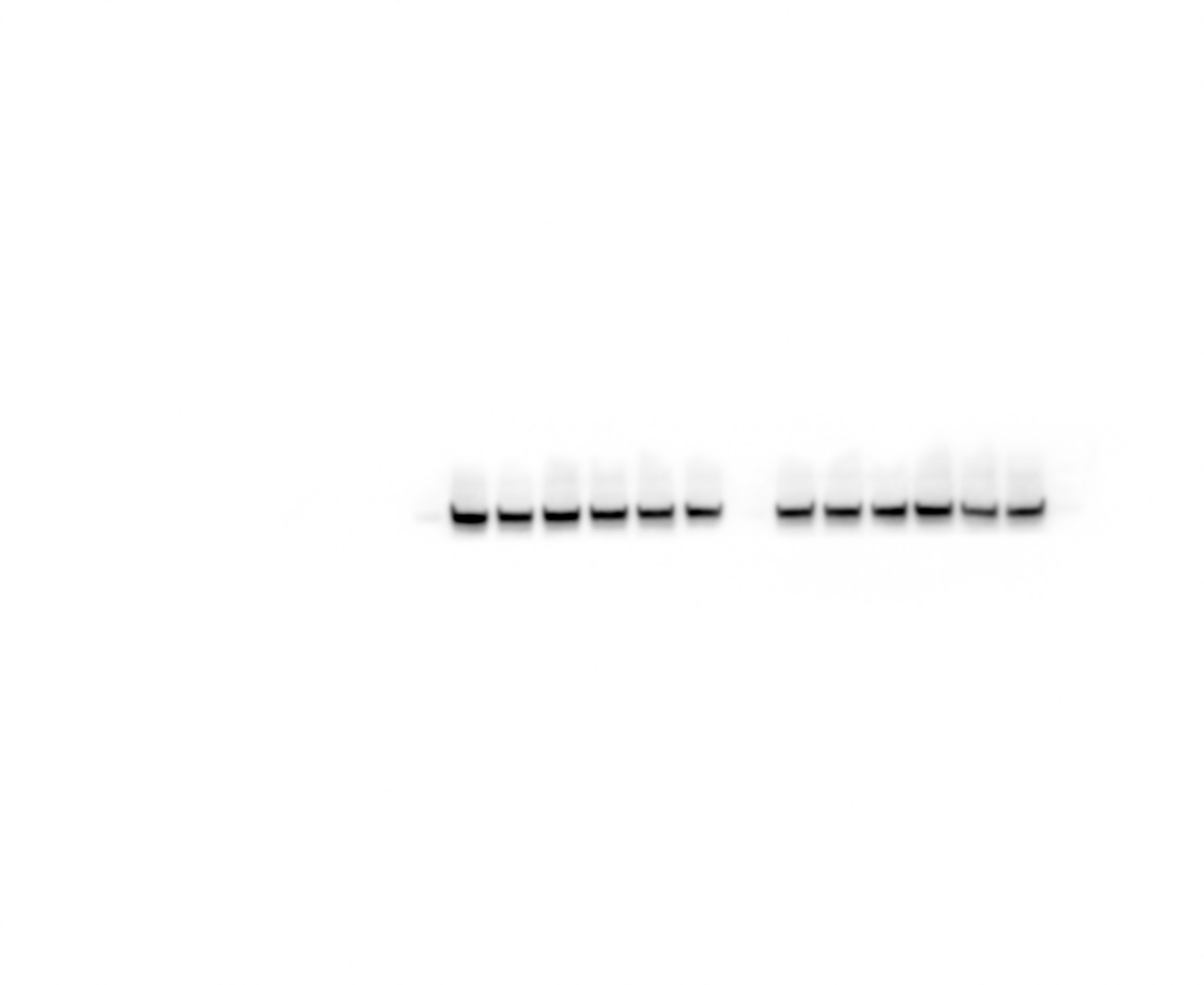

Supplement: Supplementary file 1 [file pharmaceuticals-18-01266-s001.zip › Western blot/STAT3/n3-n4 [Luminescence][STAT3].tif]

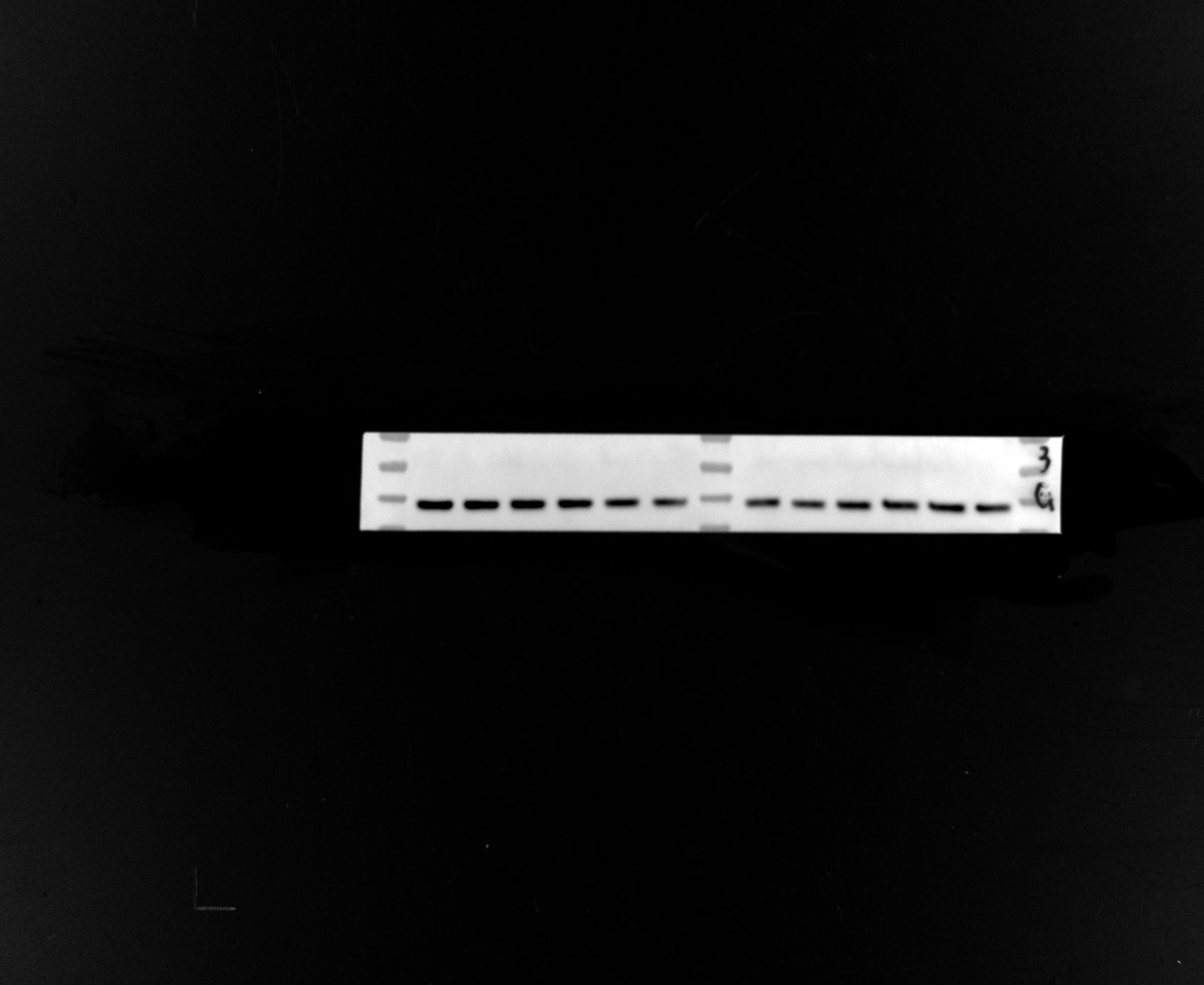

Supplement: Supplementary file 1 [file pharmaceuticals-18-01266-s001.zip › Western blot/STAT3/n3-n4 [Overlay][GAPDH].tif]

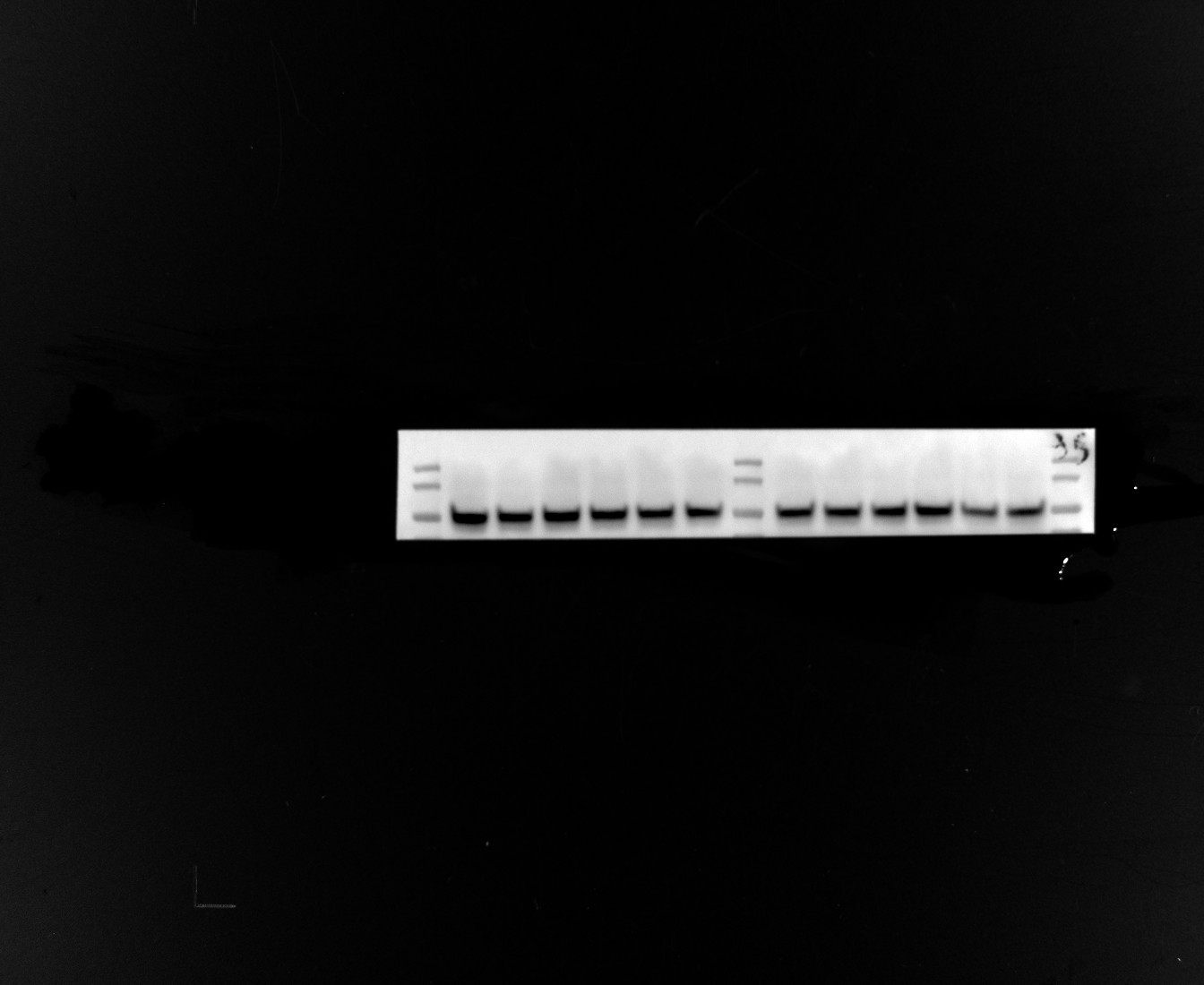

Supplement: Supplementary file 1 [file pharmaceuticals-18-01266-s001.zip › Western blot/STAT3/n3-n4 [Overlay][STAT3].tif]

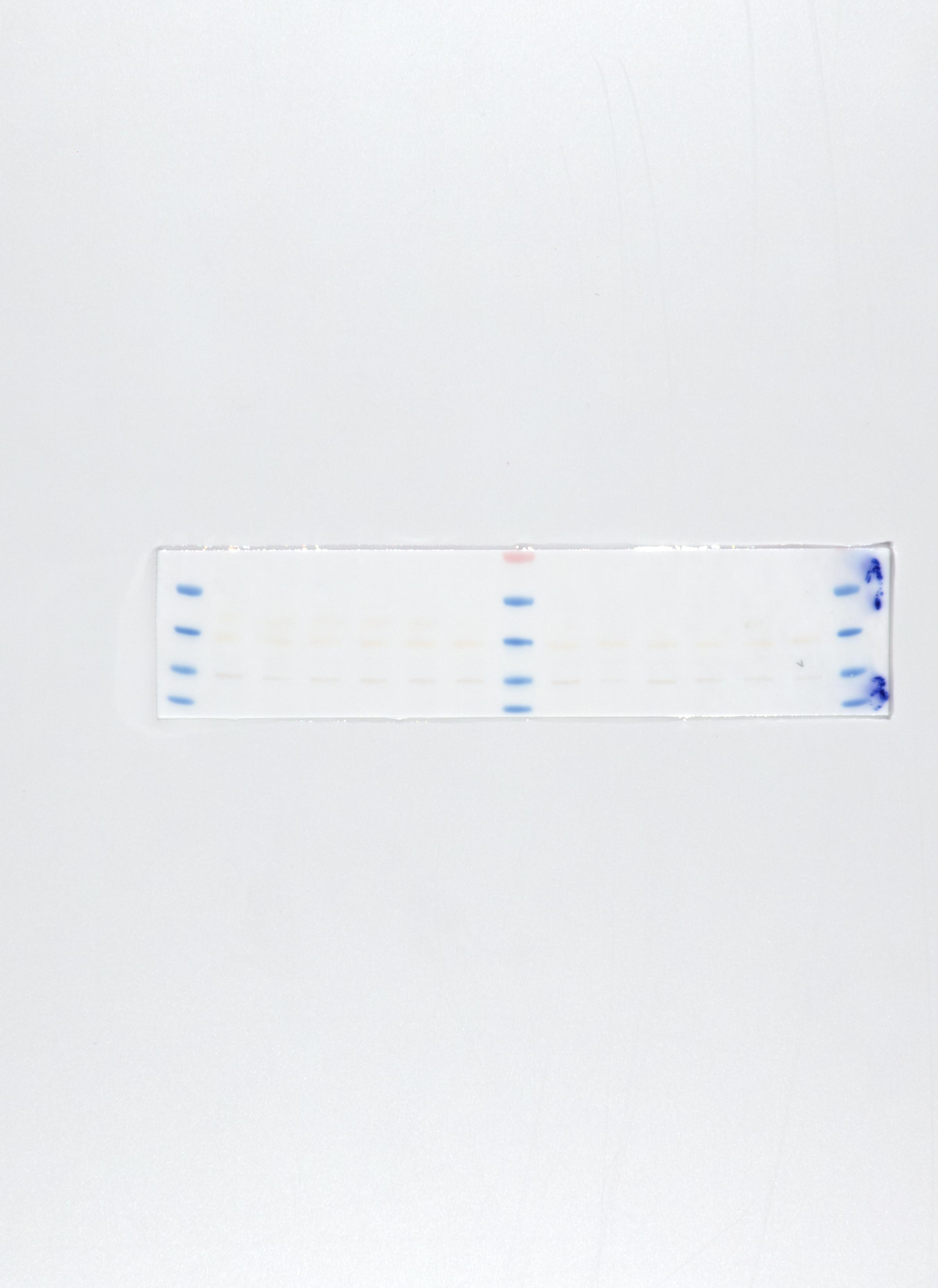

Supplement: Supplementary file 1 [file pharmaceuticals-18-01266-s001.zip › Western blot/STAT3/n5-n6 [Brightfield][GAPDH].jpg]

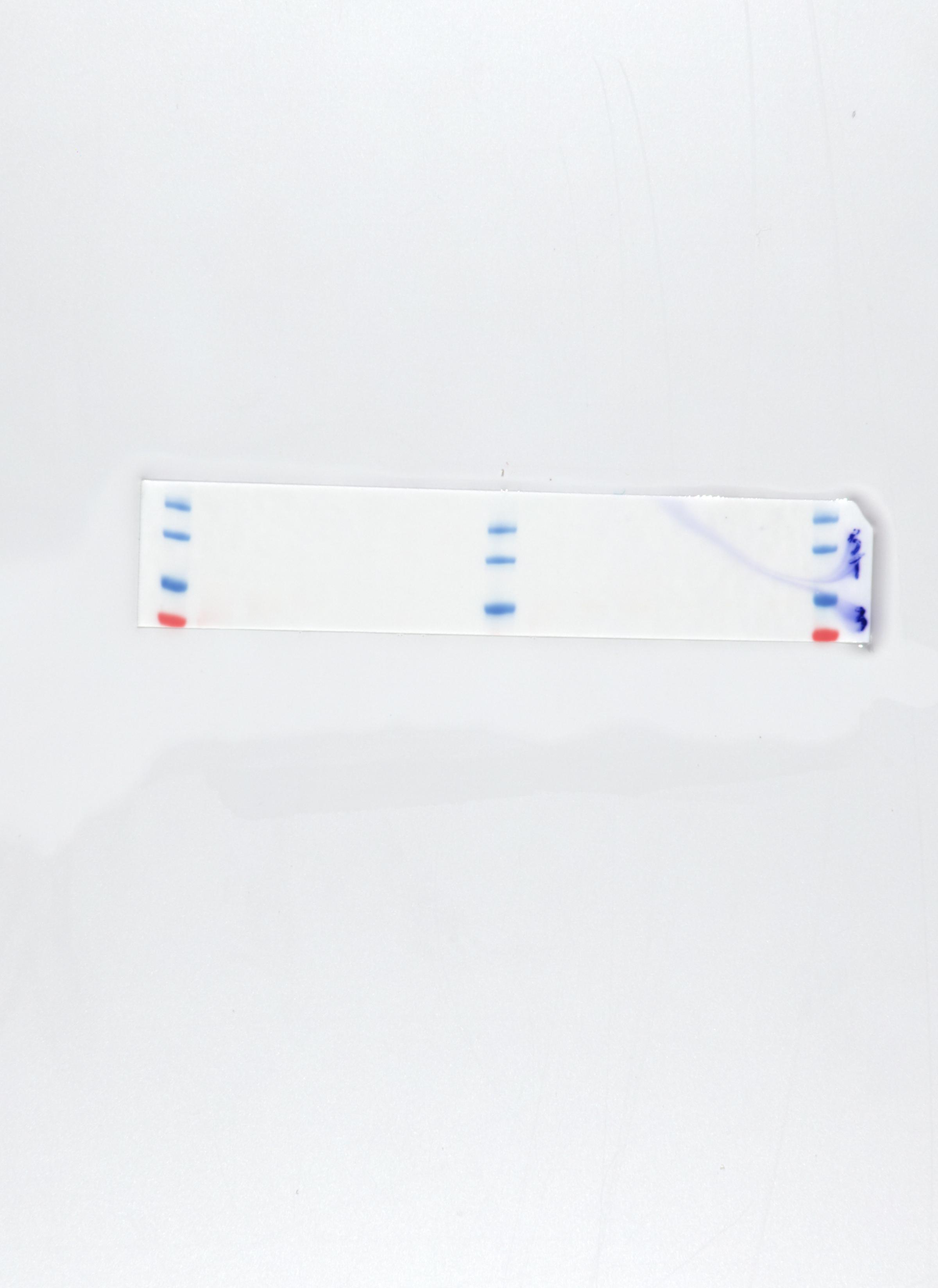

Supplement: Supplementary file 1 [file pharmaceuticals-18-01266-s001.zip › Western blot/STAT3/n5-n6 [Brightfield][STAT3].jpg]

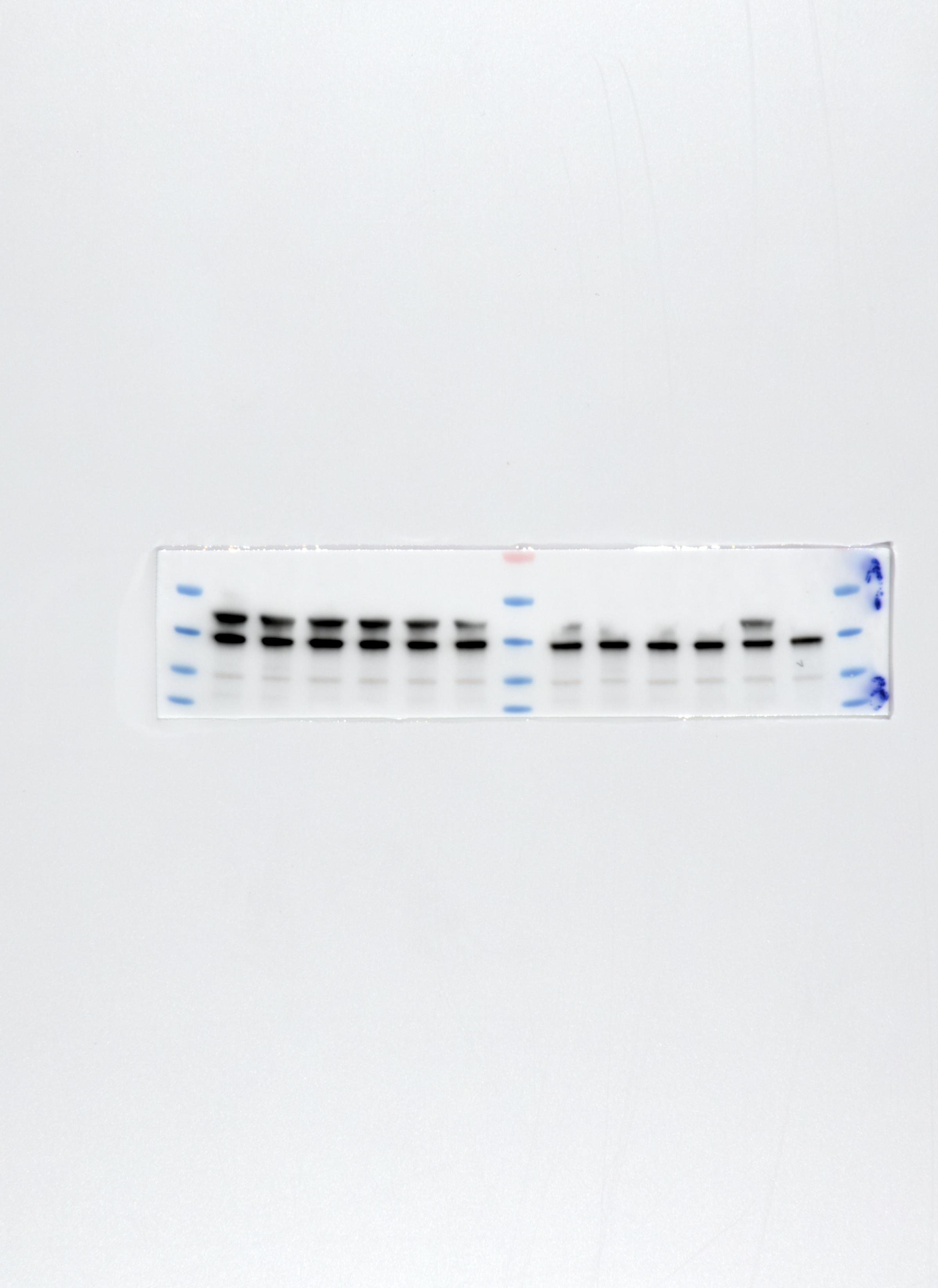

Supplement: Supplementary file 1 [file pharmaceuticals-18-01266-s001.zip › Western blot/STAT3/n5-n6 [Overlay][GAPDH].jpg]

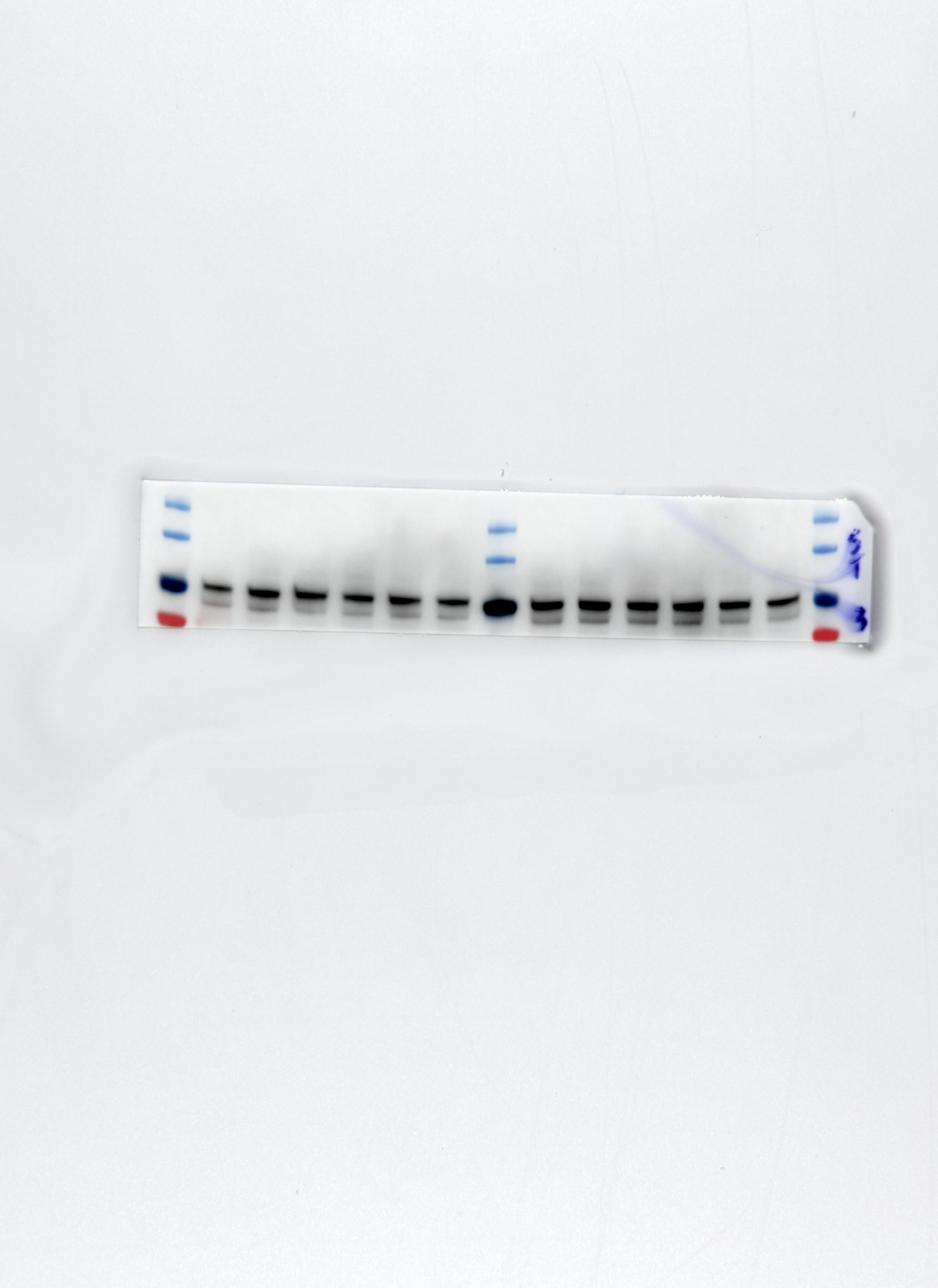

Supplement: Supplementary file 1 [file pharmaceuticals-18-01266-s001.zip › Western blot/STAT3/n5-n6 [Overlay][STAT3].jpg]
