# Supplementary figures and images for: Systems Network Integration of Transcriptomic, Proteomic, and Bioinformatic Analyses Reveals the Mechanism of XuanYunNing Tablets in Meniere’s Disease via JAK-STAT Pathway Modulation (part 1 of 2)
Source: Pharmaceuticals (Basel). 2025 Aug 25;18(9):1266. doi: 10.3390/ph18091266 (PMC12472466; doi:10.3390/ph18091266)

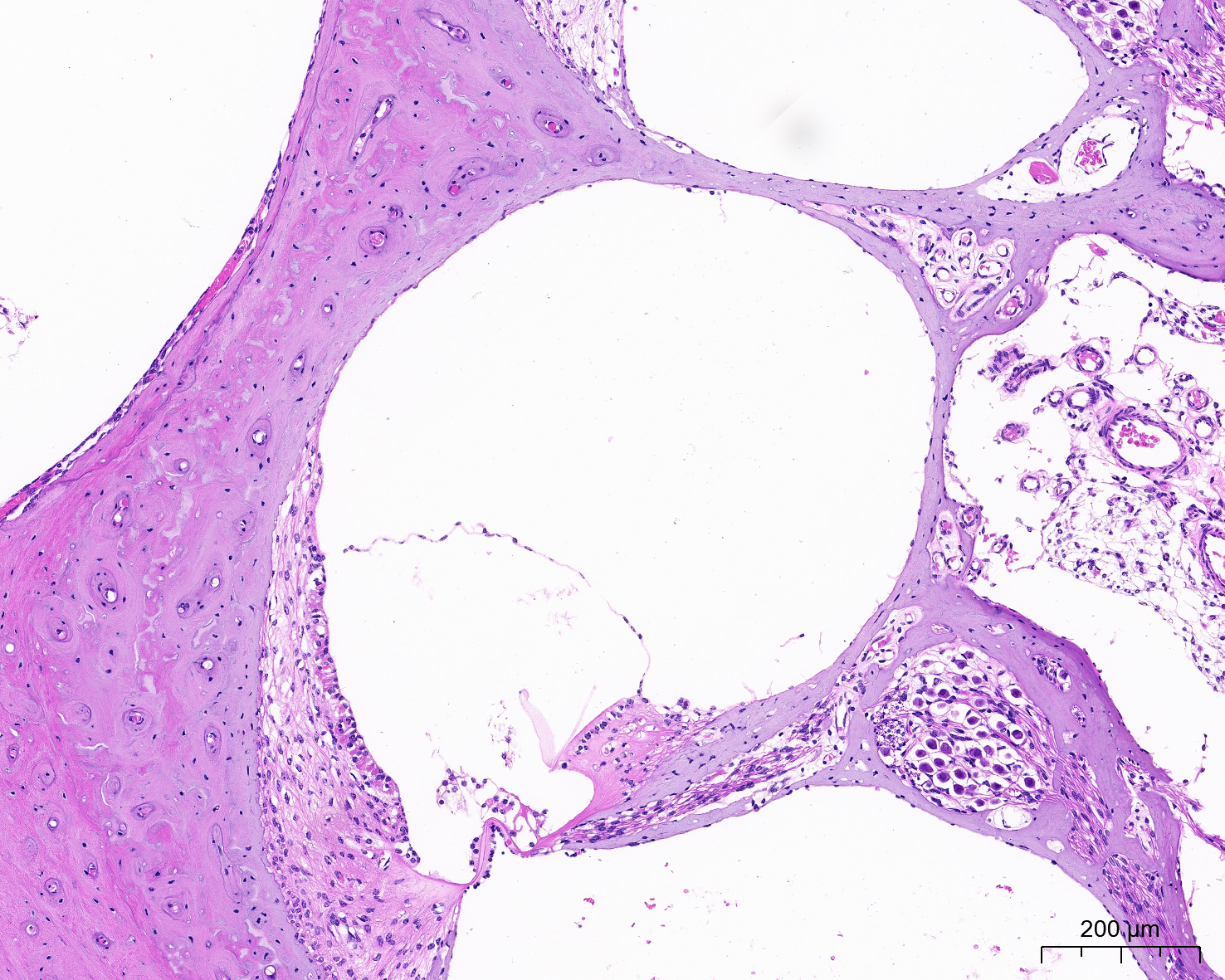

Supplement: Supplementary file 1 [file pharmaceuticals-18-01266-s001.zip › Cochlear H&E Staining/BHS/1.tif]

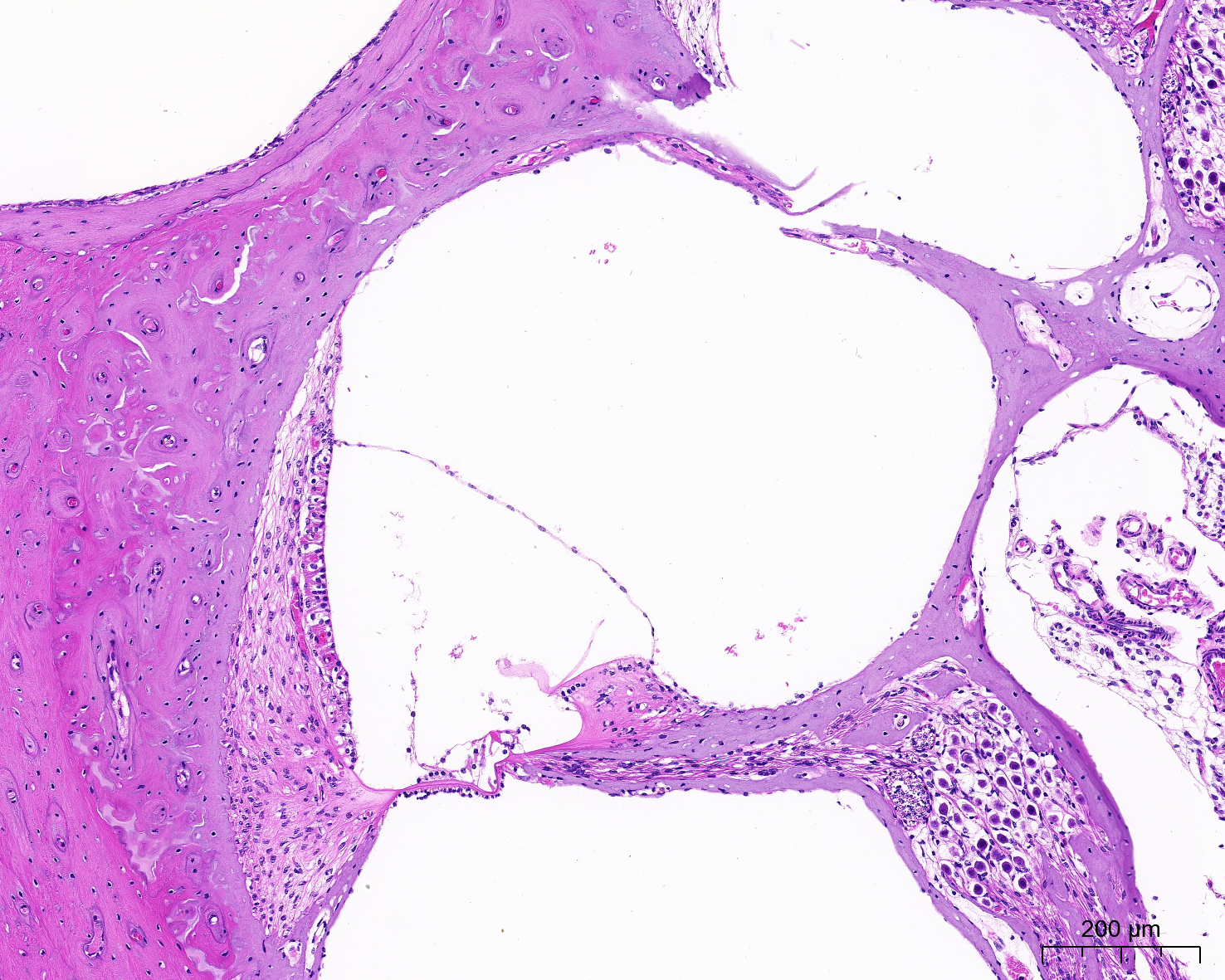

Supplement: Supplementary file 1 [file pharmaceuticals-18-01266-s001.zip › Cochlear H&E Staining/BHS/2.tif]

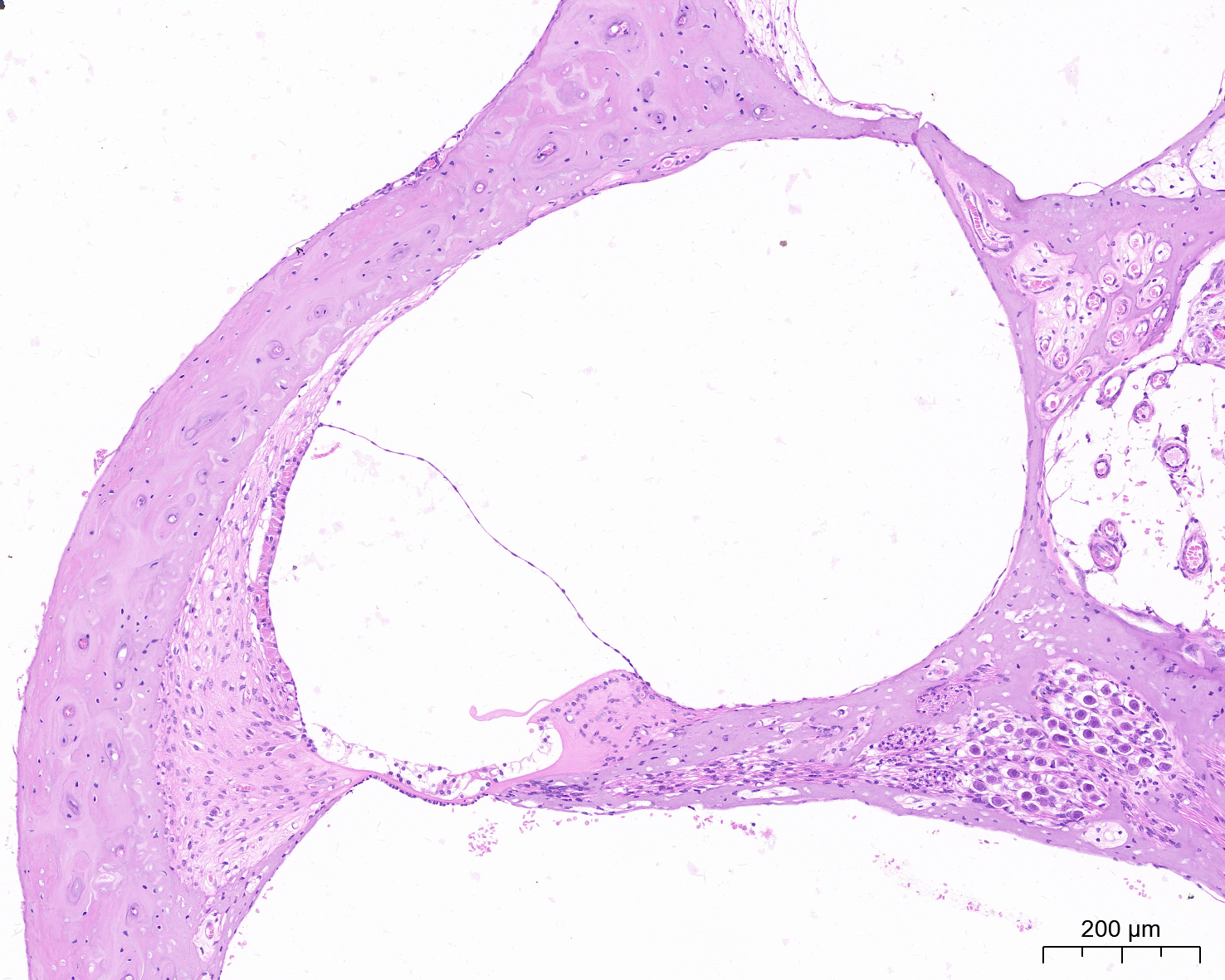

Supplement: Supplementary file 1 [file pharmaceuticals-18-01266-s001.zip › Cochlear H&E Staining/BHS/3.tif]

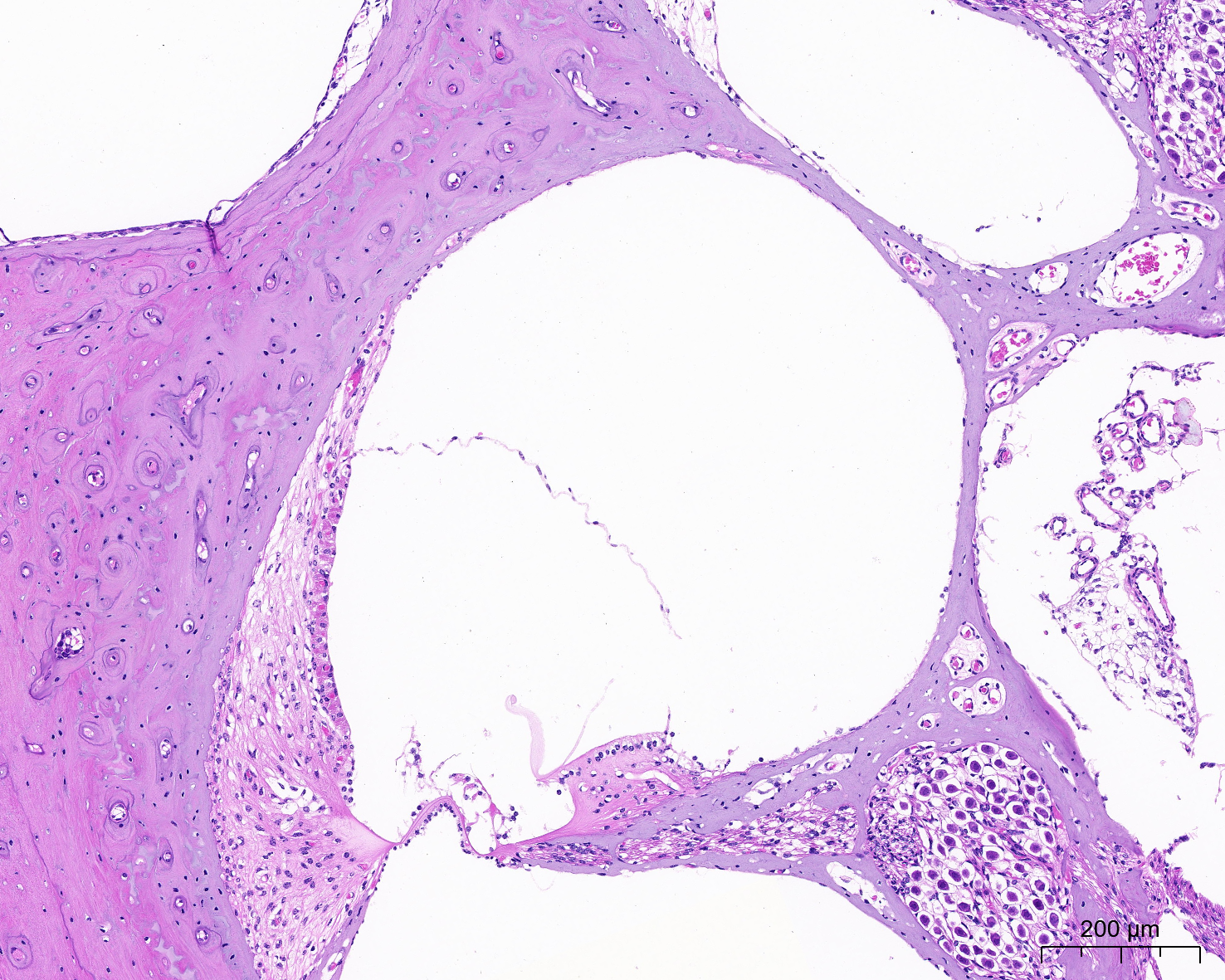

Supplement: Supplementary file 1 [file pharmaceuticals-18-01266-s001.zip › Cochlear H&E Staining/MD/1.tif]

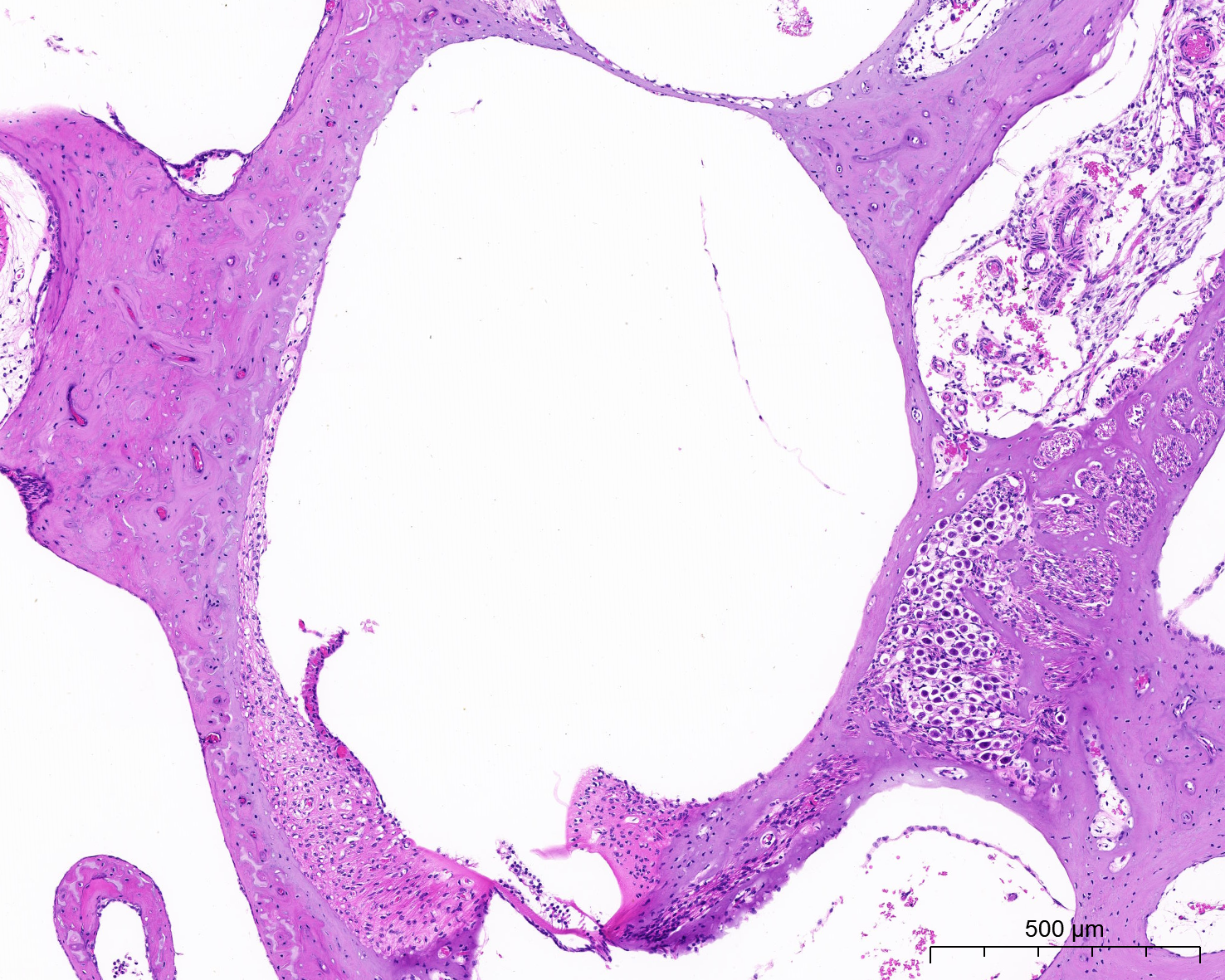

Supplement: Supplementary file 1 [file pharmaceuticals-18-01266-s001.zip › Cochlear H&E Staining/MD/2.tif]

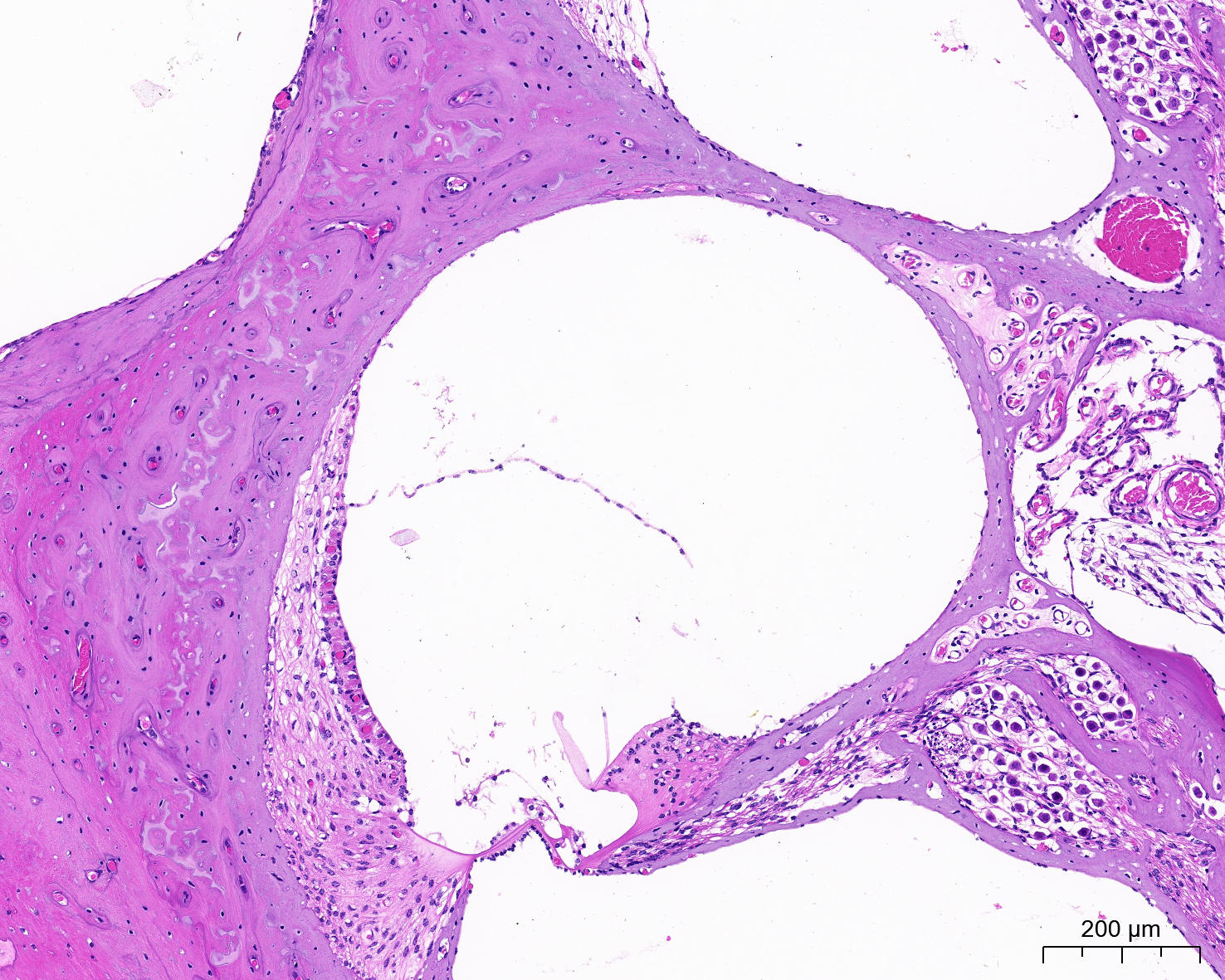

Supplement: Supplementary file 1 [file pharmaceuticals-18-01266-s001.zip › Cochlear H&E Staining/MD/3.tif]

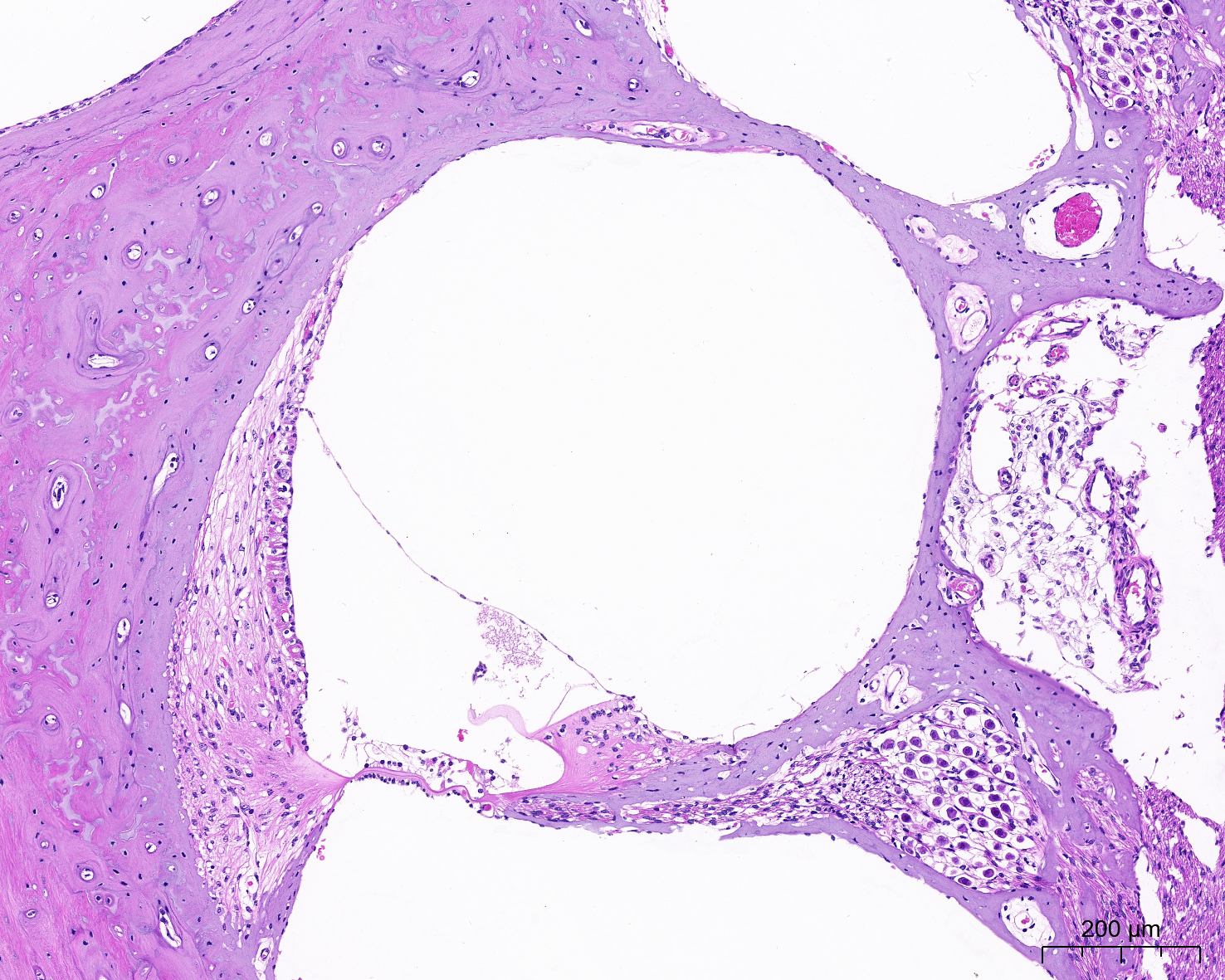

Supplement: Supplementary file 1 [file pharmaceuticals-18-01266-s001.zip › Cochlear H&E Staining/NC/1.tif]

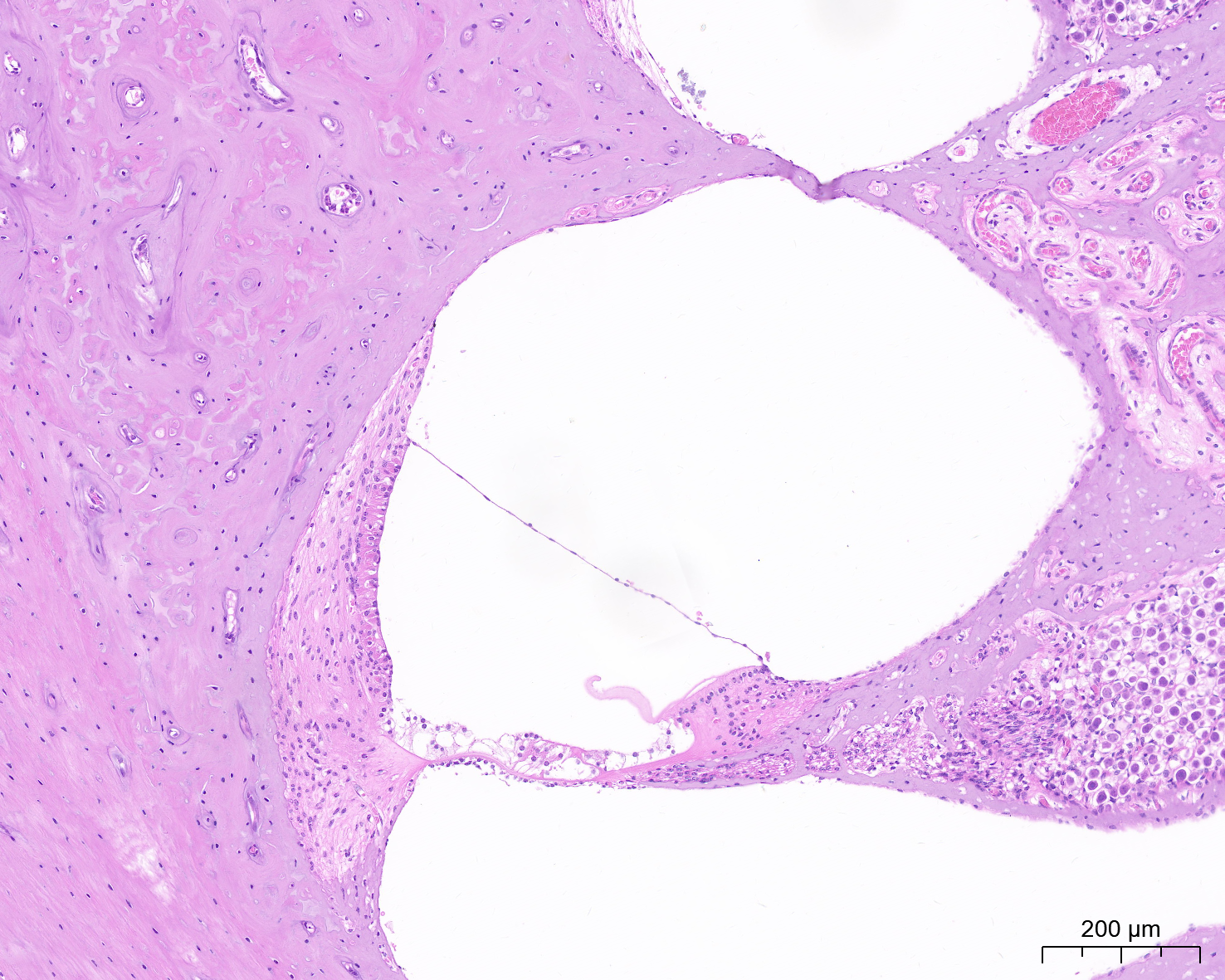

Supplement: Supplementary file 1 [file pharmaceuticals-18-01266-s001.zip › Cochlear H&E Staining/NC/2.tif]

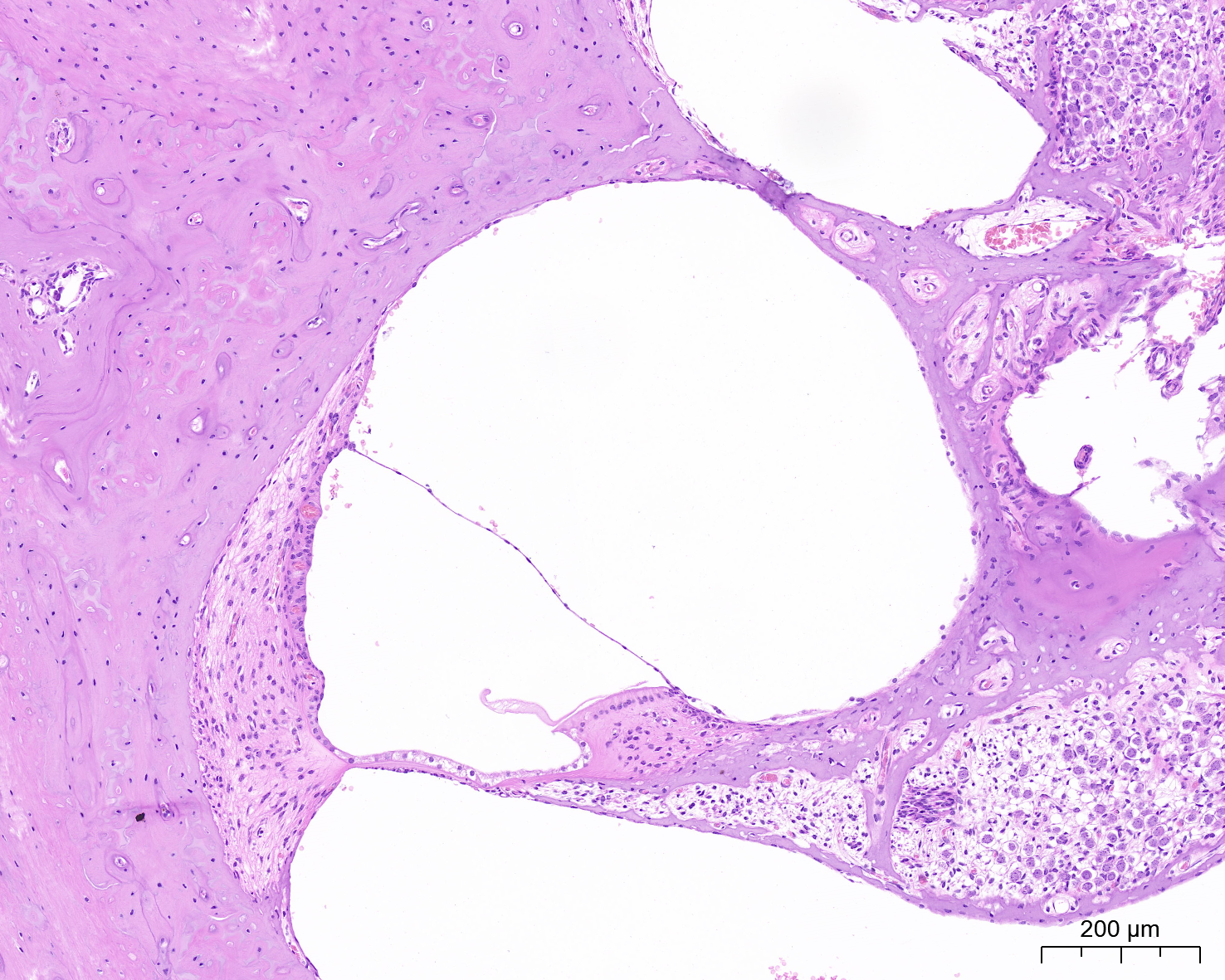

Supplement: Supplementary file 1 [file pharmaceuticals-18-01266-s001.zip › Cochlear H&E Staining/NC/3.tif]

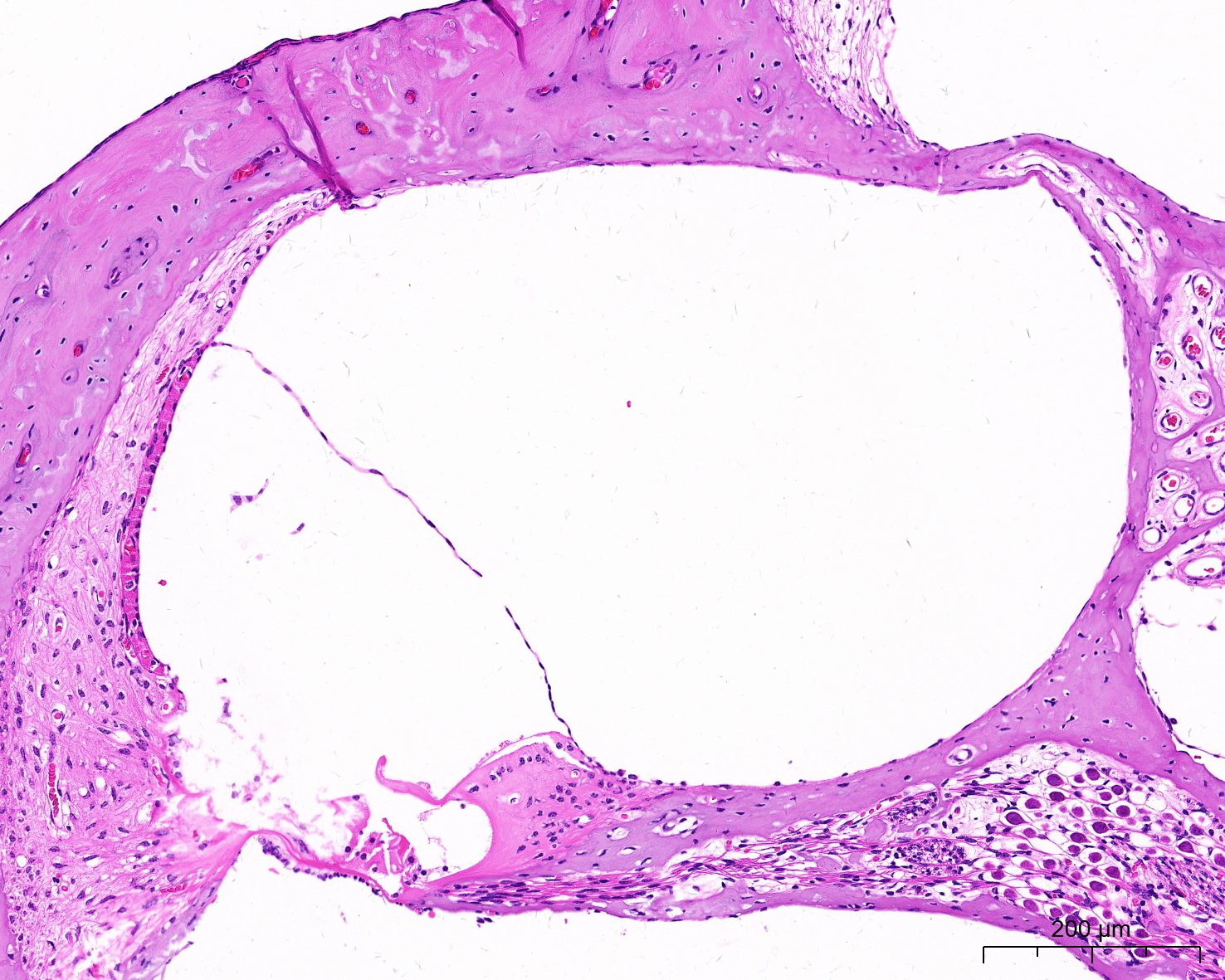

Supplement: Supplementary file 1 [file pharmaceuticals-18-01266-s001.zip › Cochlear H&E Staining/XYN 0.9/1.tif]

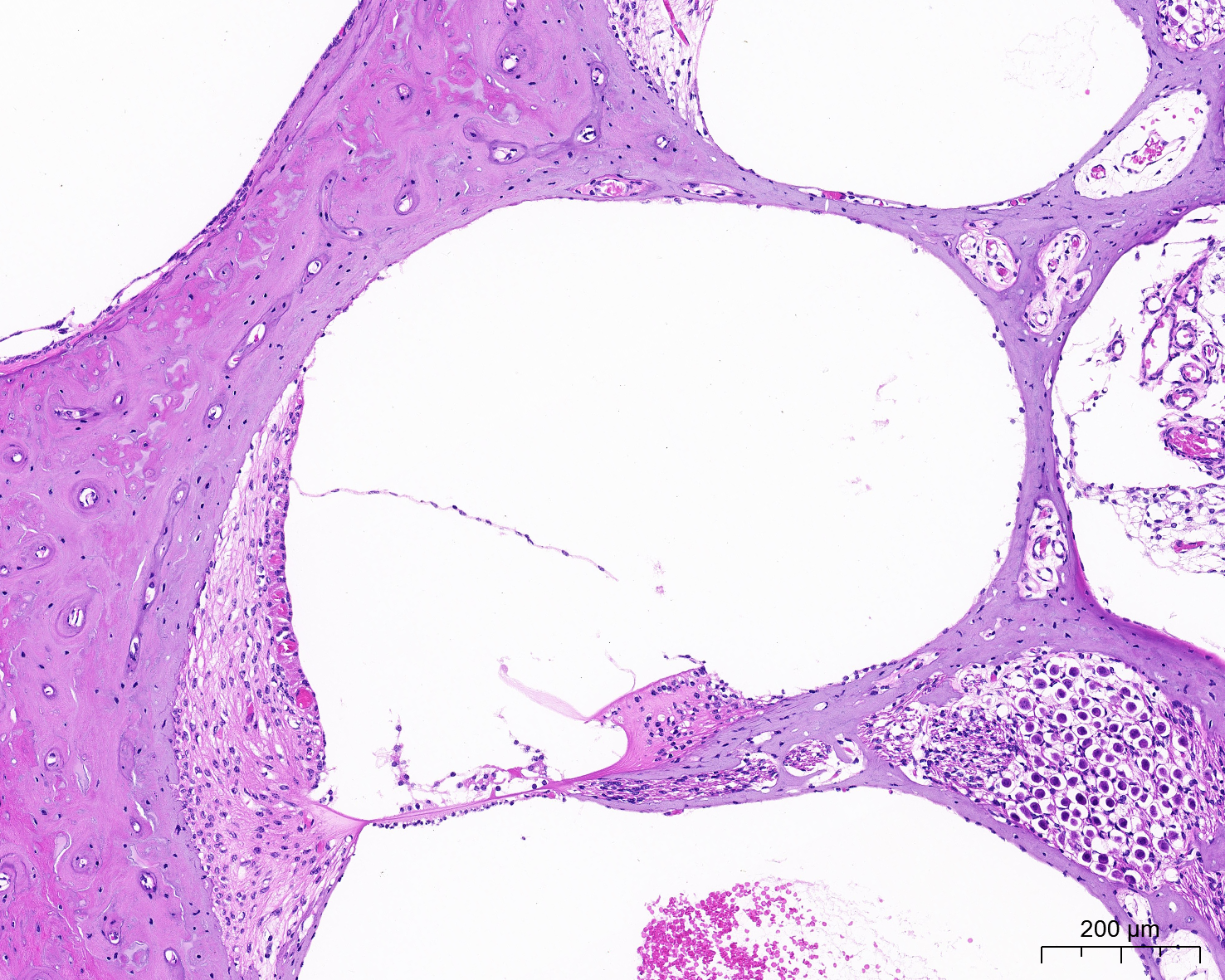

Supplement: Supplementary file 1 [file pharmaceuticals-18-01266-s001.zip › Cochlear H&E Staining/XYN 0.9/2.tif]

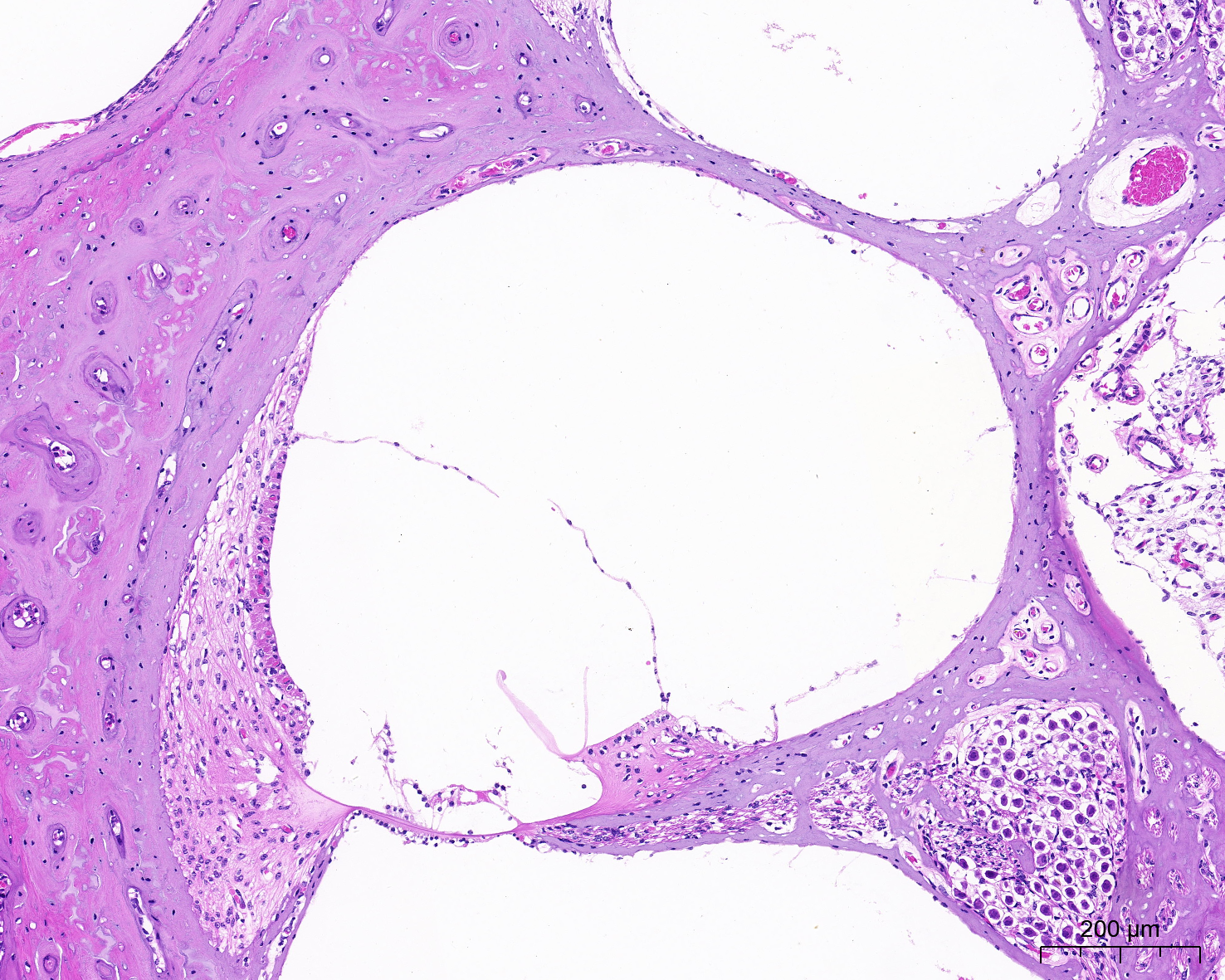

Supplement: Supplementary file 1 [file pharmaceuticals-18-01266-s001.zip › Cochlear H&E Staining/XYN 0.9/3.tif]

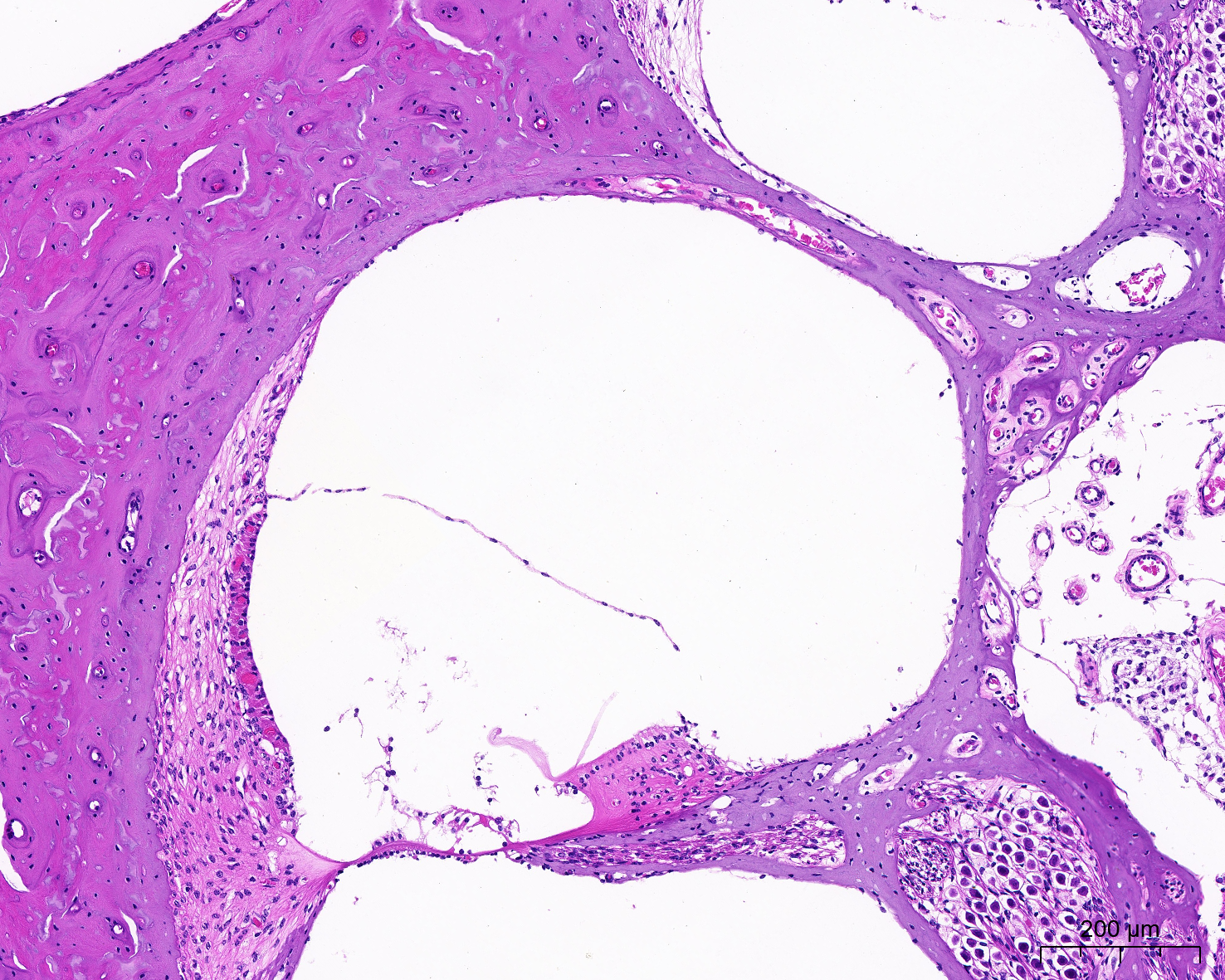

Supplement: Supplementary file 1 [file pharmaceuticals-18-01266-s001.zip › Cochlear H&E Staining/XYN 1.8/1.tif]

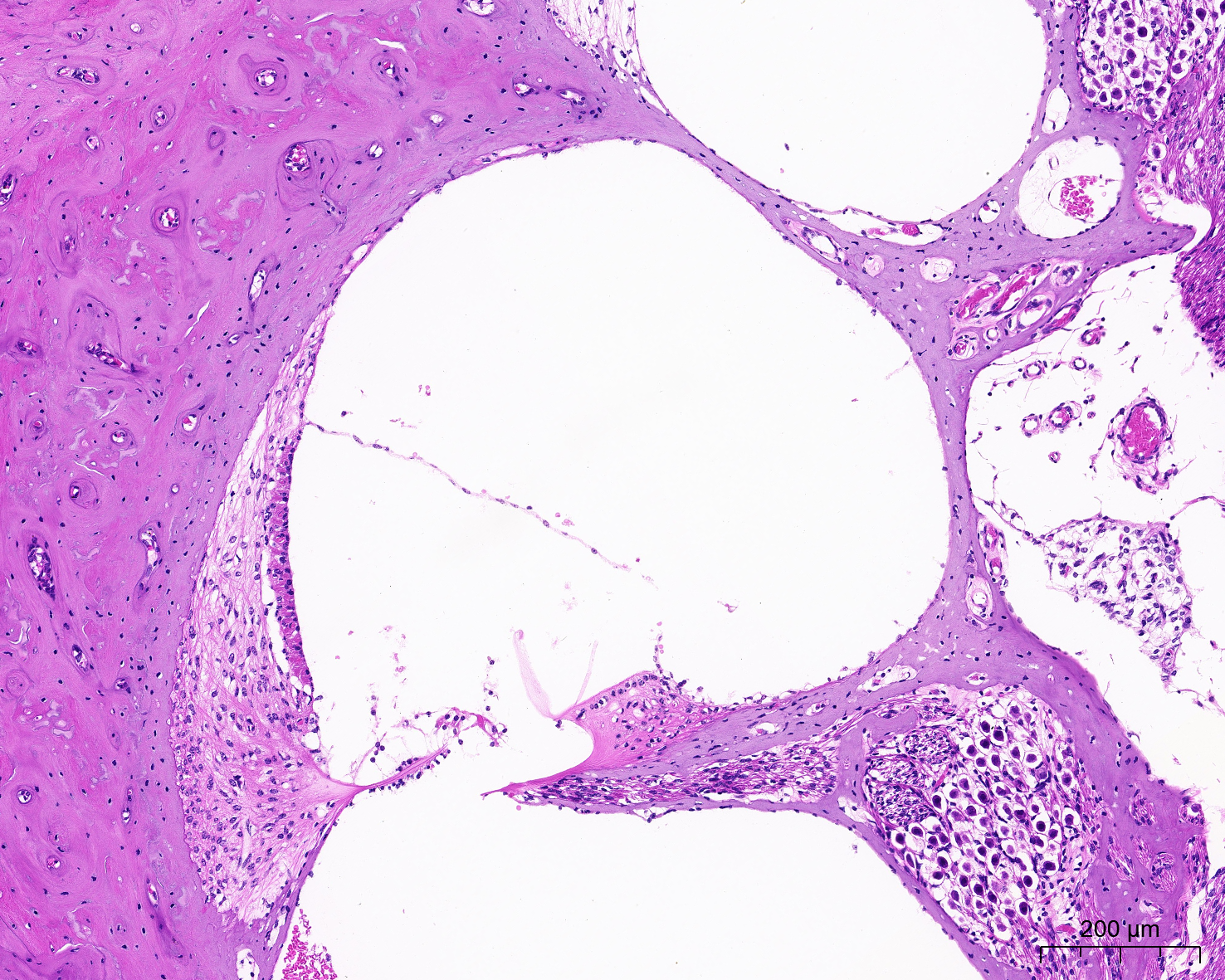

Supplement: Supplementary file 1 [file pharmaceuticals-18-01266-s001.zip › Cochlear H&E Staining/XYN 1.8/2.tif]

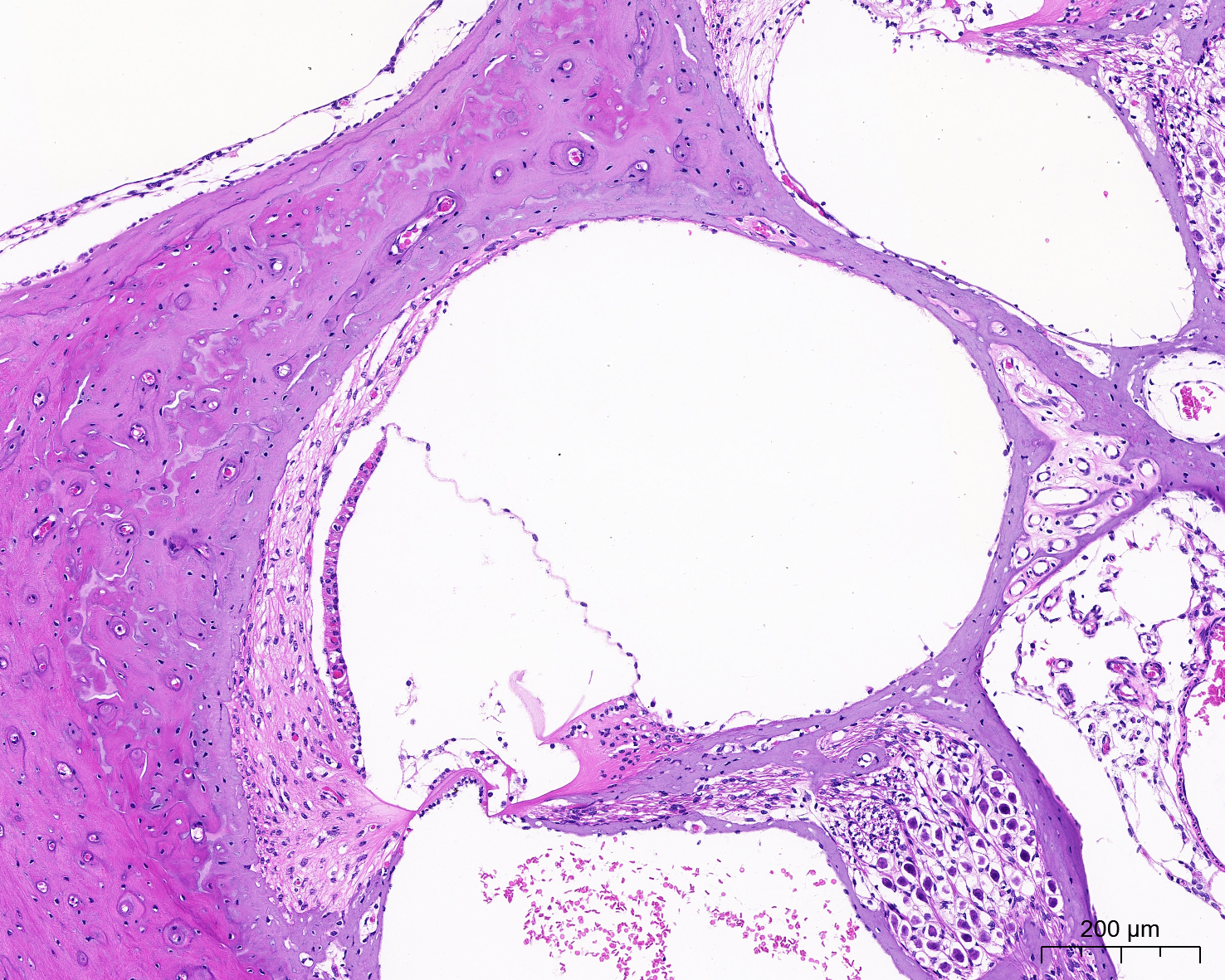

Supplement: Supplementary file 1 [file pharmaceuticals-18-01266-s001.zip › Cochlear H&E Staining/XYN 1.8/3.tif]

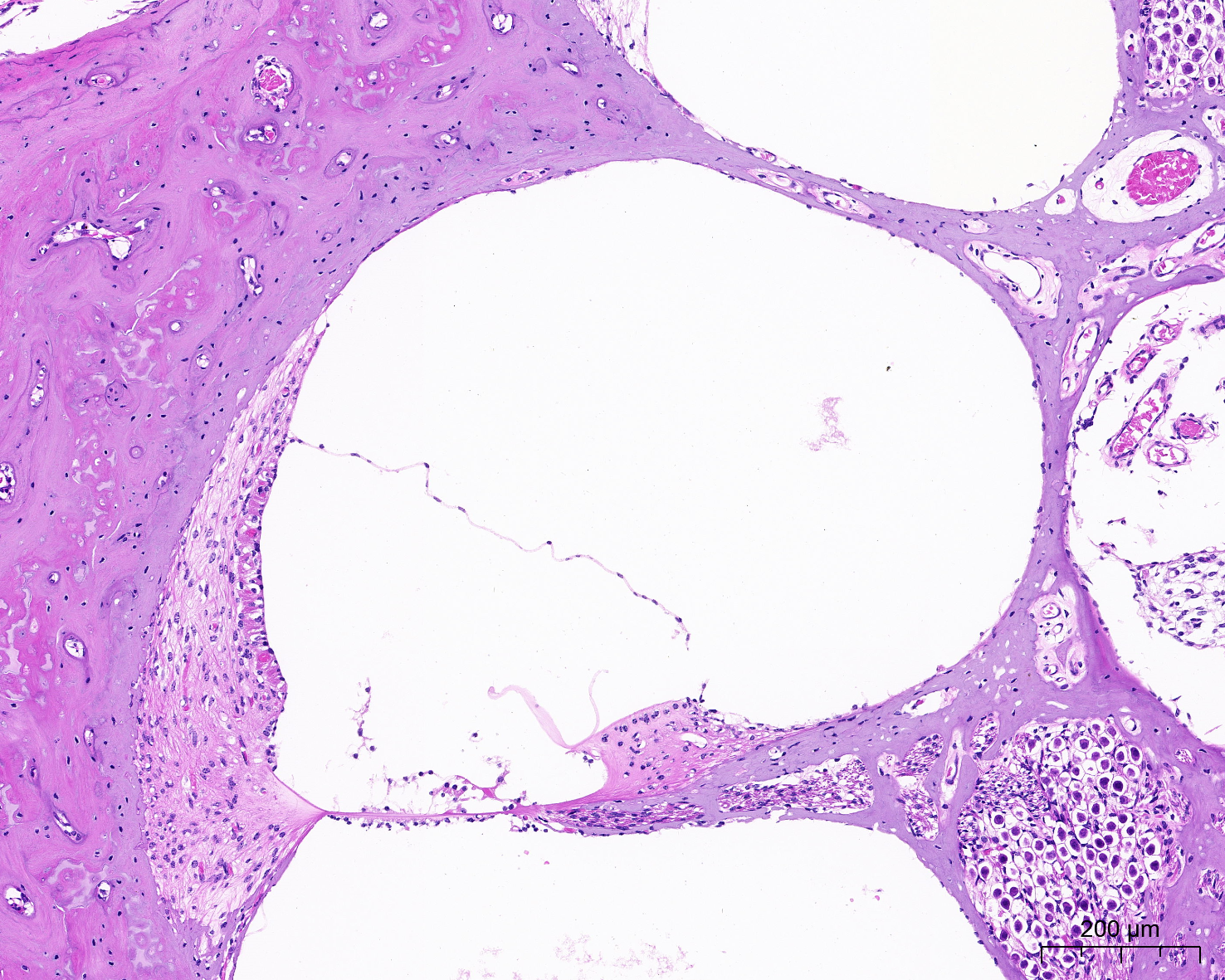

Supplement: Supplementary file 1 [file pharmaceuticals-18-01266-s001.zip › Cochlear H&E Staining/XYN 3.6/1.tif]

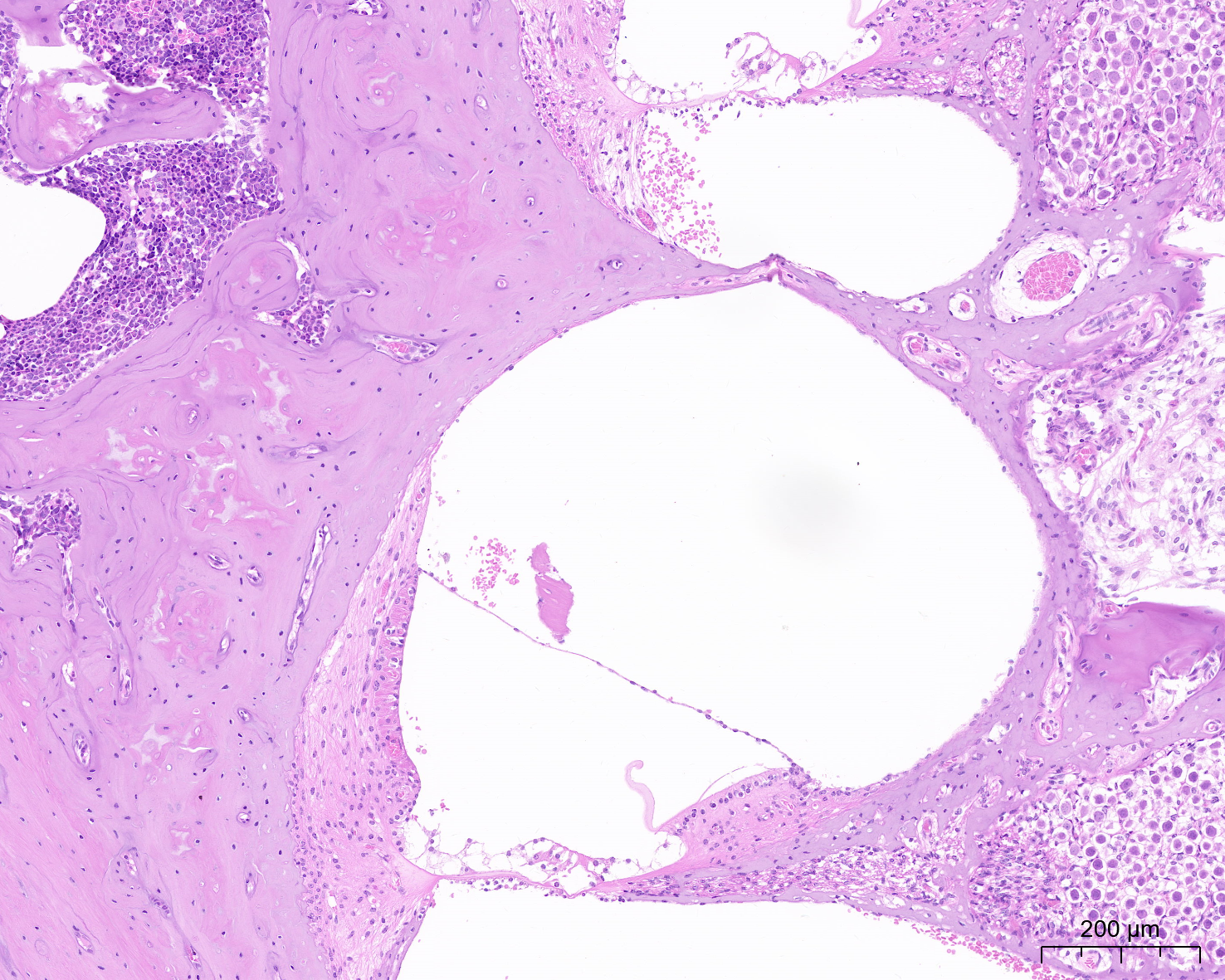

Supplement: Supplementary file 1 [file pharmaceuticals-18-01266-s001.zip › Cochlear H&E Staining/XYN 3.6/2.tif]

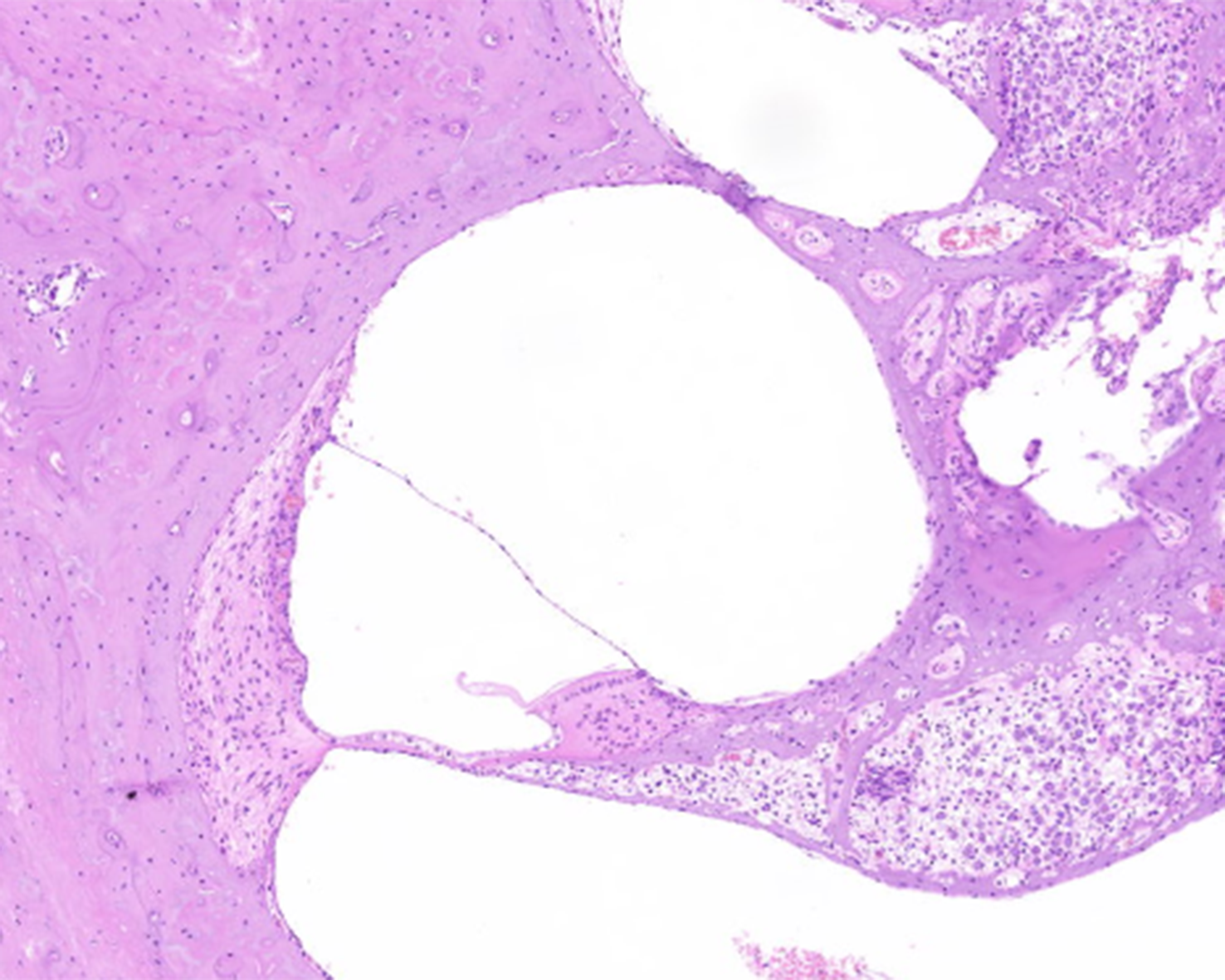

Supplement: Supplementary file 1 [file pharmaceuticals-18-01266-s001.zip › Cochlear H&E Staining/XYN 3.6/3.tif]

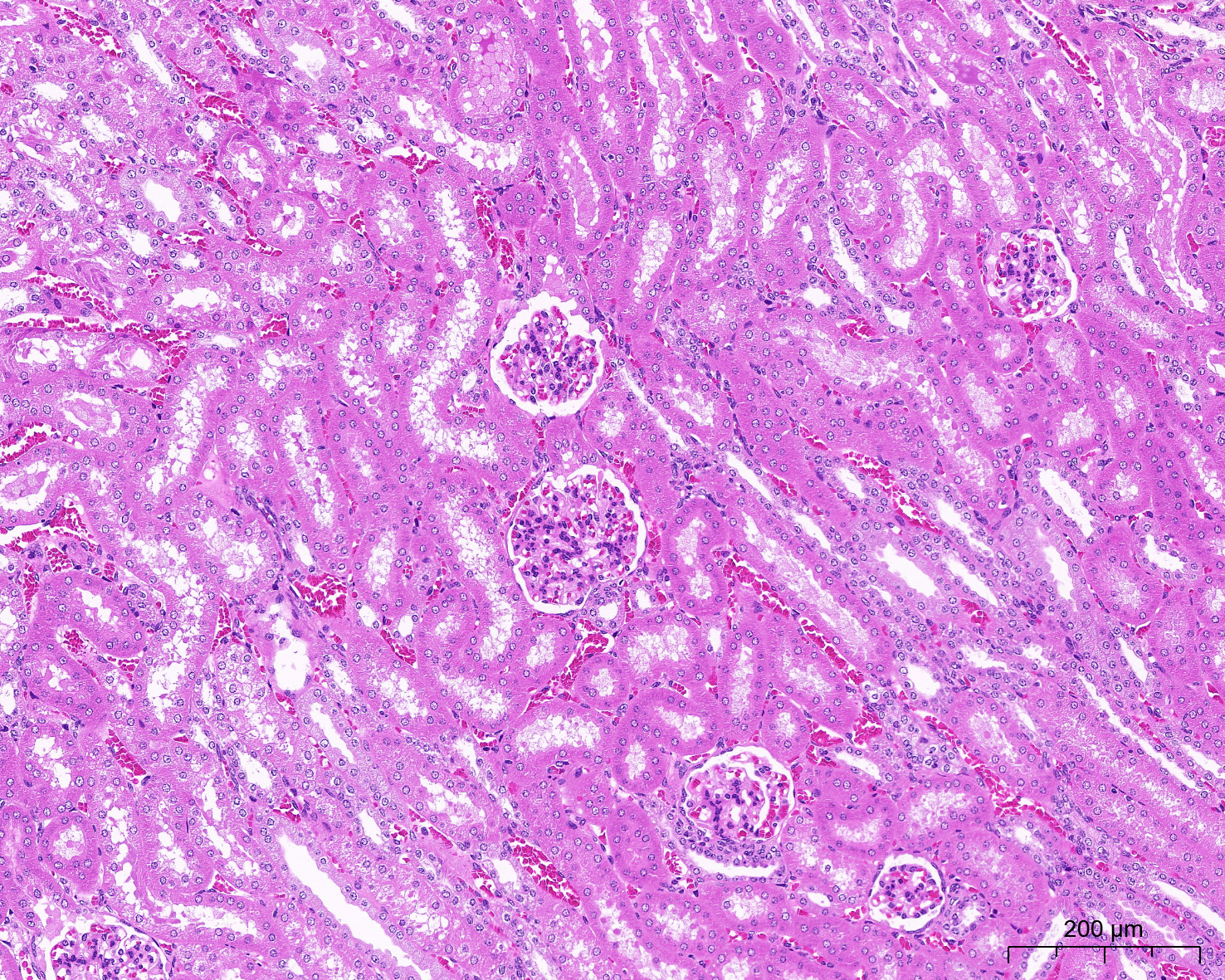

Supplement: Supplementary file 1 [file pharmaceuticals-18-01266-s001.zip › Renal H&E Staining/BHS/1.tif]

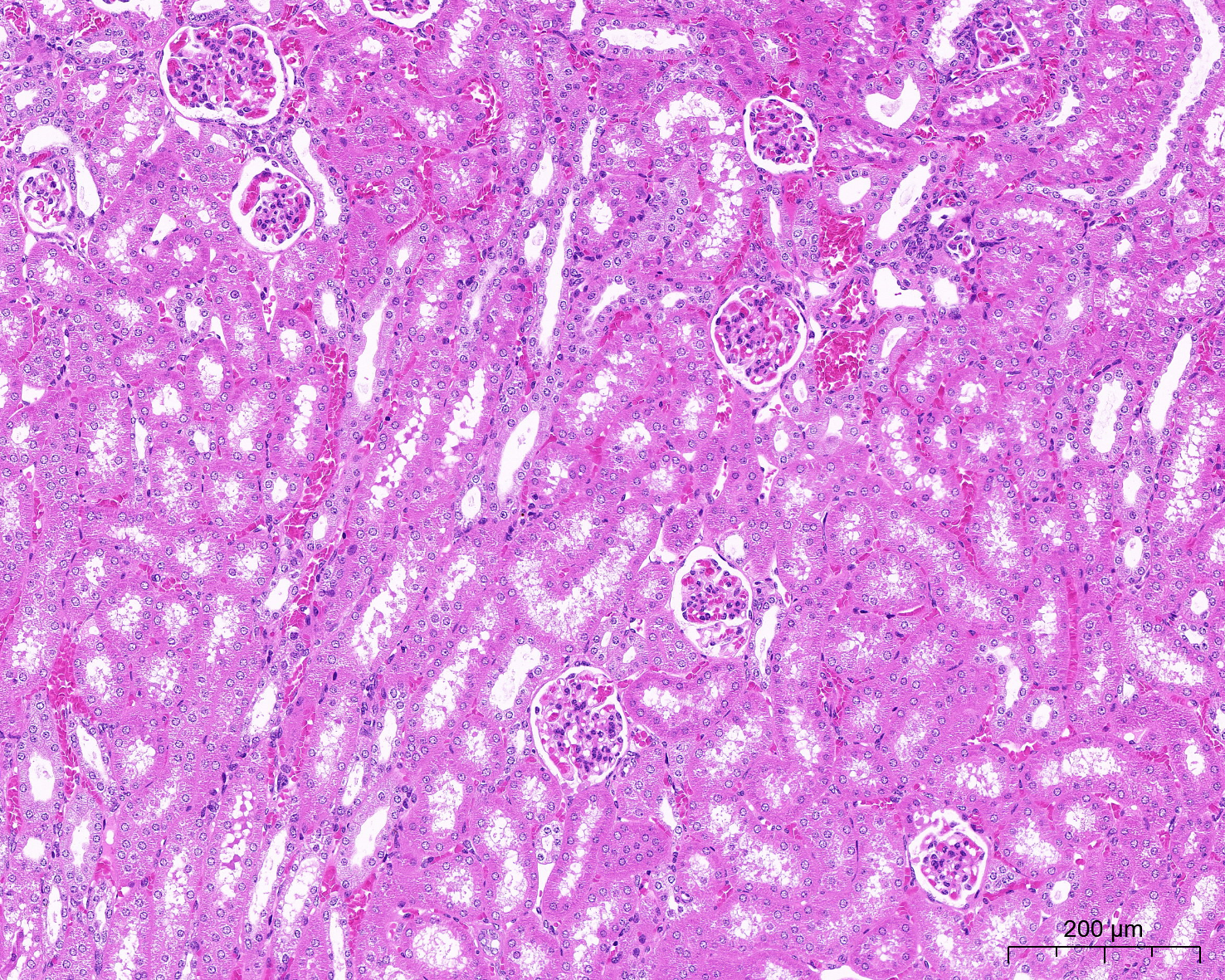

Supplement: Supplementary file 1 [file pharmaceuticals-18-01266-s001.zip › Renal H&E Staining/BHS/2.tif]

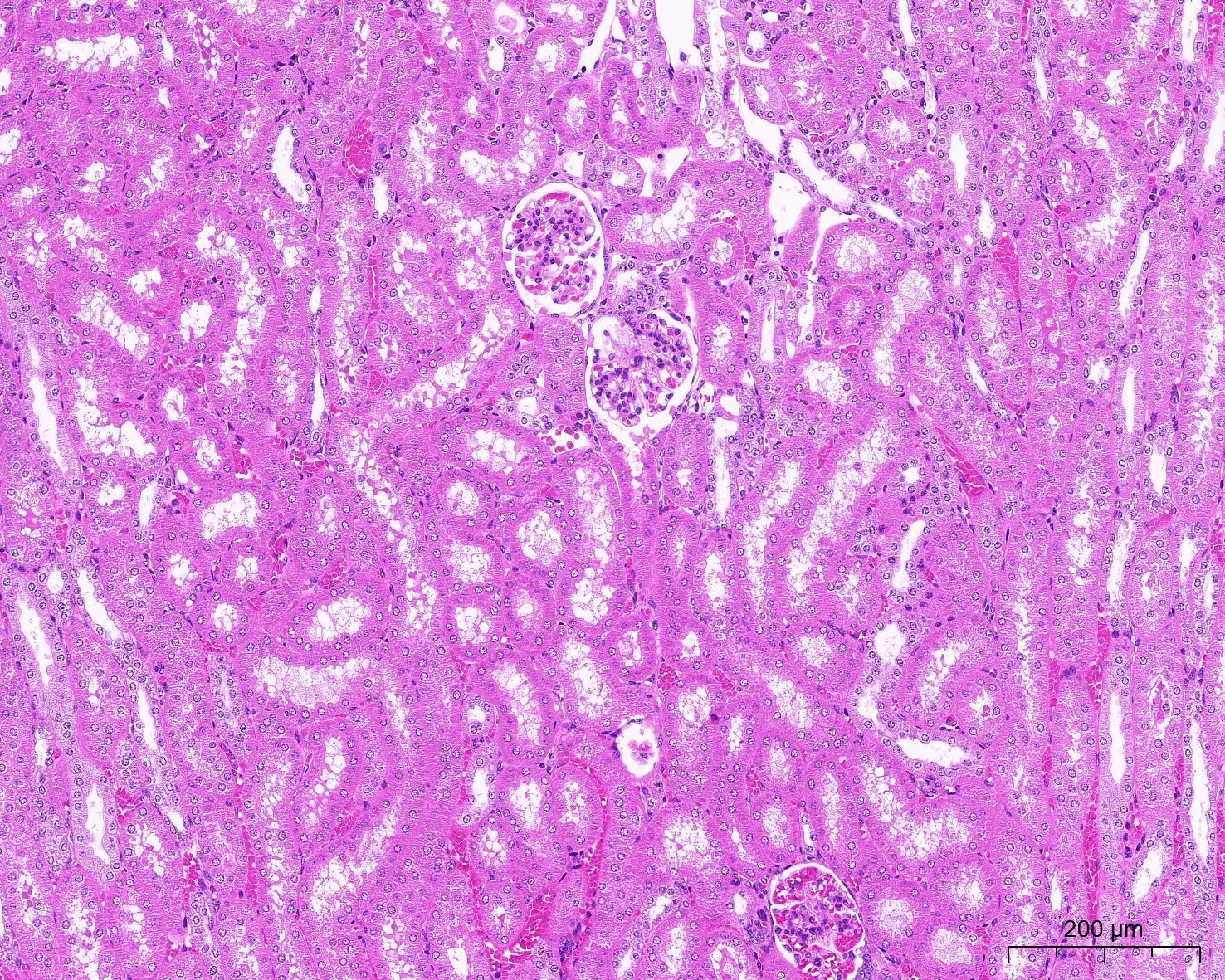

Supplement: Supplementary file 1 [file pharmaceuticals-18-01266-s001.zip › Renal H&E Staining/BHS/3.tif]

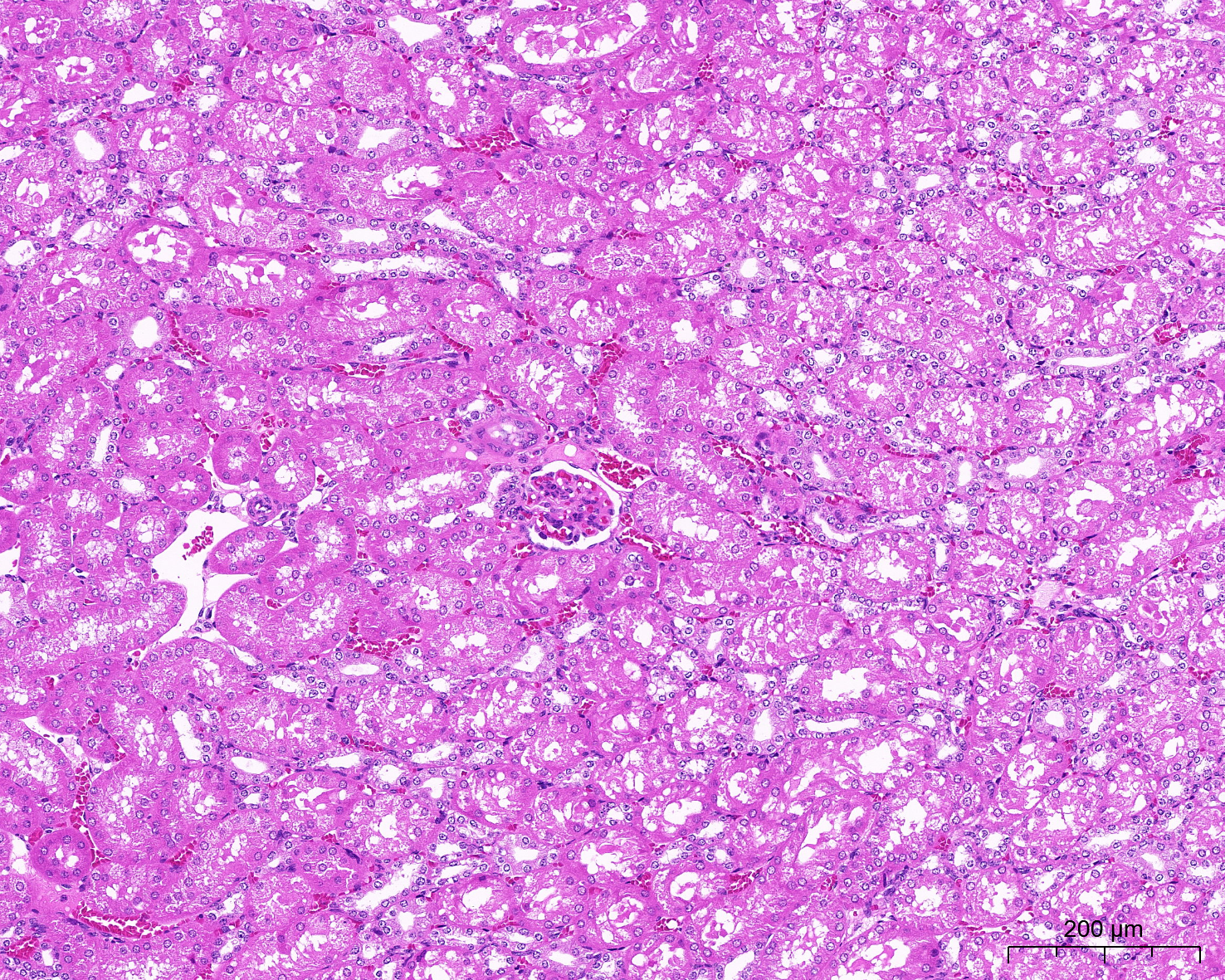

Supplement: Supplementary file 1 [file pharmaceuticals-18-01266-s001.zip › Renal H&E Staining/MD/1.tif]

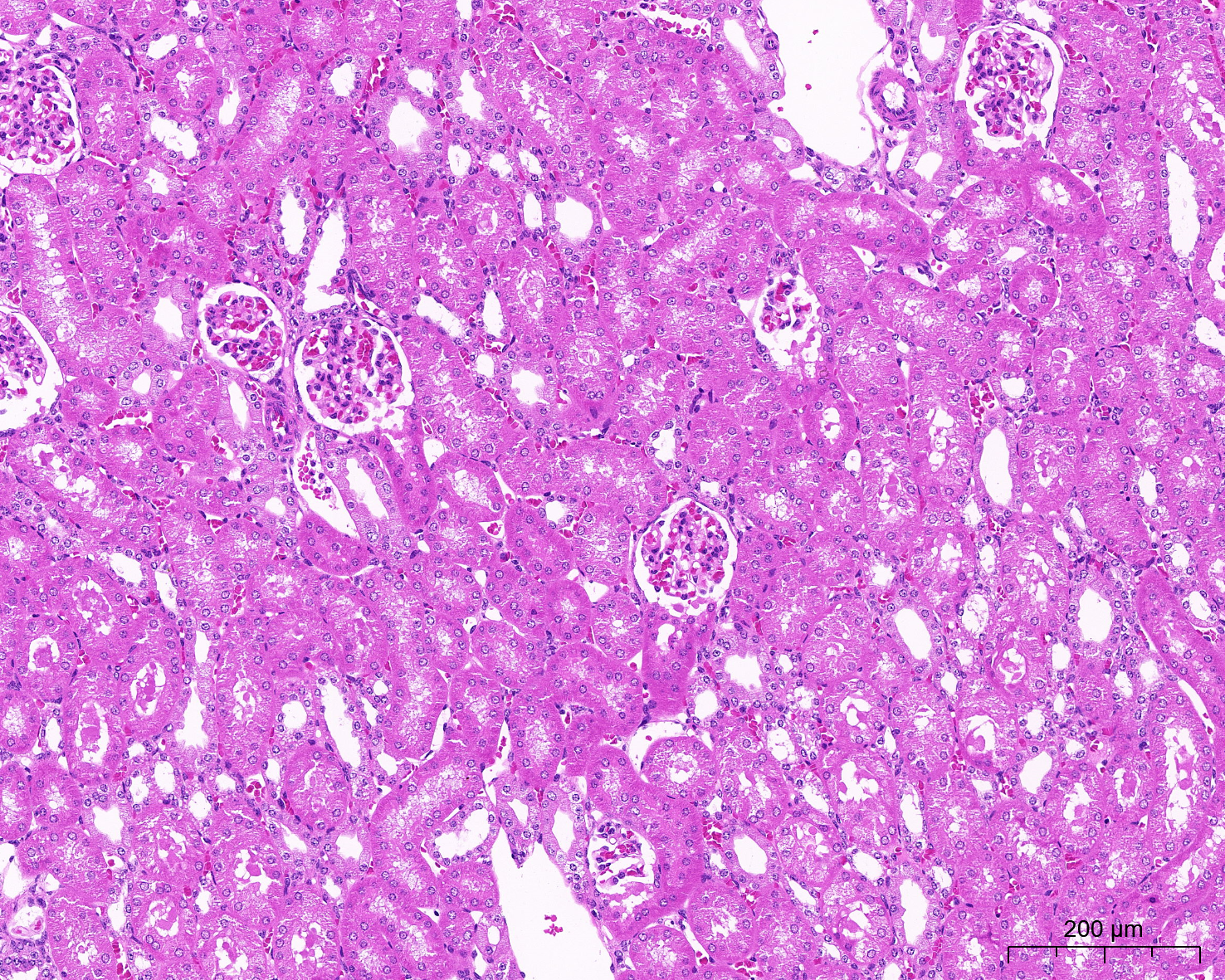

Supplement: Supplementary file 1 [file pharmaceuticals-18-01266-s001.zip › Renal H&E Staining/MD/2.tif]

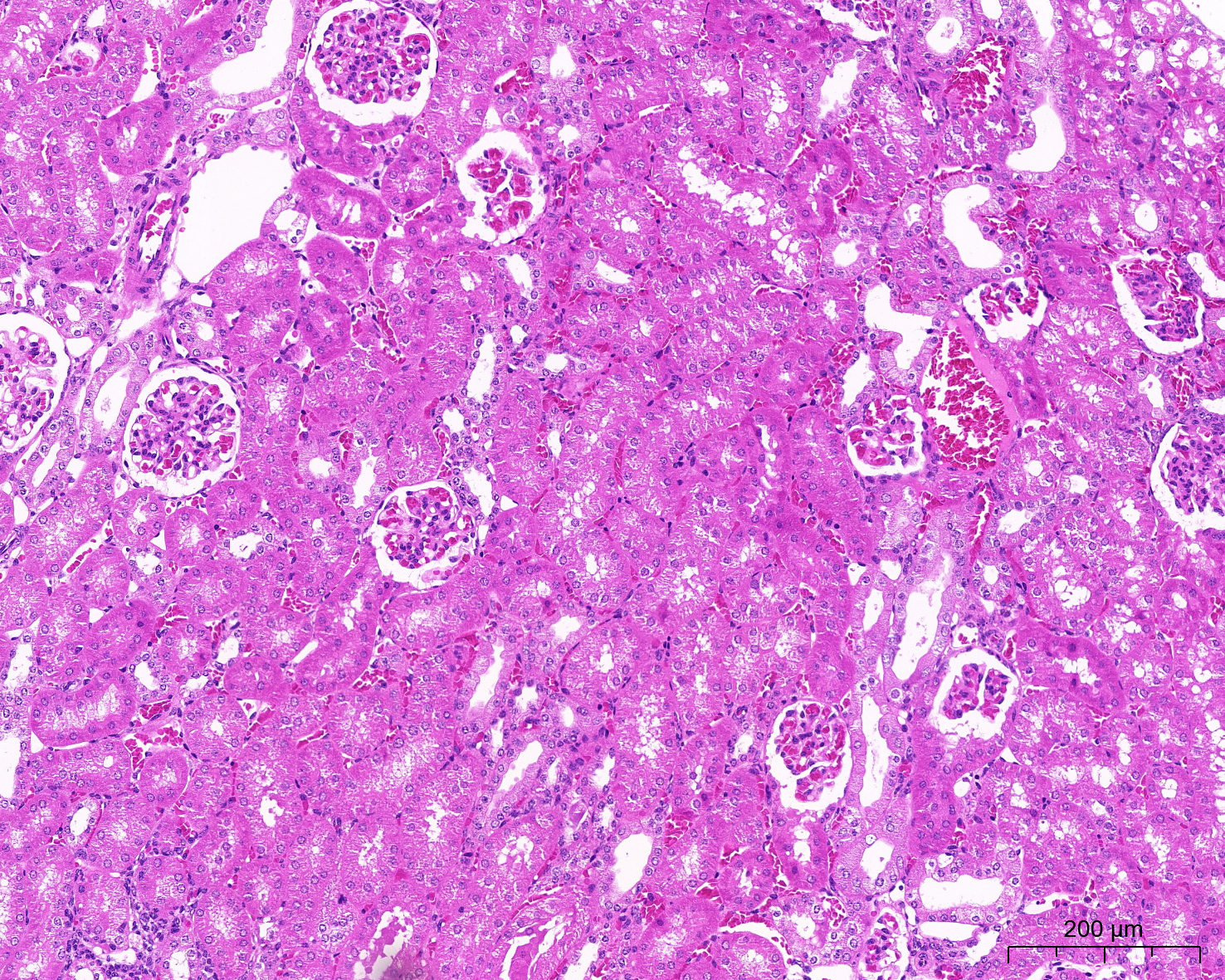

Supplement: Supplementary file 1 [file pharmaceuticals-18-01266-s001.zip › Renal H&E Staining/MD/3.tif]

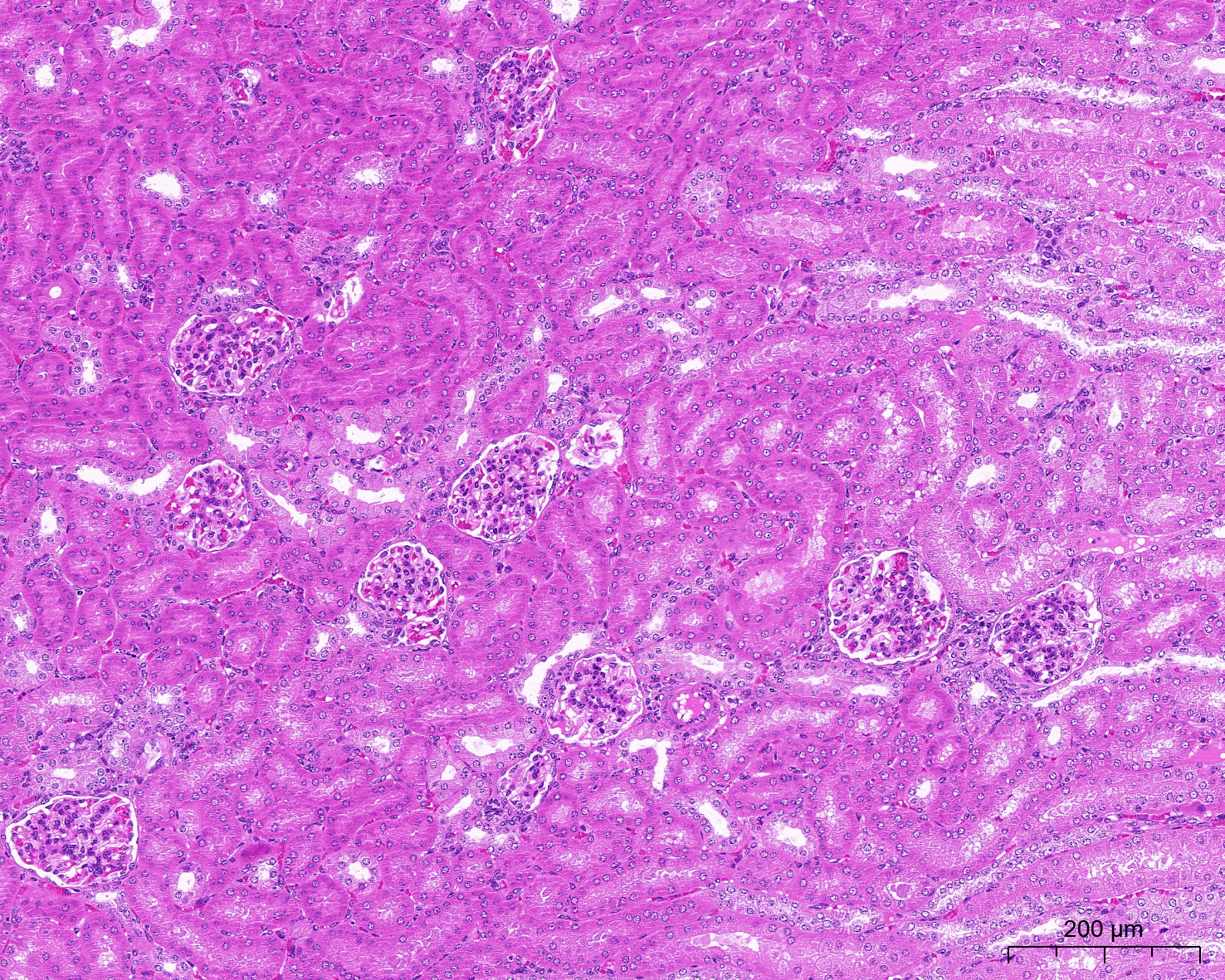

Supplement: Supplementary file 1 [file pharmaceuticals-18-01266-s001.zip › Renal H&E Staining/NC/1.tif]

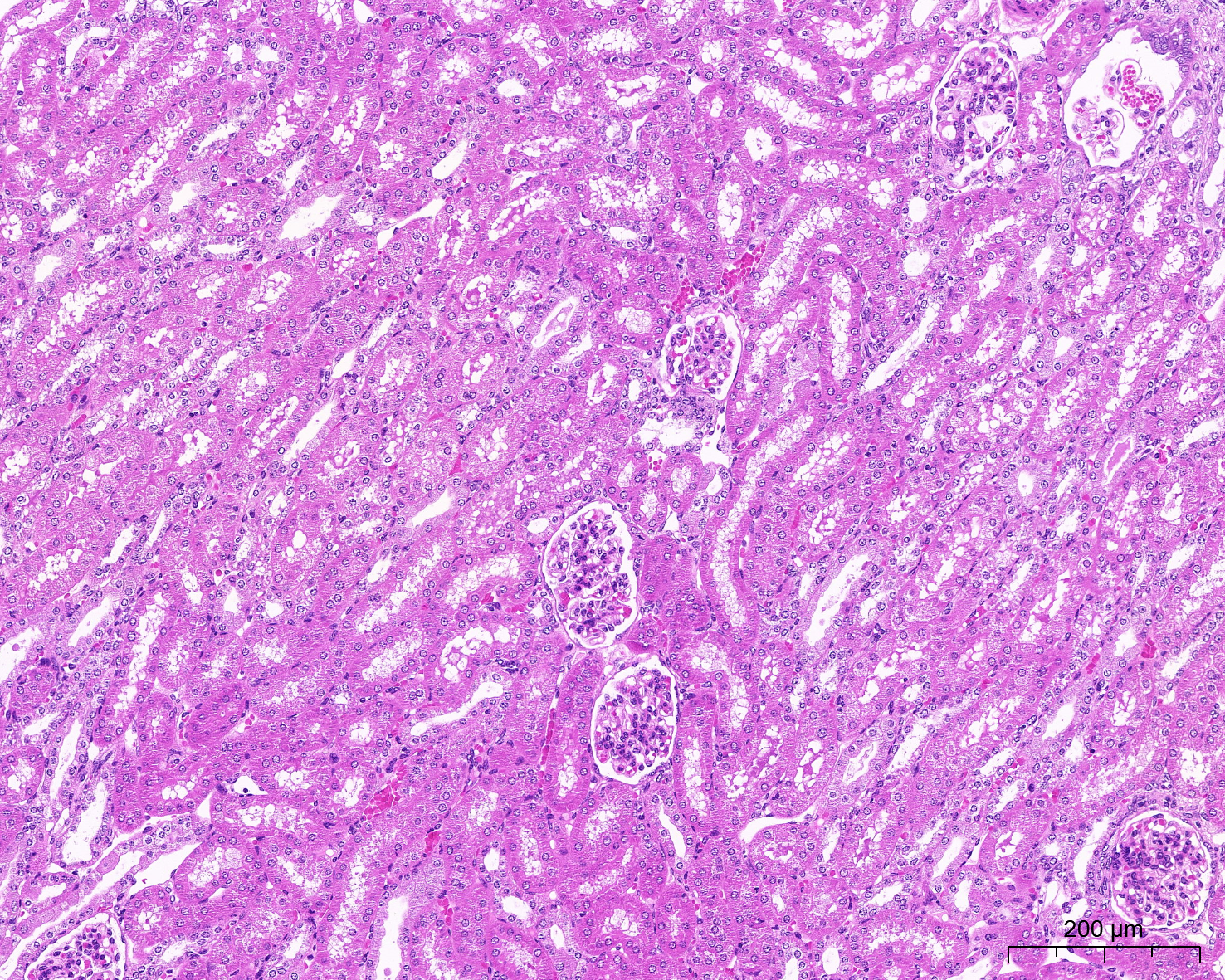

Supplement: Supplementary file 1 [file pharmaceuticals-18-01266-s001.zip › Renal H&E Staining/NC/2.tif]

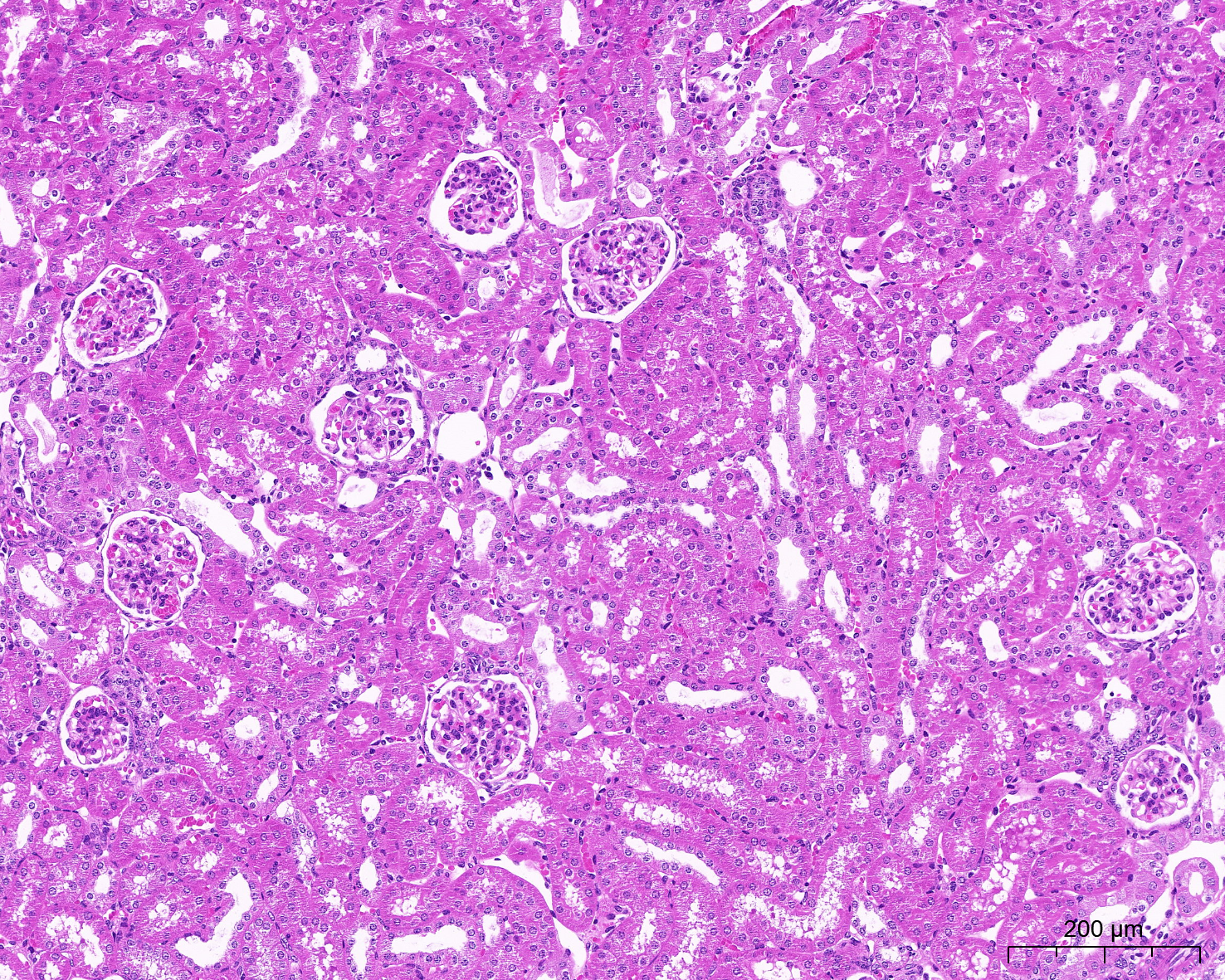

Supplement: Supplementary file 1 [file pharmaceuticals-18-01266-s001.zip › Renal H&E Staining/NC/3.tif]

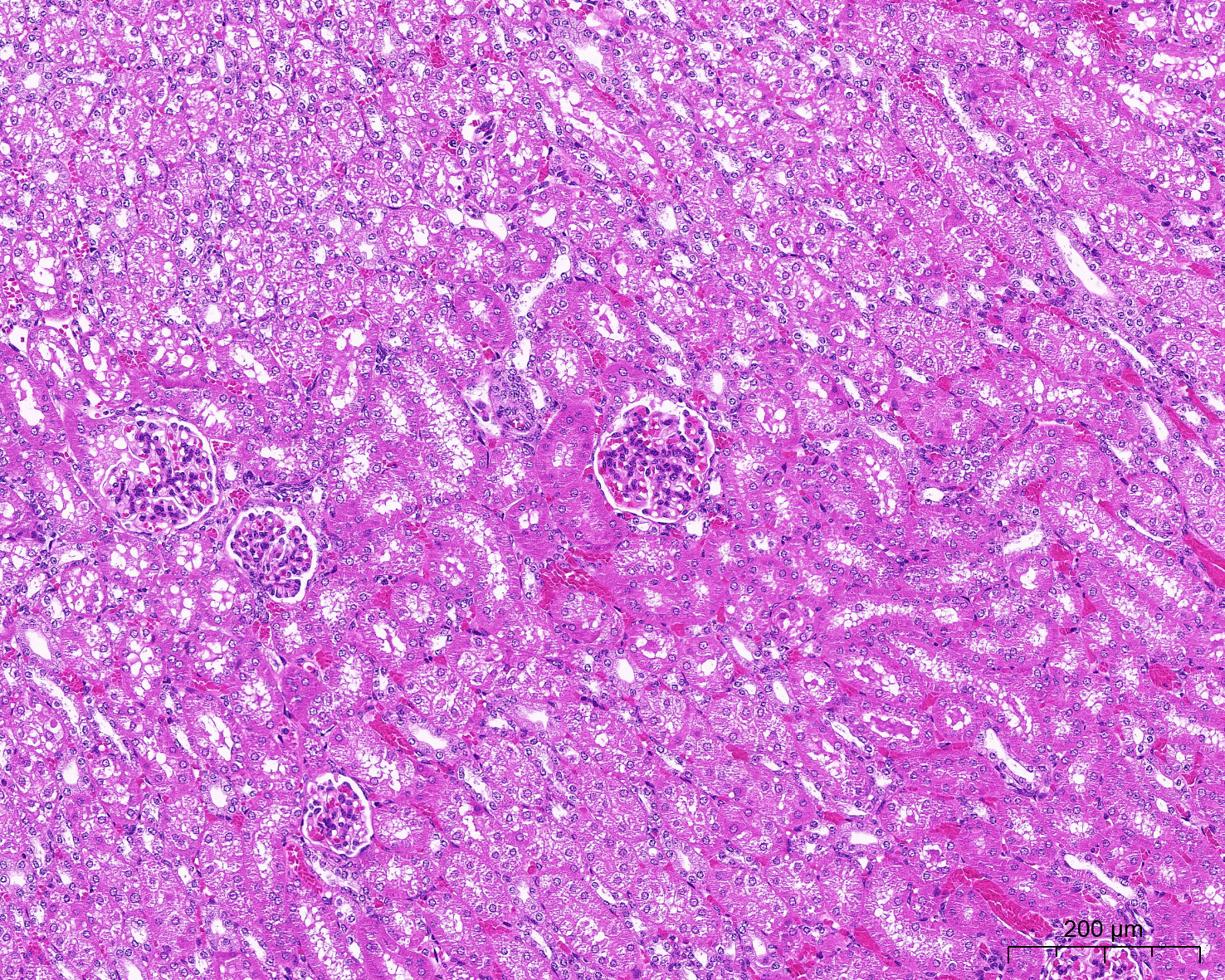

Supplement: Supplementary file 1 [file pharmaceuticals-18-01266-s001.zip › Renal H&E Staining/XYN 0.9/1.tif]

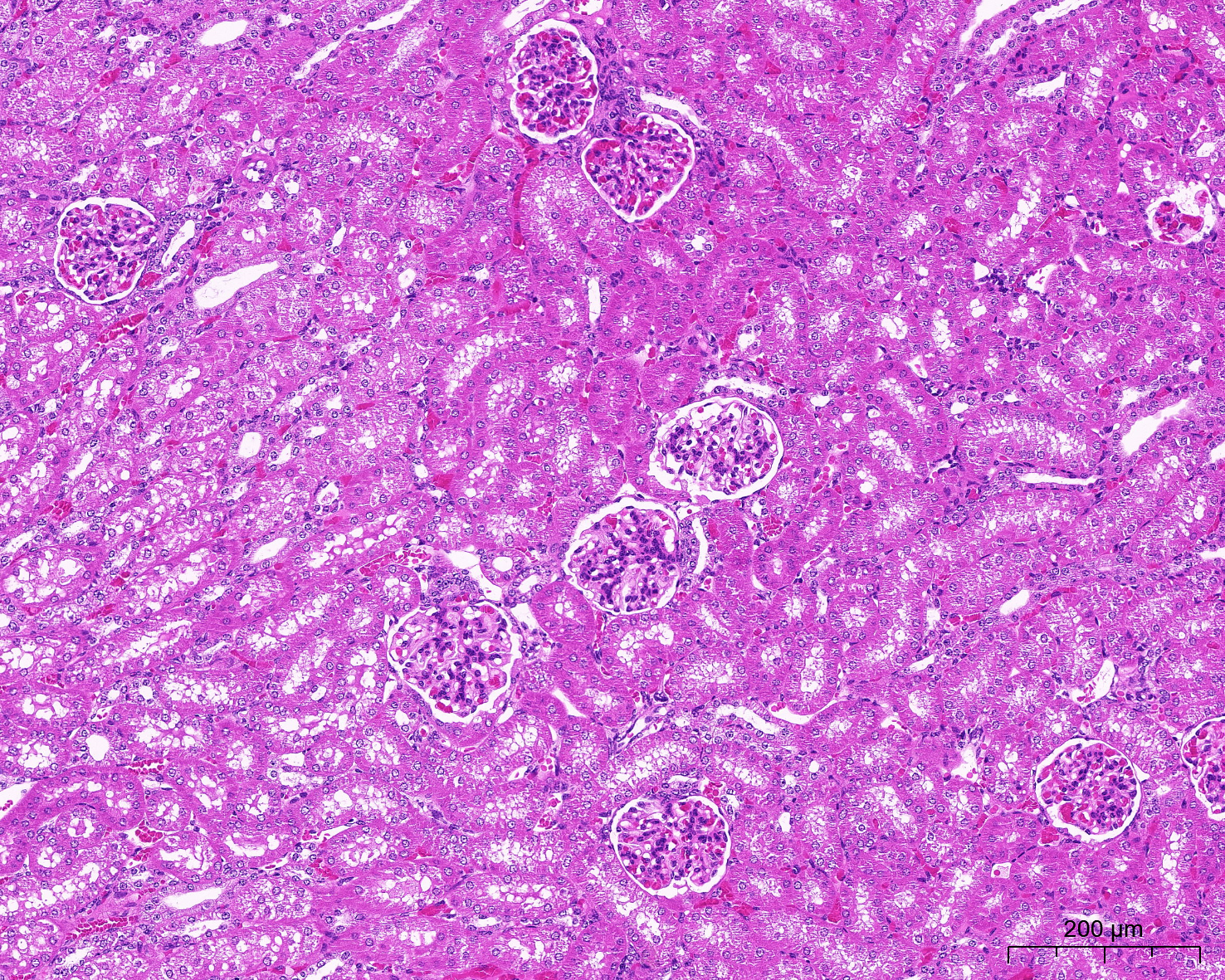

Supplement: Supplementary file 1 [file pharmaceuticals-18-01266-s001.zip › Renal H&E Staining/XYN 0.9/2.tif]

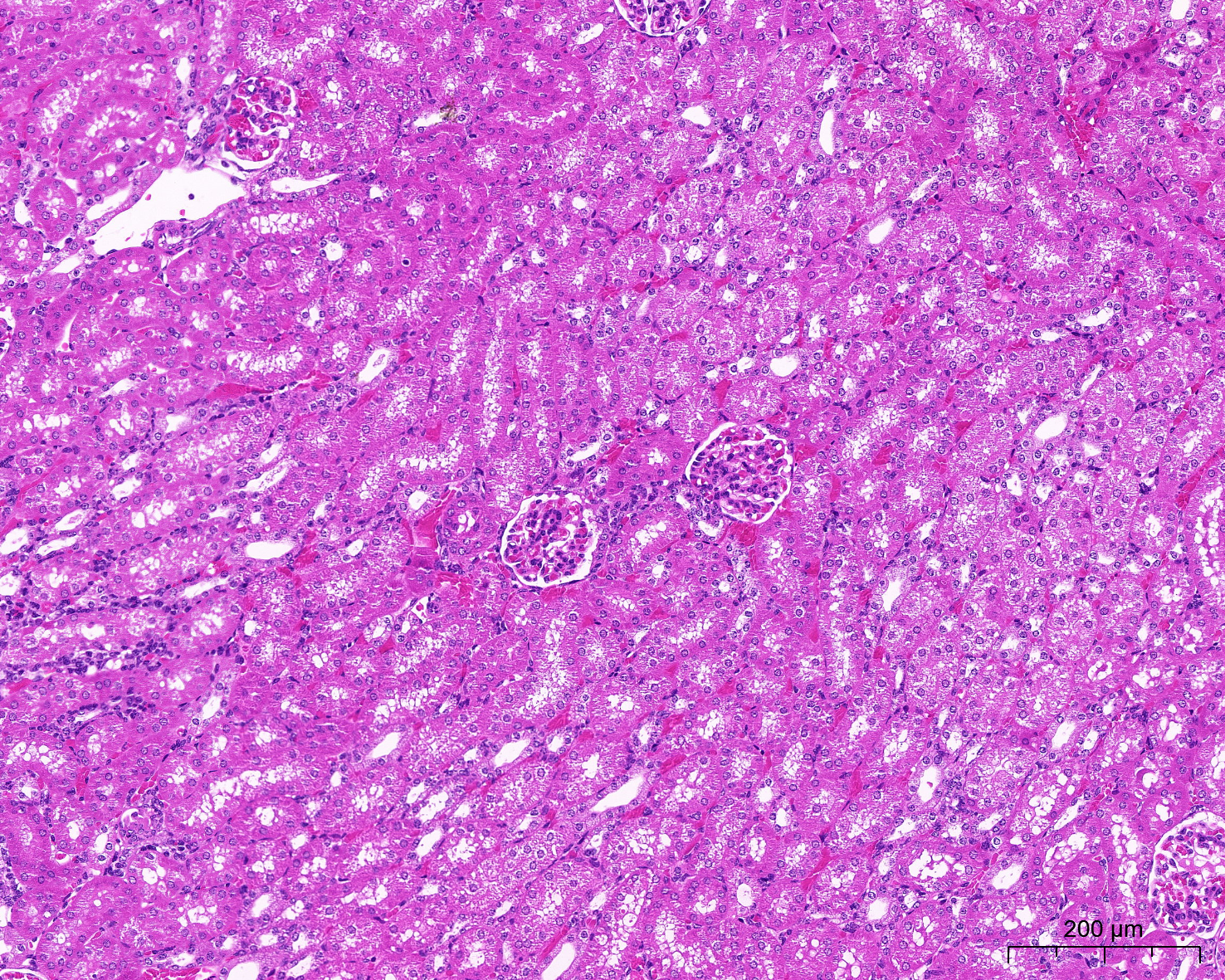

Supplement: Supplementary file 1 [file pharmaceuticals-18-01266-s001.zip › Renal H&E Staining/XYN 0.9/3.tif]

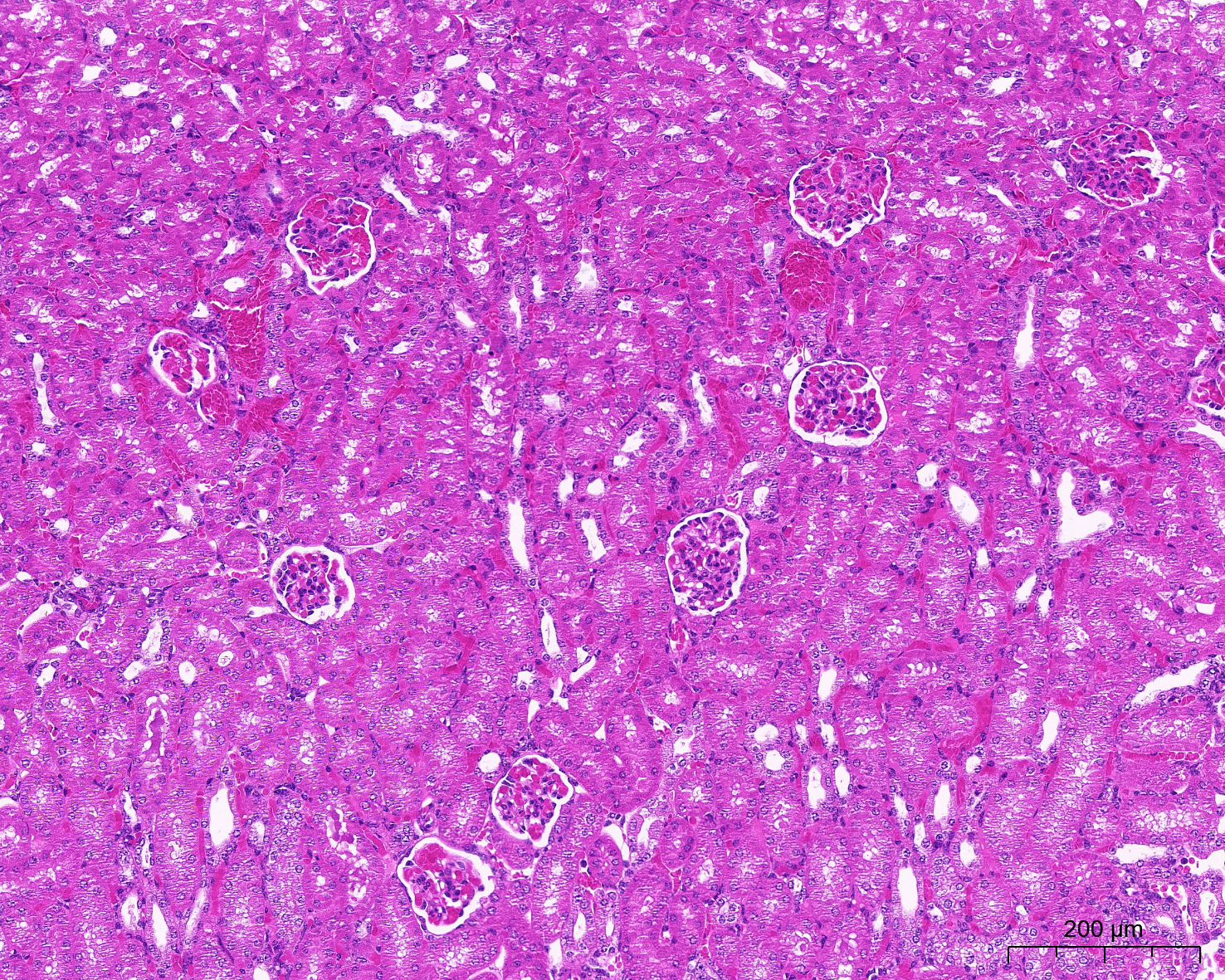

Supplement: Supplementary file 1 [file pharmaceuticals-18-01266-s001.zip › Renal H&E Staining/XYN 1.8/1.tif]

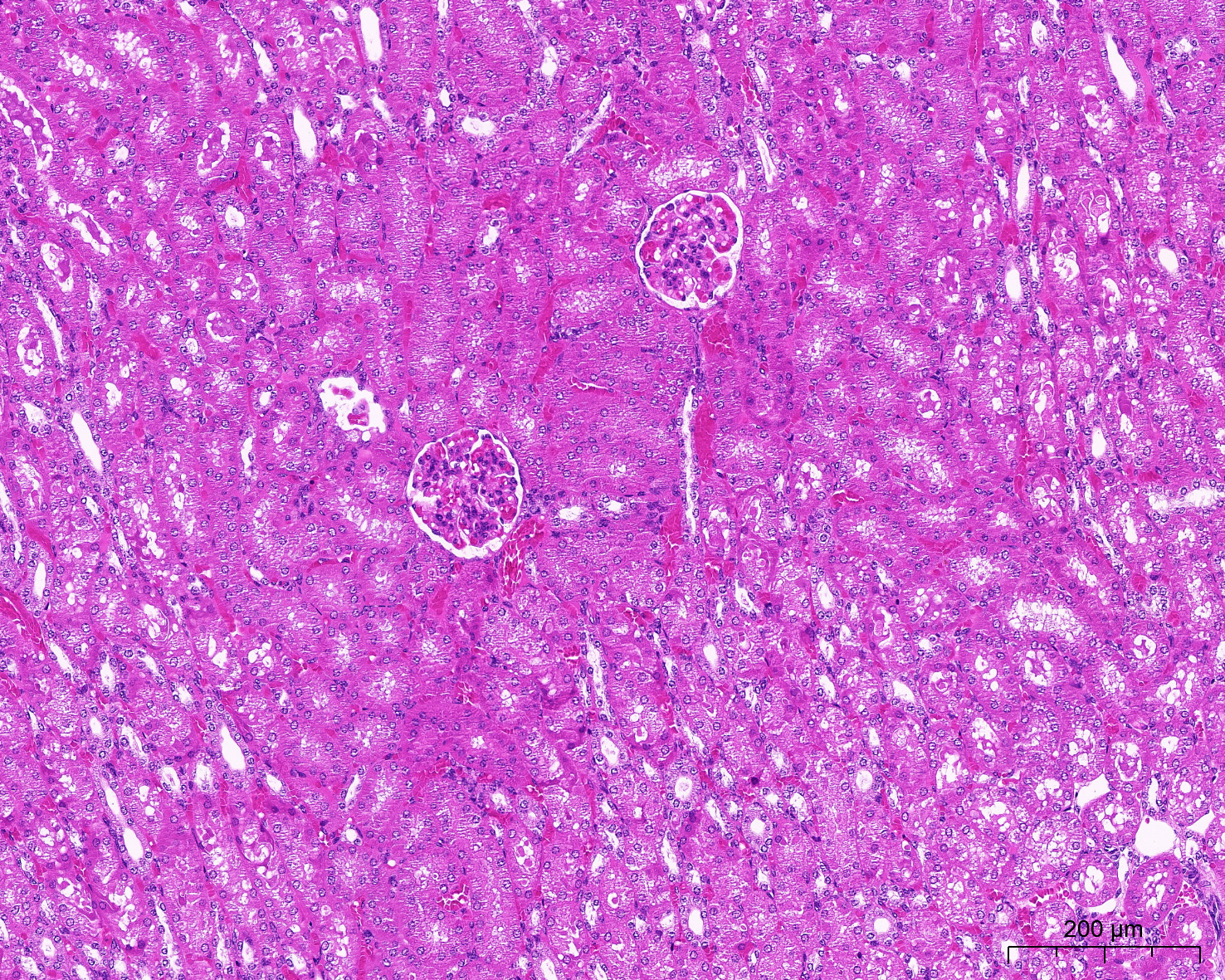

Supplement: Supplementary file 1 [file pharmaceuticals-18-01266-s001.zip › Renal H&E Staining/XYN 1.8/2.tif]

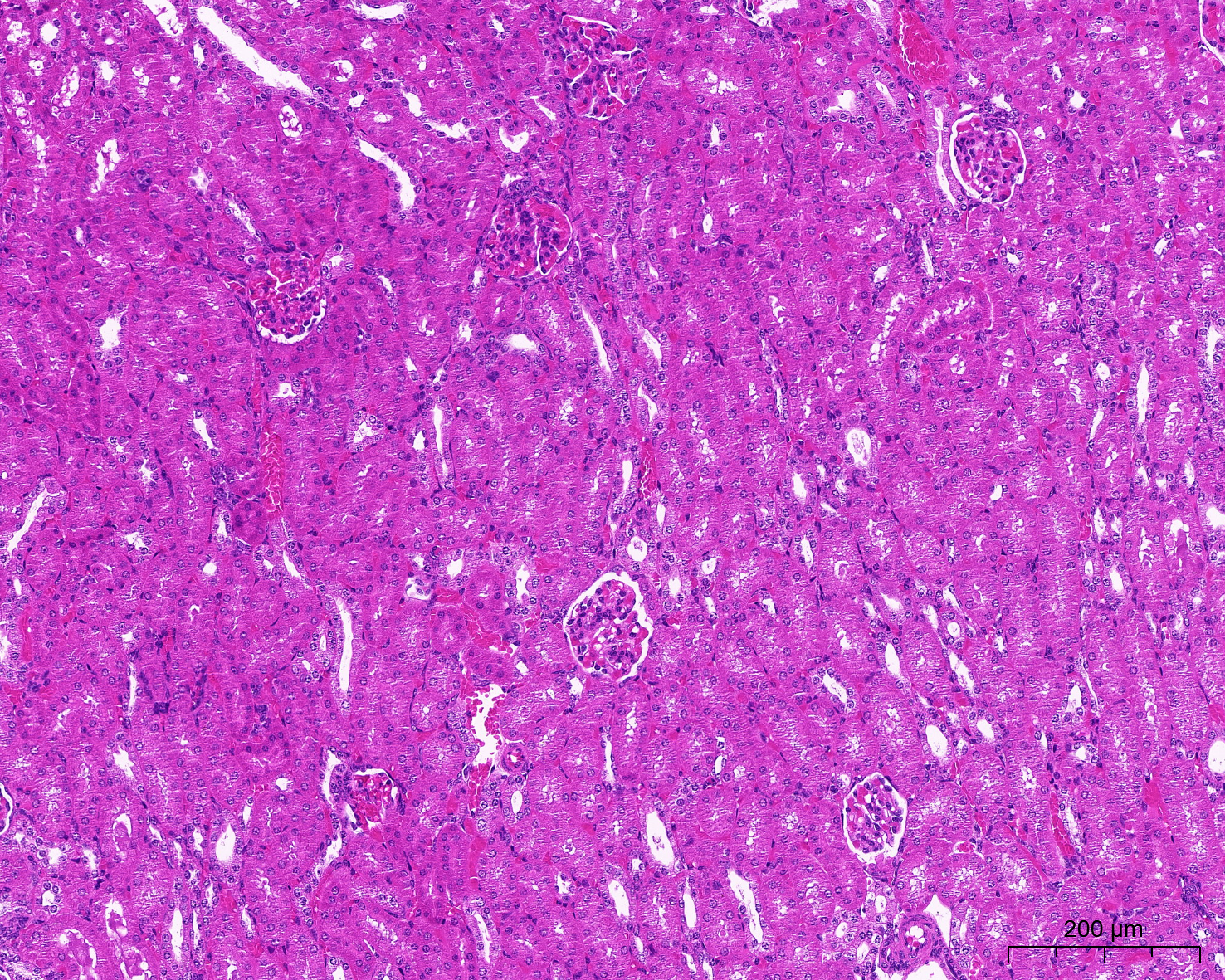

Supplement: Supplementary file 1 [file pharmaceuticals-18-01266-s001.zip › Renal H&E Staining/XYN 1.8/3.tif]

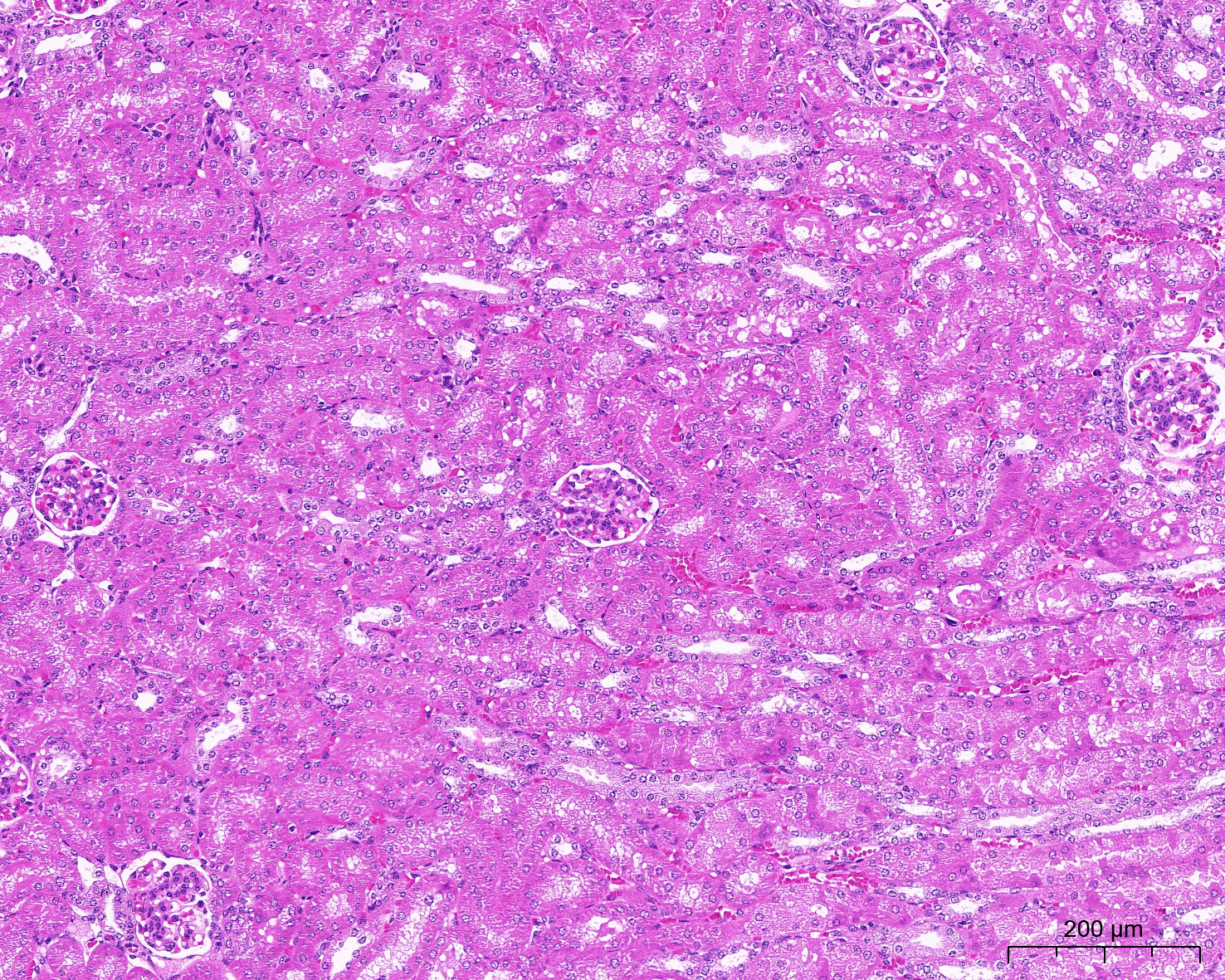

Supplement: Supplementary file 1 [file pharmaceuticals-18-01266-s001.zip › Renal H&E Staining/XYN 3.6/1.tif]

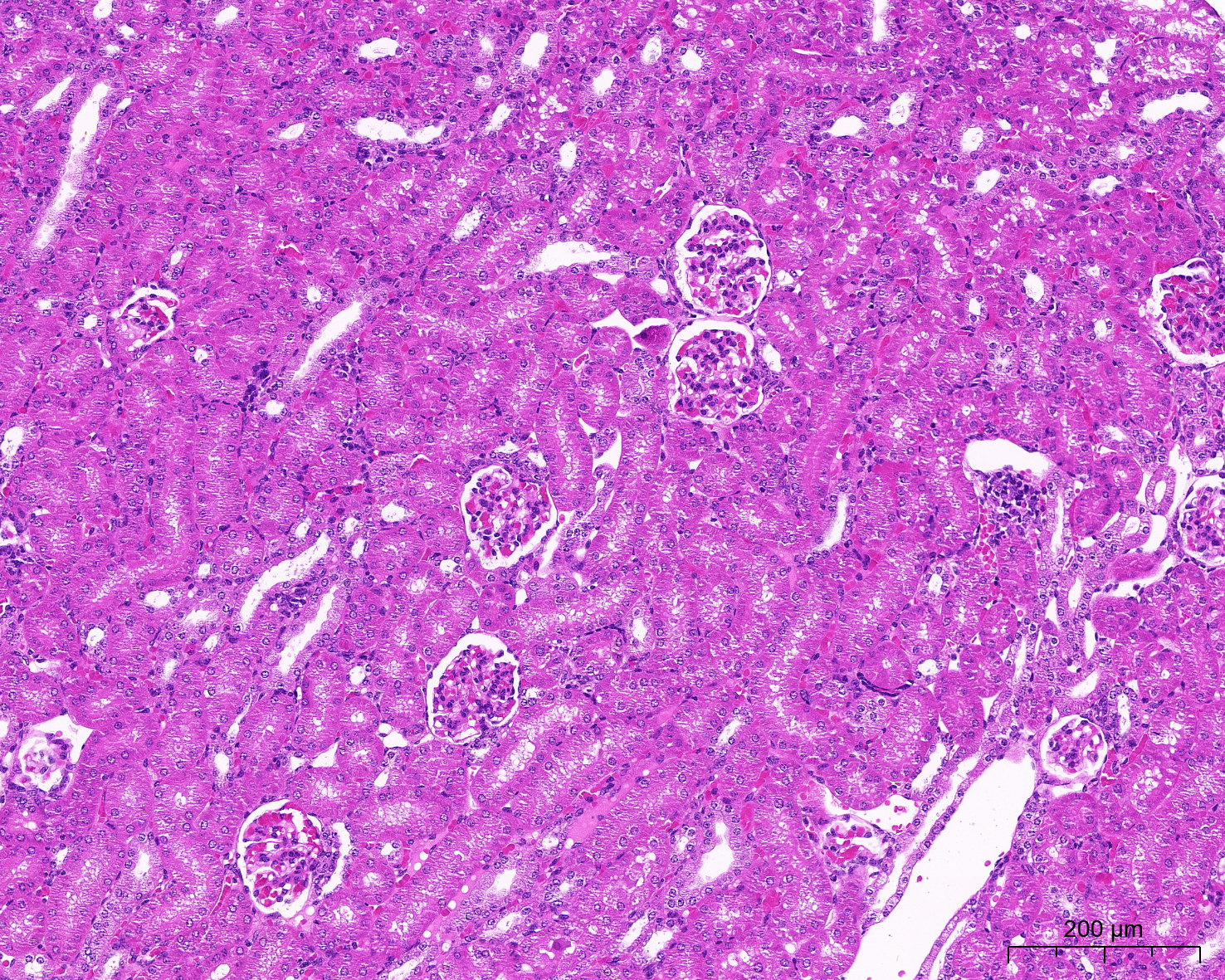

Supplement: Supplementary file 1 [file pharmaceuticals-18-01266-s001.zip › Renal H&E Staining/XYN 3.6/2.tif]

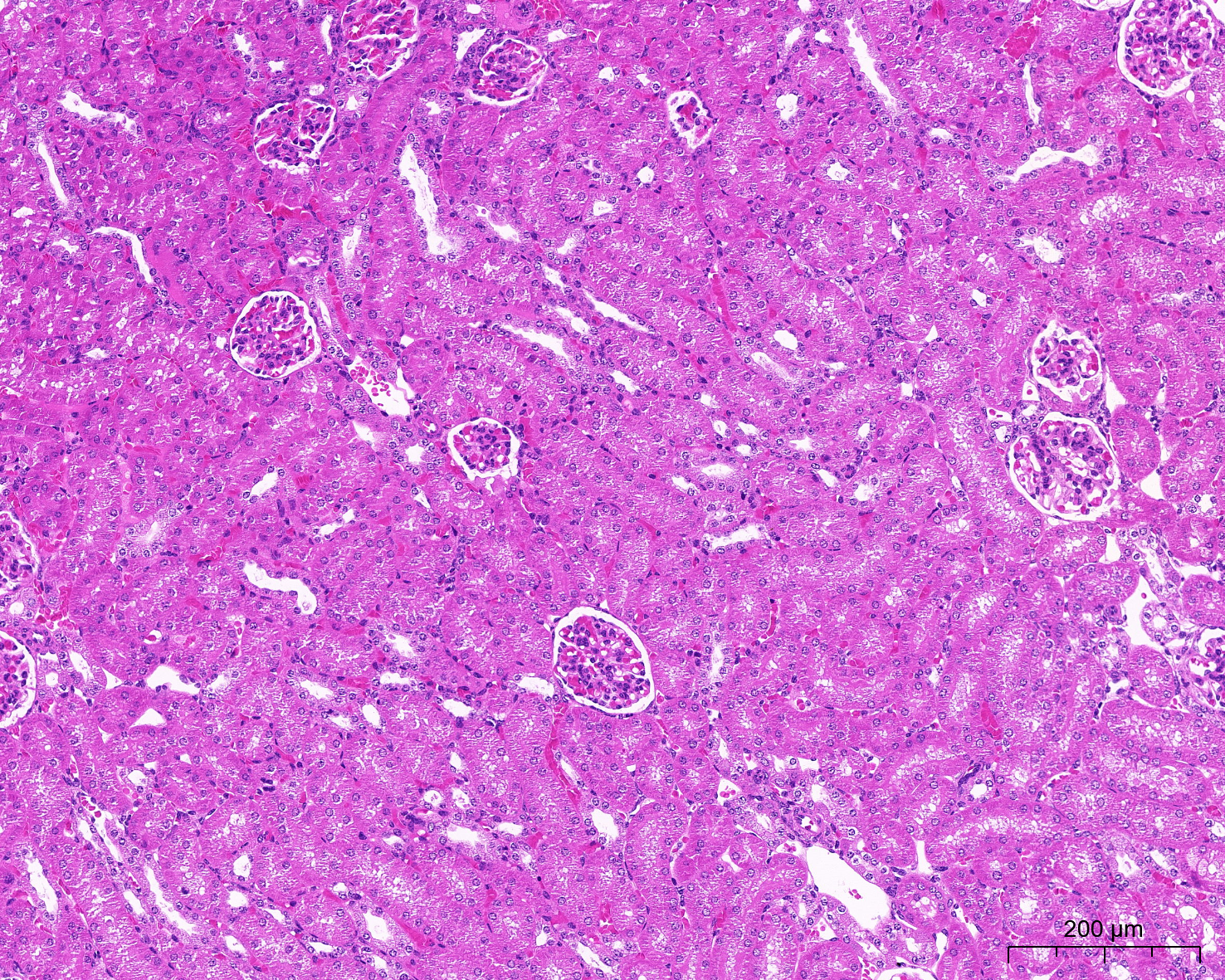

Supplement: Supplementary file 1 [file pharmaceuticals-18-01266-s001.zip › Renal H&E Staining/XYN 3.6/3.tif]

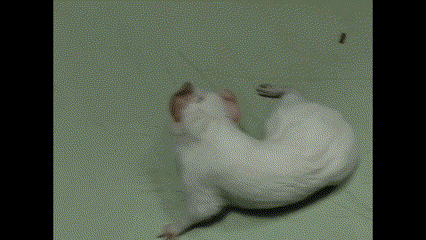

Supplement: Supplementary file 1 [file pharmaceuticals-18-01266-s001.zip › Supplementary GIFs-Behavioral Responses Before and After Drug Intervention in Guinea Pigs/Circling behavior/1. Guinea pigs exhibited circling behavior after modeling.gif]

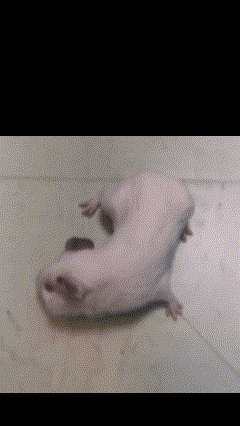

Supplement: Supplementary file 1 [file pharmaceuticals-18-01266-s001.zip › Supplementary GIFs-Behavioral Responses Before and After Drug Intervention in Guinea Pigs/Circling behavior/2. Circling behavior was alleviated after drug administration.gif]

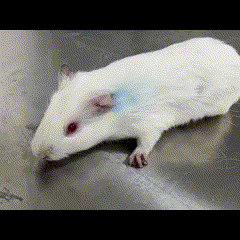

Supplement: Supplementary file 1 [file pharmaceuticals-18-01266-s001.zip › Supplementary GIFs-Behavioral Responses Before and After Drug Intervention in Guinea Pigs/Hearing function/1. Hearing loss was observed in guinea pigs after modeling.gif]

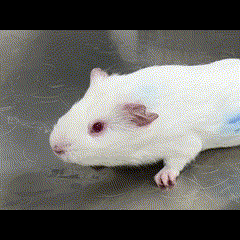

Supplement: Supplementary file 1 [file pharmaceuticals-18-01266-s001.zip › Supplementary GIFs-Behavioral Responses Before and After Drug Intervention in Guinea Pigs/Hearing function/2. Hearing function improved following drug treatment.gif]

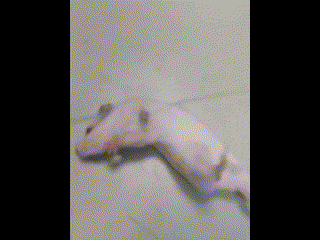

Supplement: Supplementary file 1 [file pharmaceuticals-18-01266-s001.zip › Supplementary GIFs-Behavioral Responses Before and After Drug Intervention in Guinea Pigs/Motor coordination/1. Impaired motor coordination was observed after modeling.gif]

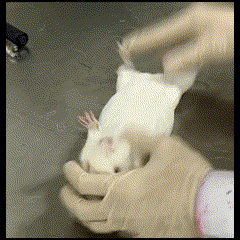

Supplement: Supplementary file 1 [file pharmaceuticals-18-01266-s001.zip › Supplementary GIFs-Behavioral Responses Before and After Drug Intervention in Guinea Pigs/Motor coordination/2. Motor coordination was enhanced after drug administration.gif]

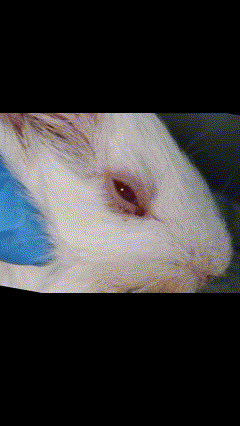

Supplement: Supplementary file 1 [file pharmaceuticals-18-01266-s001.zip › Supplementary GIFs-Behavioral Responses Before and After Drug Intervention in Guinea Pigs/Nystagmus/1. Nystagmus was observed in guinea pigs after modeling.gif]

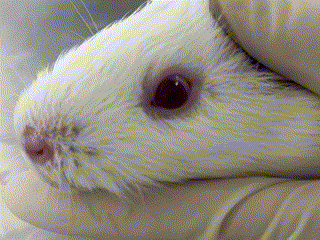

Supplement: Supplementary file 1 [file pharmaceuticals-18-01266-s001.zip › Supplementary GIFs-Behavioral Responses Before and After Drug Intervention in Guinea Pigs/Nystagmus/2. Nystagmus was reduced after drug treatment.gif]

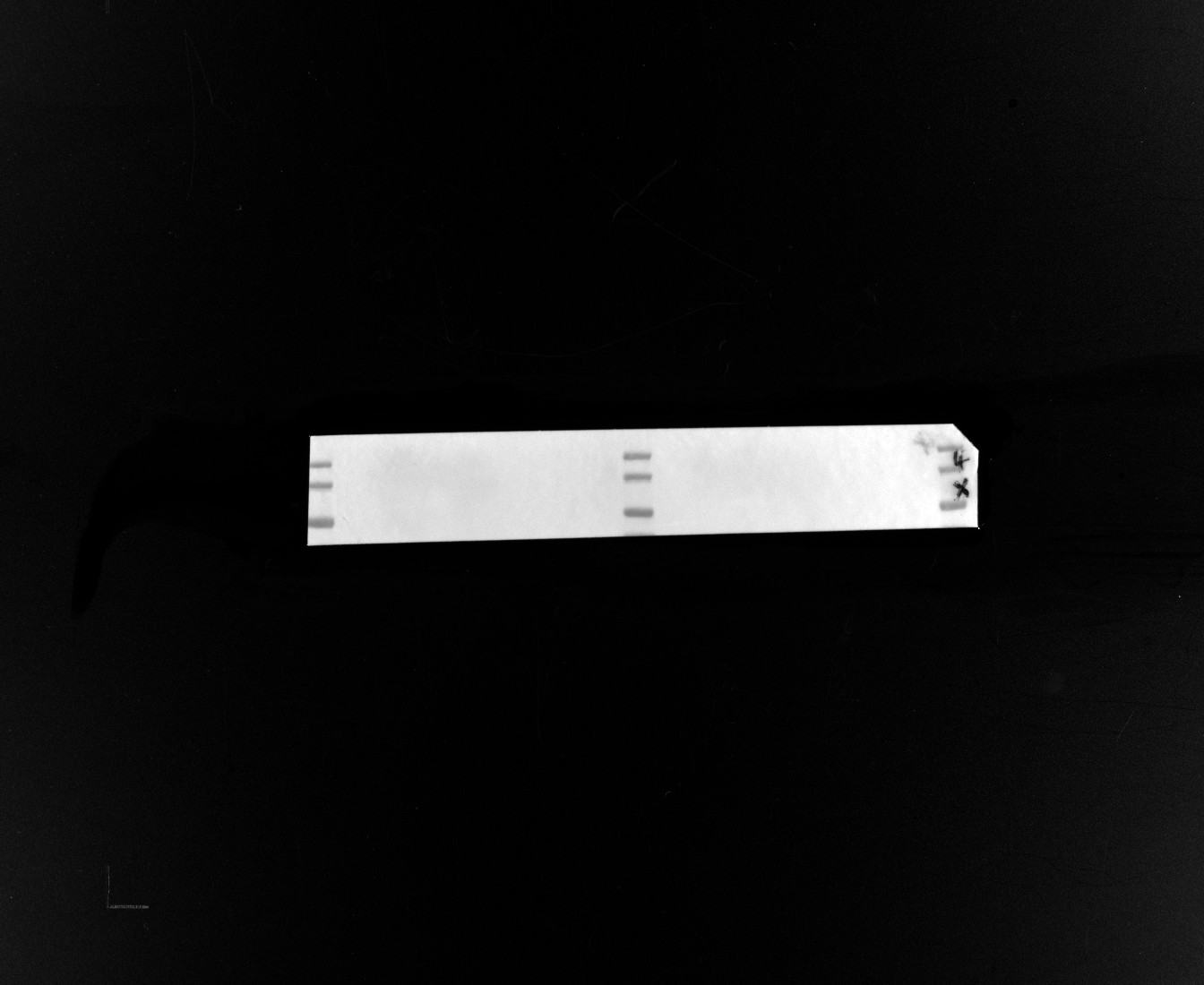

Supplement: Supplementary file 1 [file pharmaceuticals-18-01266-s001.zip › Western blot/AOX/n1-n2 [Brightfield][AOX].tif]

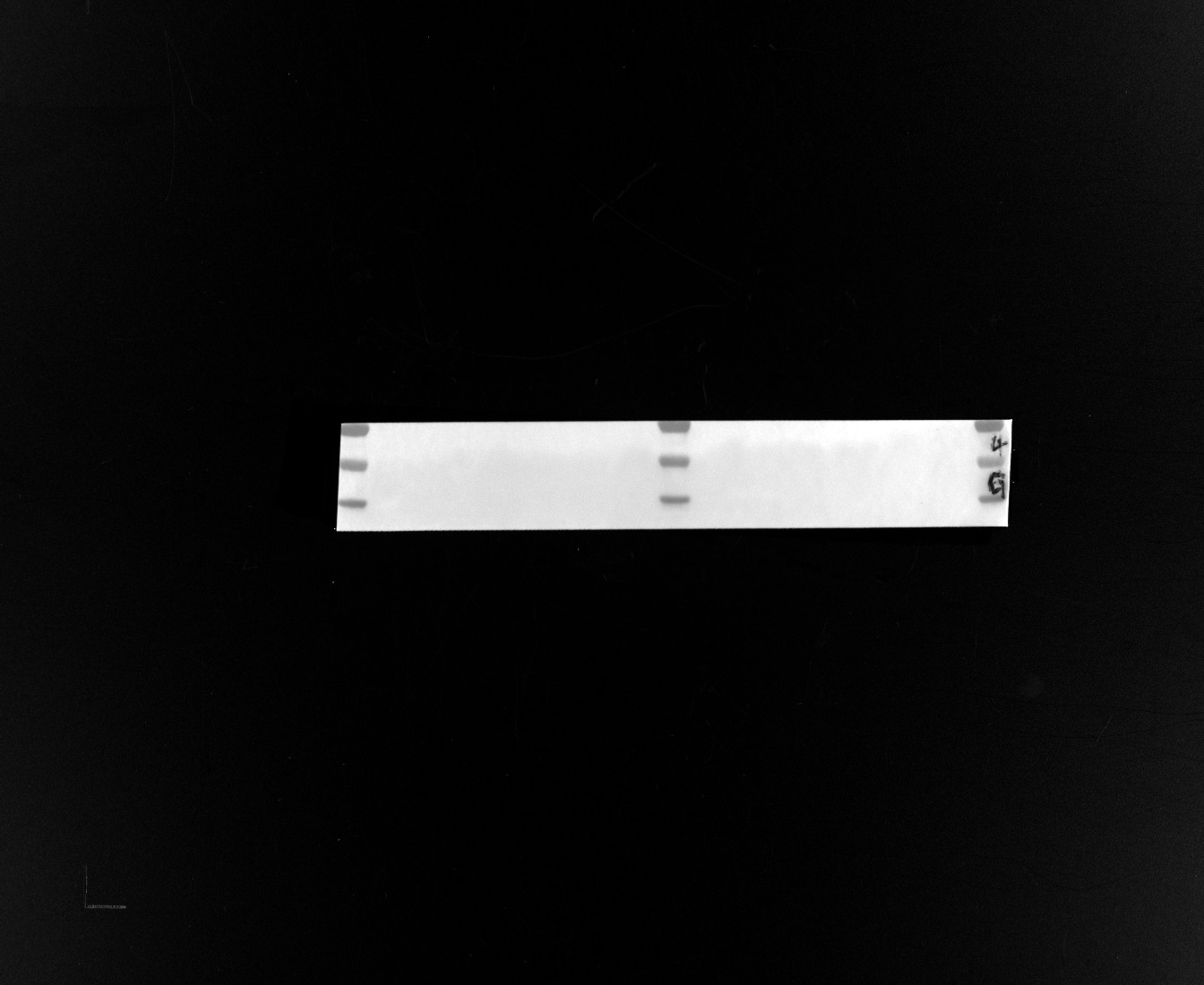

Supplement: Supplementary file 1 [file pharmaceuticals-18-01266-s001.zip › Western blot/AOX/n1-n2 [Brightfield][GAPDH].tif]

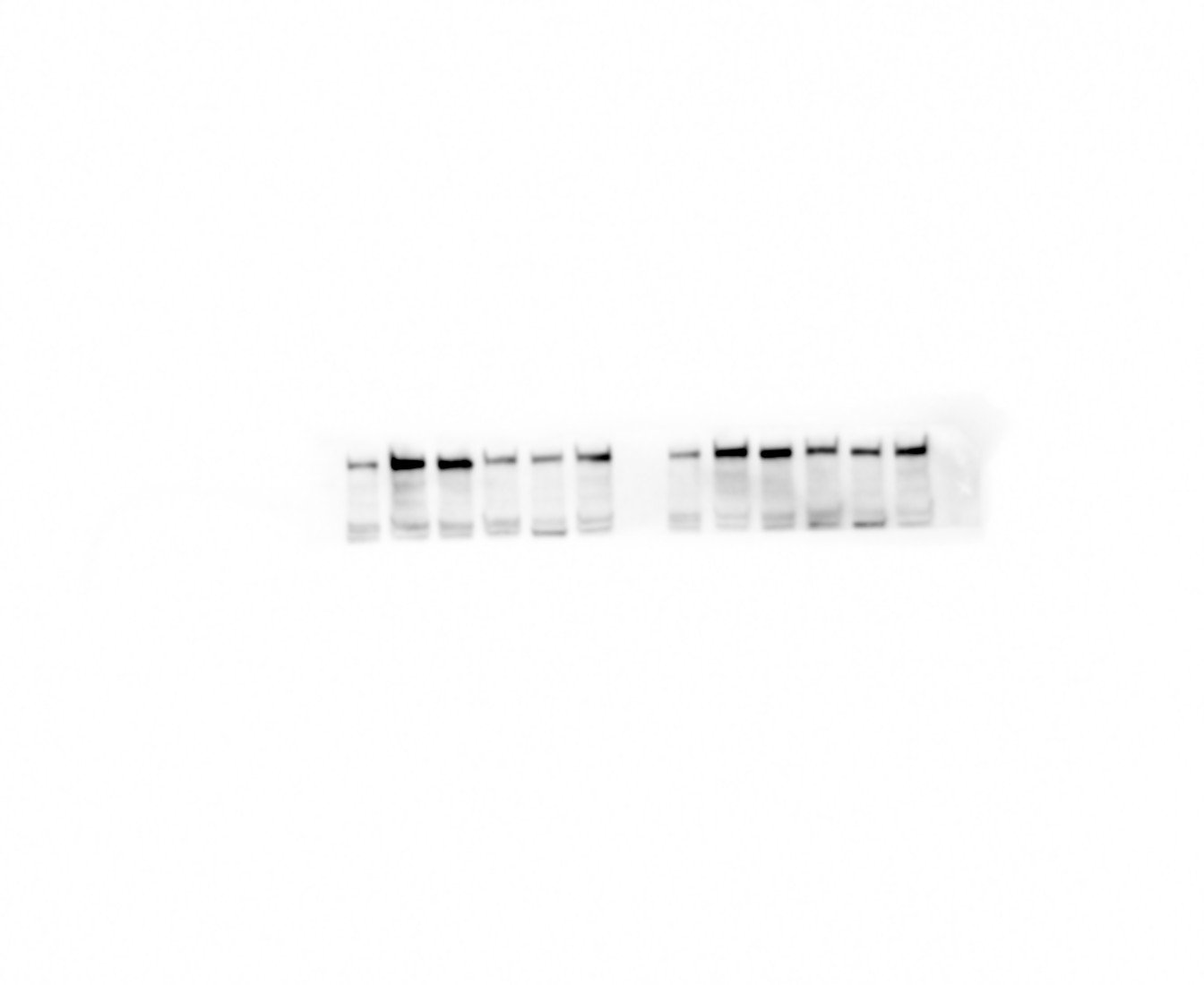

Supplement: Supplementary file 1 [file pharmaceuticals-18-01266-s001.zip › Western blot/AOX/n1-n2 [Luminescence][AOX].tif]

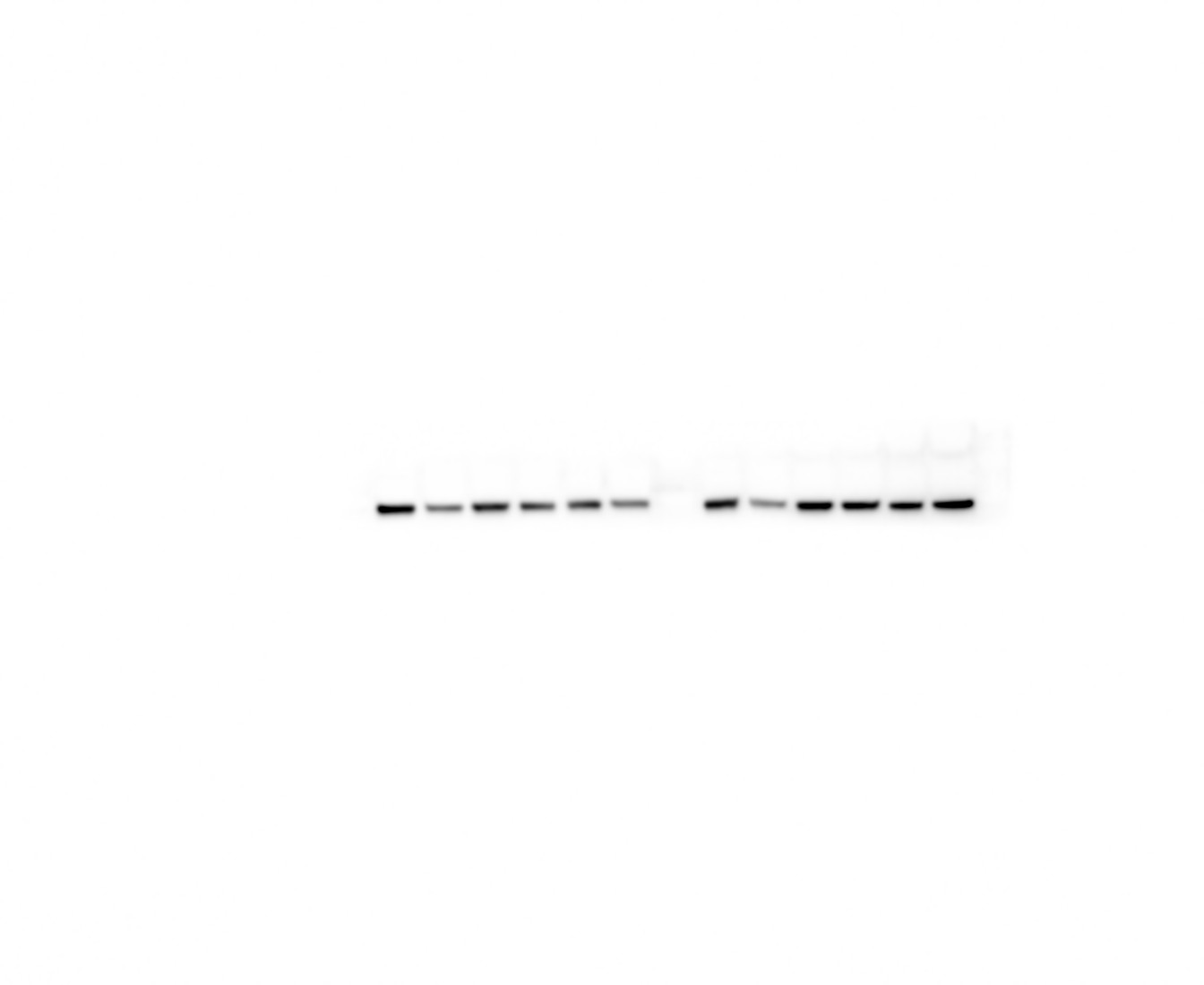

Supplement: Supplementary file 1 [file pharmaceuticals-18-01266-s001.zip › Western blot/AOX/n1-n2 [Luminescence][GAPDH].tif]

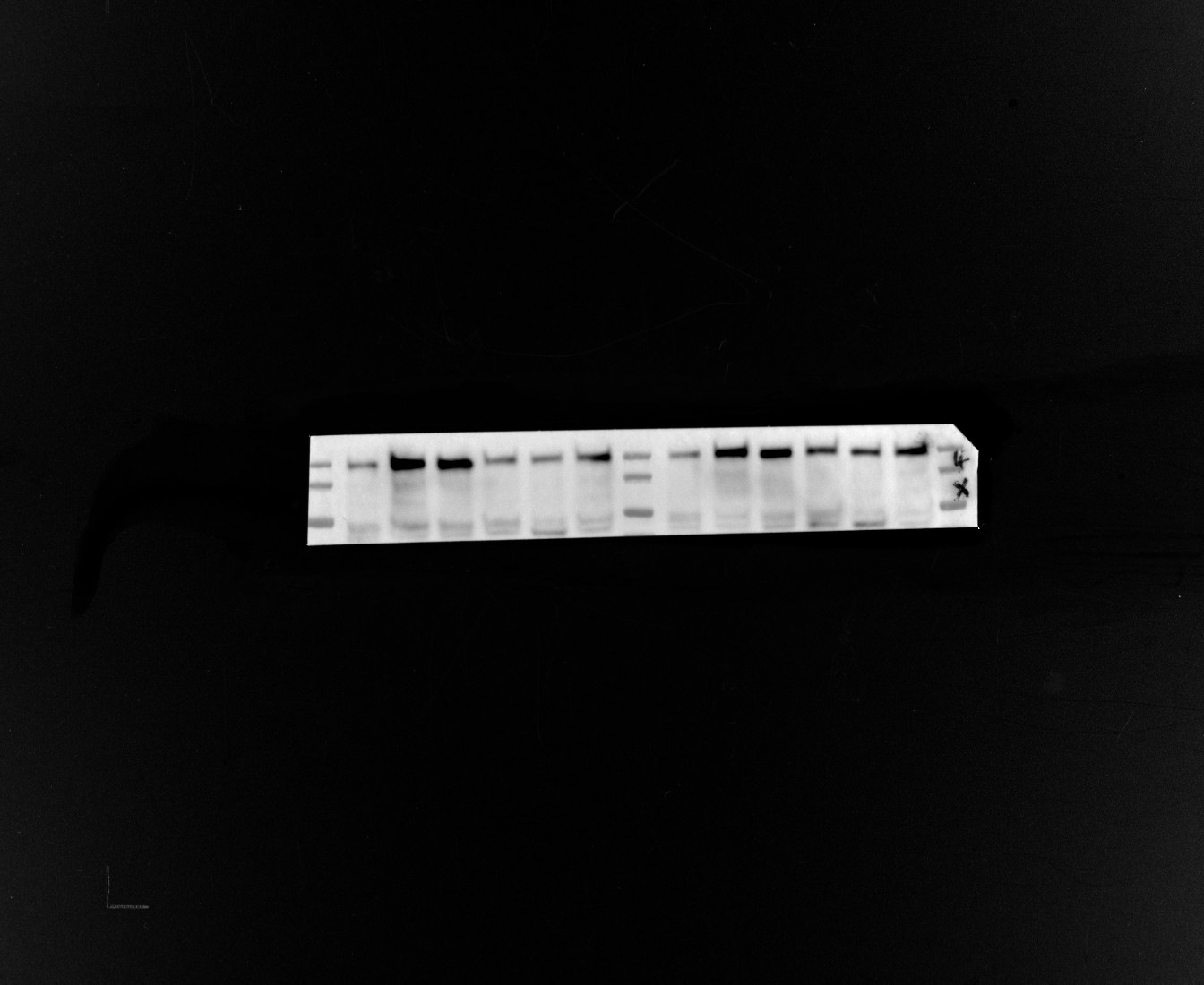

Supplement: Supplementary file 1 [file pharmaceuticals-18-01266-s001.zip › Western blot/AOX/n1-n2 [Overlay][AOX].tif]

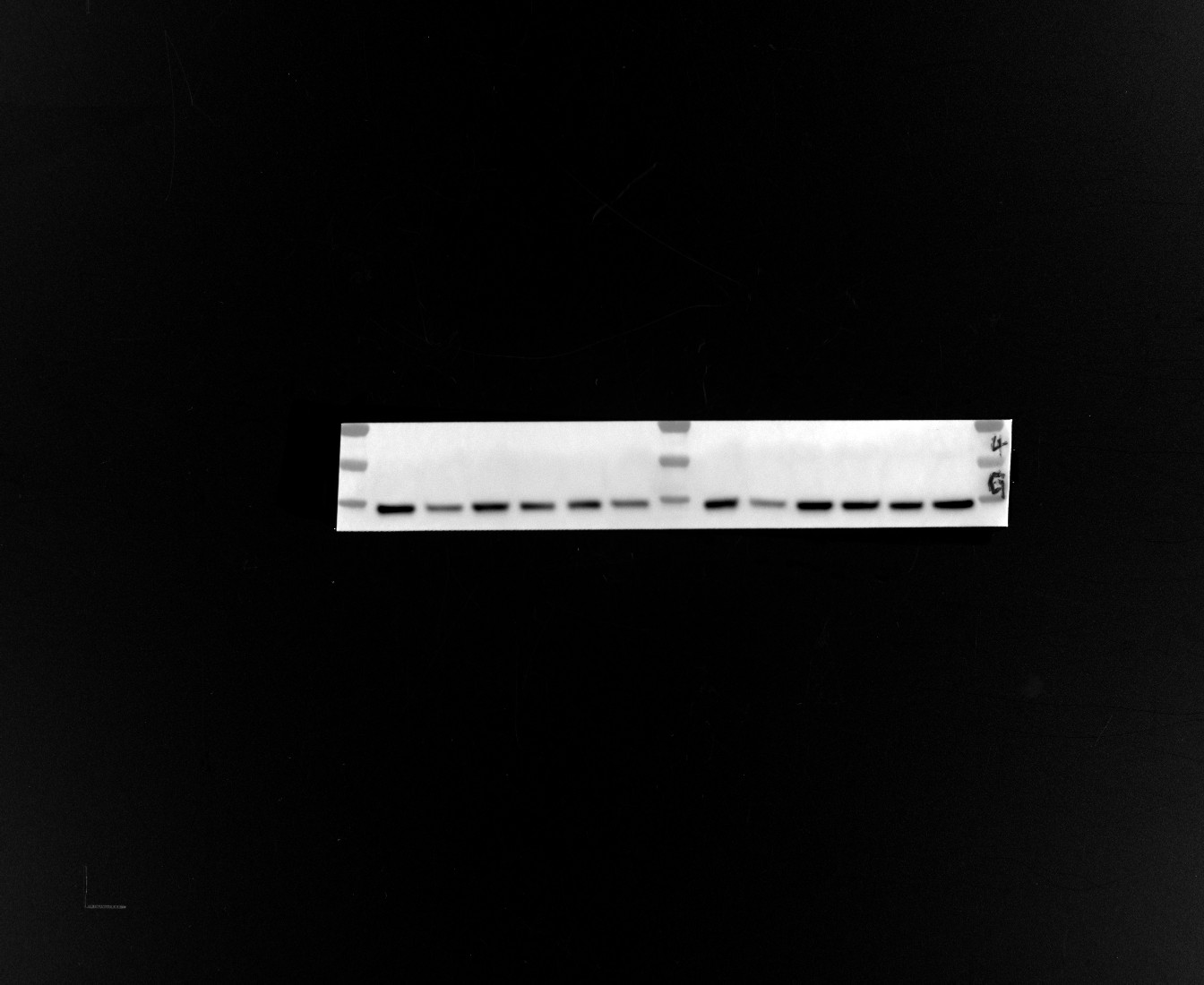

Supplement: Supplementary file 1 [file pharmaceuticals-18-01266-s001.zip › Western blot/AOX/n1-n2 [Overlay][GAPDH].tif]

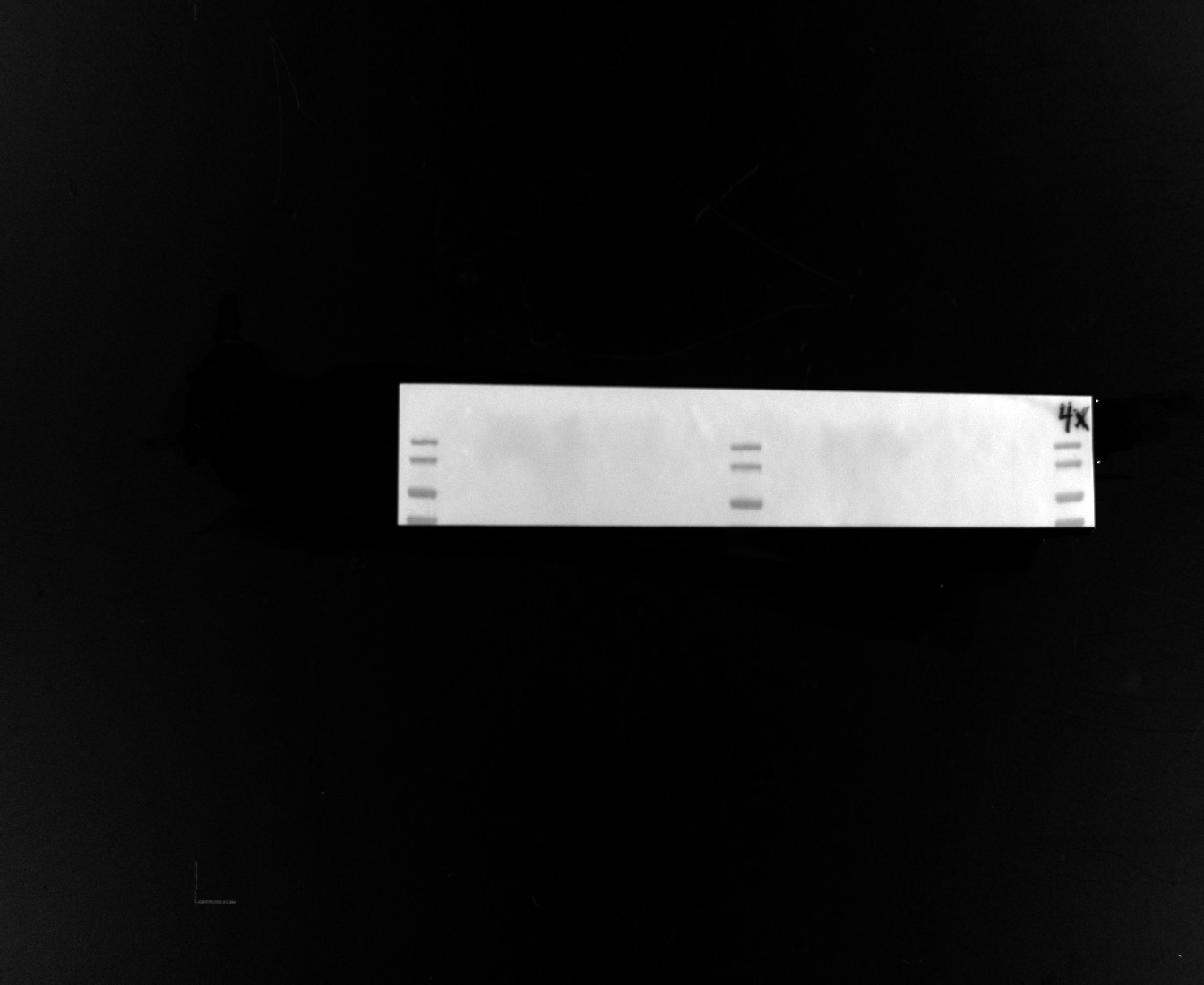

Supplement: Supplementary file 1 [file pharmaceuticals-18-01266-s001.zip › Western blot/AOX/n3-n4 [Brightfield][AOX].tif]

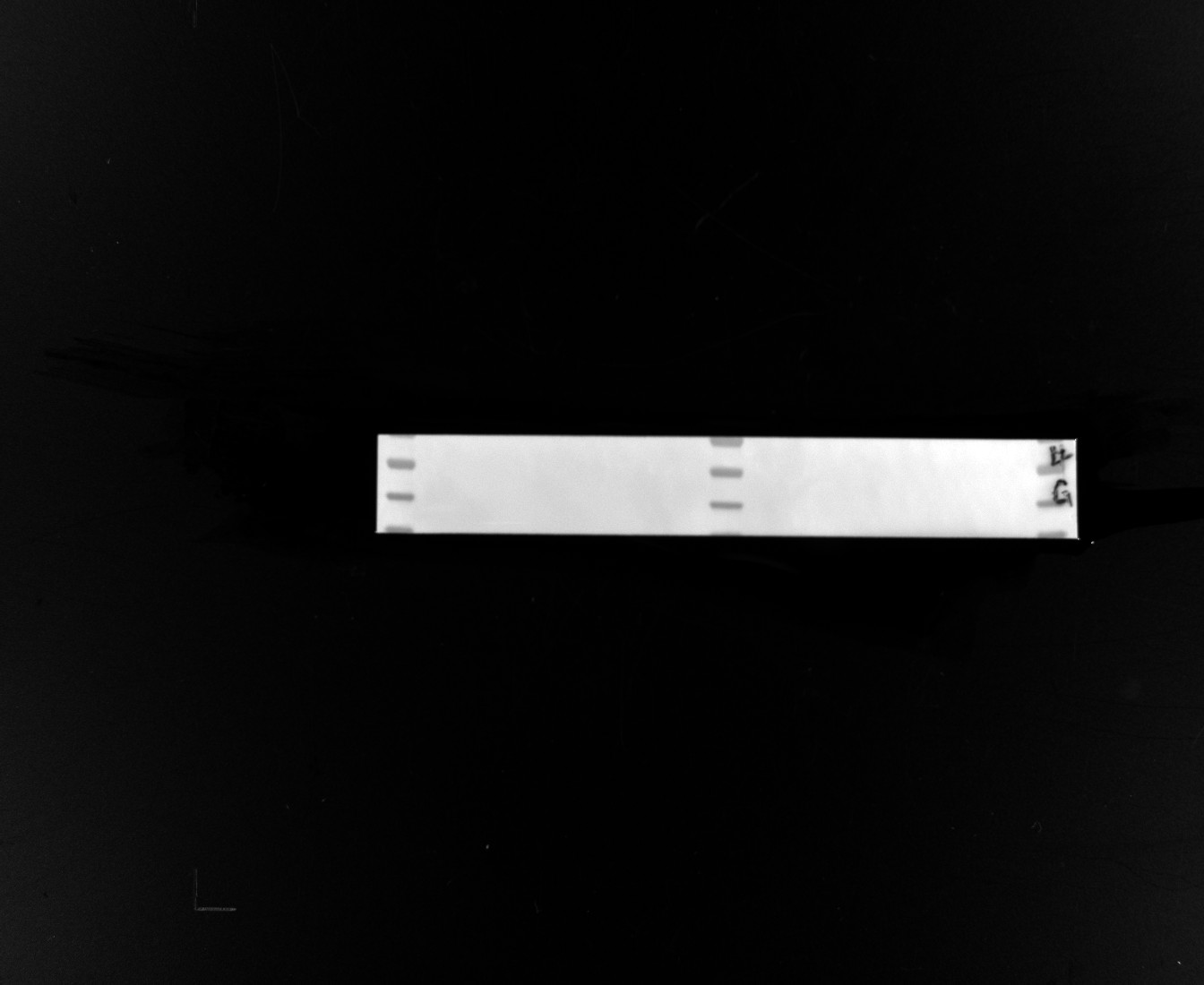

Supplement: Supplementary file 1 [file pharmaceuticals-18-01266-s001.zip › Western blot/AOX/n3-n4 [Brightfield][GAPDH].tif]

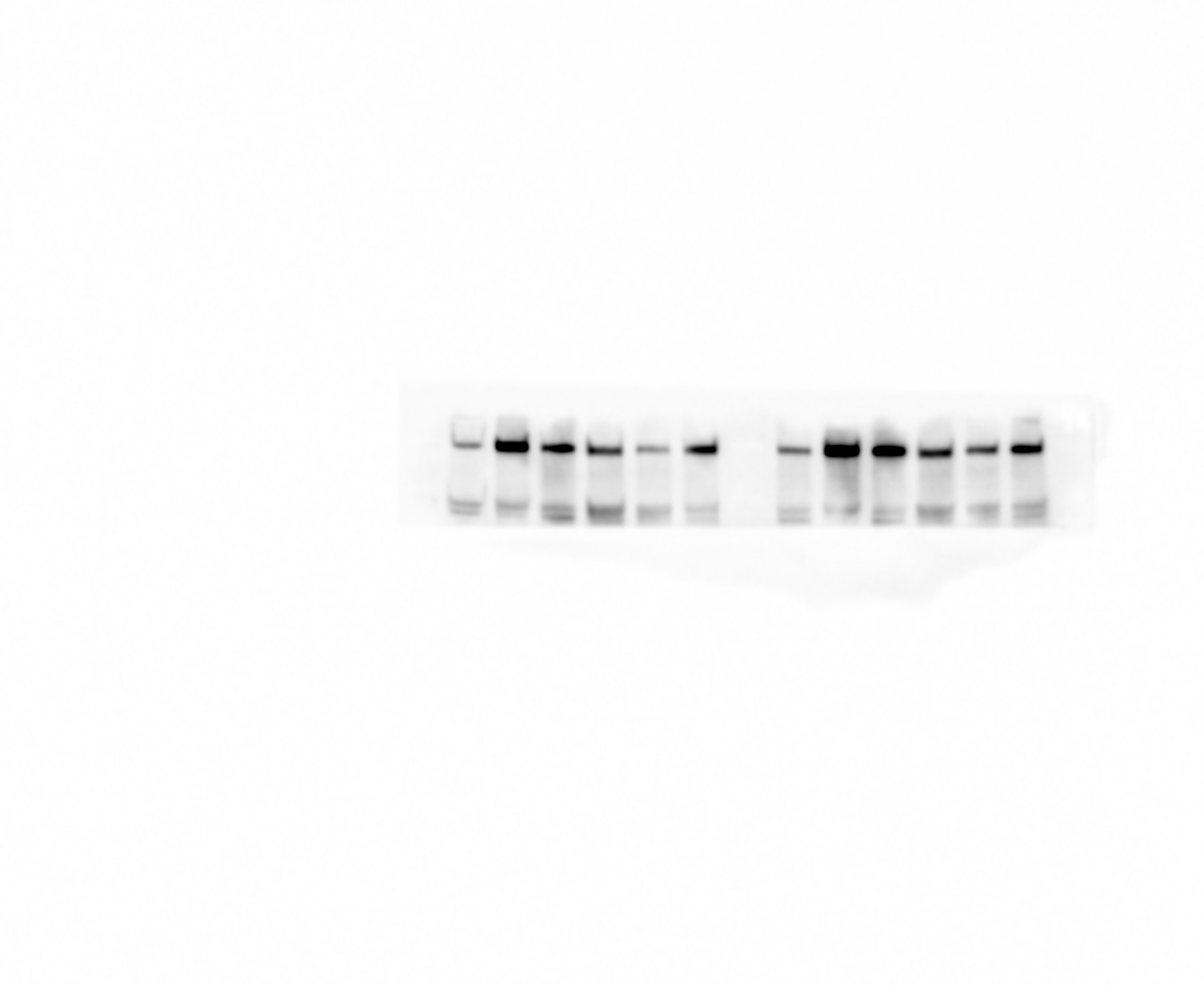

Supplement: Supplementary file 1 [file pharmaceuticals-18-01266-s001.zip › Western blot/AOX/n3-n4 [Luminescence][AOX].tif]

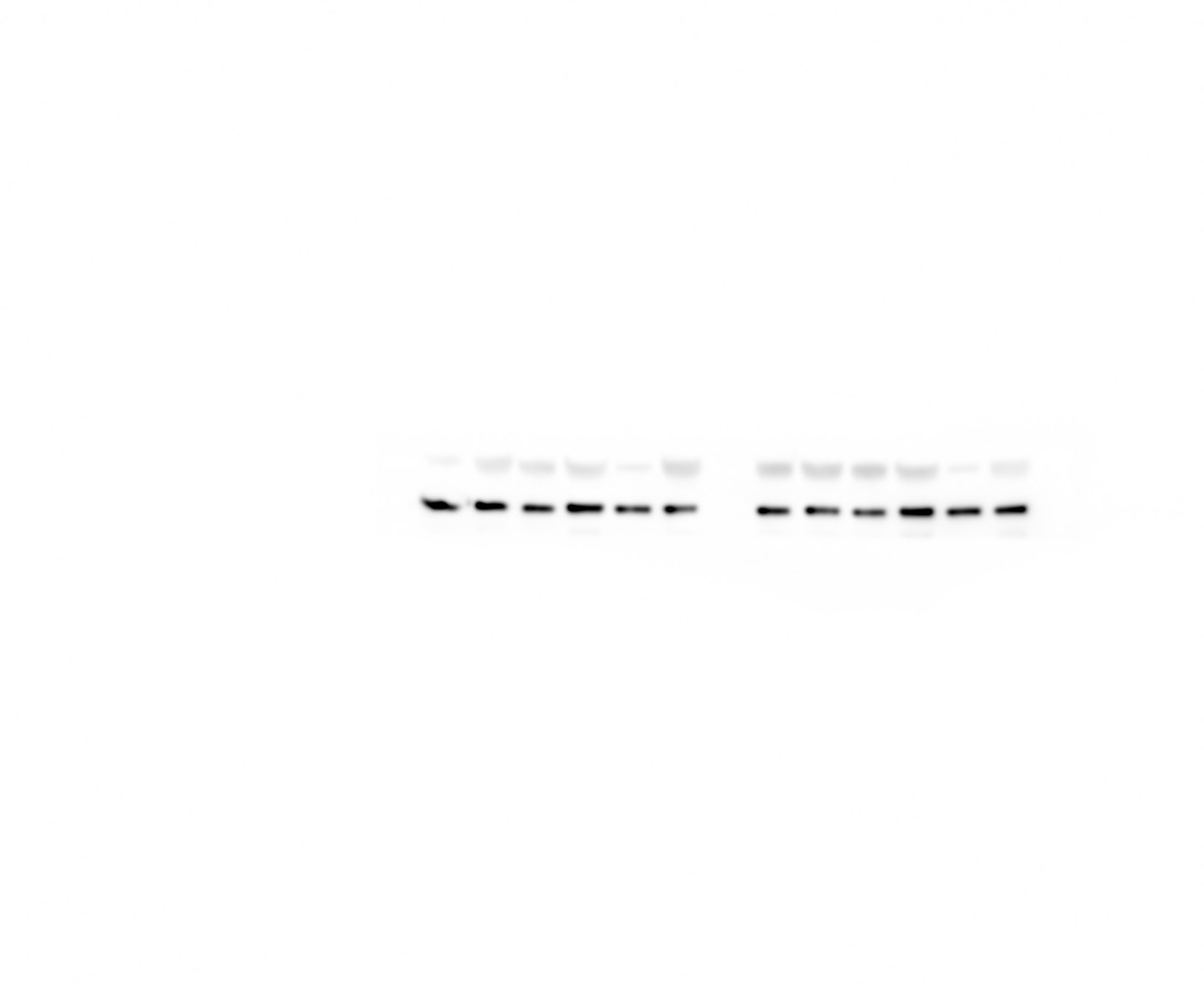

Supplement: Supplementary file 1 [file pharmaceuticals-18-01266-s001.zip › Western blot/AOX/n3-n4 [Luminescence][GAPDH].tif]

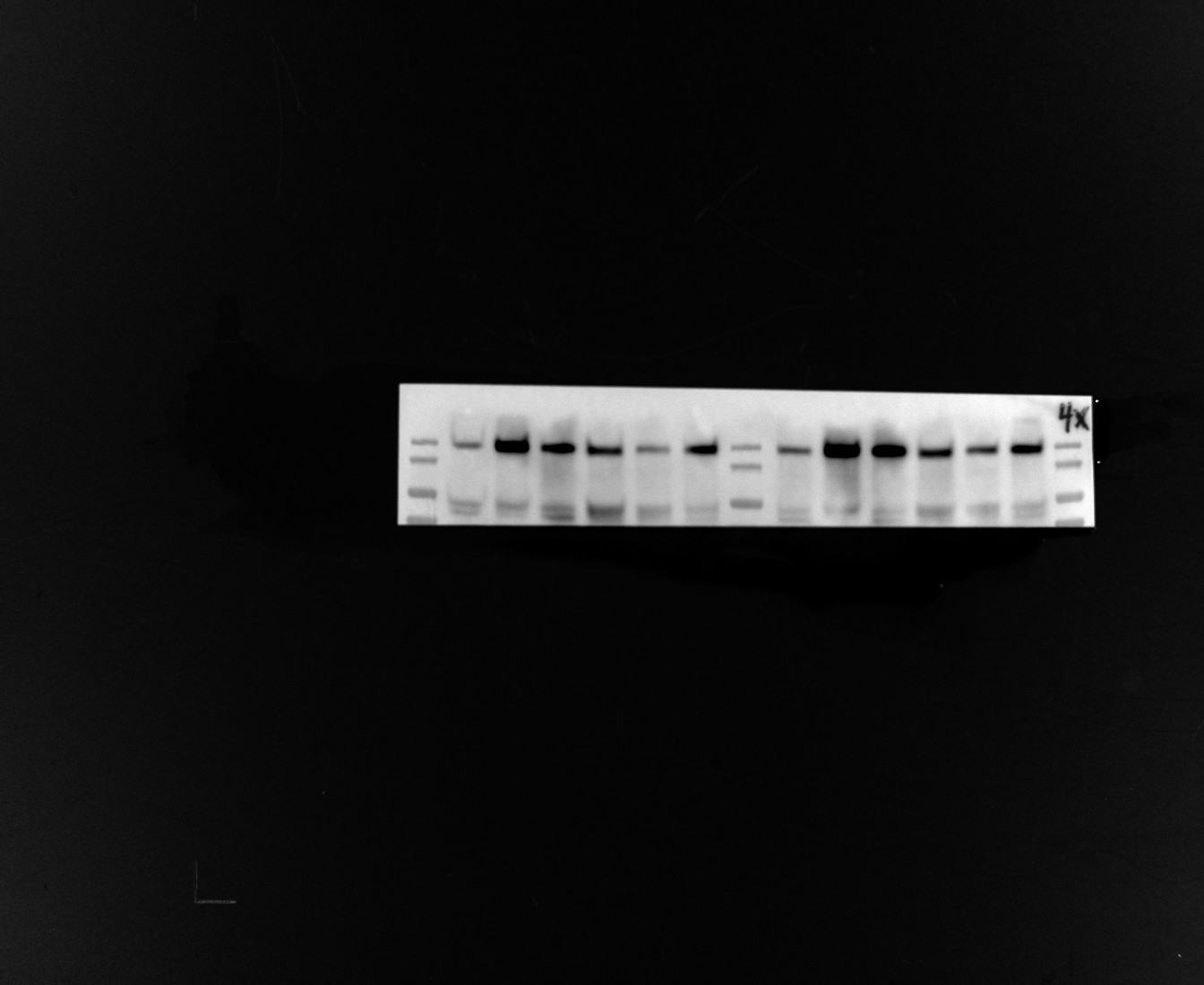

Supplement: Supplementary file 1 [file pharmaceuticals-18-01266-s001.zip › Western blot/AOX/n3-n4 [Overlay][AOX].tif]

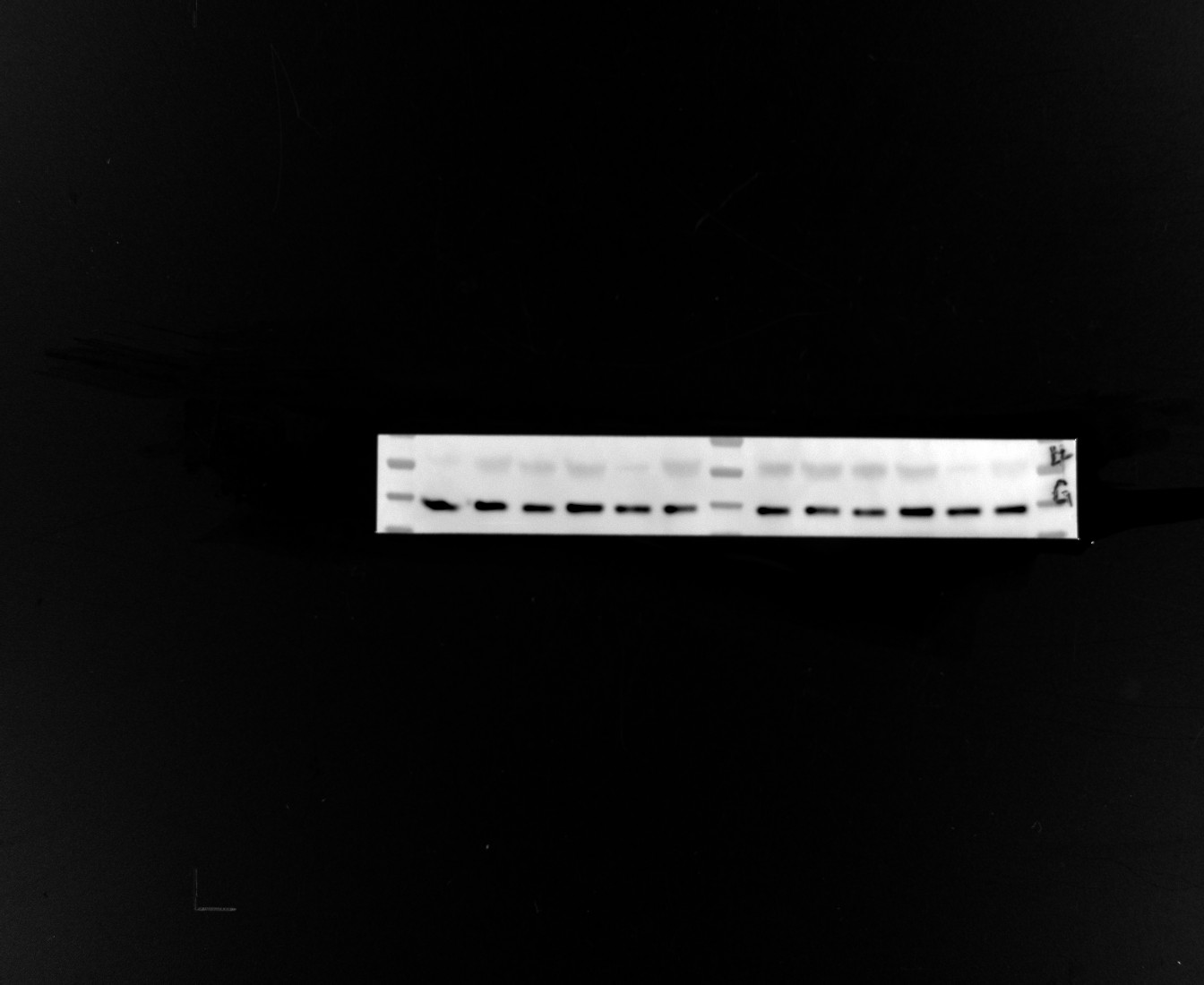

Supplement: Supplementary file 1 [file pharmaceuticals-18-01266-s001.zip › Western blot/AOX/n3-n4 [Overlay][GAPDH].tif]

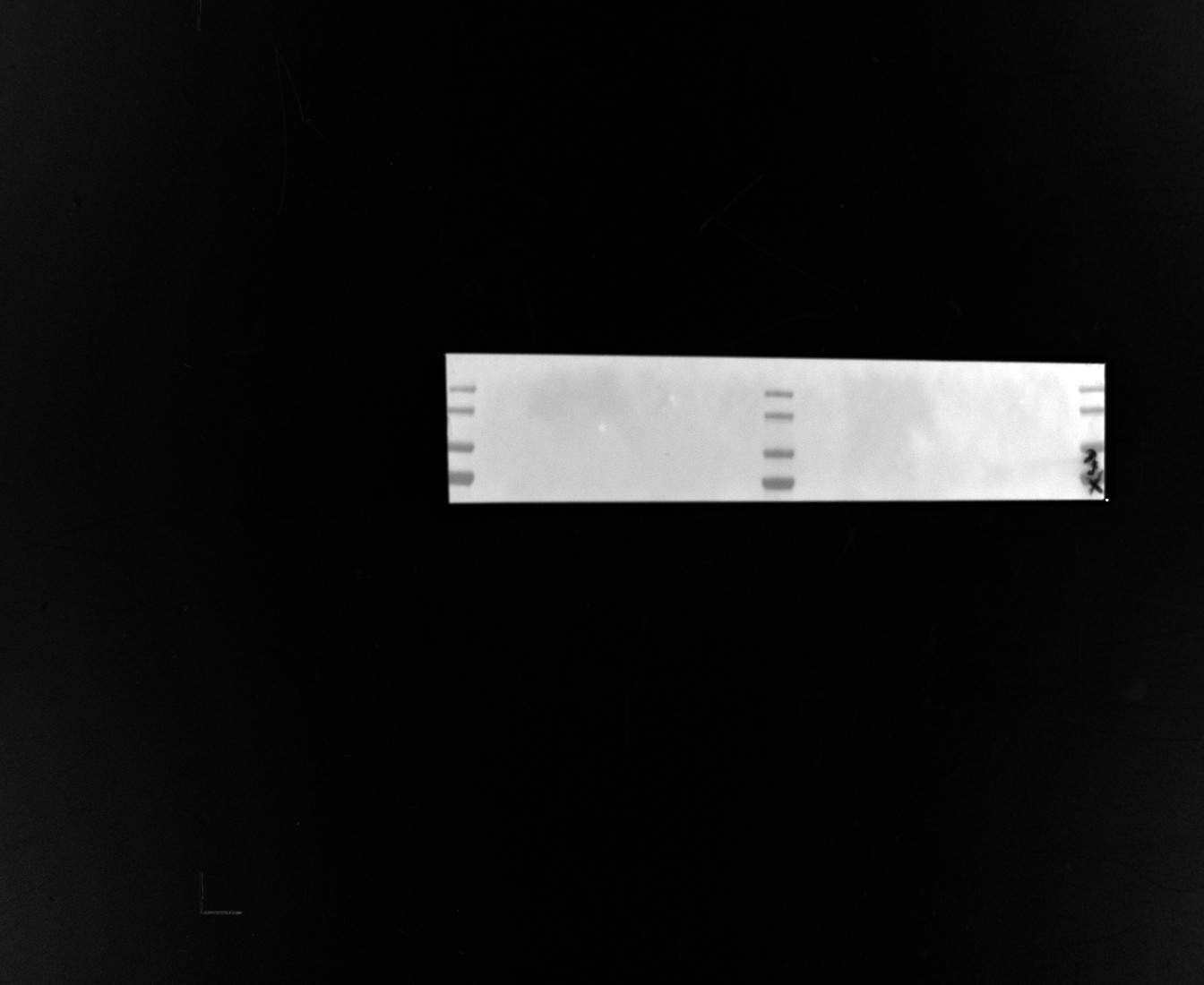

Supplement: Supplementary file 1 [file pharmaceuticals-18-01266-s001.zip › Western blot/AOX/n5-n6 [Brightfield][AOX].tif]

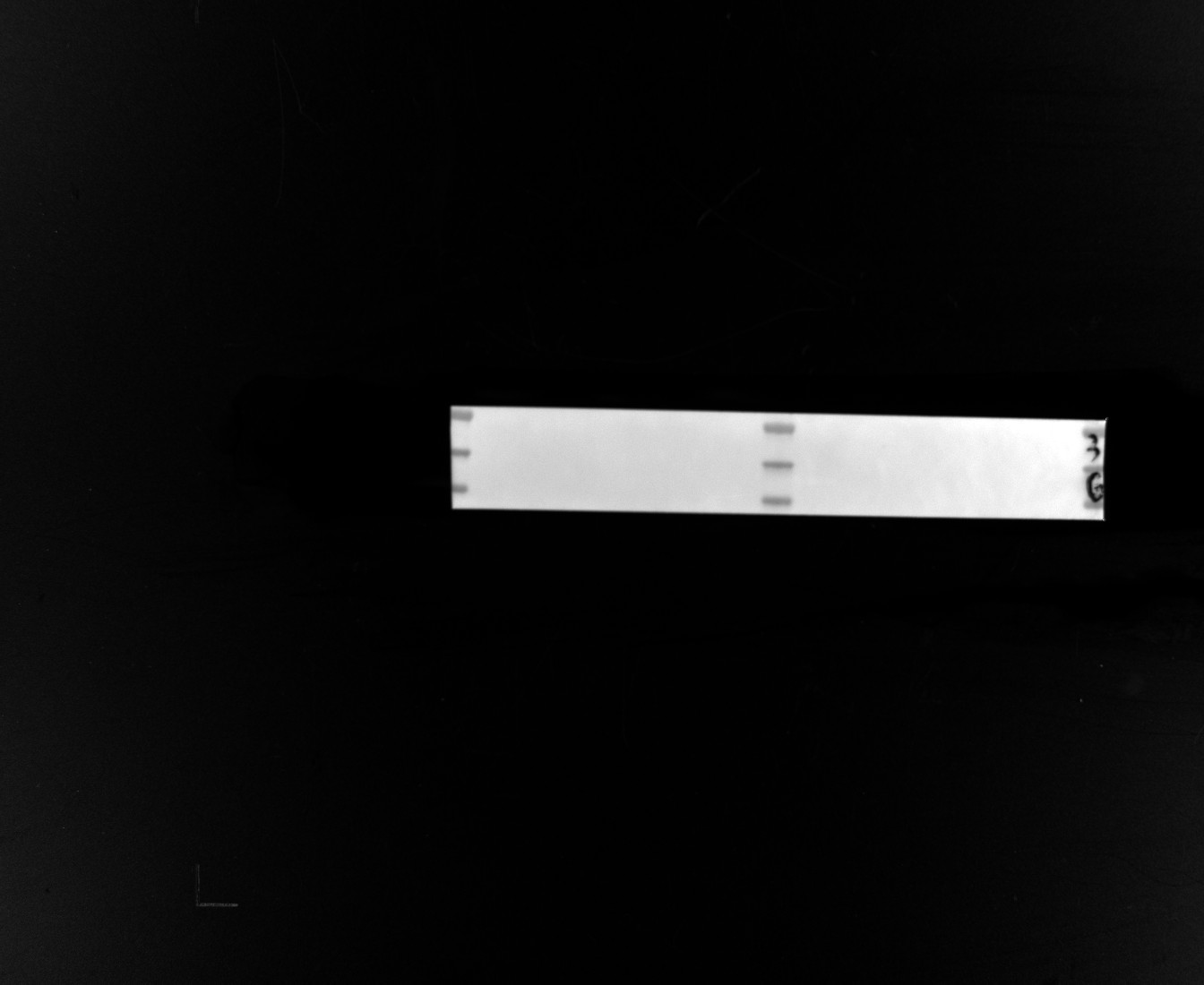

Supplement: Supplementary file 1 [file pharmaceuticals-18-01266-s001.zip › Western blot/AOX/n5-n6 [Brightfield][GAPDH].tif]

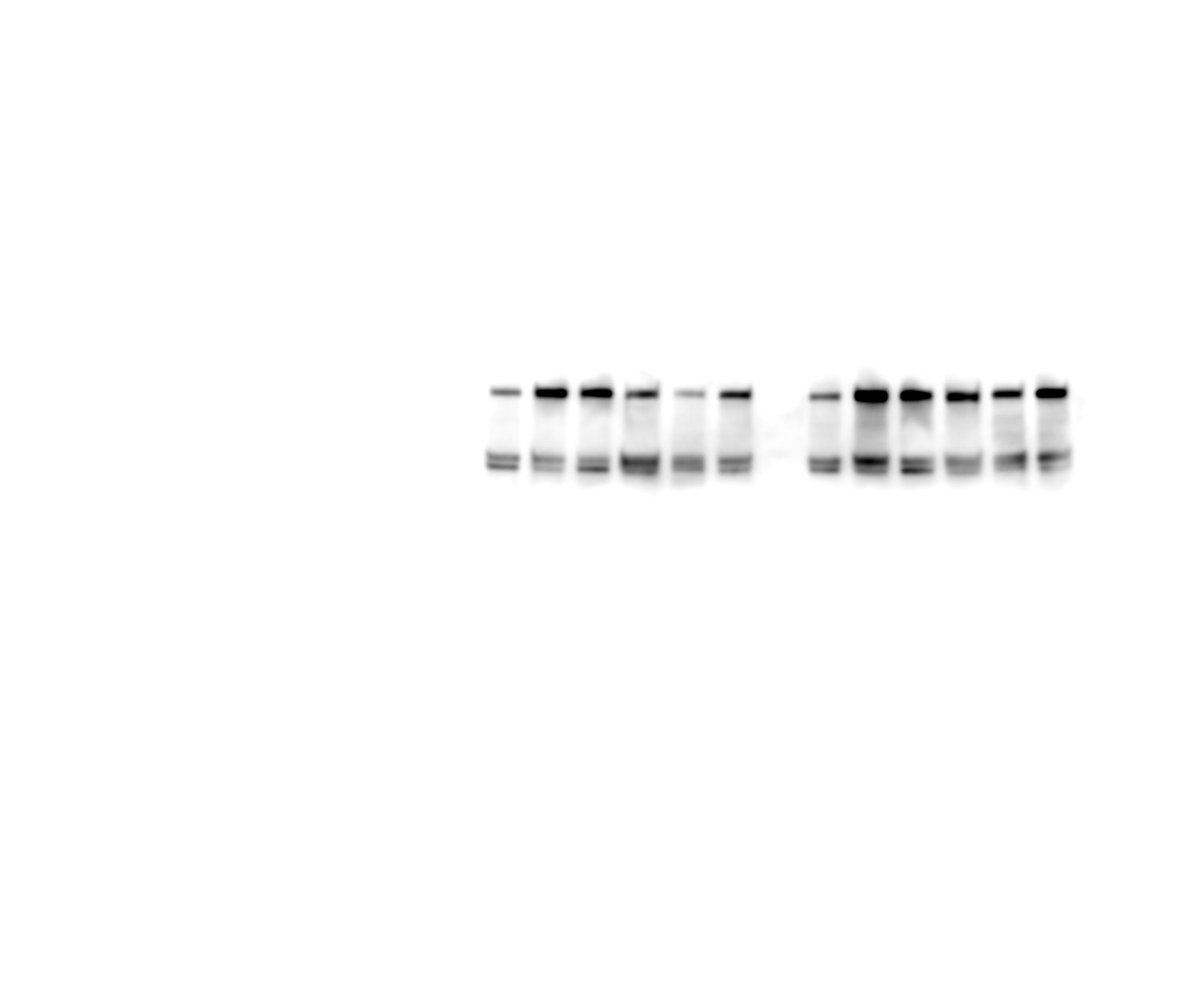

Supplement: Supplementary file 1 [file pharmaceuticals-18-01266-s001.zip › Western blot/AOX/n5-n6 [Luminescence][AOX].tif]

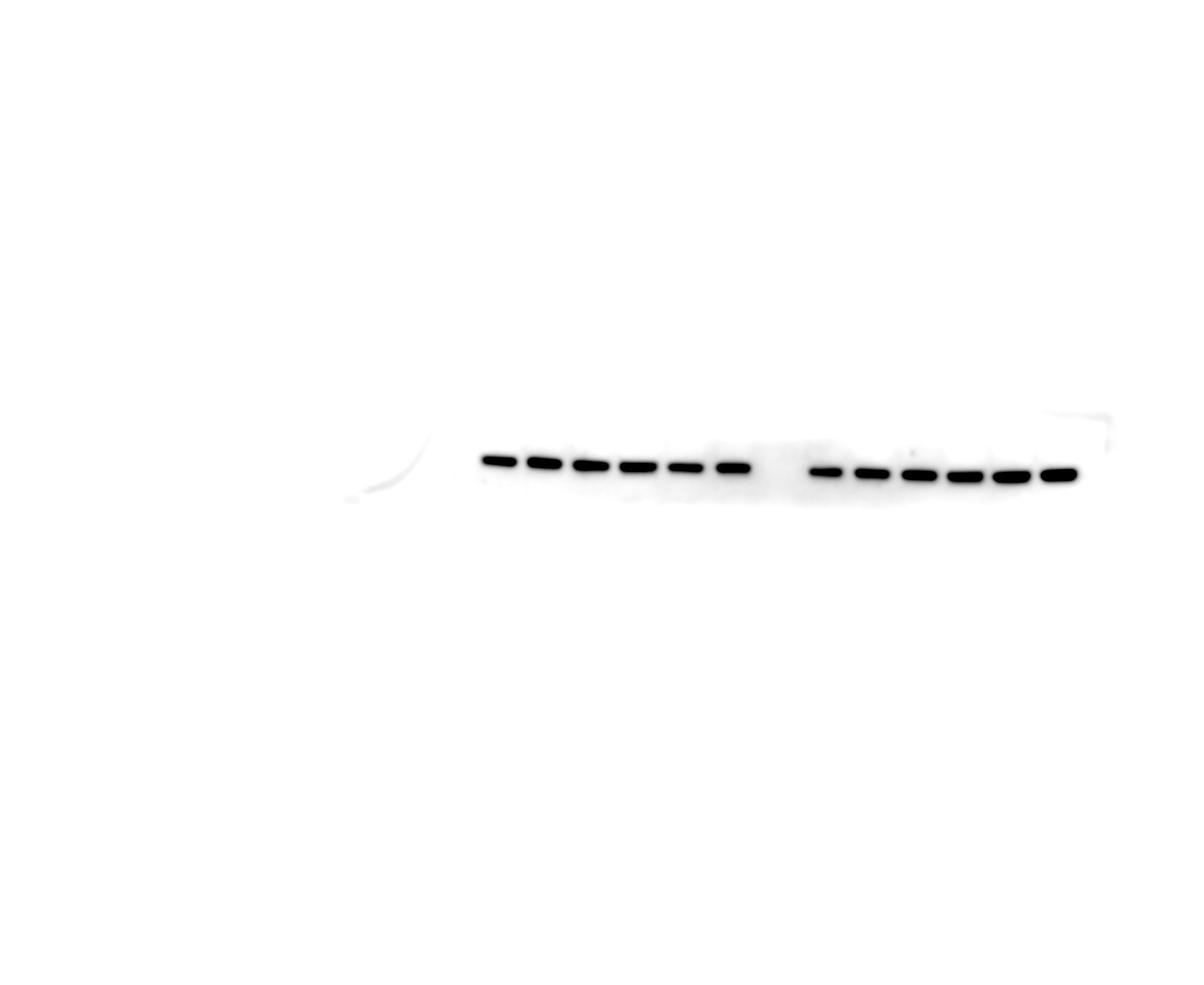

Supplement: Supplementary file 1 [file pharmaceuticals-18-01266-s001.zip › Western blot/AOX/n5-n6 [Luminescence][GAPDH].tif]

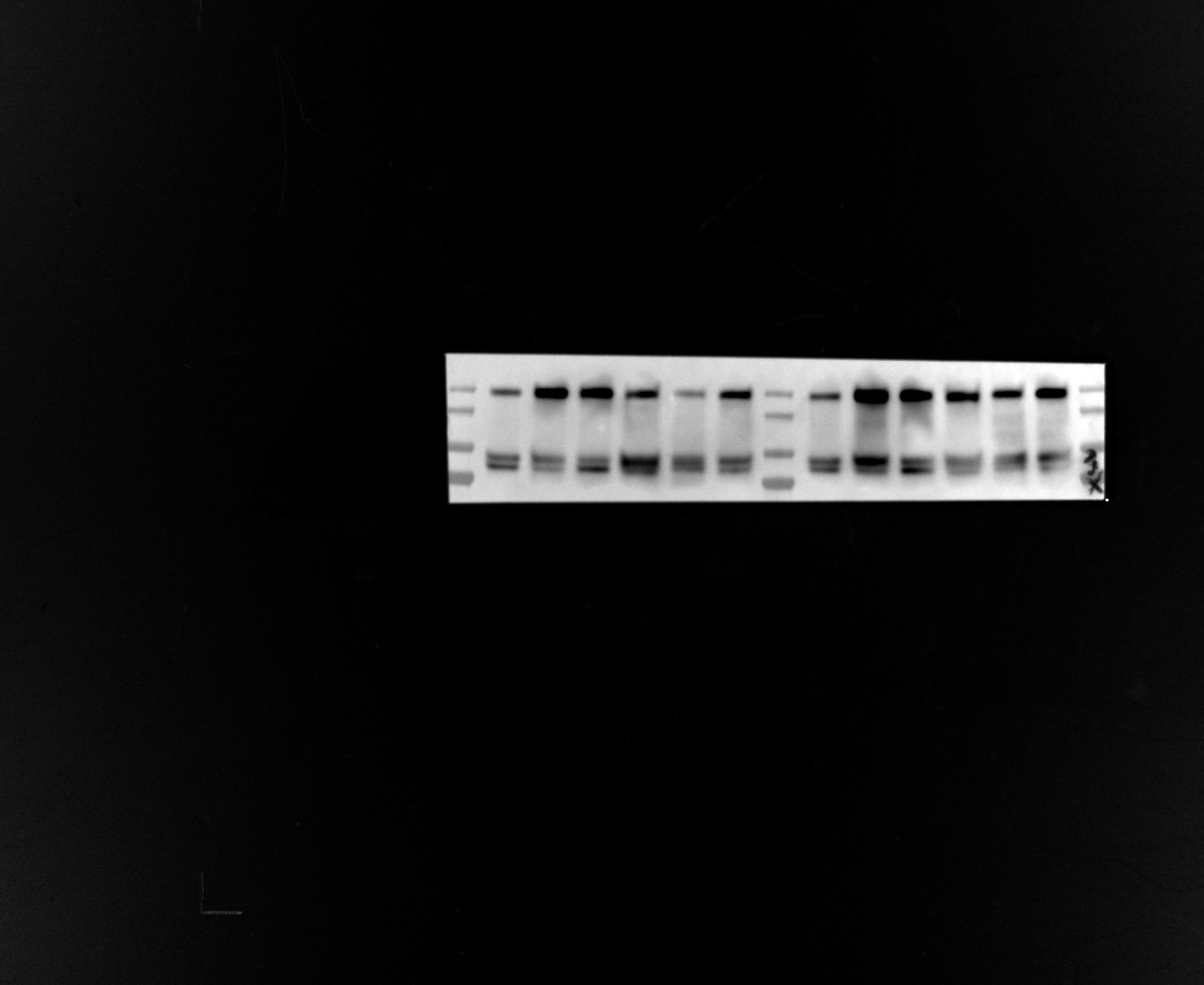

Supplement: Supplementary file 1 [file pharmaceuticals-18-01266-s001.zip › Western blot/AOX/n5-n6 [Overlay][AOX].tif]

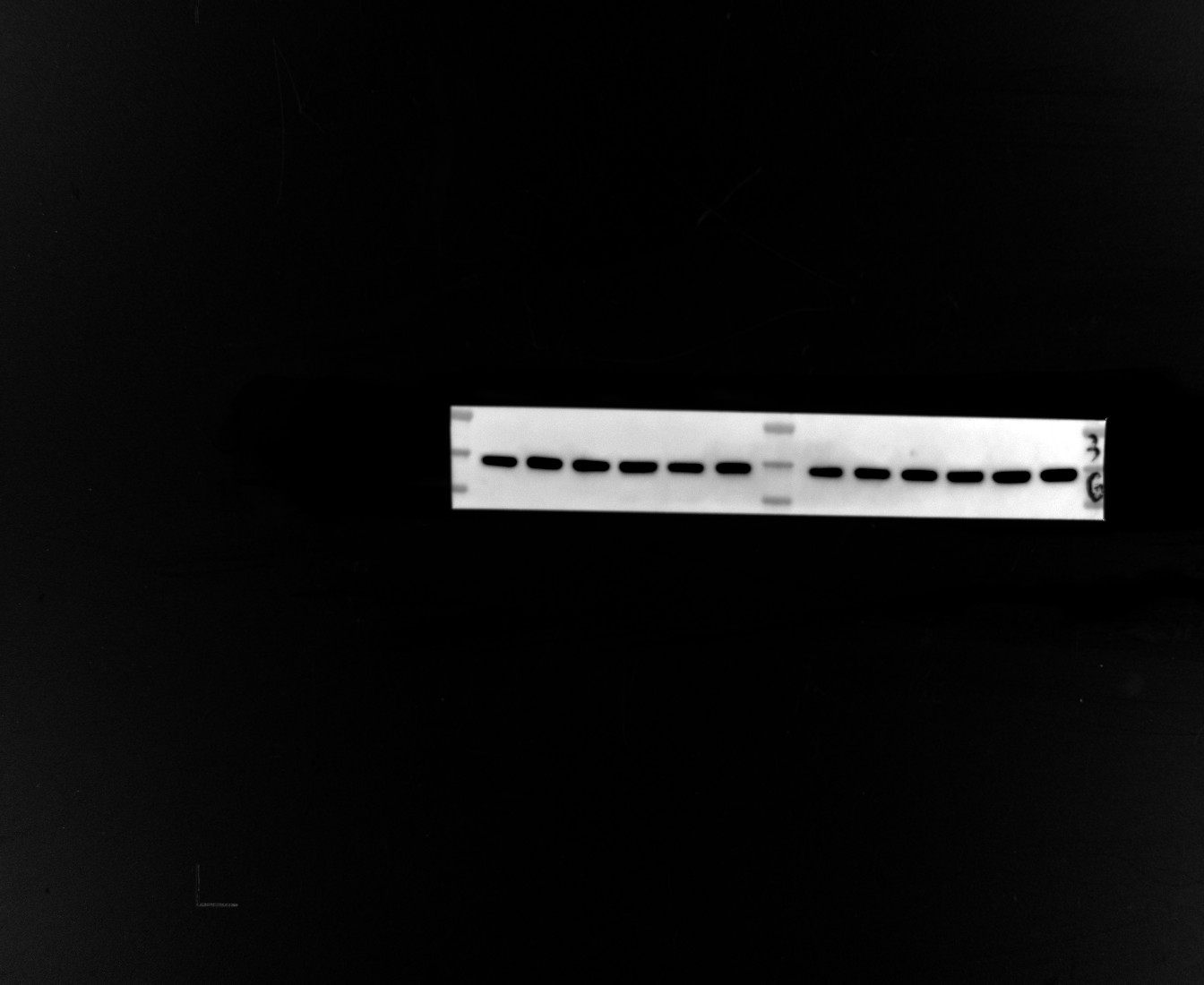

Supplement: Supplementary file 1 [file pharmaceuticals-18-01266-s001.zip › Western blot/AOX/n5-n6 [Overlay][GAPDH].tif]

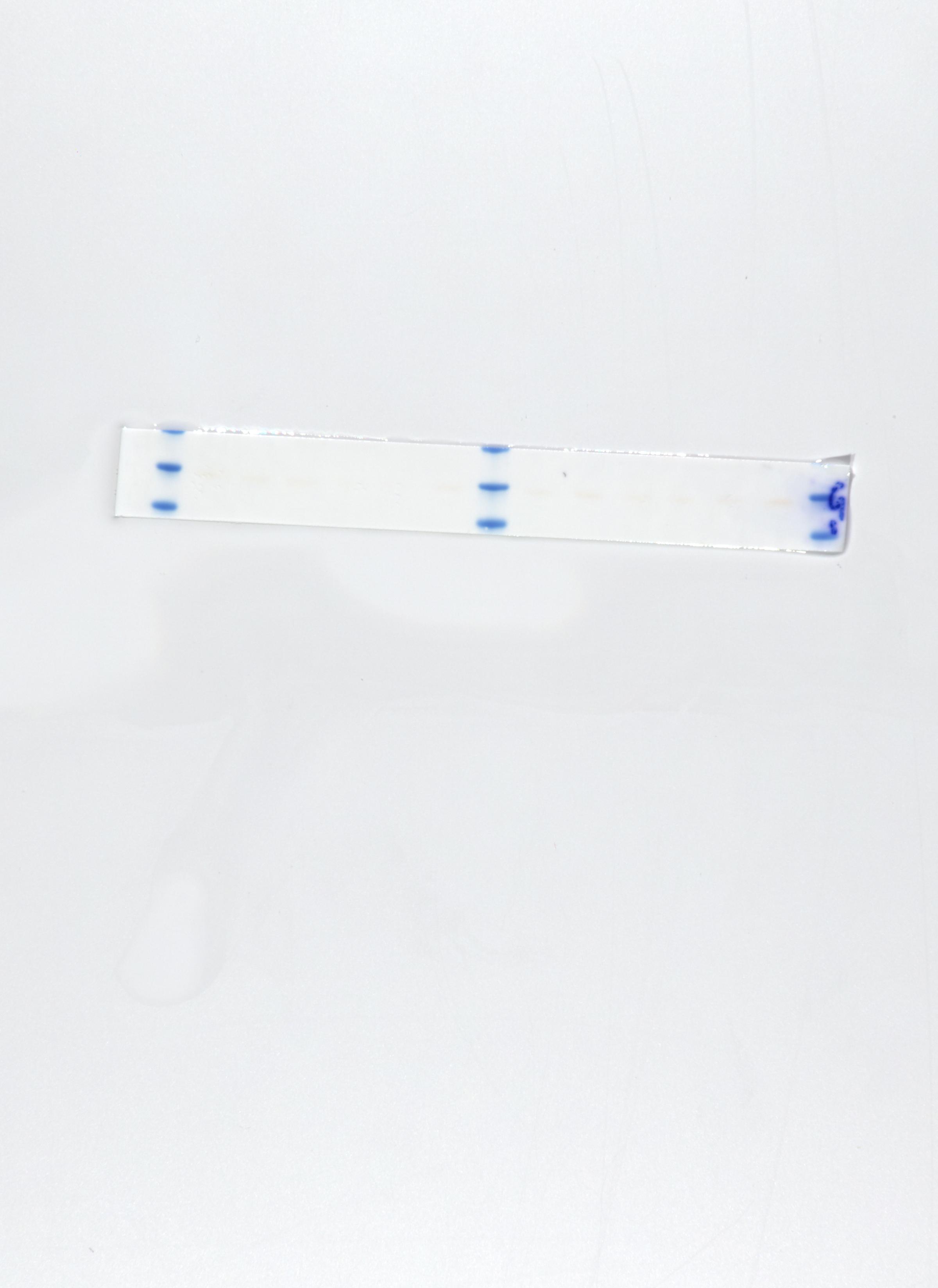

Supplement: Supplementary file 1 [file pharmaceuticals-18-01266-s001.zip › Western blot/IFNG/n1-n2 [Brightfield][GAPDH].jpg]

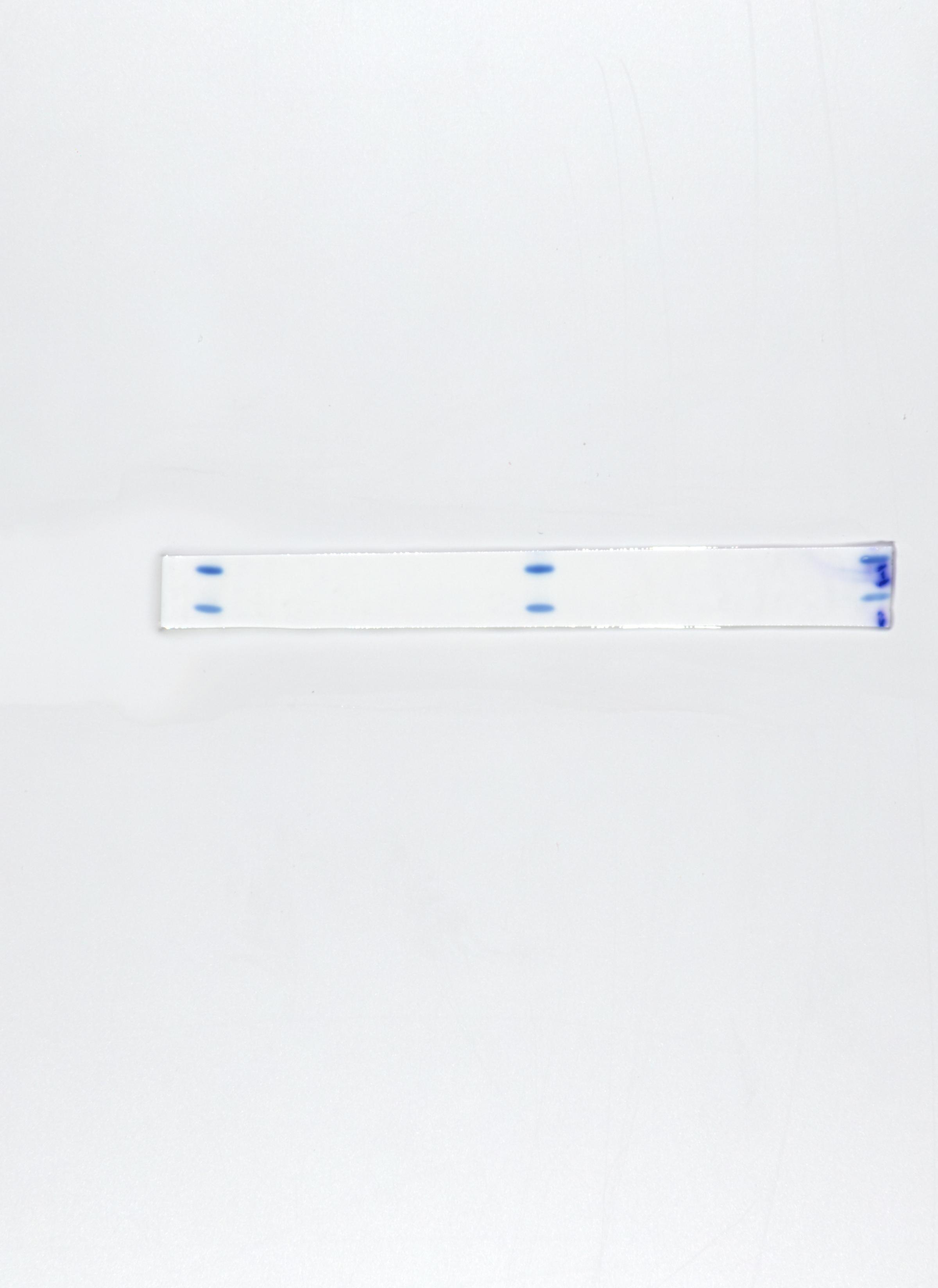

Supplement: Supplementary file 1 [file pharmaceuticals-18-01266-s001.zip › Western blot/IFNG/n1-n2 [Brightfield][IFNG].jpg]

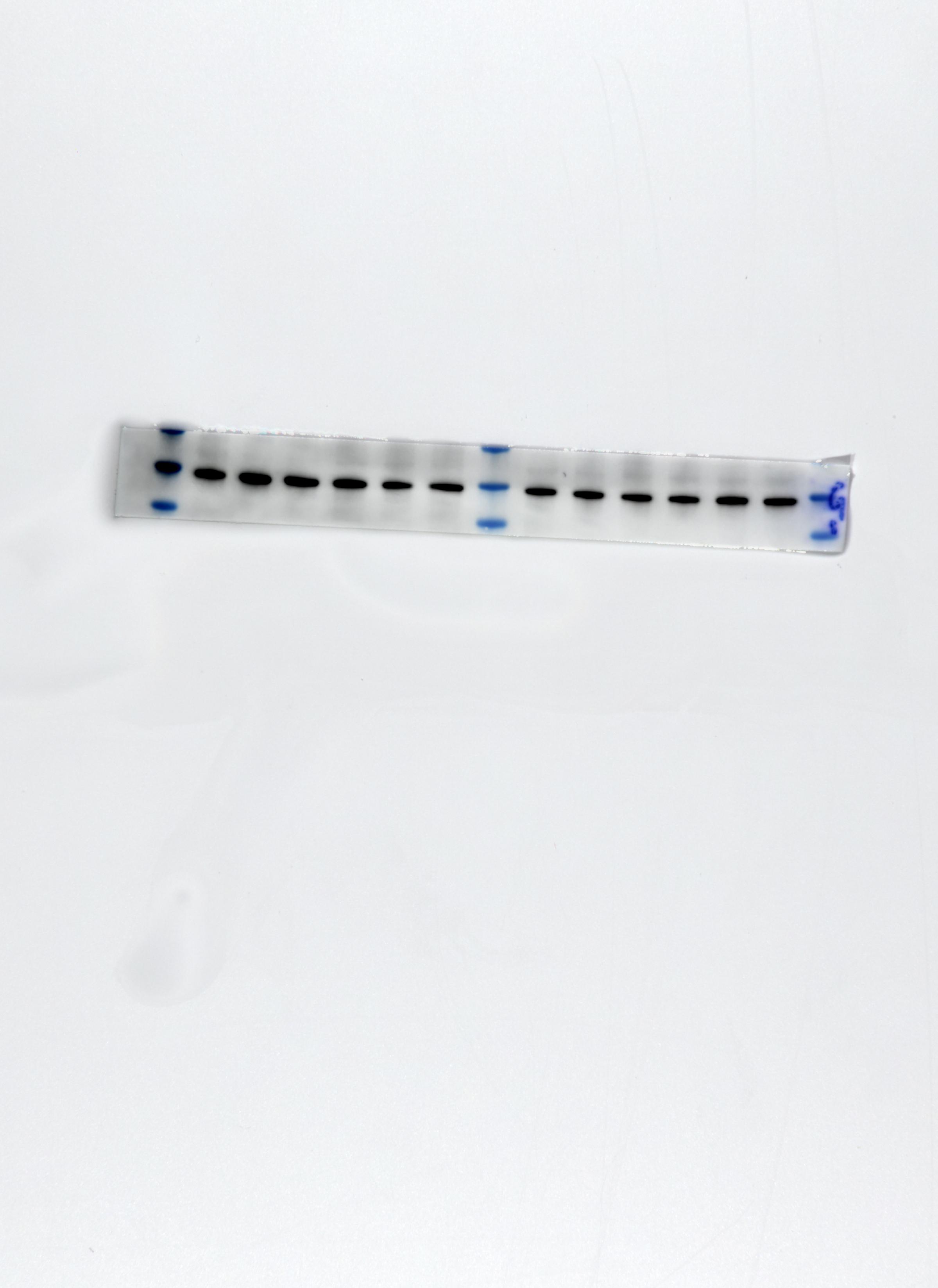

Supplement: Supplementary file 1 [file pharmaceuticals-18-01266-s001.zip › Western blot/IFNG/n1-n2 [Overlay][GAPDH].jpg]

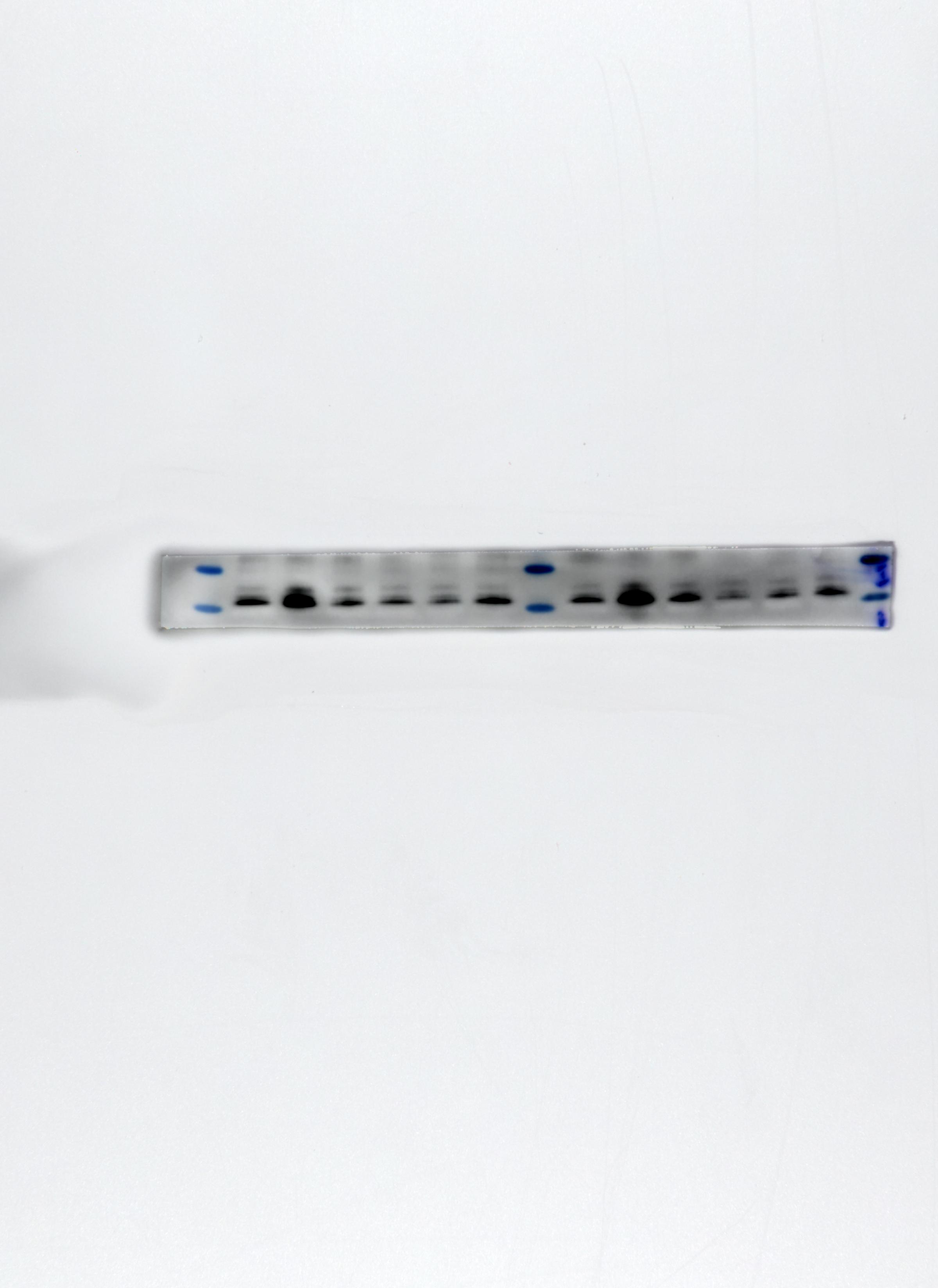

Supplement: Supplementary file 1 [file pharmaceuticals-18-01266-s001.zip › Western blot/IFNG/n1-n2 [Overlay][IFNG].jpg]

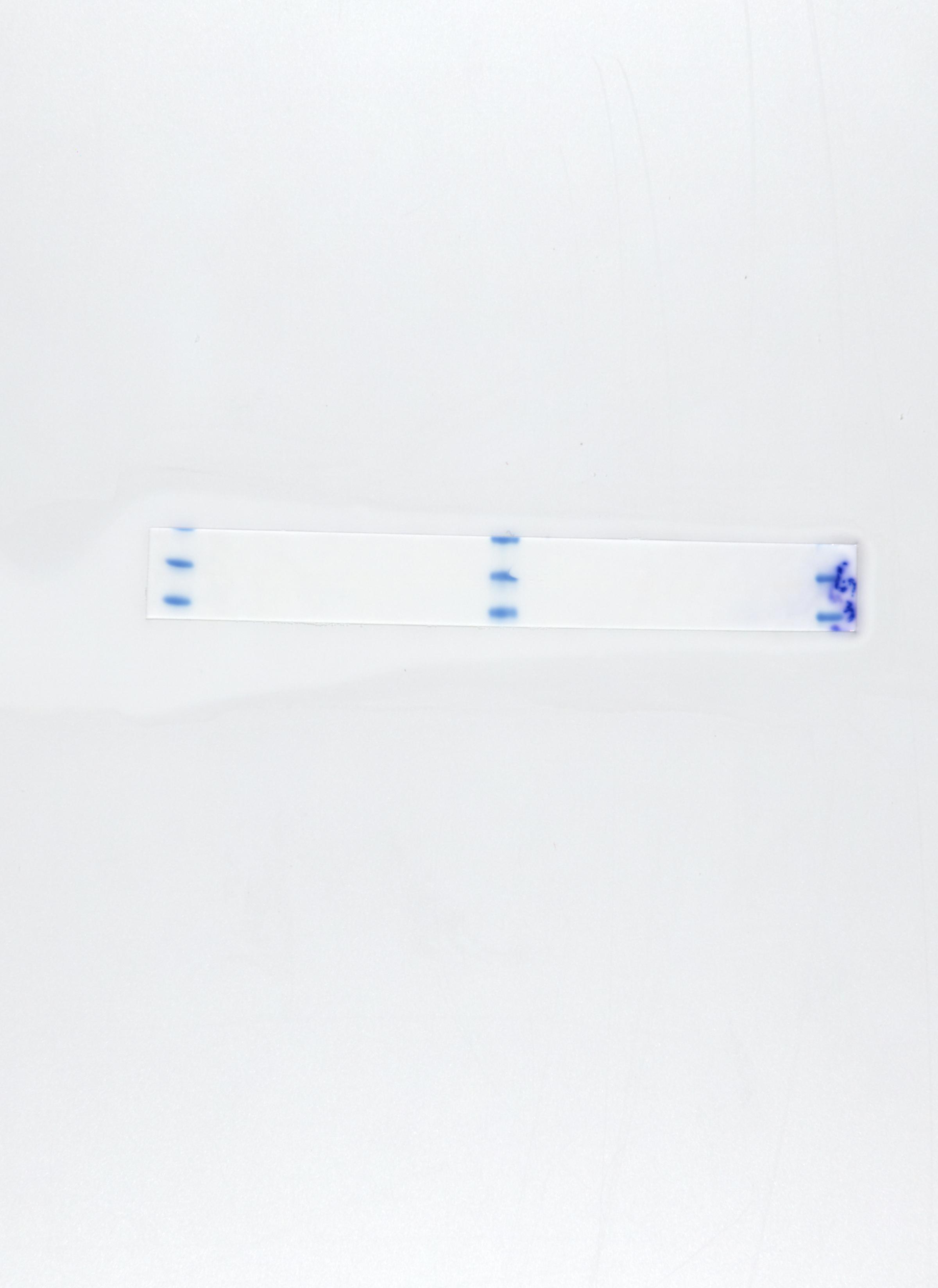

Supplement: Supplementary file 1 [file pharmaceuticals-18-01266-s001.zip › Western blot/IFNG/n3-n4 [Brightfield][GAPDH].jpg]

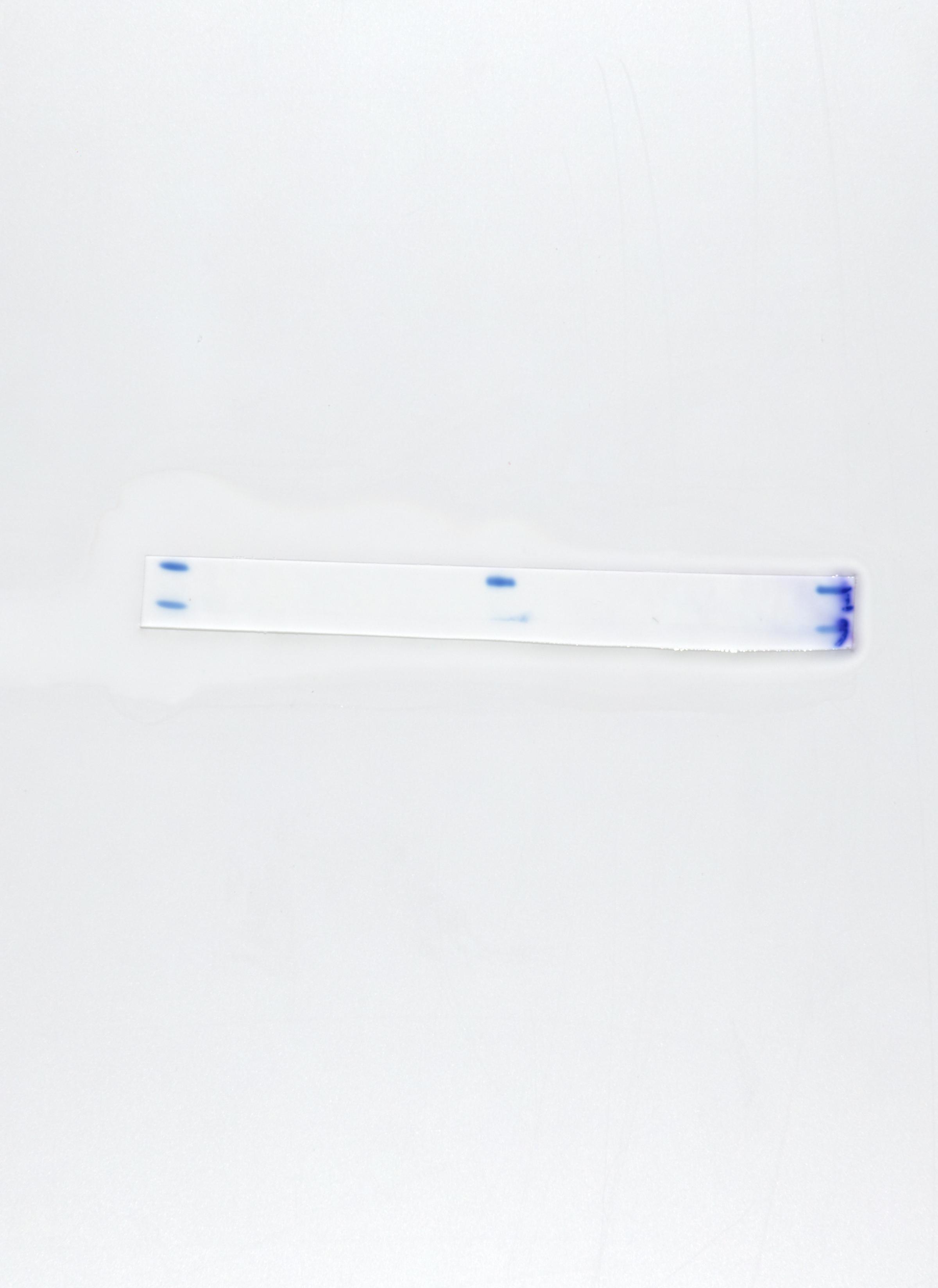

Supplement: Supplementary file 1 [file pharmaceuticals-18-01266-s001.zip › Western blot/IFNG/n3-n4 [Brightfield][IFNG].jpg]

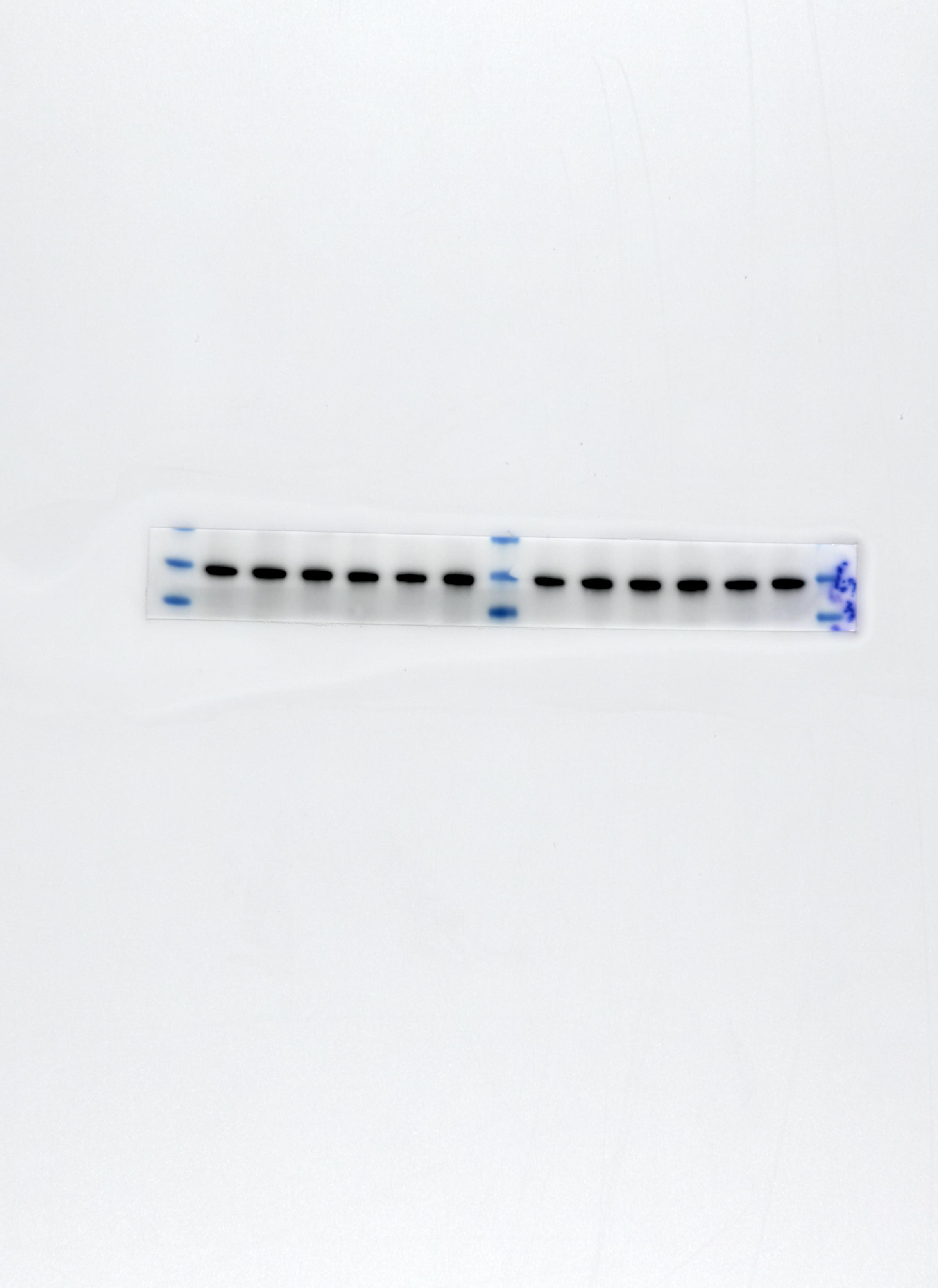

Supplement: Supplementary file 1 [file pharmaceuticals-18-01266-s001.zip › Western blot/IFNG/n3-n4 [Overlay][GAPDH].jpg]

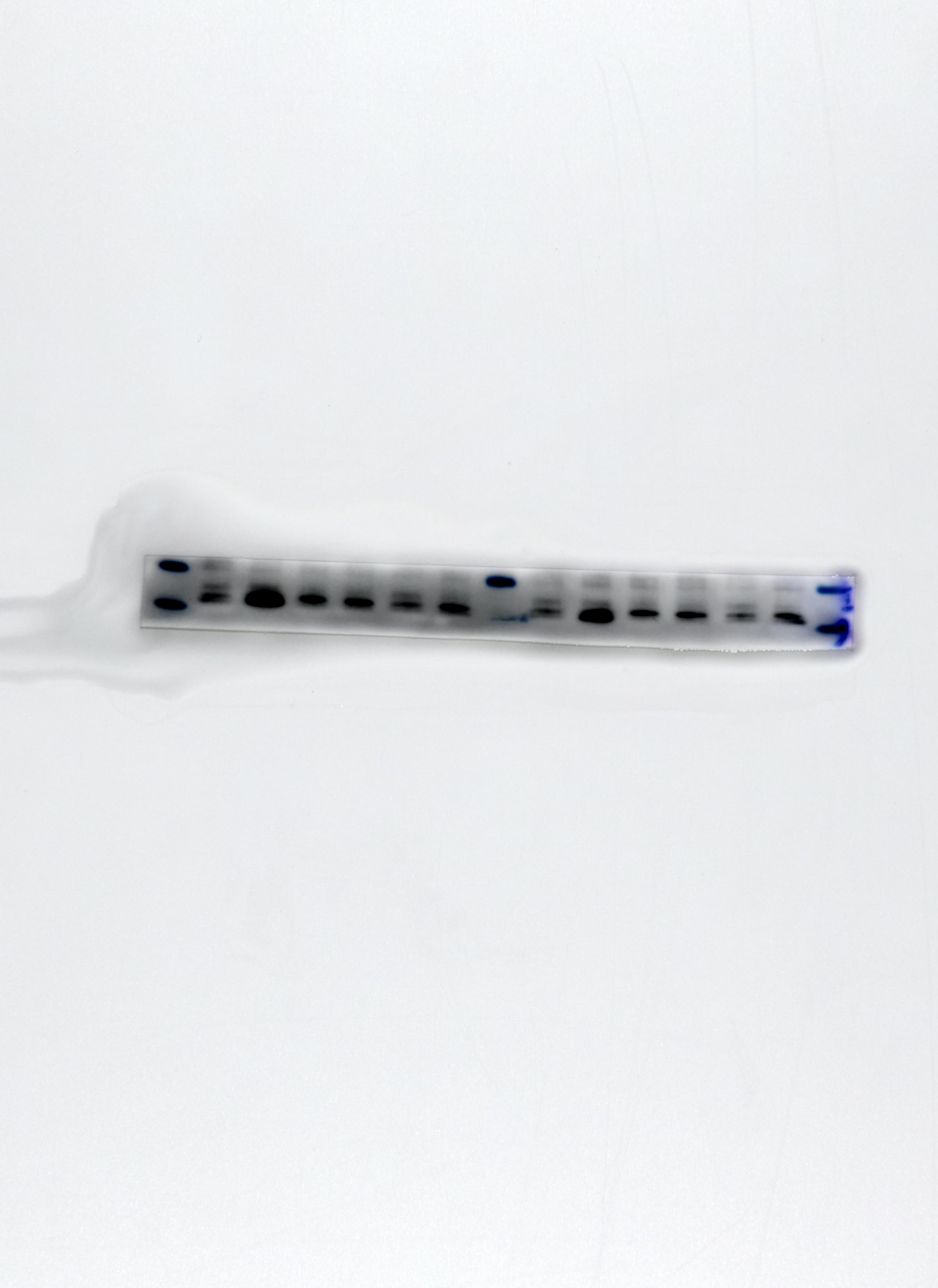

Supplement: Supplementary file 1 [file pharmaceuticals-18-01266-s001.zip › Western blot/IFNG/n3-n4 [Overlay][IFNG].jpg]

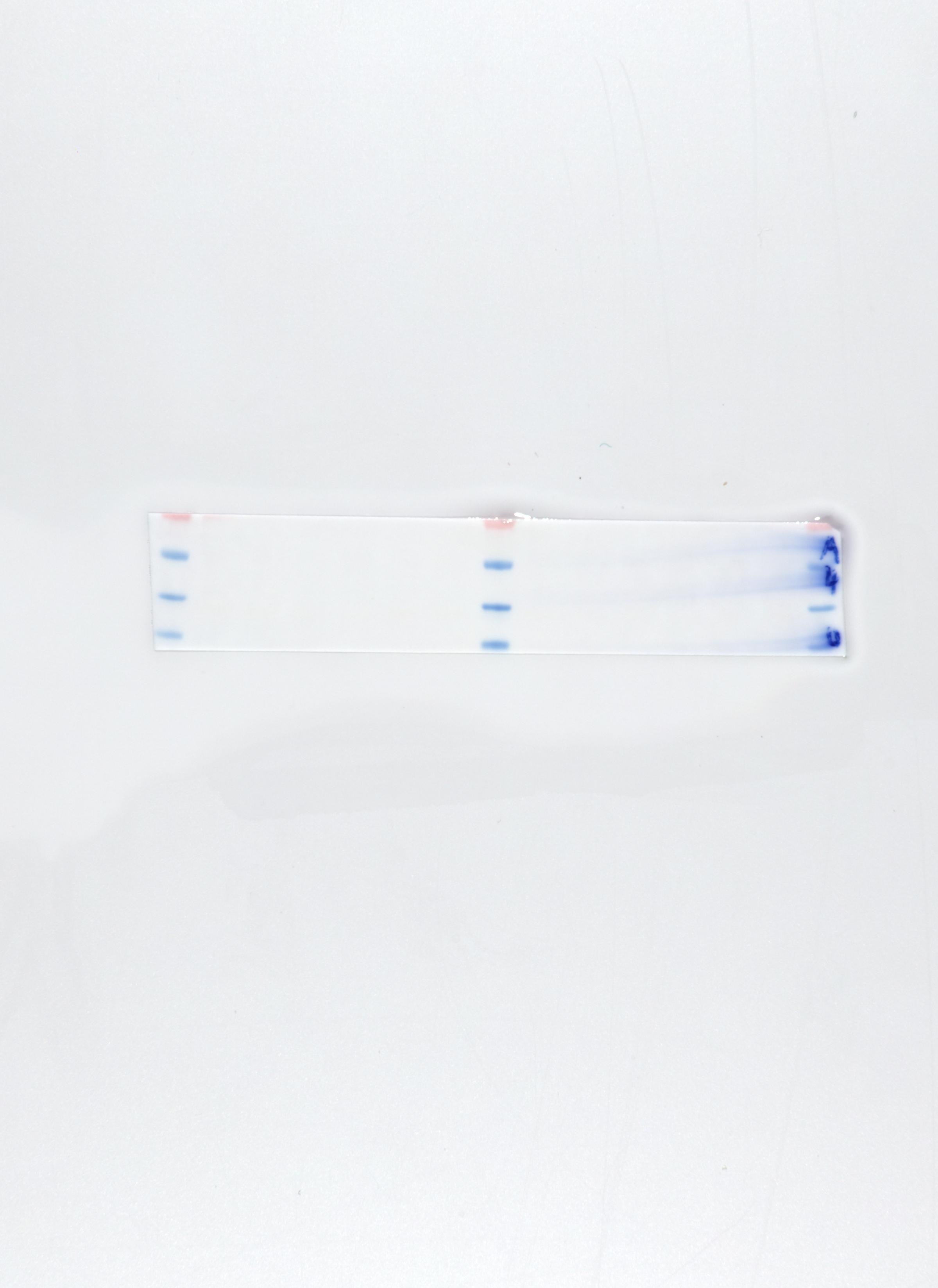

Supplement: Supplementary file 1 [file pharmaceuticals-18-01266-s001.zip › Western blot/IFNG/n5-n6 [Brightfield][GAPDH].jpg.jpg]

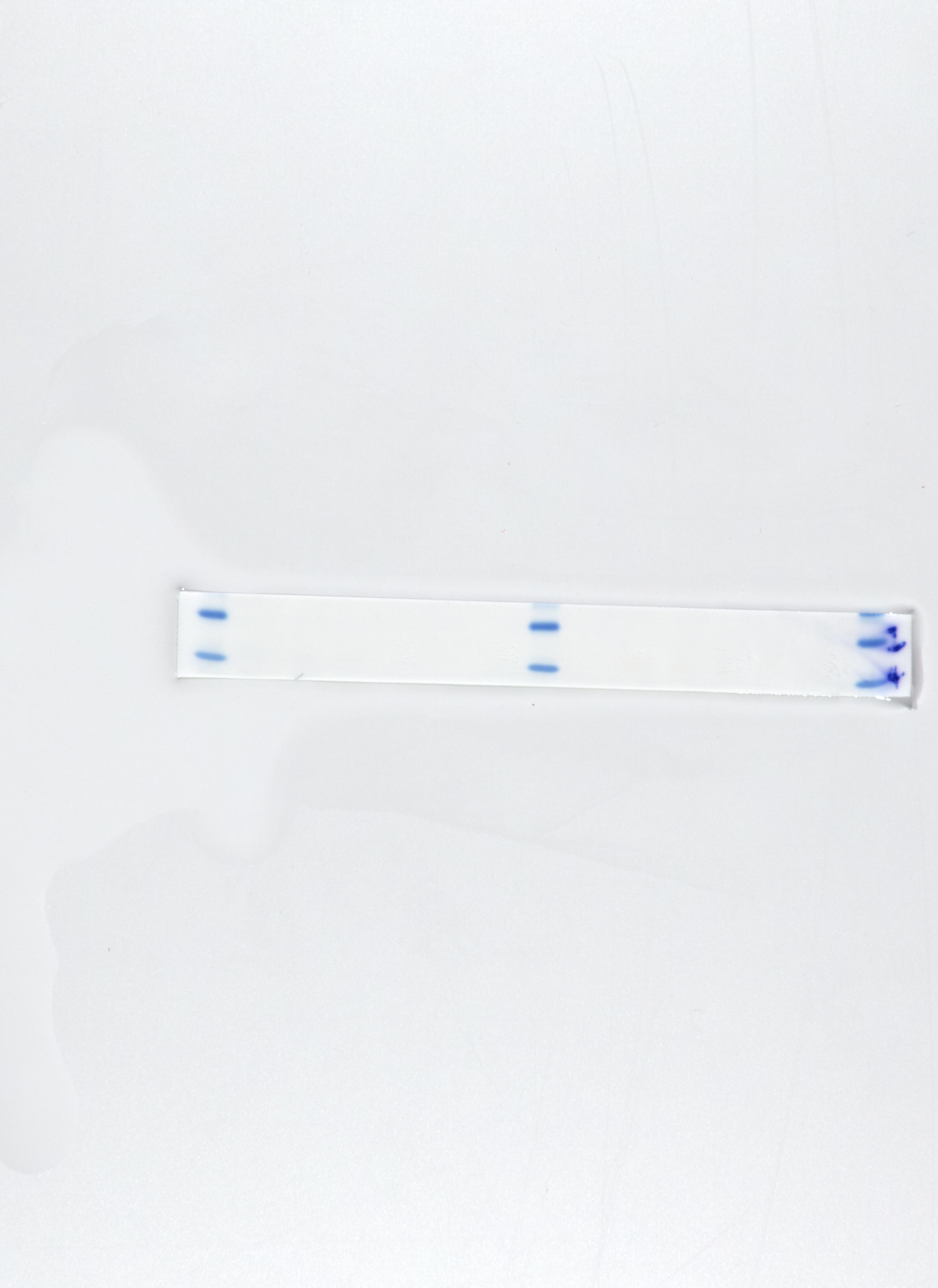

Supplement: Supplementary file 1 [file pharmaceuticals-18-01266-s001.zip › Western blot/IFNG/n5-n6 [Brightfield][IFNG].jpg]

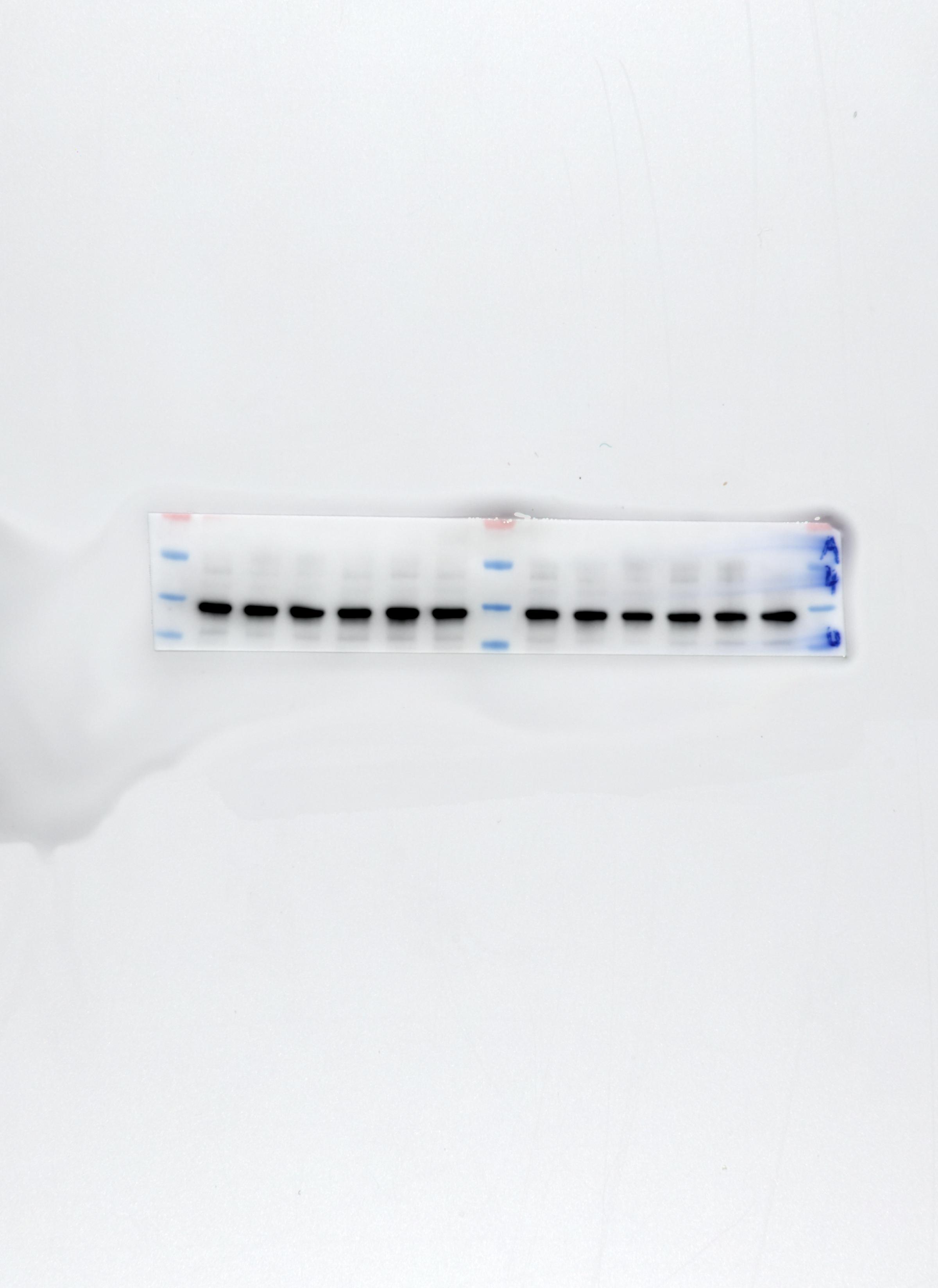

Supplement: Supplementary file 1 [file pharmaceuticals-18-01266-s001.zip › Western blot/IFNG/n5-n6 [Overlay][GAPDH].jpg]

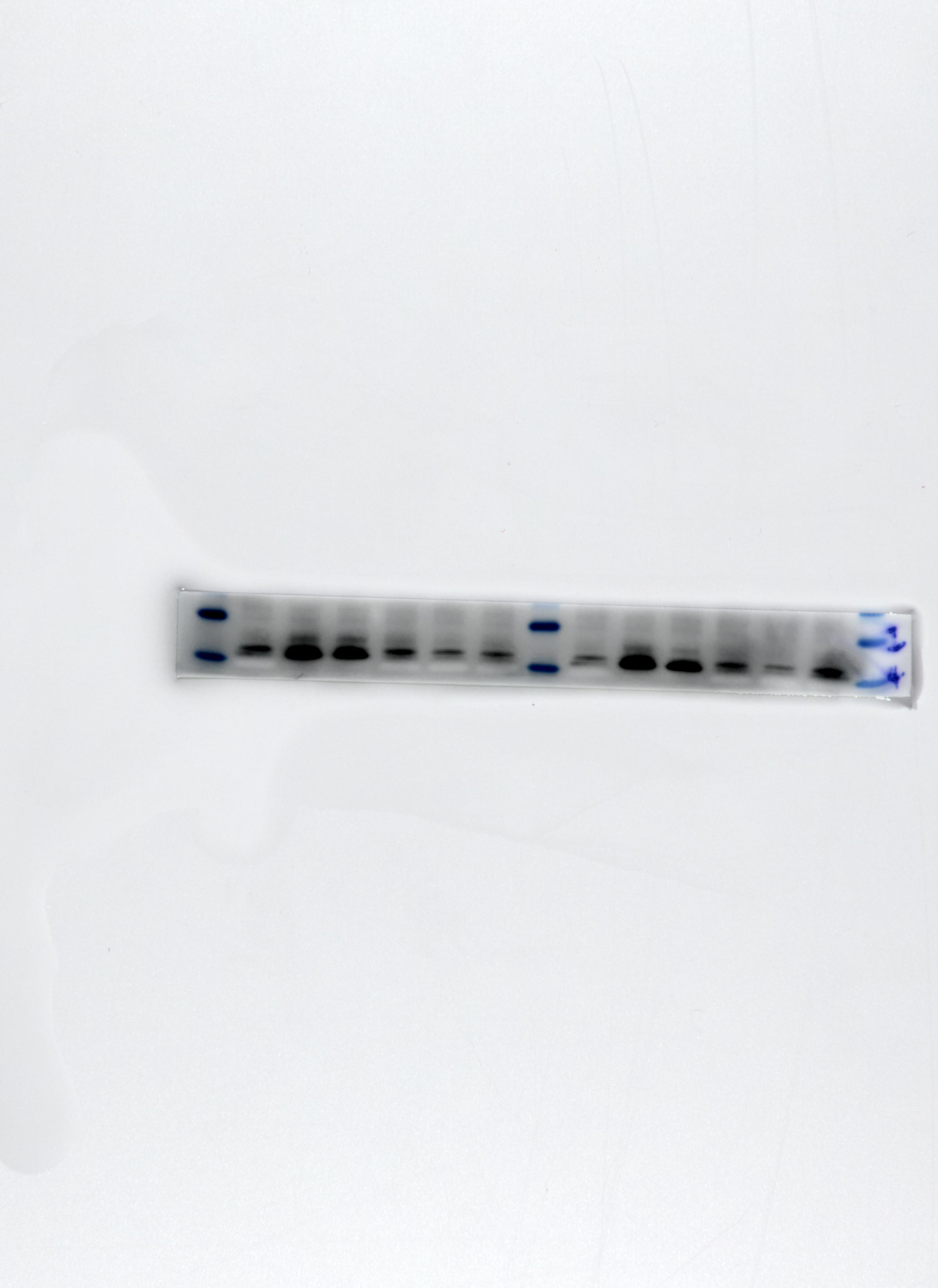

Supplement: Supplementary file 1 [file pharmaceuticals-18-01266-s001.zip › Western blot/IFNG/n5-n6 [Overlay][IFNG].tif.jpg]

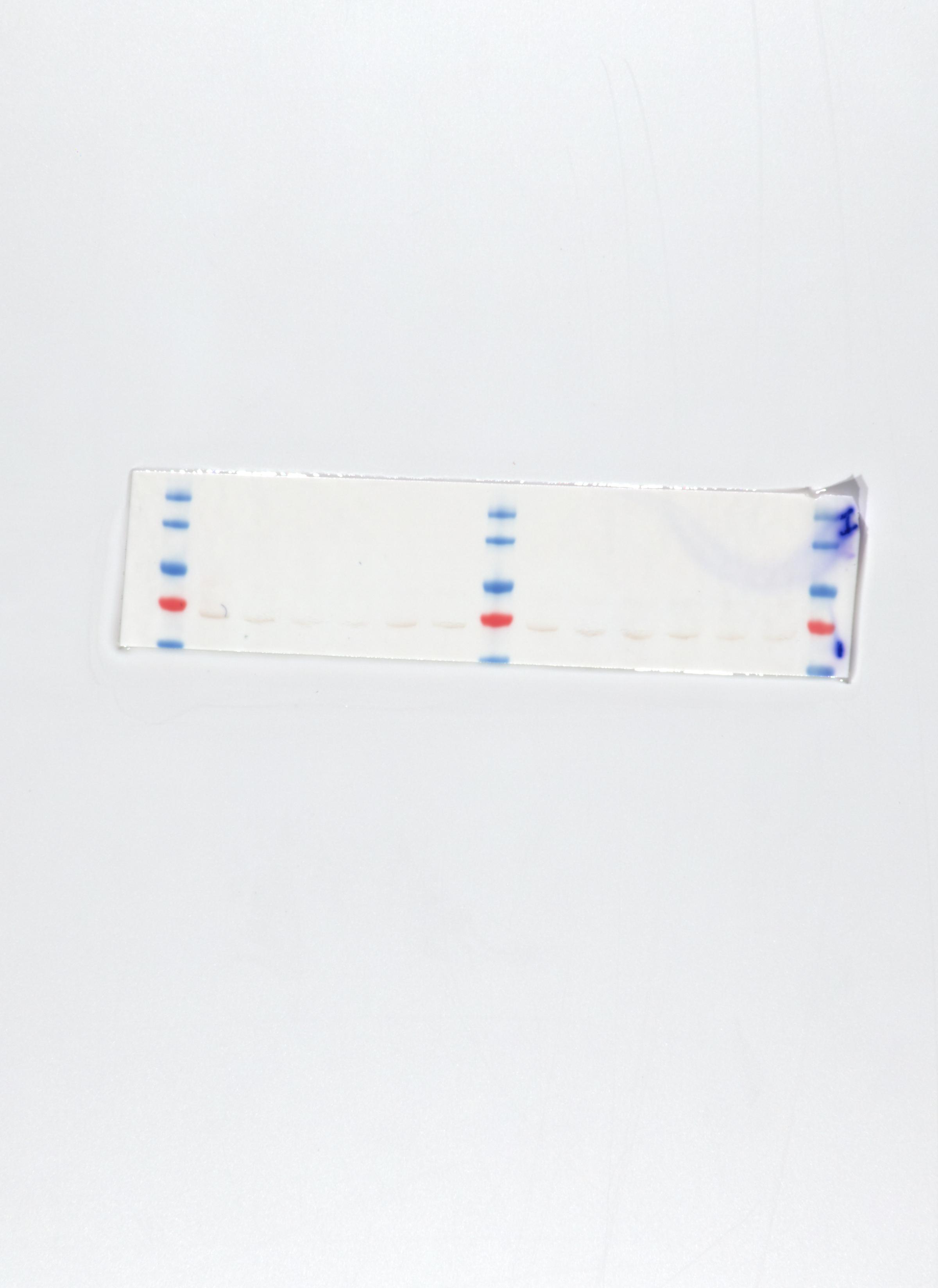

Supplement: Supplementary file 1 [file pharmaceuticals-18-01266-s001.zip › Western blot/IFNGR1/n1-n2 [Brightfield][IFNGR1].jpg]

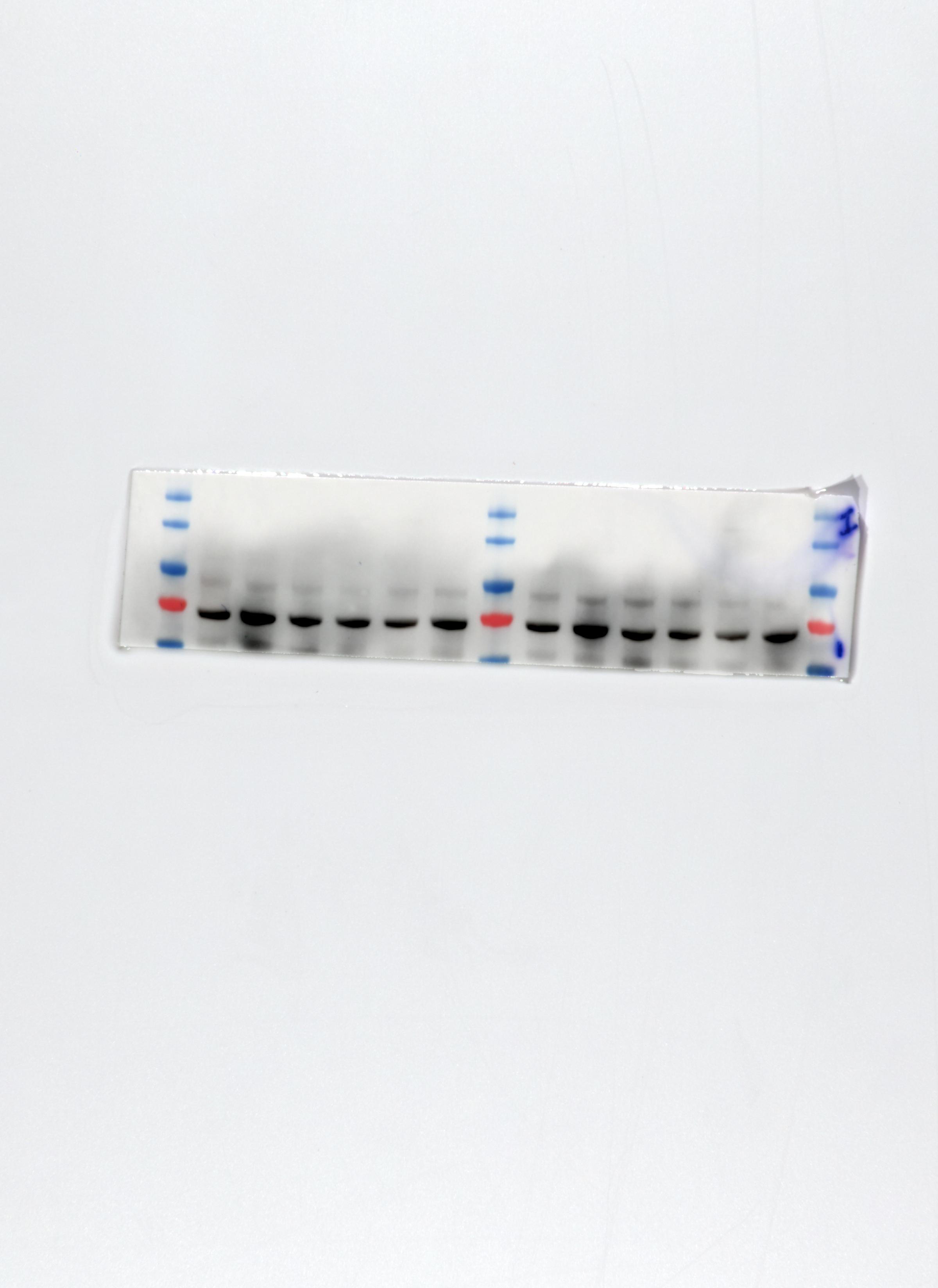

Supplement: Supplementary file 1 [file pharmaceuticals-18-01266-s001.zip › Western blot/IFNGR1/n1-n2 [Overlay][IFNGR1].jpg]

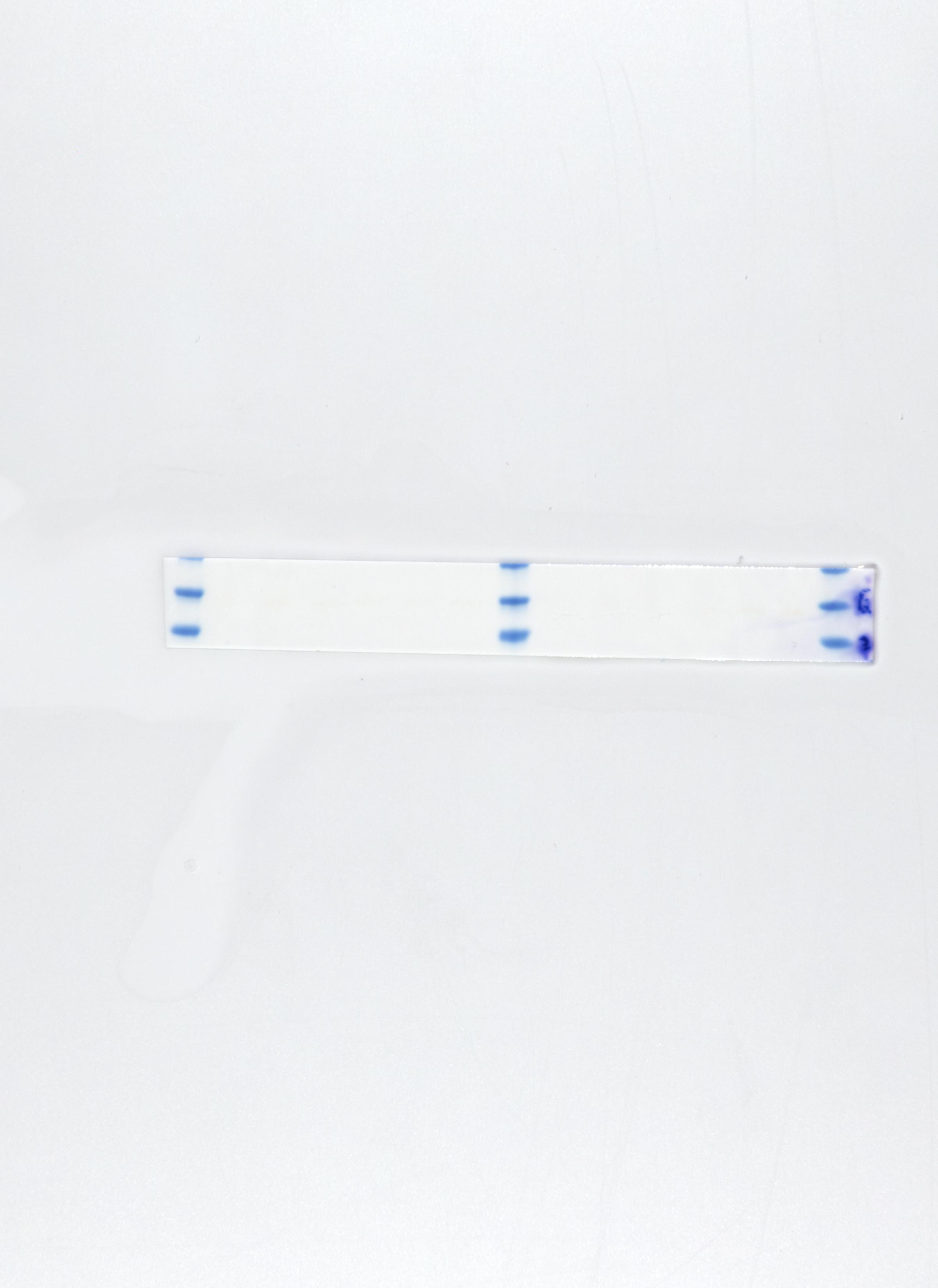

Supplement: Supplementary file 1 [file pharmaceuticals-18-01266-s001.zip › Western blot/IFNGR1/n3(R) [Brightfield][GAPDH]-R.jpg]

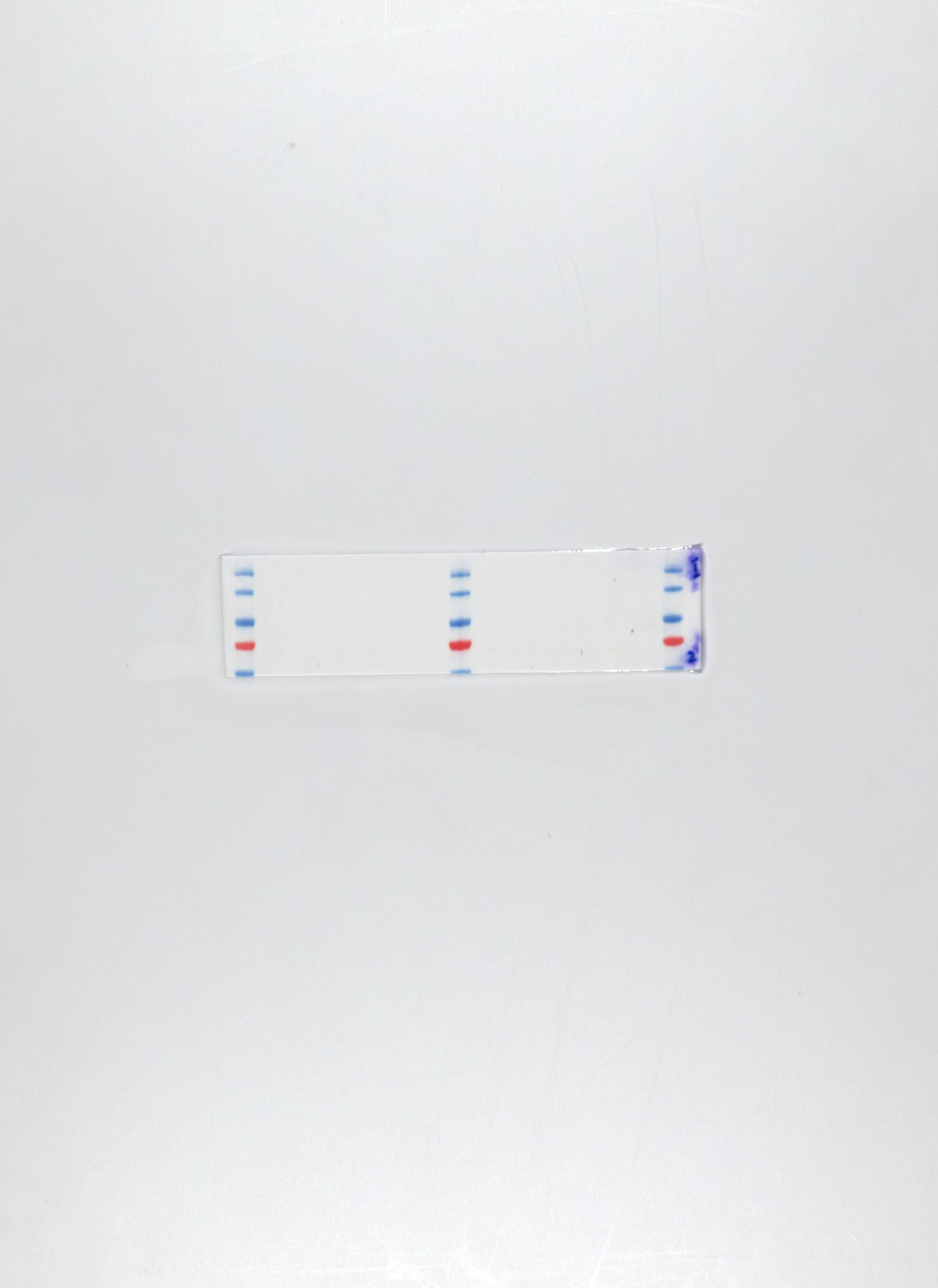

Supplement: Supplementary file 1 [file pharmaceuticals-18-01266-s001.zip › Western blot/IFNGR1/n3(R) [Brightfield][IFNGR2]-R.jpg]

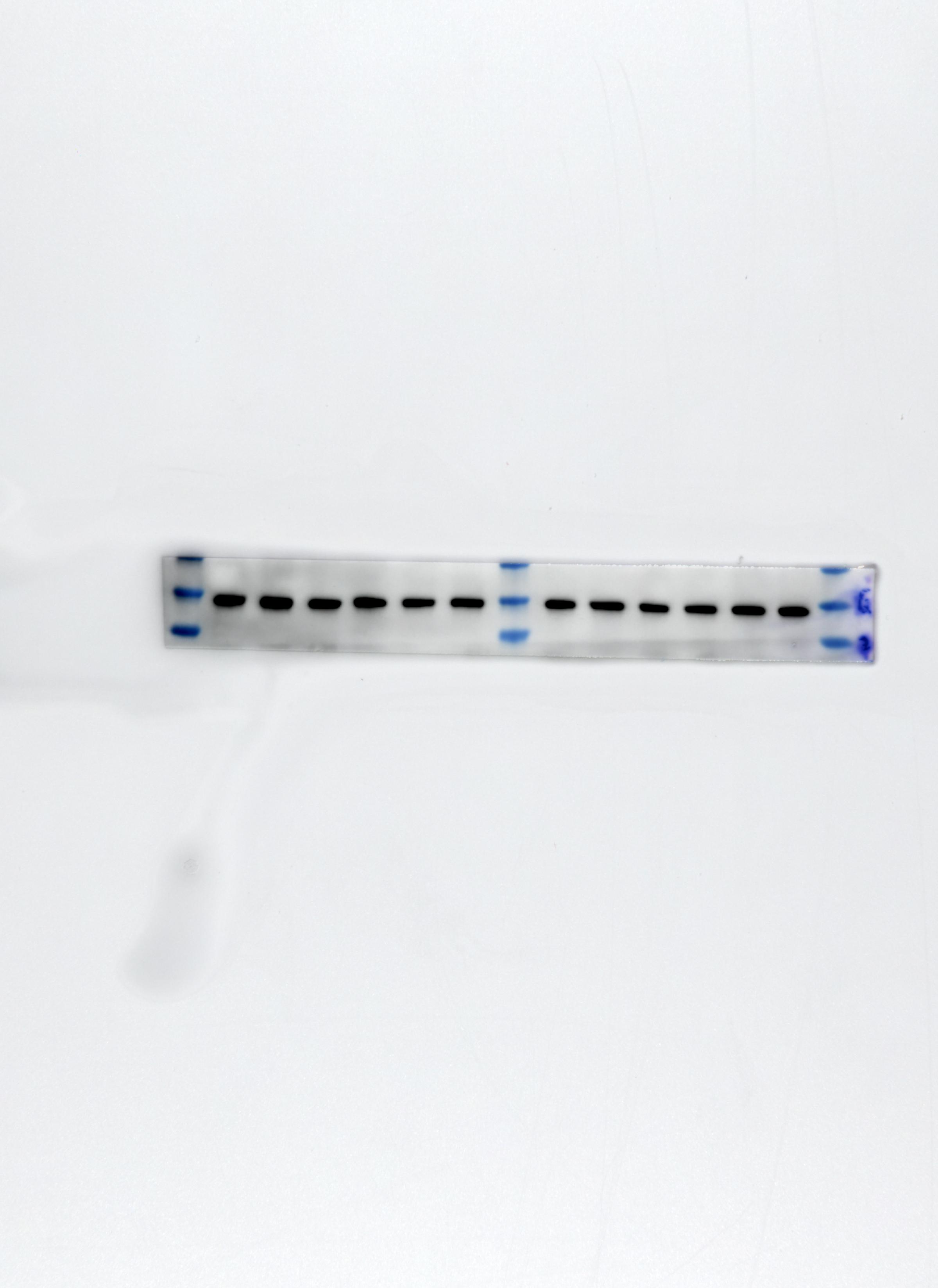

Supplement: Supplementary file 1 [file pharmaceuticals-18-01266-s001.zip › Western blot/IFNGR1/n3(R) [Overlay][GAPDH]-R.jpg]

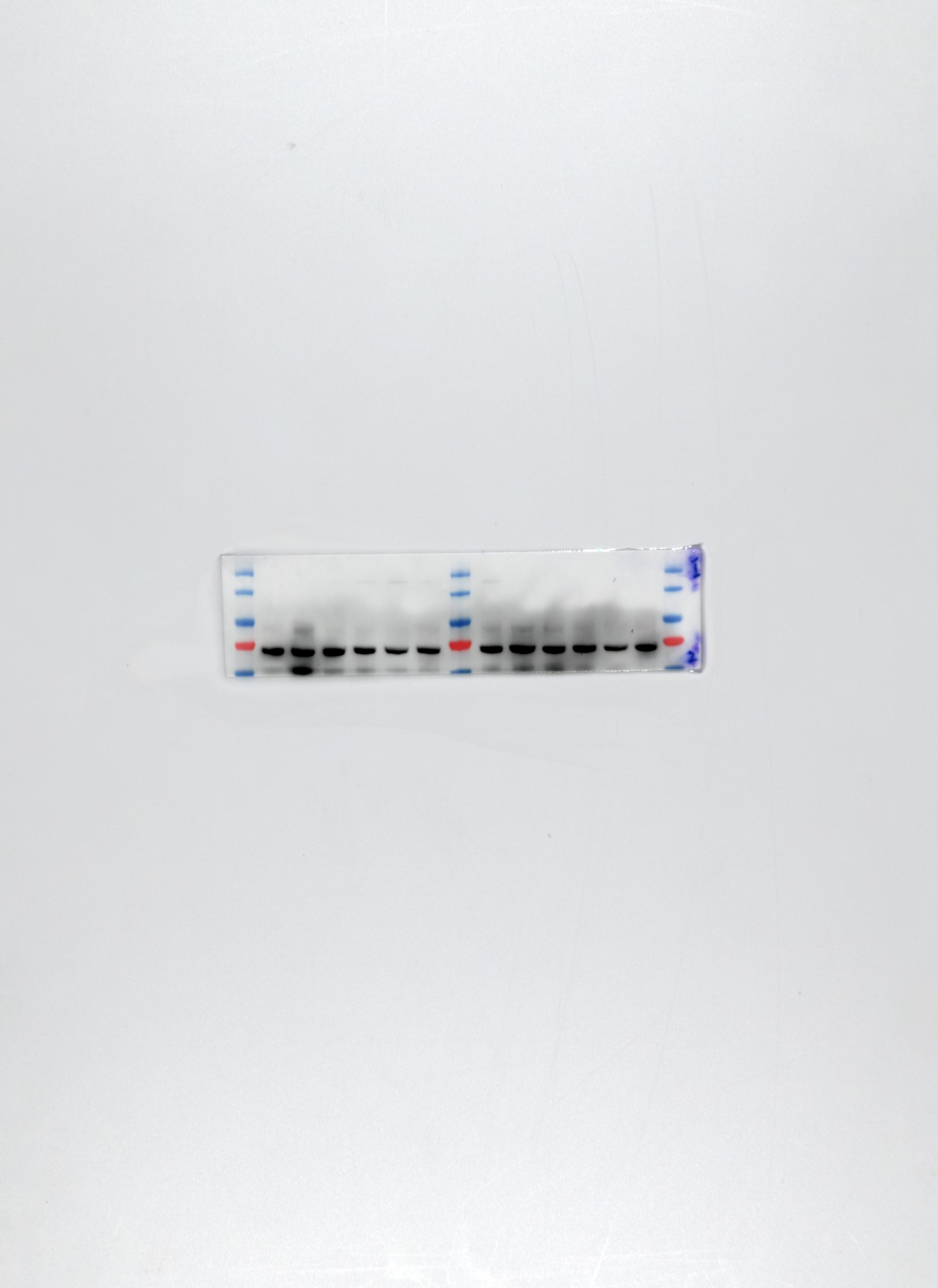

Supplement: Supplementary file 1 [file pharmaceuticals-18-01266-s001.zip › Western blot/IFNGR1/n3(R) [Overlay][IFNGR2]-R.jpg]

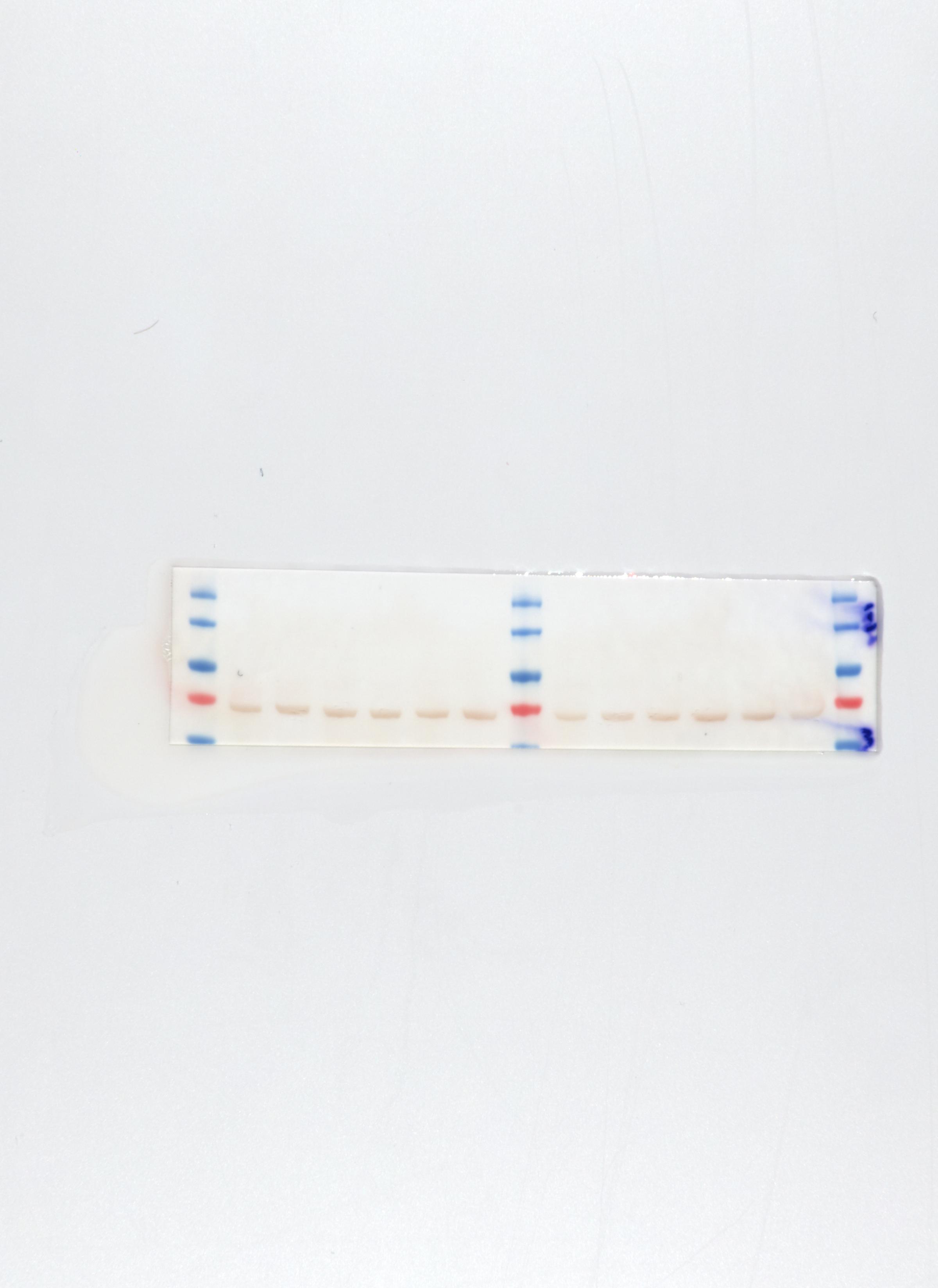

Supplement: Supplementary file 1 [file pharmaceuticals-18-01266-s001.zip › Western blot/IFNGR1/n4-n5 [Brightfield][IFNGR1].jpg]

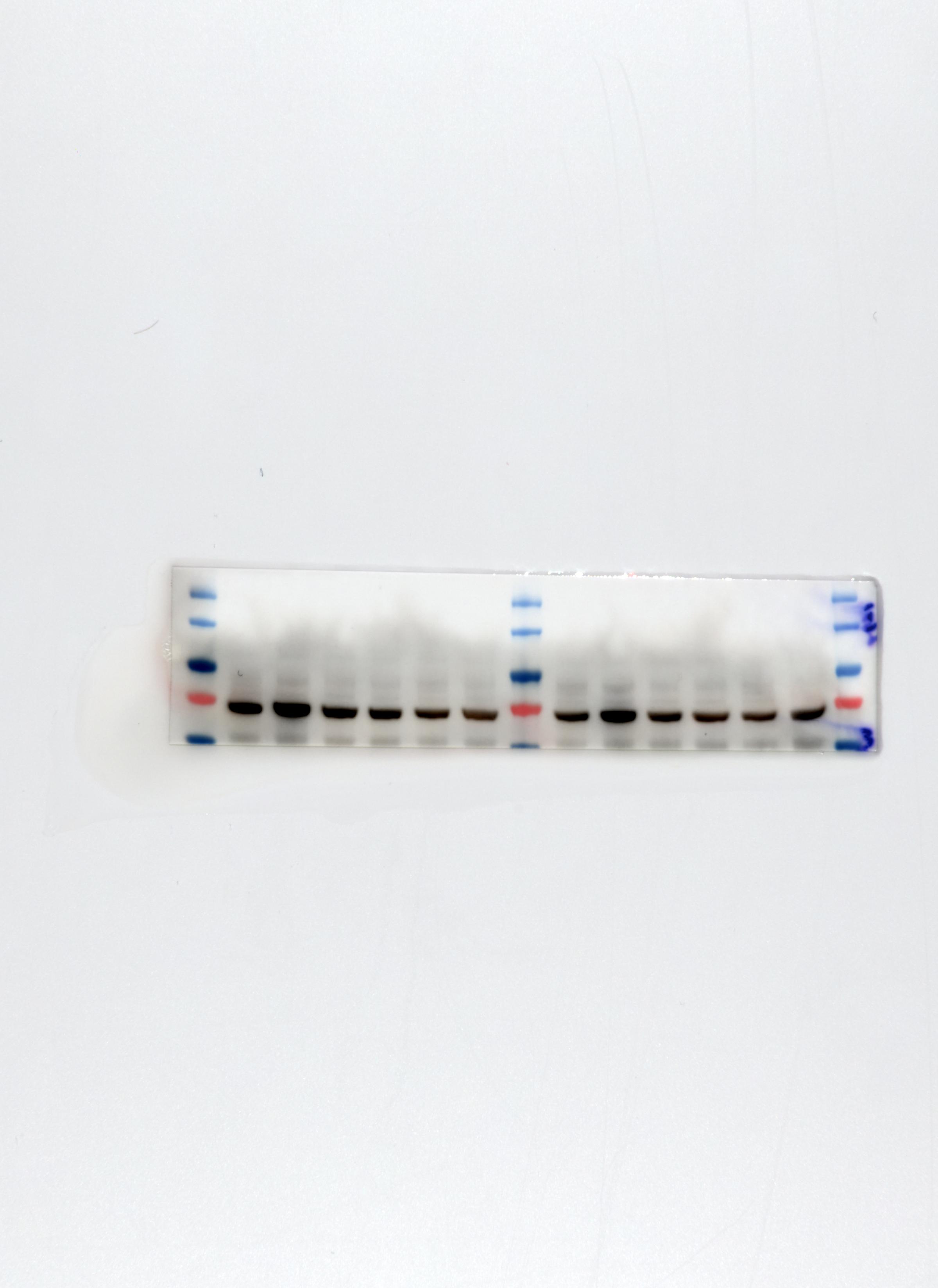

Supplement: Supplementary file 1 [file pharmaceuticals-18-01266-s001.zip › Western blot/IFNGR1/n4-n5 [Overlay][IFNGR1].jpg]

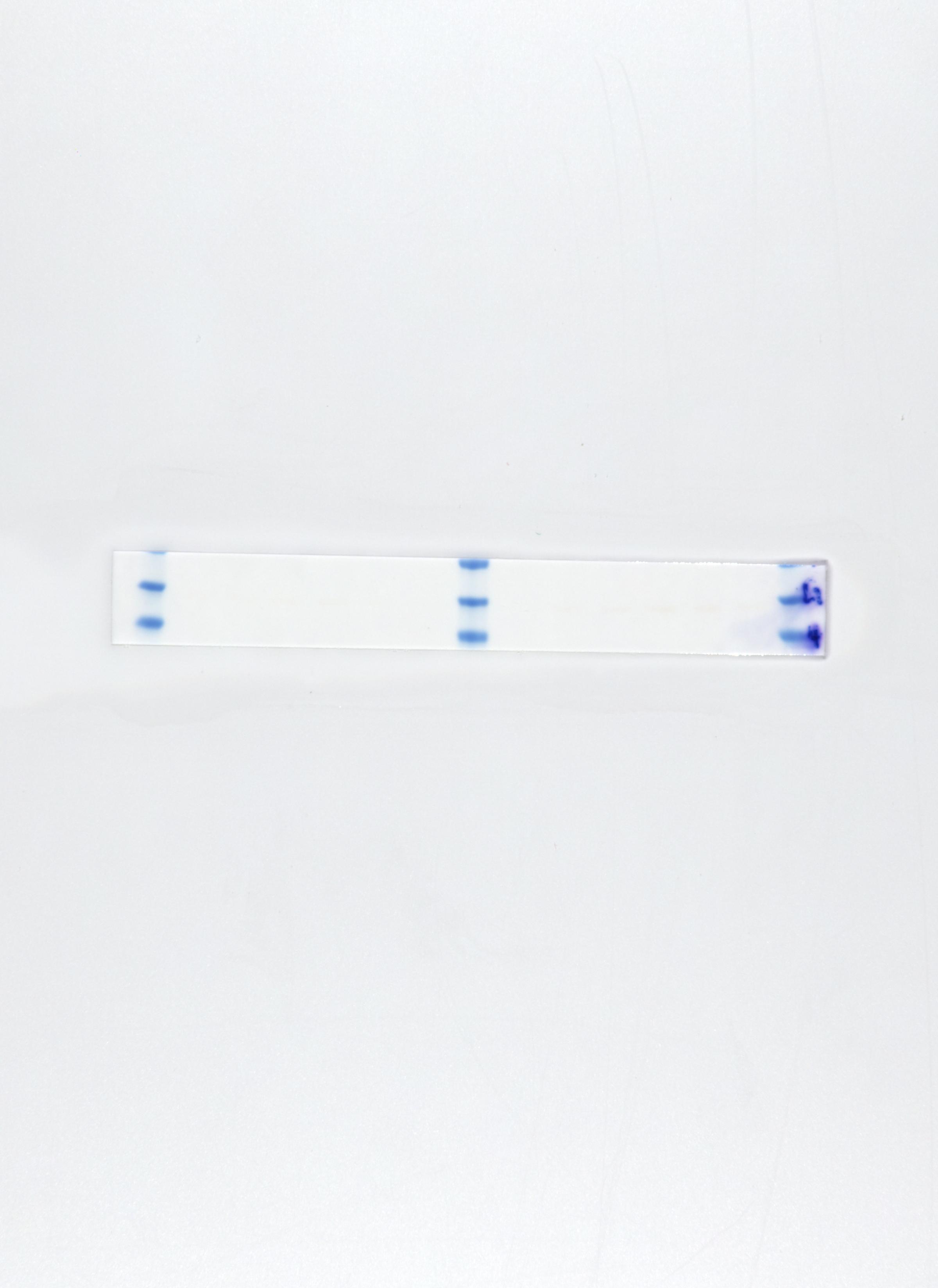

Supplement: Supplementary file 1 [file pharmaceuticals-18-01266-s001.zip › Western blot/IFNGR1/n6(R) [Brightfield][GAPDH]-R.jpg]

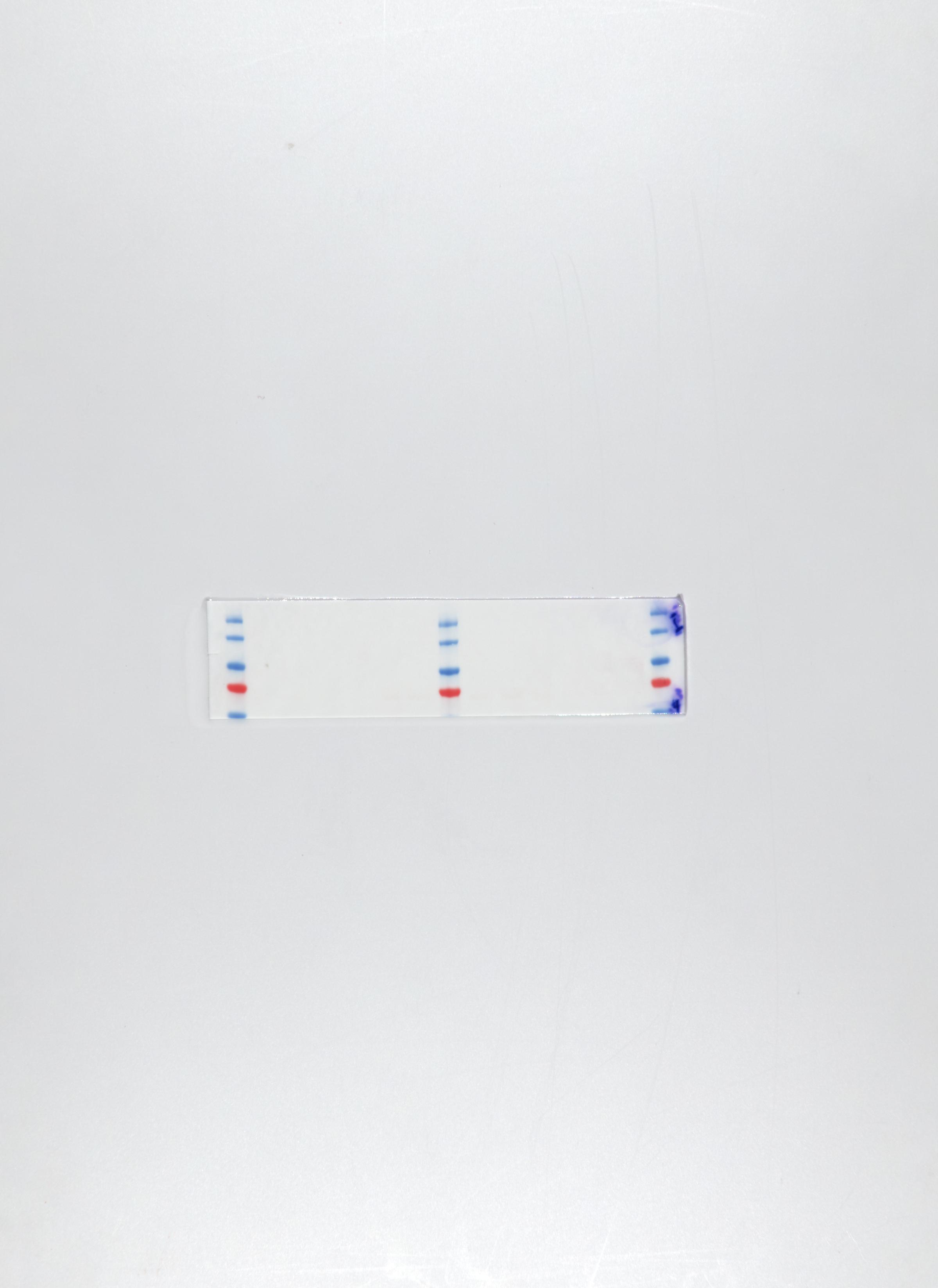

Supplement: Supplementary file 1 [file pharmaceuticals-18-01266-s001.zip › Western blot/IFNGR1/n6(R) [Brightfield][IFNGR1]-R.jpg]

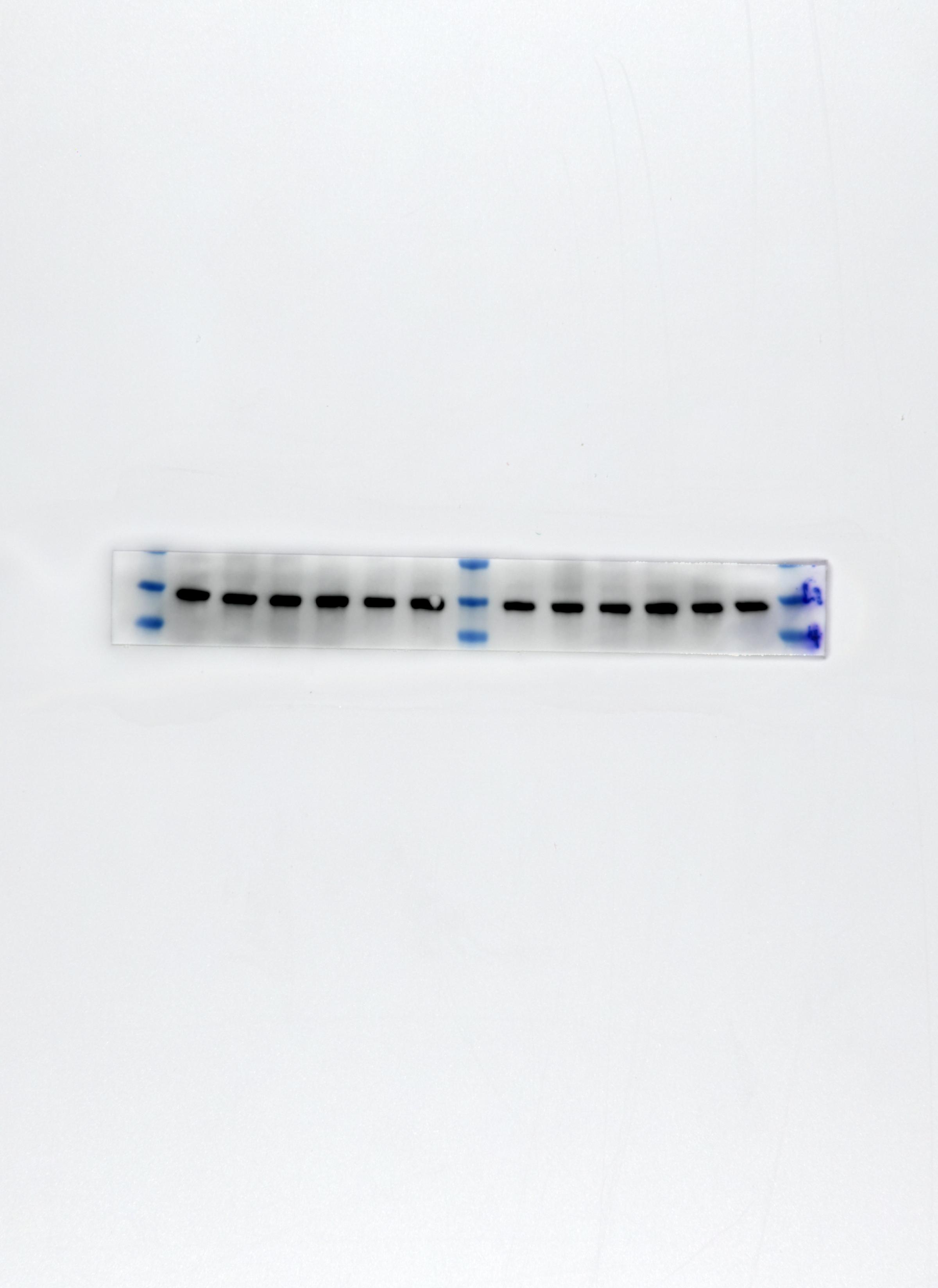

Supplement: Supplementary file 1 [file pharmaceuticals-18-01266-s001.zip › Western blot/IFNGR1/n6(R) [Overlay][GAPDH]-R.jpg]

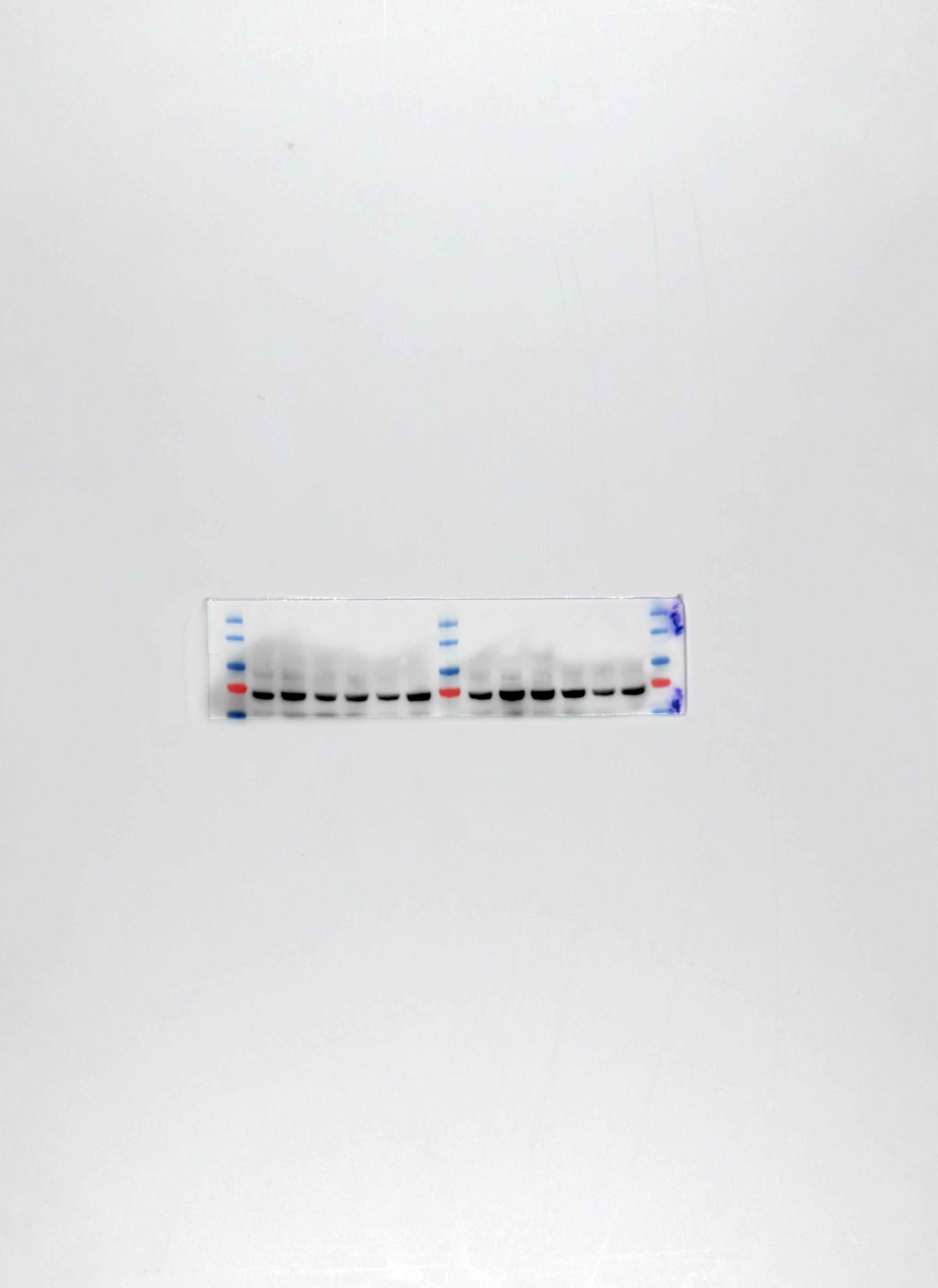

Supplement: Supplementary file 1 [file pharmaceuticals-18-01266-s001.zip › Western blot/IFNGR1/n6(R) [Overlay][IFNGR1]-R.jpg]

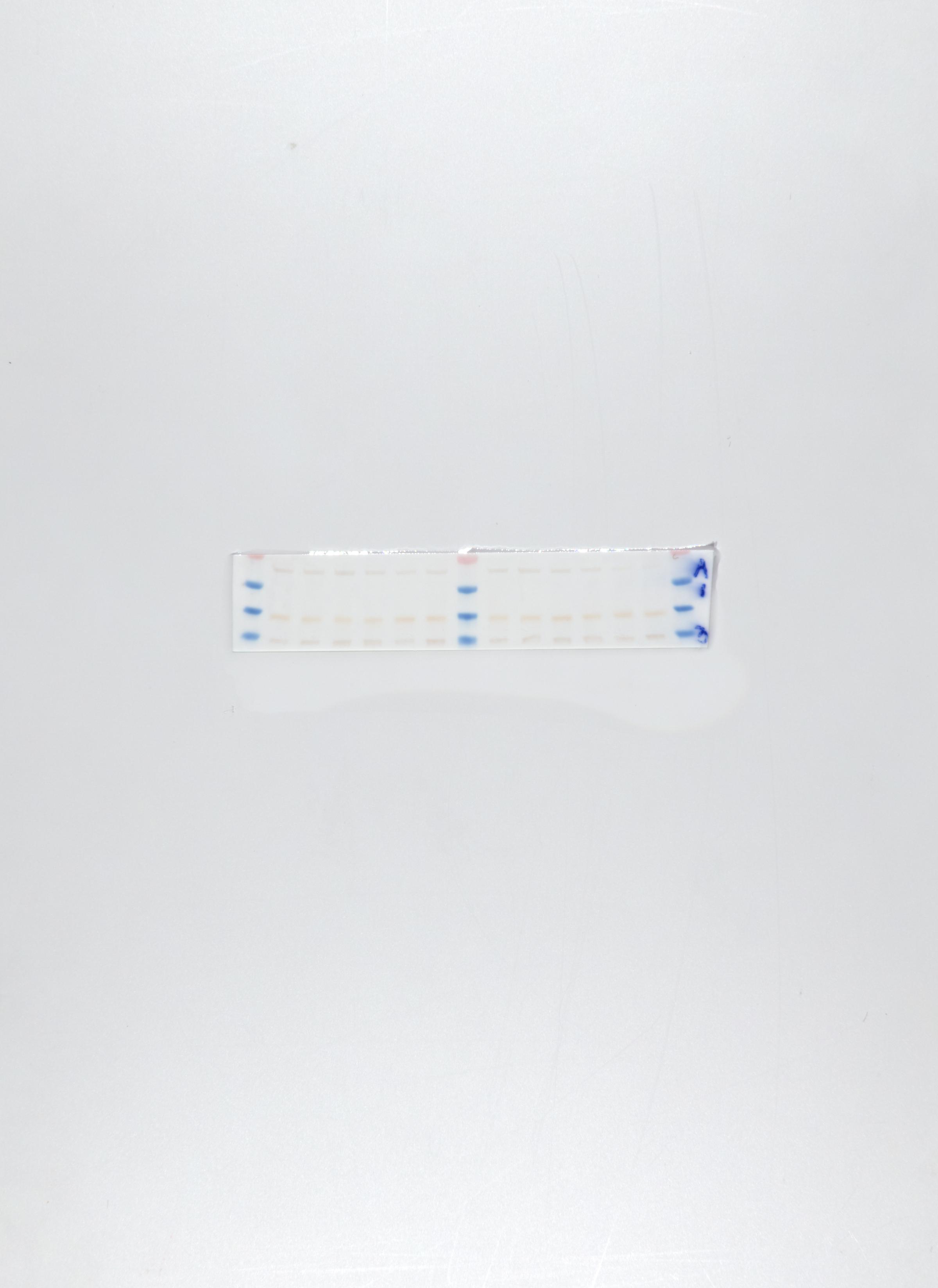

Supplement: Supplementary file 1 [file pharmaceuticals-18-01266-s001.zip › Western blot/JAK1/n1-n2 [Brightfield][GAPDH].jpg]

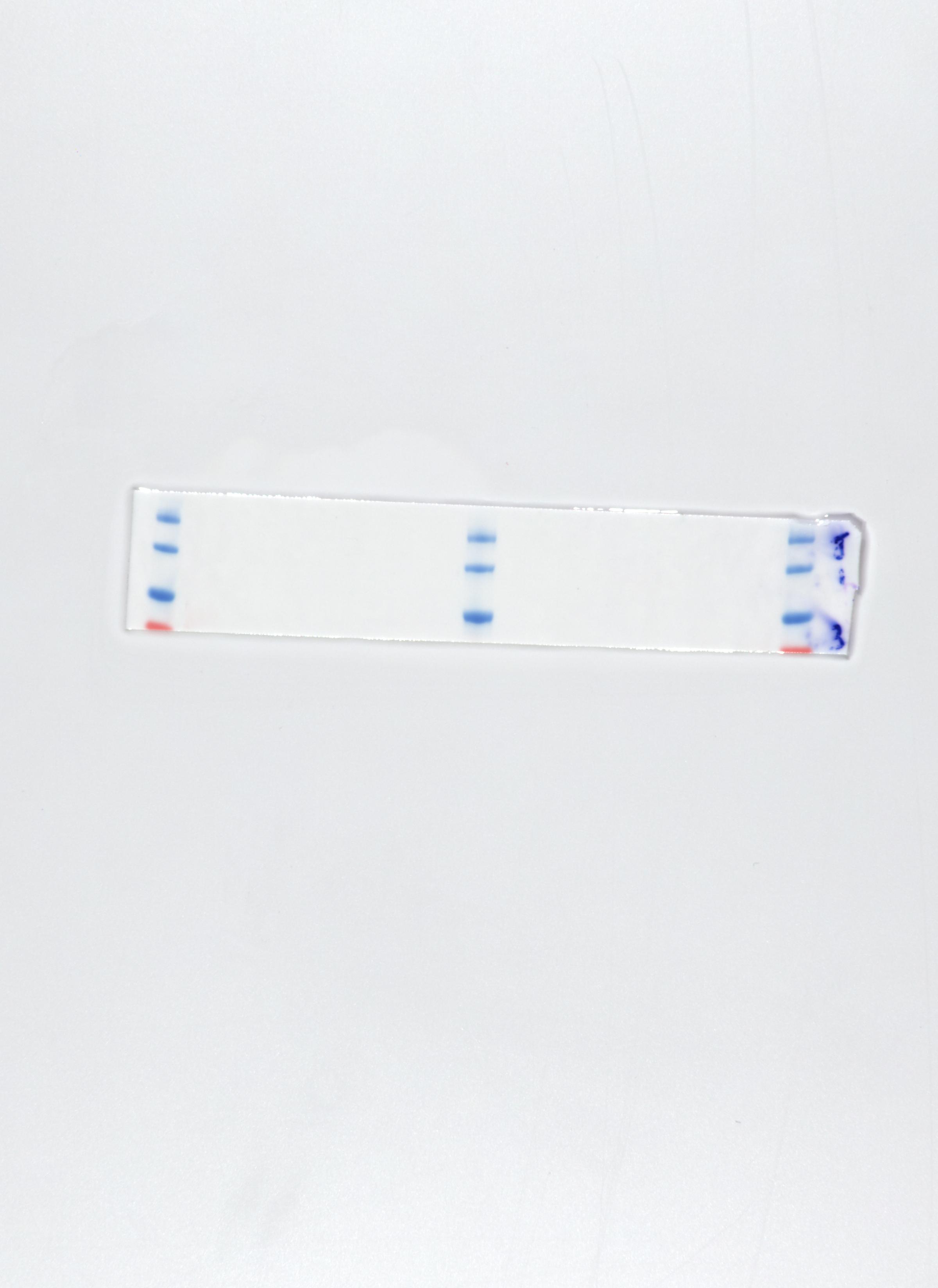

Supplement: Supplementary file 1 [file pharmaceuticals-18-01266-s001.zip › Western blot/JAK1/n1-n2 [Brightfield][JAK1].jpg]

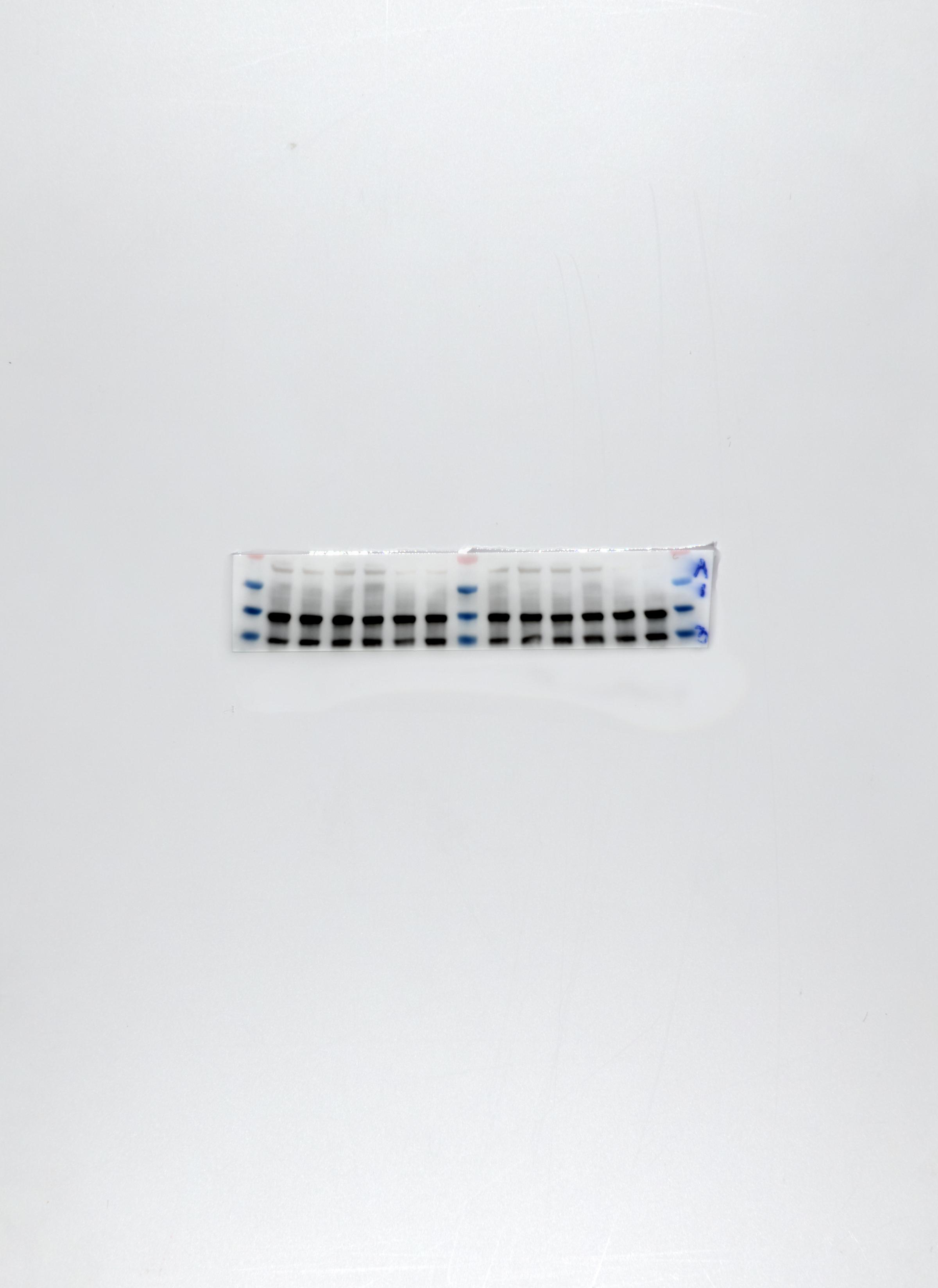

Supplement: Supplementary file 1 [file pharmaceuticals-18-01266-s001.zip › Western blot/JAK1/n1-n2 [Overlay][GAPDH].jpg]

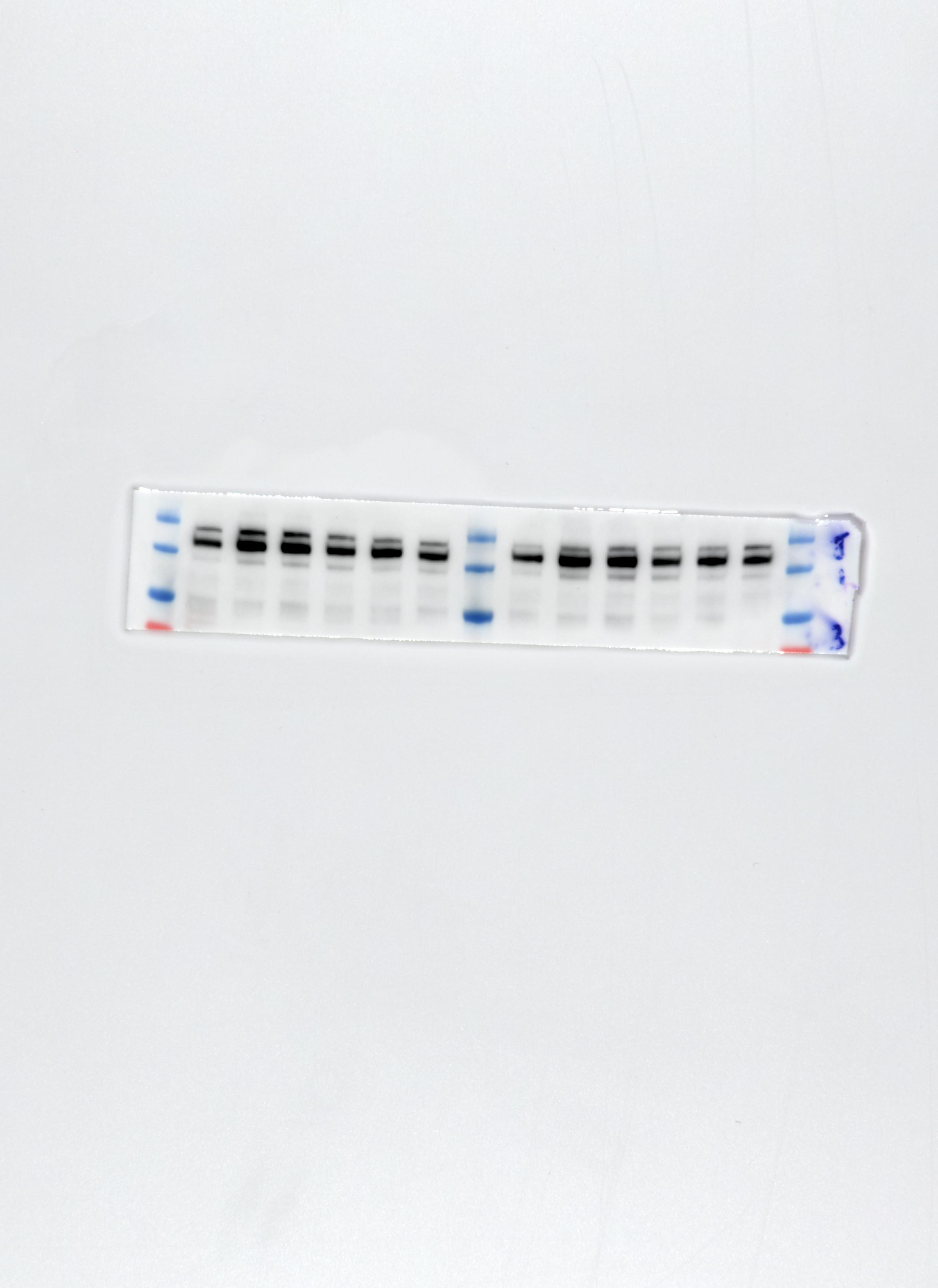

Supplement: Supplementary file 1 [file pharmaceuticals-18-01266-s001.zip › Western blot/JAK1/n1-n2 [Overlay][JAK1].jpg]

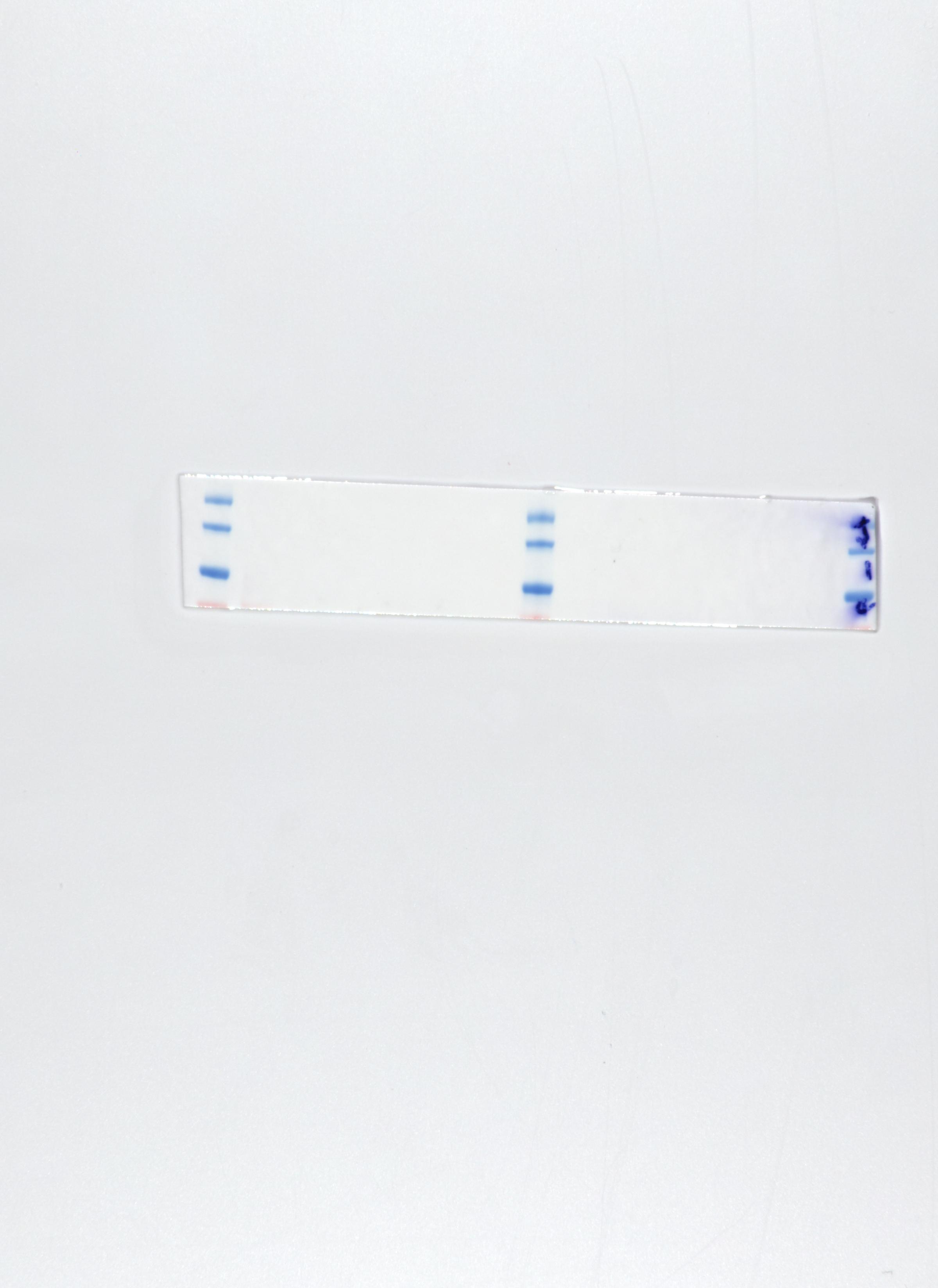

Supplement: Supplementary file 1 [file pharmaceuticals-18-01266-s001.zip › Western blot/JAK1/n3-n4 [Brightfield][JAK1].jpg]

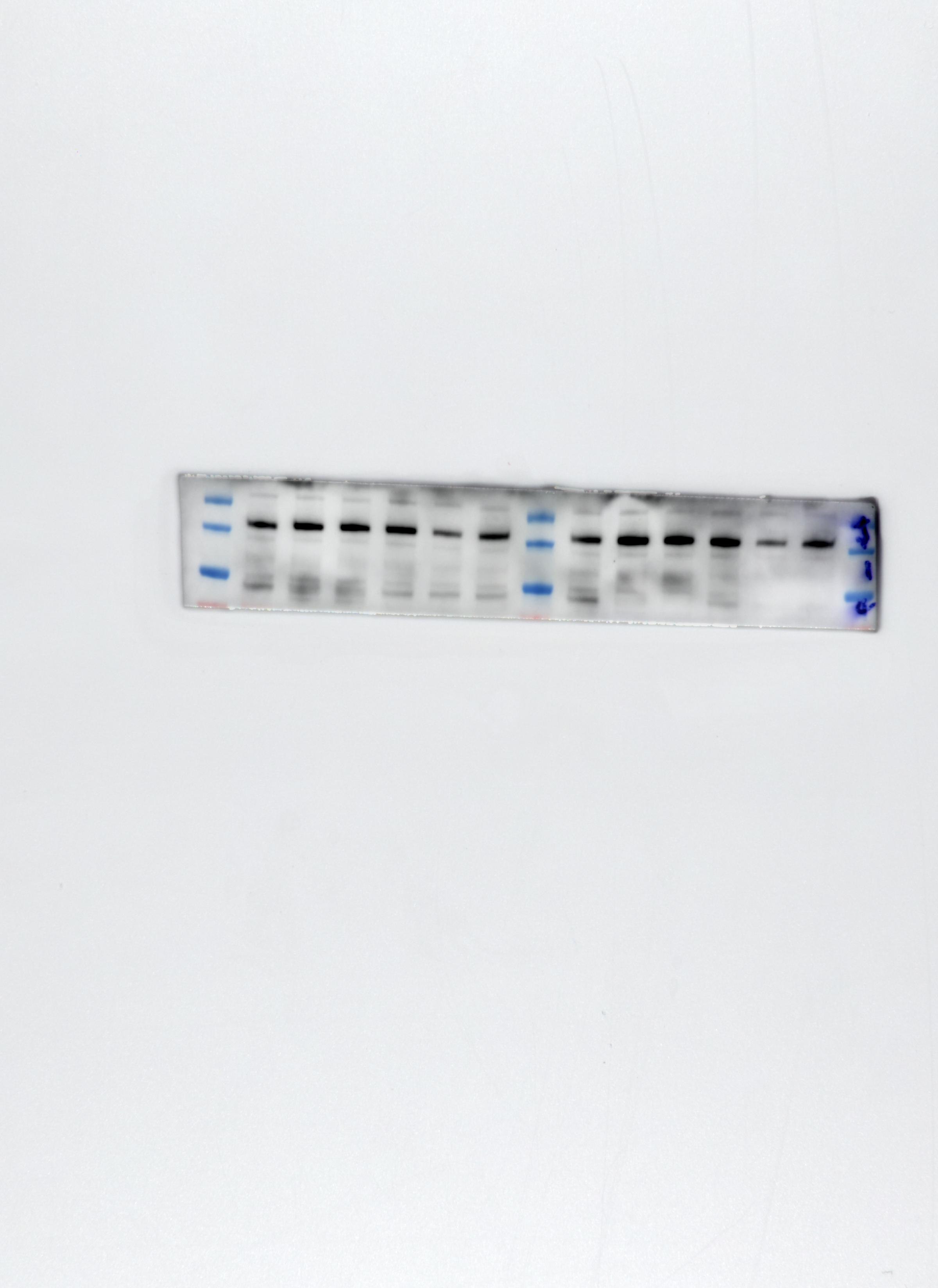

Supplement: Supplementary file 1 [file pharmaceuticals-18-01266-s001.zip › Western blot/JAK1/n3-n4 [Overlay][JAK1].jpg]

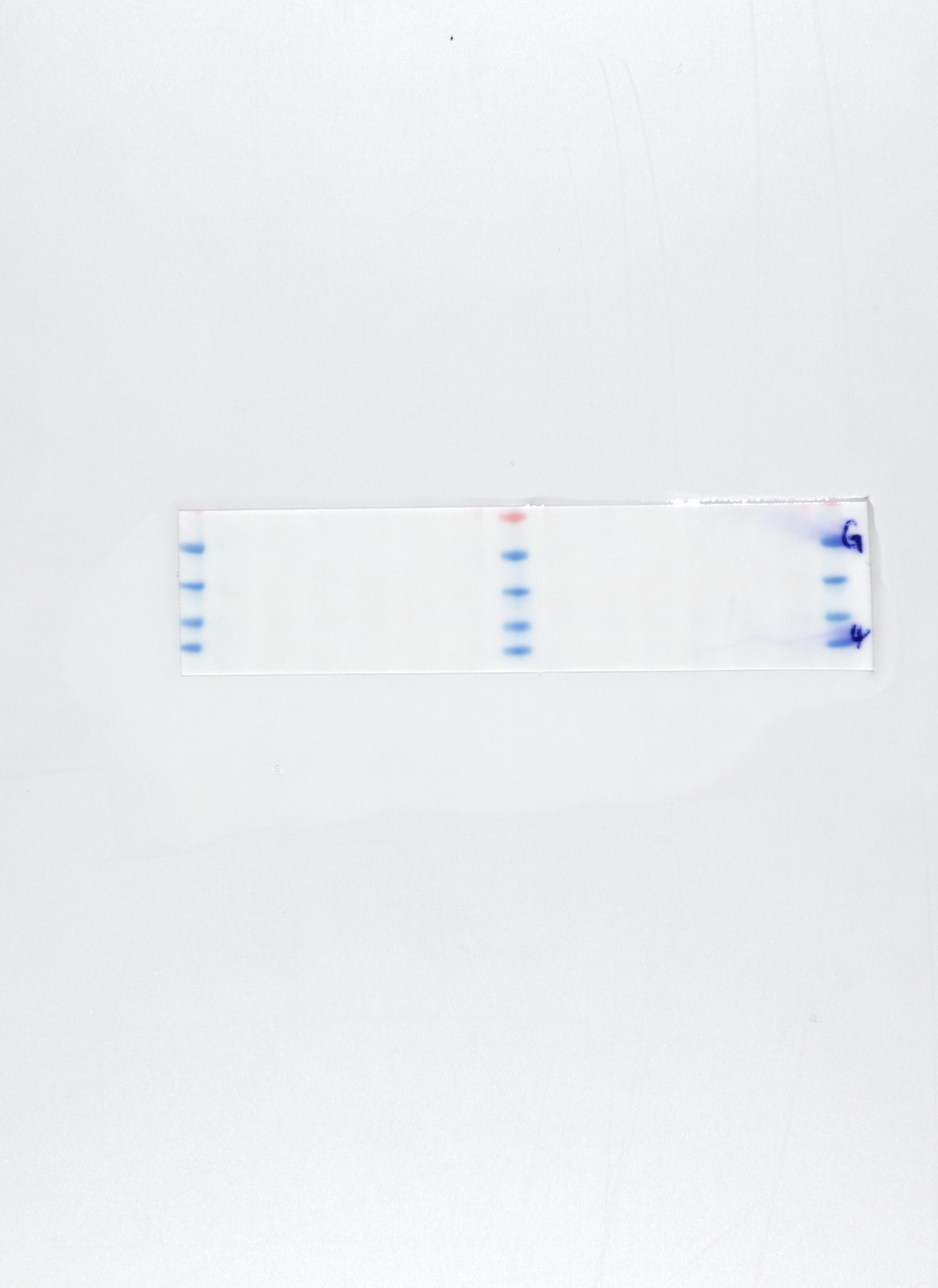

Supplement: Supplementary file 1 [file pharmaceuticals-18-01266-s001.zip › Western blot/JAK1/n5-n6 [Brightfield][GAPDH].jpg]

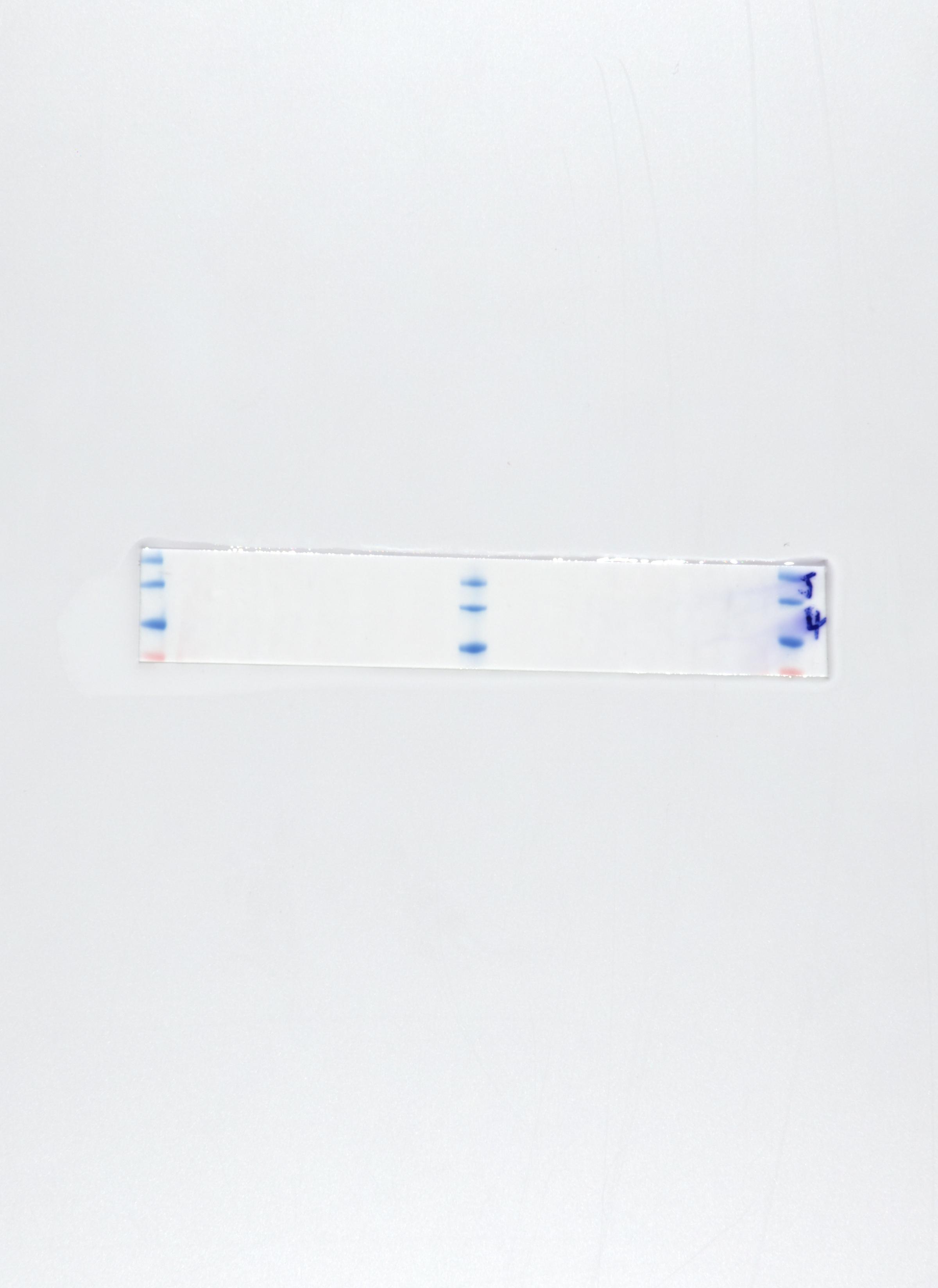

Supplement: Supplementary file 1 [file pharmaceuticals-18-01266-s001.zip › Western blot/JAK1/n5-n6 [Brightfield][JAK1].jpg]

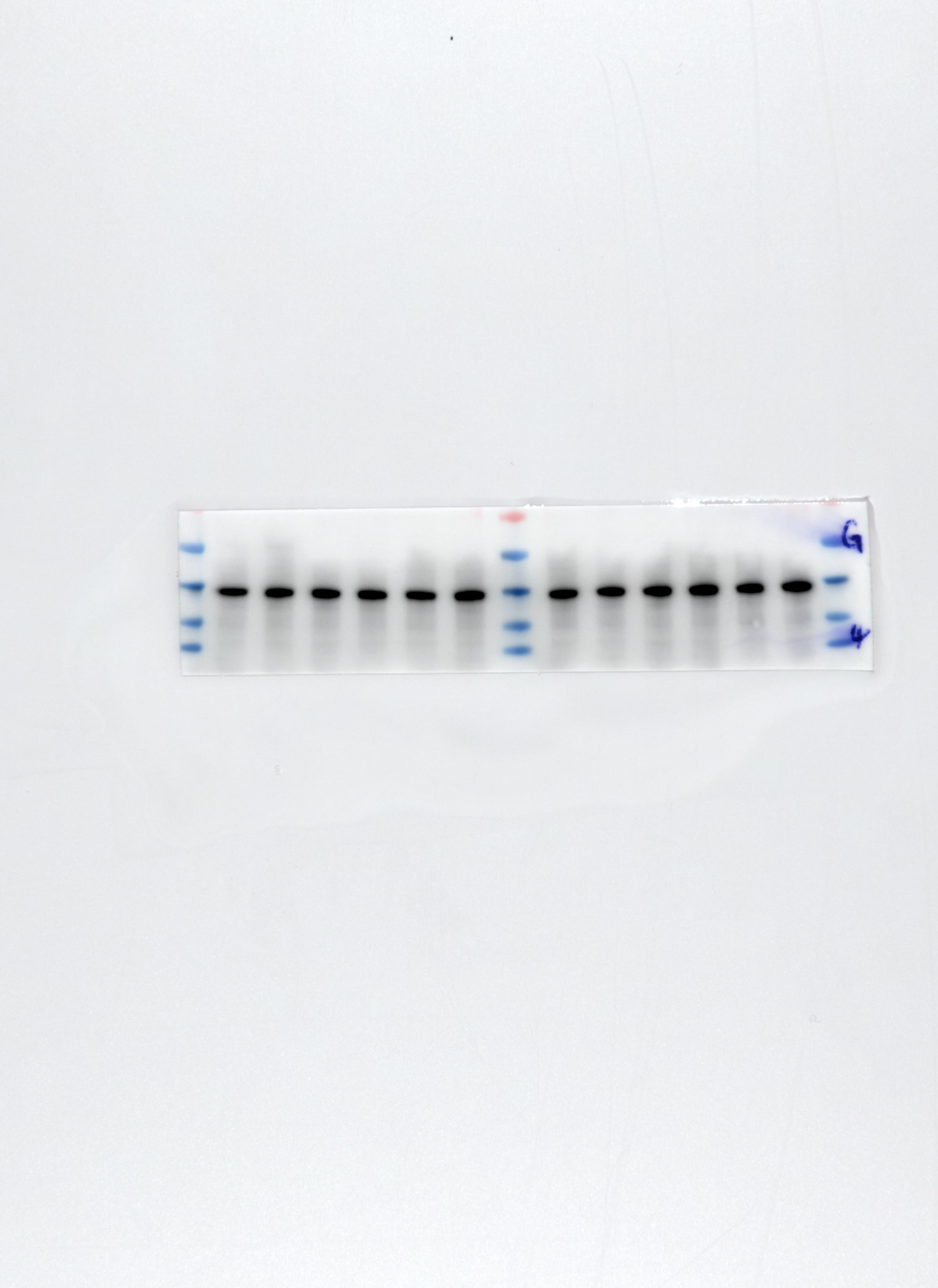

Supplement: Supplementary file 1 [file pharmaceuticals-18-01266-s001.zip › Western blot/JAK1/n5-n6 [Overlay][IFNG].tif.jpg]

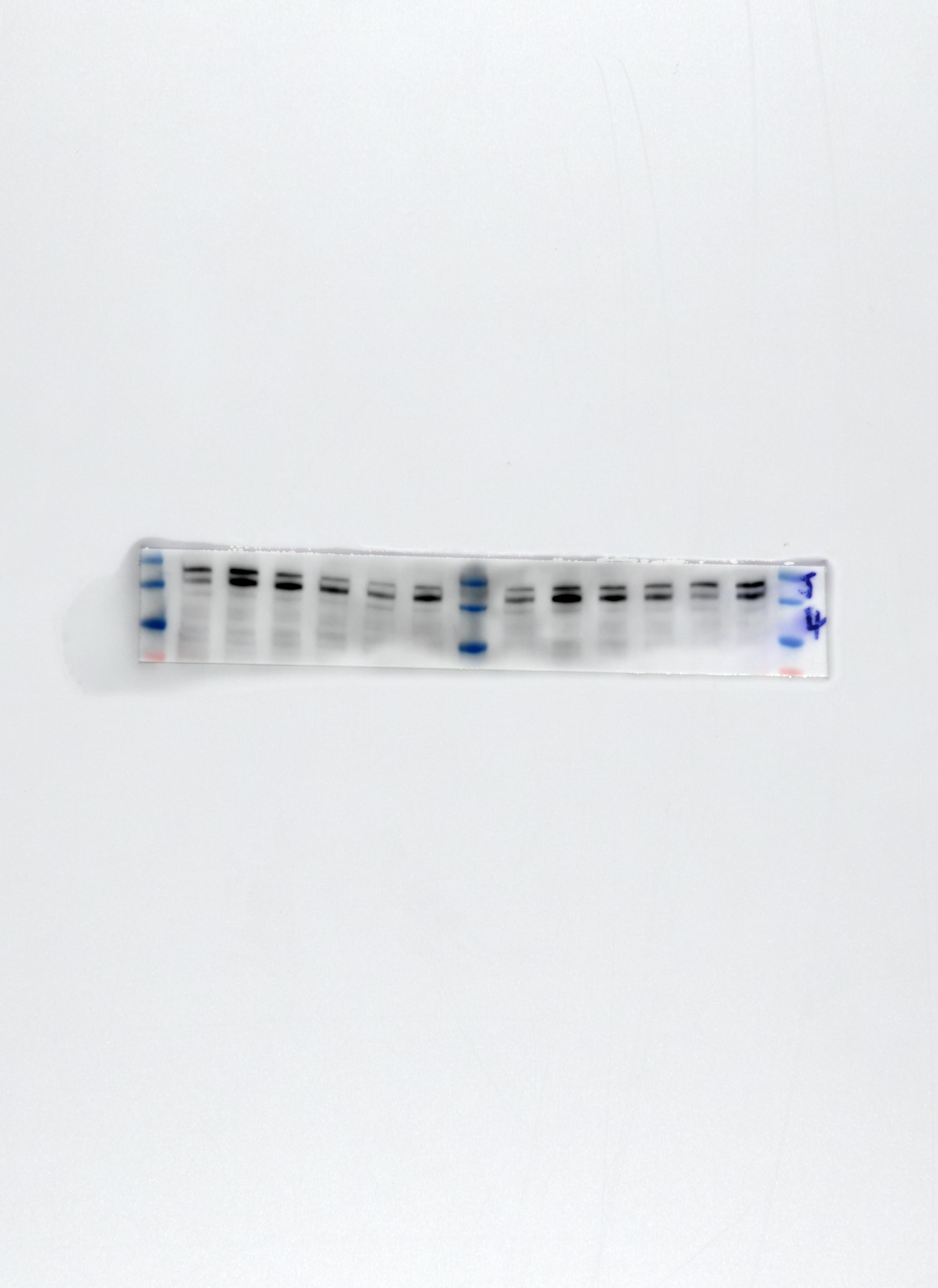

Supplement: Supplementary file 1 [file pharmaceuticals-18-01266-s001.zip › Western blot/JAK1/n5-n6 [Overlay][JAK1].tif.jpg]

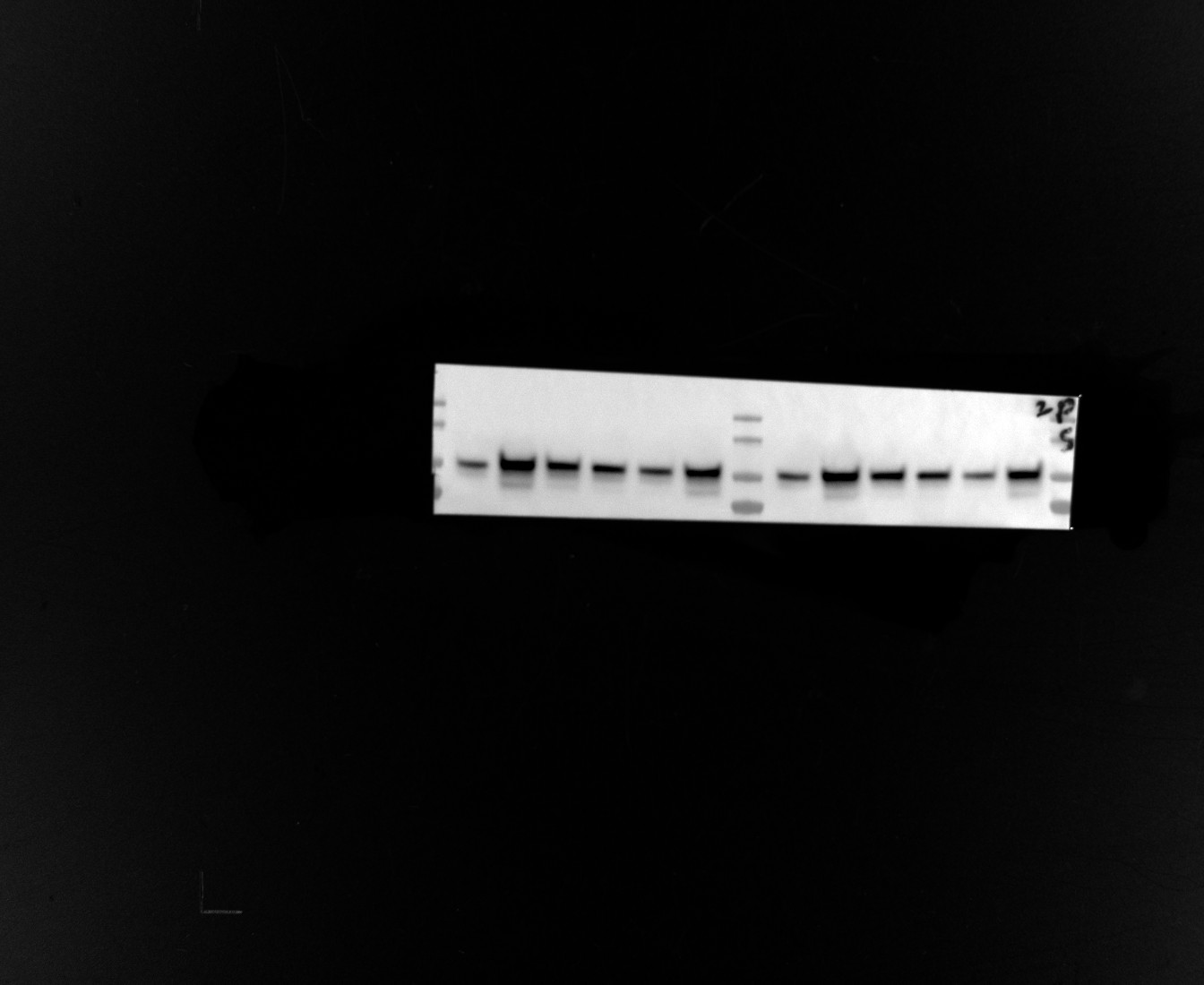

Supplement: Supplementary file 1 [file pharmaceuticals-18-01266-s001.zip › Western blot/p-STAT3/3. [Overlay][p-STAT3].tif]

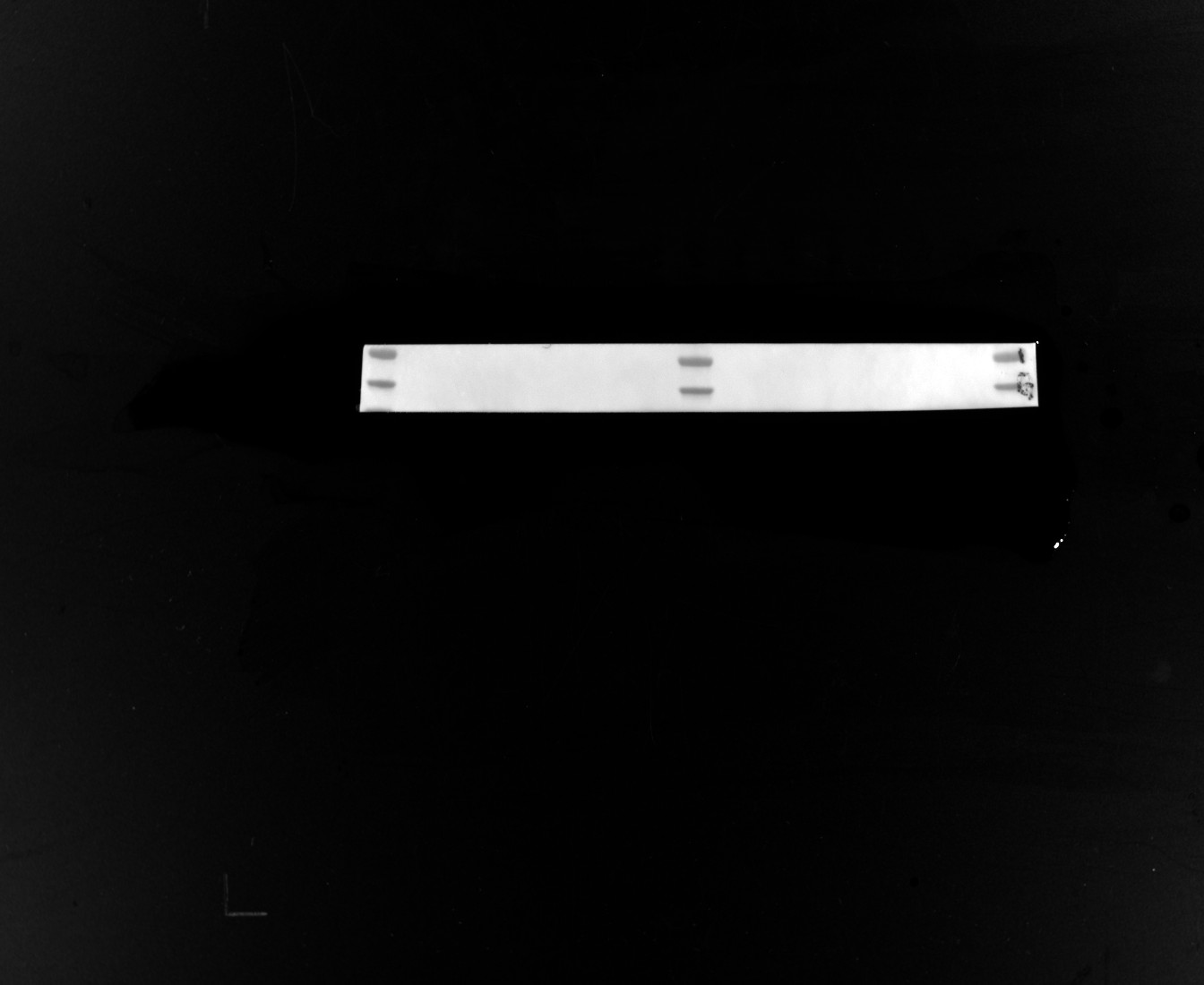

Supplement: Supplementary file 1 [file pharmaceuticals-18-01266-s001.zip › Western blot/p-STAT3/n1-n2 [Brightfield][GAPDH].tif]

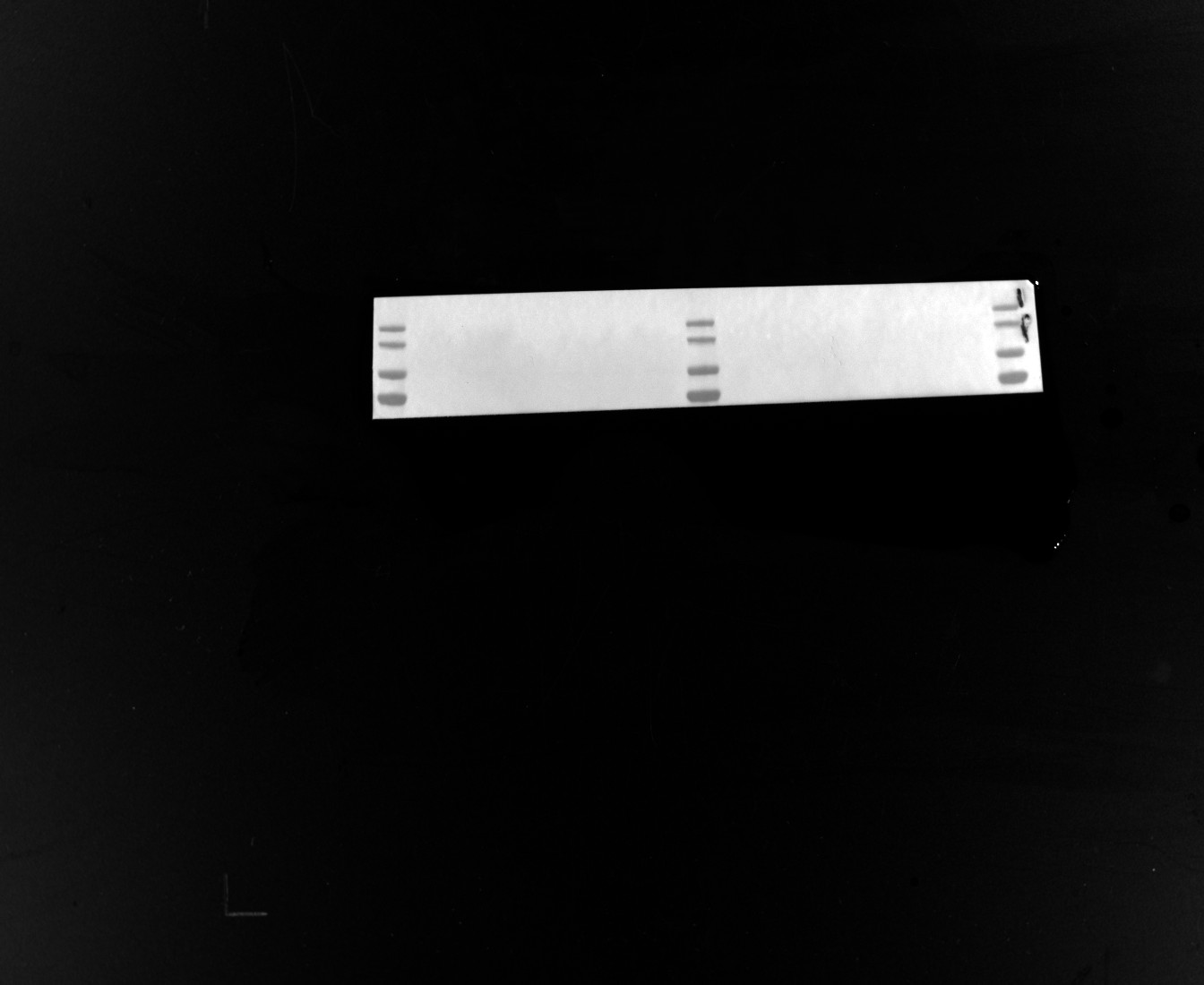

Supplement: Supplementary file 1 [file pharmaceuticals-18-01266-s001.zip › Western blot/p-STAT3/n1-n2 [Brightfield][p-STAT3].tif]

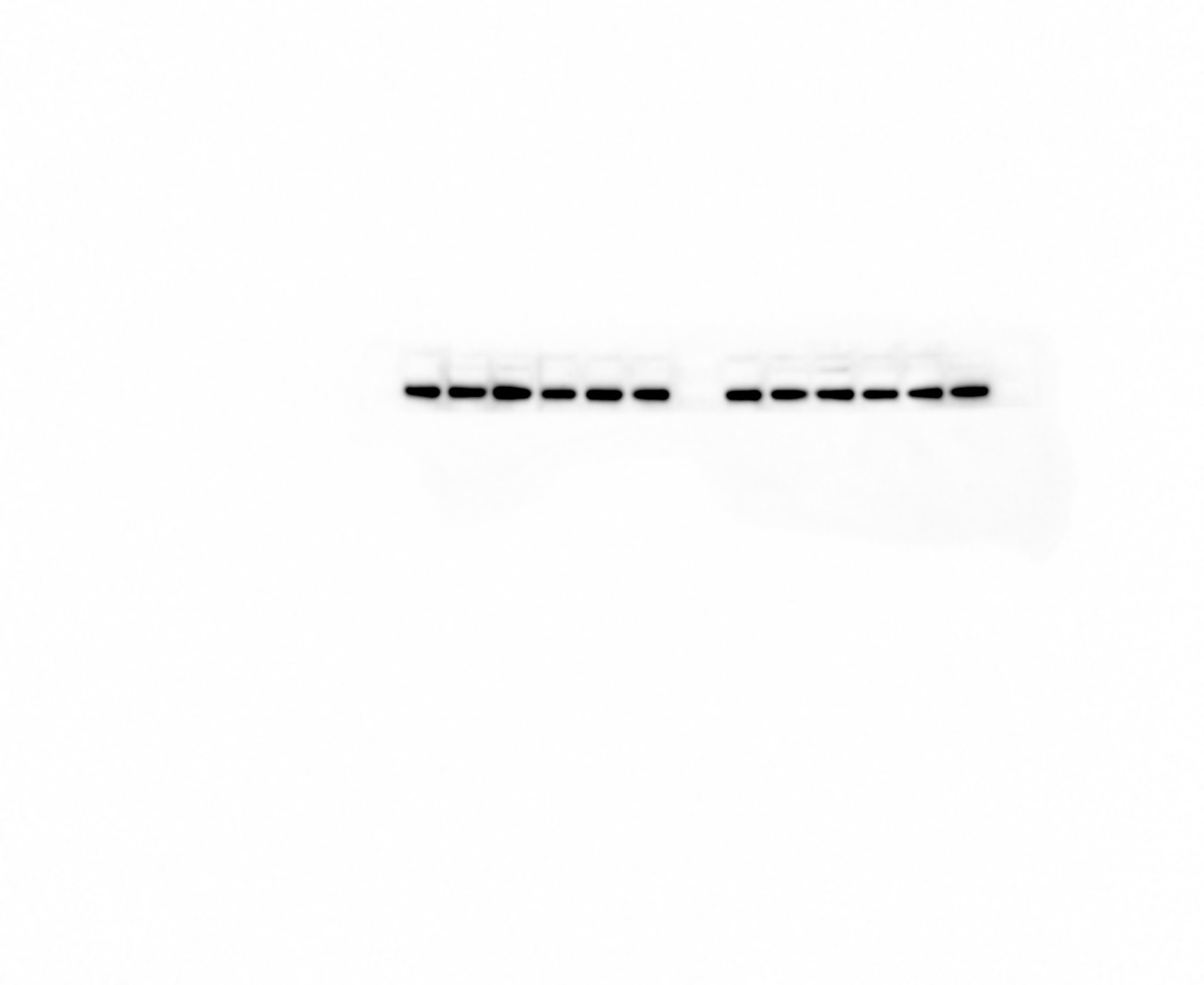

Supplement: Supplementary file 1 [file pharmaceuticals-18-01266-s001.zip › Western blot/p-STAT3/n1-n2 [Luminescence][GAPDH].tif]
